# Transforming and evaluating the UK Biobank to the OMOP Common Data Model for COVID-19 research and beyond

**DOI:** 10.1093/jamia/ocac203

**Published:** 2022-10-13

**Authors:** Vaclav Papez, Maxim Moinat, Erica A Voss, Sofia Bazakou, Anne Van Winzum, Alessia Peviani, Stefan Payralbe, Elena Garcia Lara, Michael Kallfelz, Folkert W Asselbergs, Daniel Prieto-Alhambra, Richard J B Dobson, Spiros Denaxas

**Affiliations:** Institute of Health Informatics, University College London, London, UK; Health Data Research UK, London, UK; The Hyve, Utrecht, The Netherlands; Erasmus Medical Center Rotterdam, Rotterdam, The Netherlands; Department of Epidemiology, Janssen Research & Development LLC, Raritan, New Jersey, USA; The Hyve, Utrecht, The Netherlands; The Hyve, Utrecht, The Netherlands; The Hyve, Utrecht, The Netherlands; The Hyve, Utrecht, The Netherlands; The Hyve, Utrecht, The Netherlands; Odysseus Data Services GmbH, Berlin, Germany; Institute of Health Informatics, University College London, London, UK; Health Data Research UK, London, UK; Amsterdam University Medical Centers, Department of Cardiology, University of Amsterdam, Amsterdam, The Netherlands; Erasmus Medical Center Rotterdam, Rotterdam, The Netherlands; Centre for Statistics in Medicine, NDORMS, University of Oxford, Oxford, UK; Institute of Health Informatics, University College London, London, UK; Health Data Research UK, London, UK; Department of Biostatistics and Health Informatics, Institute of Psychiatry, Psychology and Neuroscience (IoPPN), King’s College London, London, UK; Institute of Health Informatics, University College London, London, UK; Health Data Research UK, London, UK; British Heart Foundation Data Science Center, London, UK; UCL Hospitals, NIHR Biomedical Research Centre (BRC), London, UK

**Keywords:** electronic health records, medical ontologies, phenotyping, OMOP, common data model

## Abstract

**Objective:**

The coronavirus disease 2019 (COVID-19) pandemic has demonstrated the value of real-world data for public health research. International federated analyses are crucial for informing policy makers. Common data models (CDMs) are critical for enabling these studies to be performed efficiently. Our objective was to convert the UK Biobank, a study of 500 000 participants with rich genetic and phenotypic data to the Observational Medical Outcomes Partnership (OMOP) CDM.

**Materials and Methods:**

We converted UK Biobank data to OMOP CDM v. 5.3. We transformedparticipant research data on diseases collected at recruitment and electronic health records (EHRs) from primary care, hospitalizations, cancer registrations, and mortality from providers in England, Scotland, and Wales. We performed syntactic and semantic validations and compared comorbidities and risk factors between source and transformed data.

**Results:**

We identified 502 505 participants (3086 with COVID-19) and transformed 690 fields (1 373 239 555 rows) to the OMOP CDM using 8 different controlled clinical terminologies and bespoke mappings. Specifically, we transformed self-reported noncancer illnesses 946 053 (83.91% of all source entries), cancers 37 802 (70.81%), medications 1 218 935 (88.25%), and prescriptions 864 788 (86.96%). In EHR, we transformed 13 028 182 (99.95%) hospital diagnoses, 6 465 399 (89.2%) procedures, 337 896 333 primary care diagnoses (CTV3, SNOMED-CT), 139 966 587 (98.74%) prescriptions (dm+d) and 77 127 (99.95%) deaths (ICD-10). We observed good concordance across demographic, risk factor, and comorbidity factors between source and transformed data.

**Discussion and Conclusion:**

Our study demonstrated that the OMOP CDM can be successfully leveraged to harmonize complex large-scale biobanked studies combining rich multimodal phenotypic data. Our study uncovered several challenges when transforming data from questionnaires to the OMOP CDM which require further research. The transformed UK Biobank resource is a valuable tool that can enable federated research, like COVID-19 studies.

## INTRODUCTION

The coronavirus disease 2019 (COVID-19) pandemic has had a profound worldwide impact on disease and healthcare system burden.^1^ Disease severity, and interactions with the healthcare system, have been highly heterogeneous between pandemic waves and viral variants.^2^ Rapidly evolving Severe acute respiratory syndrome coronavirus 2 (SARS-CoV-2) testing patterns and clinical guidelines also meant that patients demonstrate different clinical trajectories and interact with the healthcare system in different ways. Finally, the widespread and sustained worldwide uptake of vaccinations has a huge impact in terms of patient outcomes but also raised concerns in terms of adverse reactions (eg, thrombocytopenic events following ChAdOx1/BNT162b2).^3^

During the COVID-19 pandemic, there has been a critical need for generating and providing real world high quality scientific evidence to clinicians and policy makers on COVID-19 phenotypes, treatments and prognosis. Many aspects of COVID-19 vary significantly across healthcare systems across countries and international comparisons on patients’ outcomes are vital to understand the reasons for this variability.^4^ Furthermore, rare COVID-19 vaccination side effects require multiple datasets to be analyzed given their very low prevalence in any individual source. Performing federated analyses across different datasets is challenging as data are recorded in different clinical terminologies, are generated for different purposes, and use different schemas and multiple large-scale collaborations, such as the National COVID Cohort Collaborative (N3C) were created for this purpose.^5^ Observational Health Data Sciences and Informatics (OHDSI) is an international research network aiming to generate reliable and high-quality clinical evidence for improving health and healthcare. During the COVID-19 pandemic, OHDSI used the Observational Medical Outcomes Partnership (OMOP) Common Data Model (CDM)^9^ to transform disparate data into a standardized format and perform federated analyses to produce high quality evidence about COVID-19 for policy makers and healthcare providers.^4^^,^^6–8^

The IMI European Health Data Evidence Network (EHDEN) project^10^ launched a series of Rapid Collaboration Calls in order to catalyze the conversion of datasets to OMOP with the aim of providing insights into COVID-19 rapidly and at scale across countries. As part of this initiative, we have transformed the UK Biobank (UKB), one of the world’s largest prospective longitudinal studies of 500 000 individuals with extensive genotypic (eg, GWAS, WES, WGS), phenotypic (eg, electronic health records [EHRs] linkages to primary care, hospitalizations, cancer registrations, mortality, etc.) and questionnaire information, to the OMOP CDM.

The objective of our work was to transform the UKB into the OMOP CDM and facilitate international collaboration on COVID-19 research and beyond. The primary aim of our study was to convert the UKB and linked EHR data into the OMOP CDM and evaluate the results from a syntactic and semantic perspective.

## MATERIALS AND METHODS

### Data sources

The UKB recruited 500 000 individuals (aged 40–69 years at recruitment) from England, Scotland, and Wales. UKB participants have extensive phenotyping and genotypic information collected.^11^ The study includes genome-wide genetic data on ∼488 000 participants including imputed genotype data, whole exome-sequencing and whole-genome sequencing.

All participants attended an initial assessment center visit (2006–2010), and a smaller subset were invited for repeat assessments or deeper phenotyping (eg, multimodal imaging). Phenotypic data can be categorized as: (1) research-collected data at the point of recruitment (baseline data), and (2) longitudinal health information from EHR and disease registry sources.

#### Baseline data fields

Baseline data contain a wealth of information which was collected during the baseline assessment of participants in the UKB clinics. These include: (1) participant current and past self-reported illnesses, medications and procedures which were then verified by a clinical research nurse, (2) detailed socio-demographic and lifestyle risk factor data, (3) extensive blood, saliva, and urine biomarkers, and (4) anthropometric measurements including data from multiple modalities on particular aspects of human health (eg, spirometry, bone density, eye and hearing tests, etc.). Baseline data fields are recorded using a bespoke coding system developed by the UKB, eg, field 20002 contains patient reported noncancer diseases which are encoded using 474 unique numeric codes.^12^

#### EHR linkages

Longitudinal health outcomes for study participants is collected by linking with national EHR, administrative and disease registry sources in each of the 3 countries that participants were recruited from. Specifically, these include information across these domains: (1) EHR data from primary care healthcare providers, (2) administrative data for hospital admissions, (3) cancer registration information, and (4) mortality data. Each country records data across these domains in unique data providers which use different clinical terminologies and have variable follow up times. Datasets were linked using the NHS number, a unique 10 digital healthcare-specific identifier assigned at first interaction with the healthcare system.

Primary care data are collected from English, Scottish, and Welsh general practitioner (GP) practices that make use of the EMIS (https://www.emishealth.com/), Vision (https://www.visionhealth.co.uk/), or TPP (https://www.tpp-uk.com/) primary care information systems. Data are recorded using 3 different controlled clinical terminologies: (1) SNOMED-CT^13^; (2) Clinical Terms Version 3 (CTV3)^14^; and (3) the Dictionary of Medicines and Devices (dm+d).^15^ CTV3 is part of the SNOMED Clinical Terms (SNOMED-CT) used in the UK primary care since 2018. Finally, proprietary codes are also used in each provider (eg, “EMISNQSU106—Suspected 2019-nCoV [novel coronavirus] infection”). Hospital care data and mortality data are recorded using International Classification of Diseases version 10 (ICD-10) and version 9 (ICD9) terminologies.^16^ ICD for Oncology version 3 (ICD-O^17^) was used for recording cancer registry data. Procedures during hospital admissions are recorded using an UK-specified ontology, OPCS-3 and OPCS-4.^18^

### OMOP CDM and ETL process

**Figure 1.**
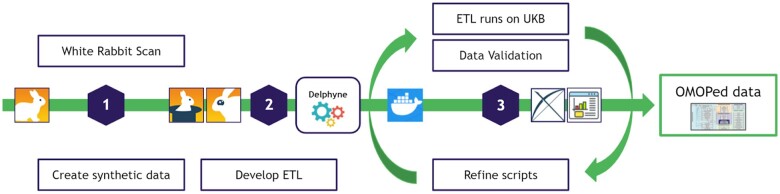
Transformation process (synthetic data development, iterative deployment); In the first phase, data profiling is performed over the source data (UK Biobank) and based on the results, synthetic data for developmental and validation purposes were generated. The second phase involves development of the ETL using the delphyne pipeline. Finally, an iterative validation and redefinition phase is performed. ETL: extract, transform, load; OMOP: Observational Medical Outcomes Partnership; UKB: UK Biobank.

We used OMOP CDM version 5.3 (Figure 1)^19^ which consists of 23 tables organized in 4 top-level domains: clinical, derived elements, health system, and health economics. Clinical data tables (*n* = 15) hold core data on patient demographics, clinical events (eg, diagnoses, laboratory measurements, medication prescriptions, surgical procedures), visit occurrences and observation periods. We preprocessed clinical events such as drug exposure periods and stored information as derived elements (*n* = 3). The health system data tables (*n* = 3) provide information on healthcare providers associated with the healthcare events held in the clinical data types. The UKB contains over 9000 individual data fields from participants and spans multiple data modalities. We excluded -omics, imaging and bespoke binary research data (eg, accelerometer). Supplementary Table S5 provides an overview of mapping methods for all data sources and their respective clinical terminologies. Detailed ETL documentation available at https://ehden.github.io/ETL-UK-Biobank.

#### Baseline data mapping

We used the Observational Health Data Sciences and Informatics (OHDSI) White Rabbit tool^20^ a data profiling tool which scans the source data to provide information on tables, fields and values. We used the tool to generate a data profile on all UKB data tables, fields, and values. The output was used to gain a better understanding of the source data and design the syntactic transformation. We prioritized 519 baseline fields by: (1) engaging with the community through the OMOP UKB Working Group we established, (2) expert review for clinicians to identify key data of high interest or related to COVID-19 research, and (3) triaging the fields by generating descriptive statistics and including the most frequently occurring values or combinations of values.

We classified baseline fields into numeric/continuous (eg, measurements such as systolic blood pressure) and discrete (eg, a patient reported past medical history). Discrete fields were further classified as: (1) Boolean fields where the answer is true or false, (2) categorical fields where the value was a string or more from a pre-set list of values. For each numeric field, 2 mappings to standard concepts were created: firstly a mapping for the event (preferably from SNOMED-CT and the Measurement domain) and, secondly a mapping for the respective unit (from Unified Code for Units of Measure [UCUM]^21^). Dates associated with discrete and numeric fields were included in the mapping (Supplementary Table S1).

**Figure 2.**
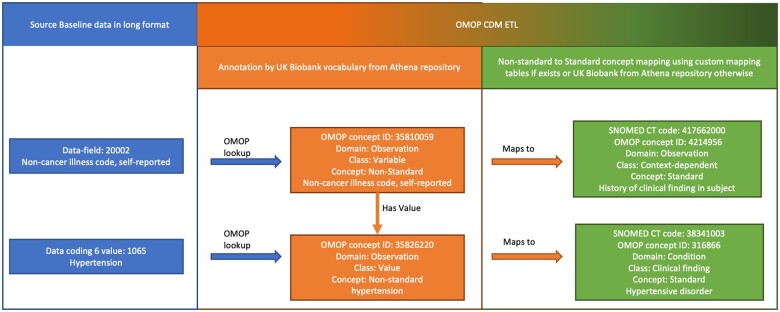
Example of a semantic mapping of self-reported hypertension. Mapping is realized in 2 steps using: (1) UK Biobank vocabulary and (2) custom created nonstandard to standard concept mapping tables. OMOP: Observational Medical Outcomes Partnership; CDM: Common Data Model. Here, the UK Biobank data field 20002 (*noncancer illness code, self-reported*) with value 1065 (*hypertension*) is transformed to an OMOP an observation record with observation_concept_id 4214956 *(History of clinical finding in subject)* and value_as_concept_id 316866 *(Hypertensive disorder)*.

In a data preprocessing phase, we traversed the wide source data format, ie, 1 row per patient with columns corresponding to each data field, into a long format, ie, 1 row per patient and specific data field. Within the syntactic mapping phase, we mapped patient identifiers, data field identifiers, data field values, units, and dates (if applicable) onto corresponding fields of the OMOP CDM tables (eg, sex, ethnicity, date of death) or as a clinical event record (self-reported diseases, blood pressure measurement); (Supplementary Figure S1). We annotated baseline data fields by OMOP Concept ID using the UK Biobank Athena vocabulary providing information about data field hierarchical structure, categories and value encoding systems used in the dataset. Athena^22^ is the OHDSI vocabulary repository that merges multiple medical ontologies and provides unique Concept IDs for terms from each source ontology. This vocabulary however does not implement nonstandard to standard concept mappings, ie, mapping from nonstandard UK Biobank fields to standard SNOMED concepts. Therefore, we created custom mapping tables for the prioritized fields (Figure 2) by using USAGI.^23^ USAGI proposes “Non-standard to Standard map” suggestions between imported nonstandard terms and standard concepts from OMOP CDM supported terminologies. The suggestions are evaluated by a textual match score. Accepted suggestions were manually reviewed and the output exported to mapping files. The UK Biobank fields were added during this project as a “nonstandard” source vocabulary, with some concepts mapping to standard OMOP concepts. In case of an occurrence of multiple mapping candidates, the final target concept ID was selected by choosing the standard concept in the OMOP vocabulary providing the best match according to OHDSI conventions. If ambiguity remained, target concepts were selected based on preferred target terminology (mostly SNOMED) and target domain (eg, condition preferred over observation for a diagnosis of Hypertension). The OMOP CDM also allows to store the nonstandard source concepts, for which we used the UKB vocabulary in Athena. We processed 31 hematology measurements (UKB field id 9081) which were directly measured (eg, white blood cell count), calculated (eg, neutrophil number) or derived (eg, mean platelet volume) from samples obtained from participants during the recruitment center assessment. All semantically unmapped fields (not mapped to a standard concept) were transformed into the OMOP Observation domain with a Concept ID 0 and the original field id as the source value to preserve this information.

#### EHR data mapping

The linked EHR data (eg, participant data from primary and secondary care, cancer registrations and mortality) were mapped to different OMOP tables (measurements, conditions, observations, or procedures) based on the domain of the target OMOP concept. The observation domain was used as default when the target OMOP concept was missing. Records with a special value were excluded from the transformation, eg, for an invalid CTV3/SNOMED-CT code (−3), for a missing code (−99), for the potential sensitivity of diagnosis code on rare occasions (−1) or for rare occupation (−2). In the interest of resource efficiency and speed, in each EHR source we prioritized for manual review and mapping the terms that accounted for at least 80% of the clinical events recorded.

Primary care EHR data uses 2 different terminologies: SNOMED-CT and CTV3. SNOMED-CT codes are natively supported in OMOP and were semantically mapped directly to target concepts by OMOP CDM mapping tables. CTV3 codes were mapped using the official CTV3 to SNOMED-CT cross maps provided by NHS Digital.^24^ The map contains 1:1 (*n* = 178 266) and 1: many mappings (*n* = 3189). One: many mappings were filtered by the following rules: (1) only mappings labeled as “Preferred” and “Active” were used; (2) only mappings where the target concept is standard were used, (3) Target domain classification respects the following priority list: Measurement, Condition, Observation, Procedure. Remaining unmapped terms (*n* = 2095) were ordered by frequency and the 80% most used (*n* = 100) were manually reviewed. During the review, more specific mappings were prioritized. Finally, proprietary EMIS and TPP codes were manually reviewed and mapped using USAGI. Primary care prescriptions were encoded by dm+d for EMIS and TPP. Records were mapped to the OMOP Drug Exposure table, using the RxNorm terminology applying existing dm+d-RxNorm^25^ mappings available in Athena.

Hospital EHR data were transformed in a similar way, with the difference that the source used ICD-10 and ICD-9 for diagnoses and OPCS-3 and OPCS-4 for procedures. These codes were mapped with the available mapping to SNOMED-CT in the OMOP vocabulary. Hospital procedures were encoded in OPCS-3 and OPCS-4 (OPCS Classification of Interventions and Procedures version 3 and 4) vocabularies. For OPCS-4 codes we used a mapping existing in the OMOP vocabulary. OPCS-3 had to be mapped manually using USAGI. We prioritized and mapped the *n* = 328 terms (out of a total of 1900 terms) that accounted for 80% of events.

#### SARS-CoV-2 infection and COVID-19 status ascertainment

Data from national COVID-19 testing laboratories made available for research^26^ were mapped onto a common concept *Measurement of Severe acute respiratory syndrome coronavirus 2 (SARS-CoV-2) (OMOP Extension vocabulary)* with specification of the specimen type used for the COVID-19 test and test result as positive or negative. For the downstream analysis, we ascertained COVID-19 status by combining information from national serology testing data, admitted hospital episodes, primary care diagnoses (including proprietary EMIS and TPP codes), and cause of death information on using a previously validated phenotyping algorithm^2^^,^^27^ (Supplementary Table S2a and S2b).

#### Evaluation and validation

During the development phase, the ETL was tested using synthetic data automatically generated by the Python library Tofu^29^ and manually written test cases. This enabled the developers to test the ETL on a large synthetic dataset. Validation of the final transformation was performed using OHDSI tools: Achilles^30^ and the DataQualityDashboard (DQD)^31^ and the EHDEN CDMInspection tool.^32^ Achilles performs ∼300 analyses on the transformed data. DataQualityDasboard runs ∼3.5k checks testing data quality on the conformance, completeness, and plausibility of the data in the OMOP CDM. CDMInspection provides 14 additional checks on top of the DataQualityDashboard, mainly focused on vocabulary and technical infrastructure of the CDM. Multiple iterations of conversion and validation were performed until the validation checks passed. We utilized Achilles to create a dashboard of visualizations of key data source characteristics (eg, demographics and most occurring clinical events) and inspected them for consistency and clinical plausibility after each iteration in collaboration with clinical colleagues.

We validated the mapping by defining and comparing a series of metrics between the raw data, the OMOP converted data and the subset of OMOP converted data which had tested positive for COVID-19. Specifically, we extracted information on: (1) key demographic fields (from the baseline assessment center visit), (2) lifestyle risk factors (eg, smoking status), (3) clinical biomarkers (eg, blood pressure) and, (4) clinical comorbidities. Clinical comorbidities were defined using a set of previously validated phenotyping algorithms from CALIBER^28^^,^^33^: Type 2 Diabetes (T2DM), Heart Failure (HF), Acute Myocardial Infarction (AMI), Chronic Obstructive Pulmonary Disease (COPD) and Hypertension (HT). We used Atlas,^34^ a unified interface for OHDSI tools, and OMOP compatible SQL queries to generate and compare the metrics between the datasets. For comorbidities, the number (and percentage) of patients identified in each dataset was compared while for continuous measurements the median and standard deviation were compared.

#### Statistical analyses

We generated and reported descriptive statistics (mean, median) for key demographic and clinical variables in the cohort, stratified by COVID-19 status. For each source, we created descriptive analyses to report the most frequent unmapped concepts per OMOP domain during the ETL process.

#### Open source mapping and vocabulary files

To perform the transformation, we used delphyne,^35^ a Python OMOP ETL pipeline developed by The Hyve. The source code and ETL mapping files for this project are available: https://ehden.github.io/ETL-UK-Biobank. We used a bespoke OMOP vocabulary for UKB baseline fields/categories available in Athena,^36^ which we extended for this project. The CTV3, and CTV3-SNOMED-CT mappings are available from NHS Digital.

##### Data availability and ethical approval

Ethical approval for this study was provided from the UKB Access Review Board, reference 58356 “Defining and redefining human disease at scale: an atlas of the human phenome.” Participant data for this project are available directly from the UKB following a protocol review and contractual agreements, more information can be found on the UKB website. We excluded participants that had withdrawn consent from the study.

## RESULTS

### Data sources and SARS-CoV-2 ascertainment

We identified 502 505 unique participants in the UKB and transformed 1 373 239 555 rows of data across all sources to the OMOP CDM (Table 1). A single participant was rejected (with all associated data) from the ETL pipeline due to a missing year of birth value. We identified 3093 (0.61% of total) participants with COVID-19 during the study period We successfully identified 3086 of these participants (99.8%) in the OMOP CDM. Seven participants were not identified as a small number of relevant clinical records were not mapped due to a missing nonstandard to standard concept mapping (eg, CTV3 code *X73lE—Coronavirus*). We transformed 690 distinct fields with 2898 values encoded by proprietary coding systems (Supplementary Table S3 presents a list of all fields).

**Table 1.** Patient demographic and clinical characteristics presented for the source population, the OMOP CDM transformed population and the subset of the transformed population with COVID-19

	Source UK Biobank data	OMOP-transformed UK Biobank data	Transformed UK Biobank COVID-19 positive sub population
Patients	502 505	502 504	3086
% Female	54.4	54.4	48.76
Median age (IQR)	58 (13)	58 (13)	58 (15)
Median Townsend deprivation index (IQR)	−2.135 (4.18)	−2.135 (4.18)	−1.111 (5.19)
BMI median—baseline (IQR)	26.652 (5.72)	26.65 (5.70)	27.7 (6.21)
BMI median—GP EMIS (IQR)	27.2 (6.9)	27.3 (6.84)	28.89 (8)
SBP median—baseline (IQR)	136 (26)	136 (26)	136 (25)
DBP median—baseline (IQR)	81 (14)	81 (14)	82 (14)
Smoking status			
Not answered	2276	Not mapped	Not mapped
Never	317 891	317 891	1676
Previous	197 949	197 949	1323
Current	55 676	55 676	395
Comorbidities			
T2DM	40 433 (8.04%)	40 476 (8.05%)	453 (14.67%)
HF	8068 (1.6%)	8053 (1.6%)	140 (4.53%)
AMI	10 593 (2.1%)	10 749 (2.13%)	110 (3.56%)
COPD	22 364 (4.45%)	22 367 (4.45%)	328 (10.62%)
HT	175 449 (34.91%)	175 539 (34.93%)	1571 (50.9%)

*Note*: Age, Townsend deprivation index, Body Mass Index (BMI), Systolic Blood Pressure (SBP) and Diastolic Blood Pressure (DBP) values collected at first assessment center visit.

T2DM: type-II diabetes; HF: heart failure; AMI: acute myocardial infarction; COPD: chronic obstructive pulmonary disease; HT: hypertension.

### Baseline and EHR data mapping

In the baseline data, we processed events from 1 127 434 self-reported noncancer illnesses (field id 20002), 53 384 cancer illnesses (field id 20001), 1 381 148 medications (field id 20003) and, 994 355 procedures entries (field id 20004) and mapped 946 053 (83.91%), 37 802 (70.81%), 1 218 935 (88.25%) and 864 788 (86.96%) entries respectively (Table 2) in addition to 45 629 849 (74.65%) hematology entries.

**Table 2.** Mapping coverage for terms in the baseline and EHR data relating to ethnic status, noncancer/cancer diseases, medication usage and surgical procedures in the UK Biobank and converted to the OMOP CDM standard vocabulary

Source vocab	Used source terms #	Mapped used terms # (%)	Events #	Mapped event # (%)
Baseline ethnic status	22	10 (45.45%)	533 612	512 158 (95.97%)
Self-reported noncancer illness	446	351 (78.69%)	1 127 434	946 053 (83.91%)
Self-reported cancer	82	48 (58.53%)	53 384	37 802 (70.81%)
Self-reported medication	3737	1100 (29.43%)	1 381 148	1 218 935 (88.25%)
Self-reported procedures	254	128 (50.39%)	994 355	864 788 (86.96%)
Hematology samples	124	93 (75%)	61 119 731	45 629 849 (74.65%)
Hospital EHR admission source	86	44 (51.16%)	3 541 594	282 505 (7.97%)
Hospital EHR admission method	63	58 (92.06%)	3 541 610	3 540 046 (99.95%)
Hospital EHR discharge destination	91	56 (61.53%)	3 484 435	3 189 509 (91.53%)

*Note*: Coverage is given as both the number of unique terms mapped and as the number of events mapped.

EHR: electronic health records.

In hospitalization EHR (Table 3), we processed 12 962 292 diagnoses using ICD-10 and mapped 12 961 962 (99.99%). Additionally, we processed 7 220 399 procedure events using the OPCS-4 classification and successfully mapped 6 449 843 (89.32%). A significantly smaller number of clinical events using deprecated terminologies (eg, ICD-9 and OPCS-3) were mapped to a high degree of accuracy (Table 3). Finally, 77 127 (99.95%) of all death events recorded in mortality registers across the 3 countries were successfully mapped (cause of death recorded using ICD-10).

**Table 3.** Mapping and event coverage for UK Biobank vocabularies for diagnoses, procedures, and death electronic health records

Source vocab	Used source terms #	Mapped used terms # (%)	Events #	Mapped event # (%)
ICD-10 diagnoses	12 094	12 088 (99.95%)	12 962 292	12 961 962 (99.99%)
ICD-9 diagnoses	3337	2847 (85.31%)	72 256	66 220 (91.64%)
OPCS-3 procedures	883	221 (25.02%)	20 077	15 556 (77.48%)
OPCS-4 procedures	8324	8276 (99.42%)	7 220 399	6 449 843 (89.32%)
ICD-10 Death Cause	1962	1961 (99.94%)	77 161	77 127 (99.95%)

*Note*: Coverage is given as both the number of unique terms mapped and as the number of events mapped.

ICD: International Classification of Diseases; OPCS: OPCS Classification of Interventions and Procedures.

In primary care EHR (Table 4), we processed 212 828 306 clinical events from EMIS and 133 092 016 clinical events from TPP. These were recorded using 51 160 SNOMED-CT and 82 669 Clinical Terms Version 3 (CTV3) terms respectively. In EMIS data, 49 968 (97.67%) of SNOMED-CT concepts were mapped successfully resulting in 207 756 102 (97.62%) of clinical events mapped. In TPP, 73 683 (89.13%) of CTV3 concepts were mapped but the proportion of successfully mapped clinical events remained equally high with 97.78% of events (*n* = 130 140 231) successfully mapped. Measurement units in EMIS for relevant clinical events (eg, mmHg for blood pressure) were recorded using 55 terms of which 44 were mapped resulting in 31.27% of events successfully transformed. We processed 141 752 534 medication prescription events which were recorded using dm+d. Overall, 30 859 (99.85%) were mapped and 139 966 587 (98.74%) were successfully transformed. Finally, we mapped 41 COVID-19-related unique proprietary codes used by primary care EHR software vendors.

**Table 4.** Mapping and event coverage for UK Biobank primary care electronic health records

Source vocab	Used source terms #	Mapped used terms # (%)	Events #	Mapped event # (%)
EMIS units	4544	44 (0.96%)	94 623 584	82 517 900 (87.2%)
SNOMED-CT (EMIS)	51 160	49 968 (97.67%)	212 828 306	207 756 102 (97.62%)
dm+d	30 903	30 859 (99.85%)	141 752 534	139 966 587 (98.74%)
CTV3 (TPP)	82 669	73 683 (89.13%)	133 092 016	130 140 231 (97.78%)
TPP and EMIS proprietary codes	20 990	41 (0.19%)	19 554 574	37 882 (0.19%)

Lists of top 10 most frequently used mapped and unmapped terms can be found in Supplementary Table S4a–S4k.

### Evaluation and validation

We identified 40 433 T2DM, 8068 HF, 10 593 AMI, 22 364 COPD and 175 449 HT patients in the source data and observed similar estimates in the converted data. A small number of patients (43 AMI, 15 HF, 157 AMI, 6 COPD and 94 HT) were identified only in the converted data and not in the source data.

DataQualityDashboard verified and validated plausibility, conformance, and completeness of the transformed dataset. On the final run, 3399 checks passed and 18 failed (Supplementary Figure S2). All remaining failed checks were investigated, and their failure was expected. Seven checks on completeness failed because the percentage of records with a value of 0 in the standard concept field exceeded a threshold (20%) due to missing mappings. Two plausibility checks failed due to an incompatible gender for a gender related clinical code, eg, 41 records with a concept 198197—male infertility are not associated with participants identified as males. This is given by the source data. Due to errors introduced during the manual mapping process (ie, incorrect mapping selections using USAGI), 9 conformance checks failed as a standard Concept ID value in a table did not conform with a corresponding domain (eg, 0.2% of unit Concept ID values in a Measurement table do not conform with a Unit domain).

## DISCUSSION

We have extracted and transformed the UKB, a complex large-scale biobank cohort study of 502 504 middle-aged individuals from England, Scotland, and Wales. The study combined self-reported data from questionnaires which were collected during recruitment and longitudinal EHR from primary care consultations, hospital admissions, cancer registrations, and mortality using 8 different clinical terminologies. Overall, >1.3 billion rows of data were processed and transformed to the OMOP CDM. Transformation of OMOP has enabled UKB to take part in federated analyses of 17 health data sources on adverse events of special interest (AESIs) associated with COVID-19 vaccination and many other studies are ongoing.^37^^,^^39^

Representing data collected through questionnaires in the CDM was a challenging task and required a significant amount of preprocessing and consolidation across multiple fields. Eight custom mapping tables together with vocabularies from the existing OMOP vocabularies were used to map data fields and data values to standard OMOP concepts. Each type of data required a different mapping approach. One challenge was that OMOP measurements do not have many attributes, eg, for the Hemoglobin concentration (field id 30020), the freeze-thaw cycles data field (field id 30021) and the device ID (field id 30023) had to be mapped as a separate observation and device record respectively.

In line with previous studies^38^ that used similar controlled clinical terminologies for EHR, our approach achieved high mapping coverage (>97% coverage) across established systems, eg, SNOMED-CT, ICD-10. Similarly, 89% of surgical procedure events recorded in OPCS-4 were transformed. Older terminologies, eg, ICD-9, OPCS-3, used in historic data had slightly less good coverage: 91% and 77%, respectively. In contrast with previous research using prescription information in primary care EHR, the establishment of dm+d as the standard used has led to a significantly improved mapping accuracy of 98.7%. Using USAGI, we mapped a small subset of the proprietary TPP and EMIS codes related to COVID-19 (41, 0.19%). The mapping of these proprietary codes had a significant impact on COVID-19 case ascertainment as it captured ∼60% of unique identified cases in primary care data and 28% in all sources.

We observed good overall concordance when comparing key demographic, risk factor and clinical comorbidities source and converted data. Broadly, we observed 2 classes of problems. Firstly, not all patients identified by comorbidity in the source data were identified in the transformed data. One cause is semantically unmapped diagnosis codes used for a cohort identification and appearing in patients’ clinical records (eg, CTV3 code X73lE—Coronavirus, used for identification COVID-19 cases; *n* = 15). A second cause are restrictions imposed by the ETL (eg, diagnosis codes outside observation period window).

Secondly, a very small number of patients were only identified as cases in the transformed data (*n* = 3 in case of COVID-19 cases). This occurs when 2 or more distinct source codes are mapped onto the same target code. If the source comorbidity definition uses one code and not the other, it is not possible to separate these using the target code (Supplementary Figure S3). Mapping of 2 or more source codes onto the same target concept could be a result of: (1) an incorrectly specified mapping, (2) specific source codes are mapped onto a more general target code or (3) synonymous source codes In the latter case the source comorbidity definition should take both source codes into account.

Our study does have limitations. Not all available data could be mapped to the OMOP CDM and must be handled separately. For example, genomic data (eg, SNPs) cannot be integrated within the OMOP CDM as the data model has been developed for routinely collected healthcare and claims data. This provides an additional layer of complexity when creating studies that need to combine information across phenotypic and genomic sources. Information collected via questionnaires is also challenging to include as it differs from typical OMOP CDM data; it uses local coding systems, storing data in a wide format and of cross-sectional nature. In addition, questionnaire data often captures negation and data missingness explicitly (eg, patient did not answer or refused to answer), which by convention is not stored in the OMOP CDM. As with previous studies, the OMOP CDM definition of an observation period (the period for which the data capture of a person is considered complete) causes some discrepancies between analysis on the source and OMOP CDM as historical medical events are considered outside the observation period. It should be noted that this has been recently revised, and events outside observation period are allowed in the OMOP CDM for some use cases.

Finally, our study findings are potentially generalizable to other large datasets consisting of research-driven questionnaires and EHR linkage that require conversion to the OMOP CDM. The UK Biobank contains detailed phenotypic data that are sourced from different data modalities (eg, patient-reported questionnaires data, research data, claims data and EHR) combined with deep genotypic information. This resulted in a challenging technical implementation including the usage of a custom OMOP vocabulary. Other similar resources in terms of complexity, such as All of Us^40^ and the MVP^41^ in the United States can potentially benefit from our findings when undergoing similar conversion to OMOP CDM for participation in OHDSI studies.

## CONCLUSION

Our study demonstrated that the OMOP CDM can be successfully leveraged to harmonize complex large-scale biobanked studies. Our study did uncover several challenges when transforming data collected using bespoke questionnaires from patients to the OMOP CDM which require further research. The transformed UK Biobank resource is a valuable research tool that can enable large-scale research in COVID-19 and other diseases.

## FUNDING

This study was supported by a European Health Data & Evidence Network (EHDEN) project grant; This project has received funding from the Innovative Medicines Initiative 2 Joint Undertaking (JU) under grant agreement No 806968. The JU receives support from the European Union’s Horizon 2020 research and innovation program and EFPIA. The grant was for the institute. SD, RJBD, and VP are funded by the UCLH NIHR Biomedical Research Centre (BRC). SD is supported by BHF Data Science Centre led by Health Data Research UK (BHF Grant no. SP/19/3/34678); the COVID-19 Longitudinal Health and Wellbeing National Core Study funded by the Medical Research Council (MC_PC_20030; MC_PC_20059) and Health Data Research UK, which receives its core funding from the UK Medical Research Council, Engineering and Physical Sciences Research Council, Economic and Social Research Council, Department of Health and Social Care (England), Chief Scientist Office of the Scottish Government Health and Social Care Directorates, Health and Social Care Research and Development Division (Welsh Government), Public Health Agency (Northern Ireland), British Heart Foundation (BHF) and the Wellcome Trust. RJBD is supported by the following: (1) NIHR Biomedical Research Centre at South London and Maudsley NHS Foundation Trust and King’s College London, London, UK; (2) Health Data Research UK, which is funded by the UK Medical Research Council, Engineering and Physical Sciences Research Council, Economic and Social Research Council, Department of Health and Social Care (England), Chief Scientist Office of the Scottish Government Health and Social Care Directorates, Health and Social Care Research and Development Division (Welsh Government), Public Health Agency (Northern Ireland), British Heart Foundation and Wellcome Trust; (3) The BigData@Heart Consortium, funded by the Innovative Medicines Initiative-2 Joint Undertaking under grant agreement No. 116074. This Joint Undertaking receives support from the European Union’s Horizon 2020 research and innovation program and EFPIA; it is chaired by DE Grobbee and SD Anker, partnering with 20 academic and industry partners and ESC; (4) the National Institute for Health Research University College London Hospitals Biomedical Research Centre; (5) the National Institute for Health Research (NIHR) Biomedical Research Centre at South London and Maudsley NHS Foundation Trust and King’s College London; (6) the UK Research and Innovation London Medical Imaging & Artificial Intelligence Centre for Value Based Healthcare; (7) the National Institute for Health Research (NIHR) Applied Research Collaboration South London (NIHR ARC South London) at King’s College Hospital NHS Foundation Trust. FA is supported by UCL Hospitals NIHR Biomedical Research Centre.

## AUTHOR CONTRIBUTIONS

SD conceived and designed the study. MM, EAV, SB, AVW, AP, SP implemented the ETL pipeline. SD, MM, VP reviewed and revised ETL mapping files. VP executed the ETL pipeline, extracted data and conducted the analyses. VP and SD analyzed and interpreted the results. SD, VP and MM wrote the report. All authors reviewed and interpreted the results, commented on the report, contributed to revisions, and read and approved the final version.

## SUPPLEMENTARY MATERIAL

Supplementary material is available at *Journal of the American Medical Informatics Association* online.

## CONFLICT OF INTEREST STATEMENT

EAV is an employee of Janssen Research and Development LLC and a shareholder of Johnson & Johnson (J&J) stock. Prof. Prieto-Alhambra’s research group has received grant support from Amgen, Chesi-Taylor, Novartis, and UCB Biopharma. His department has received advisory or consultancy fees from Amgen, Astellas, AstraZeneca, Johnson, and Johnson, and UCB Biopharma and fees for speaker services from Amgen and UCB Biopharma. Janssen, on behalf of IMI-funded EHDEN and EMIF consortiums, and Synapse Management Partners have supported training programs organized by DP-A’s department and open for external participants organized by his department outside this work.

## Supplementary Material

ocac203_Supplementary_Data
