# Correction to: Transforming and evaluating the UK Biobank to the OMOP Common Data Model for COVID-19 research and beyond

**DOI:** 10.1093/jamia/ocad032

**Published:** 2023-02-27

This is a correction to: Vaclav Papez, Maxim Moinat, Erica A Voss, Sofia Bazakou, Anne Van Winzum, Alessia Peviani, Stefan Payralbe, Elena Garcia Lara, Michael Kallfelz, Folkert W Asselbergs, Daniel Prieto-Alhambra, Richard J B Dobson, Spiros Denaxas, Transforming and evaluating the UK Biobank to the OMOP Common Data Model for COVID-19 research and beyond, *Journal of the American Medical Informatics Association*, Volume 30, Issue 1, January 2023, Pages 103–111, https://doi.org/10.1093/jamia/ocac203

In the originally published version of this manuscript, Elena Garcia Lara was erroneously omitted from the list of authors.

This error has now been corrected.

