# Supplementary material for: Transforming and evaluating the UK Biobank to the OMOP Common Data Model for COVID-19 research and beyond
Source: J Am Med Inform Assoc. 2022 Oct 13;30(1):103–11. doi: 10.1093/jamia/ocac203 (PMC9619789; doi:10.1093/jamia/ocac203)
Supplement: ocac203_Supplementary_Data [file ocac203_supplementary_data.zip › ocac203_Supplementary_Data/Supplementary Table 1.docx]

**Supplementary Table 1**: An example of baseline fields classification into numeric/continuous, discrete and date fields. For each numeric field a mapping for the event and a mapping for the respective unit is realized. Dates are associated with both discrete and numeric fields.

| **Type** | **Example** | **Approach** | **Result** |
| --- | --- | --- | --- |
| Numeric/Continuous | 30000 - White blood cell (leukocyte) count | Concept mapping  Unit mapping | 4298431 - White blood cell count (SNOMED)  1863 - Count |
| Date | 30002 - White blood cell (leukocyte) count acquisition time | Date mapping | Value in date attribute for specific concept (e.g., Measurement date) |
| Discrete - boolean | 2443 - Has your doctor ever diagnosed you with diabetes? | Concept mapping | If answer yes:  Diabetes diagnosis |
| Discrete - categorical | 1558 - Alcohol intake frequency  1 Daily or almost daily  2 Three or four times a week  3 Once or twice a week  4 One to three times a month  5 Special occasions only  6 Never  -3 Prefer not to answer | Concept mapping  Value mapping | 4052351 - Alcohol intake  45879676 - Daily or almost daily  … |
