# Supplementary material for: Transforming and evaluating the UK Biobank to the OMOP Common Data Model for COVID-19 research and beyond
Source: J Am Med Inform Assoc. 2022 Oct 13;30(1):103–11. doi: 10.1093/jamia/ocac203 (PMC9619789; doi:10.1093/jamia/ocac203)
Supplement: ocac203_Supplementary_Data [file ocac203_supplementary_data.zip › ocac203_Supplementary_Data/Supplementary Table 2.docx]

**Supplementary Table 2a**: Identified Covid19 cases in source data - breakdown by data source. In total, we identified 3093 unique Covid19 cases across all data sources. Some cases are overlapping between the data sources.

| **Data source** | **Frequency** |
| --- | --- |
| Primary care data | 1,945 |
| - TPP Primary care data using proprietary codes | 1,014 |
| - EMIS Primary care data using SNOMED CT codes | 740 |
| - EMIS Primary care data using proprietary codes | 190 |
| - TPP Primary care data using CTV3 codes | 61 |
| National laboratory testing data | 1,550 |
| Hospital admission EHR data | 1,057 |
| Mortality EHR data | 457 |

**Supplementary Table 2b:** Identified COVID-19 cases (distinct patients) in the source data - breakdown by diagnosis code from primary and hospital care data.

| **Terminology** | **Source code** | **Label** | **N** |
| --- | --- | --- | --- |
| ICD10 | U071 | COVID-19, virus identified | 1011 |
| TPP Proprietary code | Y20cf | Suspected coronavirus disease 19 caused by severe acute respiratory syndrome coronavirus | 802 |
| ICD10 | U072 | COVID-19, virus not identified | 274 |
| SNOMED CT | 1300721000000109 | Coronavirus disease 19 caused by severe acute respiratory syndrome coronavirus 2 confirmed by laboratory test (situation) | 269 |
| SNOMED CT | 1240751000000100 | Coronavirus disease 19 caused by severe acute respiratory syndrome coronavirus 2 (disorder) | 253 |
| EMIS Proprietary code | EMISNQEM35 | MIS COVID-19 care pathway template entry | 138 |
| SNOMED CT | 1240581000000104 | Severe acute respiratory syndrome coronavirus 2 ribonucleic acid detected (finding) | 124 |
| TPP Proprietary code | Y20fa | Coronavirus disease 19 caused by severe acute respiratory syndrome coronavirus 2 (disorder) | 107 |
| SNOMED CT | 186747009 | Coronavirus infection (disorder) | 96 |
| TPP Proprietary code | Y20d1 | Severe acute respiratory syndrome coronavirus 2 detected (finding) | 62 |
| TPP Proprietary code | Y228d | Coronavirus disease 19 caused by severe acute respiratory syndrome coronavirus 2 confirmed by laboratory test (situation) | 56 |
| EMIS Proprietary code | EMISNQCO303 | Confirmed 2019-nCoV (novel coronavirus) infection | 52 |
| SNOMED CT | 1300631000000101 | Coronavirus disease 19 severity score (observable entity) | 40 |
| TPP Proprietary code | Y22a2 | Coronavirus disease 19 caused by severe acute respiratory syndrome coronavirus 2 excluded by laboratory test (situation) | 35 |
| CTV3 | A795. | Coronavirus infection | 31 |
| SNOMED CT | 1300731000000106 | Coronavirus disease 19 caused by severe acute respiratory syndrome coronavirus 2 confirmed using clinical diagnostic criteria (situation) | 28 |
| SNOMED CT | 1008541000000105 | Coronavirus ribonucleic acid detection assay (observable entity) | 26 |
| TPP Proprietary code | Y210a | Pneumonia caused by severe acute respiratory syndrome coronavirus 2 (disorder) | 21 |
| SNOMED CT | 1300681000000102 | Assessment using coronavirus disease 19 severity scale (procedure) | 20 |
| TPP Proprietary code | Y228e | Coronavirus disease 19 caused by severe acute respiratory syndrome coronavirus 2 confirmed using clinical diagnostic criteria (situation) | 19 |
| SNOMED CT | 1240551000000105 | Pneumonia caused by severe acute respiratory syndrome coronavirus 2 (disorder) | 15 |
| CTV3 | X73lE | Coronavirus | 15 |
| SNOMED CT | 1240511000000106 | Detection of severe acute respiratory syndrome coronavirus 2 using polymerase chain reaction technique (procedure) | 14 |
| TPP Proprietary code | Y210e | Detection of severe acute respiratory syndrome coronavirus 2 using polymerase chain reaction technique (procedure) | 12 |
| CTV3 | X73lF | Human coronavirus | 11 |
| TPP Proprietary code | Y210b | Infection of upper respiratory tract caused by severe acute respiratory syndrome coronavirus 2 (disorder) | <10 |
| SNOMED CT | 1321541000000108 | Severe acute respiratory syndrome coronavirus 2 immunoglobulin G detected (finding) | <10 |
| SNOMED CT | 1240741000000103 | Severe acute respiratory syndrome coronavirus 2 serology (observable entity) | <10 |
| CTV3 | AyuDC | [X]Coronavirus infection, unspecified | <10 |
| SNOMED CT | 1321551000000106 | Severe acute respiratory syndrome coronavirus 2 immunoglobulin M detected (finding) | <10 |
| TPP Proprietary code | Y23f7 | SARS-CoV-2 (severe acute respiratory syndrome coronavirus 2) detection result positive | <10 |
| SNOMED CT | 1300671000000104 | Coronavirus disease 19 severity scale (assessment scale) | <10 |
| SNOMED CT | 1029481000000103 | Coronavirus nucleic acid detection assay (observable entity) | <10 |
| CTV3 | A7y00 | Coronavirus as cause of dis classified to other chapters | <10 |
