# Supplementary material for: Transforming and evaluating the UK Biobank to the OMOP Common Data Model for COVID-19 research and beyond
Source: J Am Med Inform Assoc. 2022 Oct 13;30(1):103–11. doi: 10.1093/jamia/ocac203 (PMC9619789; doi:10.1093/jamia/ocac203)
Supplement: ocac203_Supplementary_Data [file ocac203_supplementary_data.zip › ocac203_Supplementary_Data/Supplementary Table 4.docx]

## Lists of top mapped and top unmapped terms

Supplementary Tables 4a-4k shows lists of top 10 unmapped (Tables 4a-4e) and top 10 mapped (Tables 4f+4k) source terms grouped by the target domain of OMOP CDM. All tables were generated by the CDMInspection validation tool.

**Supplementary Table 4a:** Top 10 Unmapped Drugs

| Ranking | Source code | Label | Vocabulary | Records # | Patients # |
| --- | --- | --- | --- | --- | --- |
| 1 | GALI1279 | Gaviscon Liquid | EMIS Local code | 36,000 | 7,300 |
| 2 | -2 |  |  | 32,500 | 13,400 |
| 3 | CECR13101NEMIS | Cetraben Emollient Cream | EMIS Local code | 19,800 | 5,200 |
| 4 | SHCO24024NEMIS | Sharpsafe Container 1 litre | EMIS Local code | 18,200 | 3,100 |
| 5 | VIEY51060NEMIS | Vita-Pos Eye ointment (preservative-free) | EMIS Local code | 16,500 | 2,400 |
| 6 | EPCR95969NEMIS | Epimax Cream | EMIS Local code | 10,500 | 3,100 |
| 7 | GATA1280 | Gaviscon Tablets | EMIS Local code | 9,100 | 1,600 |
| 8 | EFCA19645EMIS | Efamast 40 Capsules 40 mg | EMIS Local code | 9,000 | 1,300 |
| 9 | ADTE5735NEMIS | Advantage Ii Test strips |  | 8,800 | 900 |
| 10 | TRTA2933 | Triludan Tablets 60 mg | EMIS Local code | 8,700 | 2,600 |

**Supplementary Table 4b:** Top 10 Unmapped Conditions

| Ranking | Source code | Label | Vocabulary | Records # | Patients # |
| --- | --- | --- | --- | --- | --- |
| 1 | U511 | Previously admitted to a psychiatric hospital of the provider | ICD10 | 200 | 200 |
| 2 | U510 | Not Previously admitted to a psyciatric hospital | ICD10 | 200 | 200 |
| 3 | V519 | Aftercare involving the use of plastic surgery | ICD9 | 100 | 100 |
| 4 | C42.3 | hematopoietic and reticuloendothelial systems: Reticuloendothelial system, NOS (ICD-O-3 specific) | ICD-O-3 | 100 | 100 |
| 5 | E91599 | F.b. acc. entering other orifice (at: unspecified place) | ICD9 | 100 | 100 |
| 6 | U52 | First in a series of a regular day/night admissions | ICD10 | 100 | 100 |
| 7 | U512 | Previously admitted to a psychiatric hospital of another provider | ICD10 | 100 | 100 |
| 8 | C42.4 | hematopoietic and reticuloendothelial systems: Hematopoietic system, NOS (ICD-O-3 specific) | ICD-O-3 | 100 | 100 |
| 9 | E91199 | Inhal/ingest. food caus. obst. resp. tract/suffocation (at: unspec place) | ICD9 | 100 | 100 |
| 10 | C42.1 | hematopoietic and reticuloendothelial systems: bone marrow (ICD-O-3 specific) | ICD-O-3 | 100 | 100 |

**Supplementary Table 4c**: Top 10 Unmapped Measurements

| Ranking | Source code | Label | Vocabulary | Records # | Patients # |
| --- | --- | --- | --- | --- | --- |
| 1 | Ub173 | Alcohol units per week | CTV3 | 71,400 | 39,000 |
| 2 | EMISFR4 | 10 yr CHD risk (Estimate,Fram.) | EMIS Local code | 69,200 | 27,400 |
| 3 | EMISNQAU129 | AUDIT-C score - frequency of drinking alcohol | EMIS Local code | 39,700 | 28,100 |
| 4 | Y0a8a | Joint British Societies cardiovascular 10yr risk | TPP Local code | 37,200 | 25,400 |
| 5 | EMISNQFR8 | Framingham cardiovascular disease estimated 10 year risk score | EMIS Local code | 37,000 | 16,800 |
| 6 | Y0a8b | Joint British Societies adjusted cardiovascular 10yr risk | TPP Local code | 36,100 | 24,300 |
| 7 | Y18d9 | Ideal body weight - Devine formula | TPP Local code | 31,000 | 18,100 |
| 8 | Y18db | Ideal body weight - Lemmens formula | TPP Local code | 25,400 | 15,500 |
| 9 | EMISNQAU130 | AUDIT-C score - units of alcohol drunk on a typical day | EMIS Local code | 22,200 | 16,500 |
| 10 | EMISNQFR5 | Framingham cardiovascular disease 10 year risk score | EMIS Local code | 16,800 | 8,100 |

**Supplementary Table 4d:** Top 10 Unmapped Observations

| Ranking | Source code | Label | Vocabulary | Records # | Patients # |
| --- | --- | --- | --- | --- | --- |
| 1 | EMISATTACHMENT | Attachment | EMIS Local code | 9,215,500 | 228,400 |
| 2 | 5986 | ECG, phase time | UK Biobank field ID | 5,758,700 | 95,200 |
| 3 | 5987 | ECG, trend phase name | UK Biobank field ID | 5,758,700 | 95,200 |
| 4 | 5988 | ECG, stage name | UK Biobank field ID | 5,758,700 | 95,200 |
| 5 | 5985 | Bicycle speed | UK Biobank field ID | 5,745,500 | 95,000 |
| 6 | 5984 | ECG, load | UK Biobank field ID | 5,745,500 | 95,000 |
| 7 | 4249 | Number of times 'clear' was pressed (right) | UK Biobank field ID | 3,509,800 | 207,600 |
| 8 | 4240 | Triplet played (right) | UK Biobank field ID | 3,509,800 | 207,600 |
| 9 | 4241 | Signal-to-noise-ratio (SNR) of triplet (right) | UK Biobank field ID | 3,509,800 | 207,600 |
| 10 | 4229 | Triplet played (left) | UK Biobank field ID | 3,508,800 | 207,500 |

**Supplementary Table 4e:** Top 10 Unmapped Procedures

| Ranking | Source code | Label | Vocabulary | Records # | Patients # |
| --- | --- | --- | --- | --- | --- |
| 1 | 912 | Excision of superficial cyst or fistula | OPCS3 | 400 | 400 |
| 2 | 708 | Intra-uterine introduction | OPCS3 | 300 | 300 |
| 3 | 9611 | Other therapy for cancer : chemotherapy for cancer | OPCS3 | 200 | 100 |
| 4 | 7061 | Biopsy of cervix : cone or ring biopsy | OPCS3 | 200 | 200 |
| 5 | Y795 | Transluminal approach to organ through radial artery | OPCS4 | 200 | 100 |
| 6 | 771 | Other removal of retained products | OPCS3 | 100 | 100 |
| 7 | 691 | Extended hysterectomy | OPCS3 | 100 | 100 |
| 8 | 2221 | Treatment of lesion of nasal mucosa : of turbinal bones | OPCS3 | 100 | 100 |
| 9 | 306 | Catheter manipulations, not elsewhere classified | OPCS3 | 100 | 100 |
| 10 | 785 | Removal of internal fixation | OPCS3 | 100 | 100 |

**Supplementary Table 4f:** Top 10 Mapped Drugs

| Ranking | Concept term | Records # | Patients # |
| --- | --- | --- | --- |
| 1 | simvastatin 40 MG Oral Tablet | 3,411,000 | 85,500 |
| 2 | omeprazole 20 MG Delayed Release Oral Capsule | 3,286,600 | 176,300 |
| 3 | bendroflumethiazide 2.5 MG Oral Tablet | 2,960,700 | 57,000 |
| 4 | aspirin 75 MG Disintegrating Oral Tablet | 2,663,700 | 65,300 |
| 5 | amlodipine 5 MG Oral Tablet | 2,347,600 | 87,000 |
| 6 | levothyroxine sodium 0.1 MG Oral Tablet | 1,945,700 | 24,400 |
| 7 | atorvastatin 20 MG Oral Tablet | 1,920,500 | 81,300 |
| 8 | acetaminophen 500 MG Oral Tablet | 1,861,700 | 105,300 |
| 9 | lansoprazole 30 MG Delayed Release Oral Capsule | 1,806,600 | 113,300 |
| 10 | simvastatin 20 MG Oral Tablet | 1,722,400 | 52,100 |

**Supplementary Table 4g:** Top 10 Mapped Conditions

| Ranking | Concept term | Records # | Patients # |
| --- | --- | --- | --- |
| 1 | O/E - blood pressure reading | 5,581,900 | 308,000 |
| 2 | Essential hypertension | 1,731,900 | 175,800 |
| 3 | Cervical smear - negative | 1,235,800 | 200,700 |
| 4 | O/E - pulse rhythm regular | 1,033,300 | 277,400 |
| 5 | Bowel cancer screening programme faecal occult blood test normal | 771,400 | 273,100 |
| 6 | Urine glucose test negative | 751,100 | 228,700 |
| 7 | Urine protein test negative | 736,700 | 224,100 |
| 8 | History of clinical finding in subject | 552,800 | 132,300 |
| 9 | Mammography normal | 549,600 | 161,500 |
| 10 | Asthma | 528,100 | 63,300 |

**Supplementary Table 4h:** Top 10 Mapped Measurements

| Ranking | Concept term | Records # | Patients # |
| --- | --- | --- | --- |
| 1 | Heart rate | 5,804,900 | 142,000 |
| 2 | Systolic arterial pressure | 5,470,100 | 241,700 |
| 3 | Diastolic arterial pressure | 5,454,400 | 241,700 |
| 4 | Platelet count | 5,099,400 | 496,900 |
| 5 | Neutrophil count | 5,001,100 | 496,700 |
| 6 | Lymphocyte count | 4,984,300 | 496,700 |
| 7 | Monocyte count | 4,968,200 | 496,700 |
| 8 | Eosinophil count | 4,968,000 | 496,700 |
| 9 | Basophil count | 4,853,000 | 496,600 |
| 10 | Body mass index | 4,742,200 | 501,800 |

**Supplementary Table 4i:** Top 10 Mapped Observations

| Ranking | Concept term | Records # | Patients # |
| --- | --- | --- | --- |
| 1 | Laboratory reporting | 13,184,400 | 491,400 |
| 2 | Correction applied | 13,168,000 | 491,400 |
| 3 | Corrected | 11,296,100 | 491,400 |
| 4 | SMS (short message service) text message sent to patient | 6,825,900 | 267,600 |
| 5 | No history of clinical finding in subject | 3,256,500 | 501,300 |
| 6 | Reason for no laboratory result | 3,183,100 | 489,200 |
| 7 | History of clinical finding in subject | 2,640,800 | 465,000 |
| 8 | Never smoked tobacco | 2,621,000 | 368,500 |
| 9 | No evidence of | 2,485,700 | 411,900 |
| 10 | Blood sample taken | 2,124,400 | 277,000 |

**Supplementary Table 4j**: Top 10 Mapped Procedures

| Ranking | Concept term | Records # | Patients # |
| --- | --- | --- | --- |
| 1 | Specimen aliquoting | 14,196,700 | 475,000 |
| 2 | Review of medication | 2,401,900 | 273,200 |
| 3 | Seasonal influenza vaccination | 1,196,400 | 224,900 |
| 4 | Medication review with patient | 1,132,900 | 226,000 |
| 5 | Smoking cessation education | 965,200 | 197,300 |
| 6 | Diet education | 898,800 | 240,100 |
| 7 | Administration of influenza vaccine | 629,100 | 127,600 |
| 8 | Patient review | 591,300 | 122,200 |
| 9 | Lifestyle education | 546,000 | 194,000 |
| 10 | Education about alcohol consumption | 459,600 | 157,400 |

**Supplementary Table 4k:** Top 10 Mapped Devices

| Ranking | Concept term | Records # | Patients # |
| --- | --- | --- | --- |
| 1 | Biomedical device | 20,790,900 | 499,000 |
| 2 | Olive oil | 5,300 | 2,900 |
| 3 | Ear fitting hearing aid | 2,300 | 2,000 |
| 4 | Hearing aid | 2,100 | 1,800 |
| 5 | Gastroscope | 1,600 | 1,400 |
| 6 | Total hip replacement prosthesis | 1,600 | 1,400 |
| 7 | Contraceptive diaphragm | 1,300 | 800 |
| 8 | Indwelling urethral catheter | 1,300 | 800 |
| 9 | Shin splint | 500 | 500 |
| 10 | Support stockings - garment | 500 | 400 |
