# Supplementary material for: Transforming and evaluating the UK Biobank to the OMOP Common Data Model for COVID-19 research and beyond
Source: J Am Med Inform Assoc. 2022 Oct 13;30(1):103–11. doi: 10.1093/jamia/ocac203 (PMC9619789; doi:10.1093/jamia/ocac203)
Supplement: ocac203_Supplementary_Data [file ocac203_supplementary_data.zip › ocac203_Supplementary_Data/Supplementary Table 5.docx]

**Supplementary Table 5**: An overview of mapping methods used for each data source and type of data.  We distinguish two types of mappings: Bespoke (created a custom code mapping for this project), Reused (using existing external code mapping resources). UCUM = Unified Code for Units of Measure ; CTV3 = Clinical Terms Version 3; dm+d = dictionary of medicines and devices; EMIS= Egton Medical Information Systems; TPP= The Phoenix Partnership; OMOP = Observational Medical Outcomes Partnership ; CDM = Common Data Model; OPCS = OPCS Classification of Interventions and Procedures; ICD= International Classification of Diseases; International Classification of Diseases – Oncology; EHR = Electronic Health Records.

| **Data source** | **Data type** | **Source terminology** | **Target terminology** | **Type of mapping** |
| --- | --- | --- | --- | --- |
| Baseline | Continuous | UK Biobank proprietary | SNOMED CT | Bespoke |
| Baseline | Discrete | UK Biobank proprietary | SNOMED CT | Bespoke |
| Baseline | Units | UK Biobank proprietary | UCUM | Bespoke |
| Baseline | Dates |  | OMOP CDM table attributes |  |
| Baseline | Demographics | UK Biobank proprietary | OMOP CDM table attributes |  |
| COVID-19 tests | Test results | UK Biobank proprietary | SNOMED CT | Bespoke |
| Primary Care EHR | Diagnoses, laboratory results, symptoms | SNOMED CT | SNOMED CT | Reused via Athena |
| Primary Care EHR | Diagnoses, laboratory results, symptoms | CTV3 | SNOMED CT | Bespoke |
| Primary Care EHR | Diagnoses, laboratory results, symptoms | EMIS/TPP proprietary codes | SNOMED CT | Bespoke |
| Primary Care EHR | Prescriptions | dm+d | RxNorm | Reused |
| Hospital Care EHR | Diagnoses | ICD-9 | SNOMED CT | Reused via Athena |
| Hospital Care EHR | Diagnoses | ICD-10 | SNOMED CT | Reused via Athena |
| Hospital Care EHR | Surgical procedures and interventions | OPCS-3 | SNOMED CT | Reused via Athena |
| Hospital Care EHR | Surgical procedures and interventions | OPCS-4 | SNOMED CT | Reused via Athena |
| Cancer registry | Cancer diagnoses | ICDO-3 | SNOMED CT | Bespoke |
| Death registry | Primary and secondary causes of death | ICD-9 | SNOMED CT | Reused via Athena |
| Death registry | Primary and secondary causes of death | ICD-10 | SNOMED CT | Reused via Athena |
