# Supplementary material for: Transforming and evaluating the UK Biobank to the OMOP Common Data Model for COVID-19 research and beyond
Source: J Am Med Inform Assoc. 2022 Oct 13;30(1):103–11. doi: 10.1093/jamia/ocac203 (PMC9619789; doi:10.1093/jamia/ocac203)
Supplement: ocac203_Supplementary_Data [file ocac203_supplementary_data.zip › ocac203_Supplementary_Data/Supplementary_Table_3.docx]

| Supplementary Table 3 | | | | | |
| --- | --- | --- | --- | --- | --- |
| **Category** | **Category Name** | **Field** | **Field name** | **Prioritized** | **Mapped** |
| **152** | Process durations | 3 | Verbal interview duration | No | No |
| **152** | Process durations | 4 | Biometrics duration | No | No |
| **152** | Process durations | 5 | Sample collection duration | No | No |
| **152** | Process durations | 6 | Conclusion duration | No | No |
| **100018** | Bone-densitometry of heel | 19 | Heel ultrasound method | No | No |
| **100010** | Body size measures | 21 | Weight method | No | No |
| **100020** | Spirometry | 23 | Spirometry method | No | No |
| **100094** | Baseline characteristics | 31 | Sex | No | Yes |
| **100094** | Baseline characteristics | 33 | Date of birth | No | No |
| **100094** | Baseline characteristics | 34 | Year of birth | No | Yes |
| **100002** | Blood sample collection | 35 | Was blood sampling attempted | No | No |
| **100011** | Blood pressure | 36 | Blood pressure device ID | Yes | Yes |
| **100011** | Blood pressure | 37 | Blood pressure manual sphygmomanometer device ID | Yes | Yes |
| **100019** | Hand grip strength | 38 | Hand grip dynamometer device ID | Yes | Yes |
| **100010** | Body size measures | 39 | Height measure device ID | Yes | Yes |
| **100010** | Body size measures | 40 | Manual scales device ID | Yes | Yes |
| **100010** | Body size measures | 41 | Seating box device ID | Yes | Yes |
| **100020** | Spirometry | 42 | Spirometer device ID | Yes | Yes |
| **100009** | Impedance measures | 43 | Impedance device ID | Yes | Yes |
| **100010** | Body size measures | 44 | Tape measure device ID | Yes | Yes |
| **100018** | Bone-densitometry of heel | 45 | Heel ultrasound device id | No | No |
| **100019** | Hand grip strength | 46 | Hand grip strength (left) | Yes | Yes |
| **100019** | Hand grip strength | 47 | Hand grip strength (right) | Yes | Yes |
| **100010** | Body size measures | 48 | Waist circumference | Yes | Yes |
| **100010** | Body size measures | 49 | Hip circumference | Yes | Yes |
| **100010** | Body size measures | 50 | Standing height | Yes | Yes |
| **100010** | Body size measures | 51 | Seated height | Yes | Yes |
| **100094** | Baseline characteristics | 52 | Month of birth | No | Yes |
| **100024** | Reception | 53 | Date of attending assessment centre | No | Yes |
| **100024** | Reception | 54 | UK Biobank assessment centre | No | Yes |
| **100024** | Reception | 55 | Month of attending assessment centre | No | No |
| **100004** | Procedural metrics | 62 | Willing to attempt cognitive tests | No | No |
| **100002** | Blood sample collection | 68 | Number of blood samples taken | No | No |
| **100002** | Blood sample collection | 74 | Fasting time | No | No |
| **100018** | Bone-densitometry of heel | 77 | Heel bone ultrasound T-score, manual entry | No | Yes |
| **100018** | Bone-densitometry of heel | 78 | Heel bone mineral density (BMD) T-score, automated | No | Yes |
| **100074** | Medical conditions | 84 | Cancer year/age first occurred | No | No |
| **100074** | Medical conditions | 87 | Non-cancer illness year/age first occurred | No | No |
| **100076** | Operations | 92 | Operation year/age first occurred | No | No |
| **100011** | Blood pressure | 93 | Systolic blood pressure, manual reading | No | Yes |
| **100011** | Blood pressure | 94 | Diastolic blood pressure, manual reading | No | Yes |
| **100011** | Blood pressure | 95 | Pulse rate (during blood-pressure measurement) | Yes | Yes |
| **100011** | Blood pressure | 96 | Time since interview start at which blood pressure screen(s) shown | No | No |
| **100011** | Blood pressure | 102 | Pulse rate, automated reading | No | Yes |
| **100004** | Procedural metrics | 111 | Aide-memoire completed | No | No |
| **100072** | Early life factors | 115 | Father's month of birth | No | No |
| **100072** | Early life factors | 117 | Mother's month of birth | No | No |
| **100072** | Early life factors | 118 | Mother's day of birth | No | No |
| **100072** | Early life factors | 120 | Birth weight known | No | No |
| **100072** | Early life factors | 129 | Place of birth in UK - north co-ordinate | No | No |
| **100072** | Early life factors | 130 | Place of birth in UK - east co-ordinate | No | No |
| **100073** | Employment | 132 | Job code at visit - entered | No | No |
| **100074** | Medical conditions | 134 | Number of self-reported cancers | No | No |
| **100074** | Medical conditions | 135 | Number of self-reported non-cancer illnesses | No | No |
| **100076** | Operations | 136 | Number of operations, self-reported | No | No |
| **100075** | Medications | 137 | Number of treatments/medications taken | No | No |
| **100072** | Early life factors | 146 | Father's day of birth | No | No |
| **100094** | Baseline characteristics | 189 | Townsend deprivation index at recruitment | No | No |
| **2** | Ongoing characteristics | 190 | Reason lost to follow-up | Yes | Yes |
| **2** | Ongoing characteristics | 191 | Date lost to follow-up | Yes | Yes |
| **100023** | Consent | 200 | Date of consenting to join UK Biobank | No | No |
| **100023** | Consent | 393 | Program (tactus) version ID (compiler timestamp) | No | No |
| **100030** | Pairs matching | 396 | Number of columns displayed in round | No | No |
| **100030** | Pairs matching | 397 | Number of rows displayed in round | No | No |
| **100030** | Pairs matching | 398 | Number of correct matches in round | No | No |
| **100030** | Pairs matching | 399 | Number of incorrect matches in round | No | No |
| **100030** | Pairs matching | 400 | Time to complete round | No | No |
| **100032** | Reaction time | 401 | Index for card A in round | No | No |
| **100032** | Reaction time | 402 | Index for card B in round | No | No |
| **100032** | Reaction time | 403 | Number of times snap-button pressed | No | No |
| **100032** | Reaction time | 404 | Duration to first press of snap-button in each round | No | No |
| **152** | Process durations | 630 | Touchscreen duration | No | No |
| **100066** | Household | 670 | Type of accommodation lived in | No | No |
| **100066** | Household | 680 | Own or rent accommodation lived in | No | No |
| **100066** | Household | 699 | Length of time at current address | No | No |
| **100066** | Household | 709 | Number in household | No | No |
| **100066** | Household | 728 | Number of vehicles in household | No | No |
| **100066** | Household | 738 | Average total household income before tax | No | No |
| **100064** | Employment | 757 | Time employed in main current job | No | No |
| **100064** | Employment | 767 | Length of working week for main job | No | No |
| **100064** | Employment | 777 | Frequency of travelling from home to job workplace | No | No |
| **100064** | Employment | 796 | Distance between home and job workplace | No | No |
| **100064** | Employment | 806 | Job involves mainly walking or standing | No | No |
| **100064** | Employment | 816 | Job involves heavy manual or physical work | No | No |
| **100064** | Employment | 826 | Job involves shift work | No | No |
| **100063** | Education | 845 | Age completed full time education | No | No |
| **100054** | Physical activity | 864 | Number of days/week walked 10+ minutes | No | No |
| **100054** | Physical activity | 874 | Duration of walks | No | No |
| **100054** | Physical activity | 884 | Number of days/week of moderate physical activity 10+ minutes | No | No |
| **100054** | Physical activity | 894 | Duration of moderate activity | No | No |
| **100054** | Physical activity | 904 | Number of days/week of vigorous physical activity 10+ minutes | No | No |
| **100054** | Physical activity | 914 | Duration of vigorous activity | No | No |
| **100054** | Physical activity | 924 | Usual walking pace | No | No |
| **100054** | Physical activity | 943 | Frequency of stair climbing in last 4 weeks | No | No |
| **100054** | Physical activity | 971 | Frequency of walking for pleasure in last 4 weeks | No | No |
| **100054** | Physical activity | 981 | Duration walking for pleasure | No | No |
| **100054** | Physical activity | 991 | Frequency of strenuous sports in last 4 weeks | No | No |
| **100054** | Physical activity | 1001 | Duration of strenuous sports | No | No |
| **100054** | Physical activity | 1011 | Frequency of light DIY in last 4 weeks | No | No |
| **100054** | Physical activity | 1021 | Duration of light DIY | No | No |
| **100061** | Social support | 1031 | Frequency of friend/family visits | No | No |
| **100055** | Sun exposure | 1050 | Time spend outdoors in summer | No | No |
| **100055** | Sun exposure | 1060 | Time spent outdoors in winter | No | No |
| **100054** | Physical activity | 1070 | Time spent watching television (TV) | No | No |
| **100054** | Physical activity | 1080 | Time spent using computer | No | No |
| **100054** | Physical activity | 1090 | Time spent driving | No | No |
| **100054** | Physical activity | 1100 | Drive faster than motorway speed limit | No | No |
| **100053** | Electronic device use | 1110 | Length of mobile phone use | No | No |
| **100053** | Electronic device use | 1120 | Weekly usage of mobile phone in last 3 months | No | No |
| **100053** | Electronic device use | 1130 | Hands-free device/speakerphone use with mobile phone in last 3 month | No | No |
| **100053** | Electronic device use | 1140 | Difference in mobile phone use compared to two years previously | No | No |
| **100053** | Electronic device use | 1150 | Usual side of head for mobile phone use | No | No |
| **100057** | Sleep | 1160 | Sleep duration | No | No |
| **100057** | Sleep | 1170 | Getting up in morning | No | No |
| **100057** | Sleep | 1180 | Morning/evening person (chronotype) | No | No |
| **100057** | Sleep | 1190 | Nap during day | No | No |
| **100057** | Sleep | 1200 | Sleeplessness / insomnia | No | No |
| **100057** | Sleep | 1210 | Snoring | No | No |
| **100057** | Sleep | 1220 | Daytime dozing / sleeping (narcolepsy) | No | No |
| **100058** | Smoking | 1239 | Current tobacco smoking | No | No |
| **100058** | Smoking | 1249 | Past tobacco smoking | No | No |
| **100058** | Smoking | 1259 | Smoking/smokers in household | No | No |
| **100058** | Smoking | 1269 | Exposure to tobacco smoke at home | No | No |
| **100058** | Smoking | 1279 | Exposure to tobacco smoke outside home | No | No |
| **100052** | Diet | 1289 | Cooked vegetable intake | No | No |
| **100052** | Diet | 1299 | Salad / raw vegetable intake | No | No |
| **100052** | Diet | 1309 | Fresh fruit intake | No | No |
| **100052** | Diet | 1319 | Dried fruit intake | No | No |
| **100052** | Diet | 1329 | Oily fish intake | No | No |
| **100052** | Diet | 1339 | Non-oily fish intake | No | No |
| **100052** | Diet | 1349 | Processed meat intake | No | No |
| **100052** | Diet | 1359 | Poultry intake | No | No |
| **100052** | Diet | 1369 | Beef intake | No | No |
| **100052** | Diet | 1379 | Lamb/mutton intake | No | No |
| **100052** | Diet | 1389 | Pork intake | No | No |
| **100052** | Diet | 1408 | Cheese intake | No | No |
| **100052** | Diet | 1418 | Milk type used | No | No |
| **100052** | Diet | 1428 | Spread type | No | No |
| **100052** | Diet | 1438 | Bread intake | No | No |
| **100052** | Diet | 1448 | Bread type | No | No |
| **100052** | Diet | 1458 | Cereal intake | No | No |
| **100052** | Diet | 1468 | Cereal type | No | No |
| **100052** | Diet | 1478 | Salt added to food | No | No |
| **100052** | Diet | 1488 | Tea intake | No | No |
| **100052** | Diet | 1498 | Coffee intake | No | No |
| **100052** | Diet | 1508 | Coffee type | No | No |
| **100052** | Diet | 1518 | Hot drink temperature | No | No |
| **100052** | Diet | 1528 | Water intake | No | No |
| **100052** | Diet | 1538 | Major dietary changes in the last 5 years | No | No |
| **100052** | Diet | 1548 | Variation in diet | No | No |
| **100051** | Alcohol | 1558 | Alcohol intake frequency. | Yes | Yes |
| **100051** | Alcohol | 1568 | Average weekly red wine intake | No | No |
| **100051** | Alcohol | 1578 | Average weekly champagne plus white wine intake | No | No |
| **100051** | Alcohol | 1588 | Average weekly beer plus cider intake | No | No |
| **100051** | Alcohol | 1598 | Average weekly spirits intake | No | No |
| **100051** | Alcohol | 1608 | Average weekly fortified wine intake | No | No |
| **100051** | Alcohol | 1618 | Alcohol usually taken with meals | No | No |
| **100051** | Alcohol | 1628 | Alcohol intake versus 10 years previously | No | No |
| **100033** | Early life factors | 1647 | Country of birth (UK/elsewhere) | No | No |
| **100033** | Early life factors | 1677 | Breastfed as a baby | No | No |
| **100033** | Early life factors | 1687 | Comparative body size at age 10 | No | No |
| **100033** | Early life factors | 1697 | Comparative height size at age 10 | No | No |
| **100033** | Early life factors | 1707 | Handedness (chirality/laterality) | No | No |
| **100055** | Sun exposure | 1717 | Skin colour | No | No |
| **100055** | Sun exposure | 1727 | Ease of skin tanning | No | No |
| **100055** | Sun exposure | 1737 | Childhood sunburn occasions | No | No |
| **100055** | Sun exposure | 1747 | Hair colour (natural, before greying) | No | No |
| **100055** | Sun exposure | 1757 | Facial ageing | No | No |
| **100033** | Early life factors | 1767 | Adopted as a child | No | No |
| **100033** | Early life factors | 1777 | Part of a multiple birth | No | No |
| **100033** | Early life factors | 1787 | Maternal smoking around birth | No | No |
| **100034** | Family history | 1797 | Father still alive | No | No |
| **100034** | Family history | 1807 | Father's age at death | No | No |
| **100034** | Family history | 1835 | Mother still alive | No | No |
| **100034** | Family history | 1845 | Mother's age | No | No |
| **100034** | Family history | 1873 | Number of full brothers | No | No |
| **100034** | Family history | 1883 | Number of full sisters | No | No |
| **100060** | Mental health | 1920 | Mood swings | No | No |
| **100060** | Mental health | 1930 | Miserableness | No | No |
| **100060** | Mental health | 1940 | Irritability | No | No |
| **100060** | Mental health | 1950 | Sensitivity / hurt feelings | No | No |
| **100060** | Mental health | 1960 | Fed-up feelings | No | No |
| **100060** | Mental health | 1970 | Nervous feelings | No | No |
| **100060** | Mental health | 1980 | Worrier / anxious feelings | No | No |
| **100060** | Mental health | 1990 | Tense / 'highly strung' | No | No |
| **100060** | Mental health | 2000 | Worry too long after embarrassment | No | No |
| **100060** | Mental health | 2010 | Suffer from 'nerves' | No | No |
| **100060** | Mental health | 2020 | Loneliness, isolation | No | No |
| **100060** | Mental health | 2030 | Guilty feelings | No | No |
| **100060** | Mental health | 2040 | Risk taking | No | No |
| **100060** | Mental health | 2050 | Frequency of depressed mood in last 2 weeks | No | No |
| **100060** | Mental health | 2060 | Frequency of unenthusiasm / disinterest in last 2 weeks | No | No |
| **100060** | Mental health | 2070 | Frequency of tenseness / restlessness in last 2 weeks | No | No |
| **100060** | Mental health | 2080 | Frequency of tiredness / lethargy in last 2 weeks | No | No |
| **100060** | Mental health | 2090 | Seen doctor (GP) for nerves, anxiety, tension or depression | No | No |
| **100060** | Mental health | 2100 | Seen a psychiatrist for nerves, anxiety, tension or depression | No | No |
| **100061** | Social support | 2110 | Able to confide | No | No |
| **100056** | Sexual factors | 2129 | Answered sexual history questions | No | No |
| **100056** | Sexual factors | 2139 | Age first had sexual intercourse | No | No |
| **100056** | Sexual factors | 2149 | Lifetime number of sexual partners | No | No |
| **100056** | Sexual factors | 2159 | Ever had same-sex intercourse | No | No |
| **100042** | General health | 2178 | Overall health rating | No | No |
| **100042** | General health | 2188 | Long-standing illness, disability or infirmity | No | No |
| **100041** | Eyesight | 2207 | Wears glasses or contact lenses | No | No |
| **100041** | Eyesight | 2217 | Age started wearing glasses or contact lenses | No | No |
| **100041** | Eyesight | 2227 | Other eye problems | No | No |
| **100053** | Electronic device use | 2237 | Plays computer games | No | No |
| **100043** | Hearing | 2247 | Hearing difficulty/problems | No | No |
| **100043** | Hearing | 2257 | Hearing difficulty/problems with background noise | No | No |
| **100055** | Sun exposure | 2267 | Use of sun/uv protection | No | No |
| **100055** | Sun exposure | 2277 | Frequency of solarium/sunlamp use | No | No |
| **100042** | General health | 2296 | Falls in the last year | No | No |
| **100042** | General health | 2306 | Weight change compared with 1 year ago | No | No |
| **100037** | Breathing | 2316 | Wheeze or whistling in the chest in last year | No | No |
| **100039** | Chest pain | 2335 | Chest pain or discomfort | Yes | Yes |
| **100040** | Cancer screening | 2345 | Ever had bowel cancer screening | Yes | Yes |
| **100040** | Cancer screening | 2355 | Most recent bowel cancer screening | No | No |
| **100040** | Cancer screening | 2365 | Ever had prostate specific antigen (PSA) test | Yes | Yes |
| **100070** | Male-specific factors | 2375 | Relative age of first facial hair | No | No |
| **100070** | Male-specific factors | 2385 | Relative age voice broke | No | No |
| **100070** | Male-specific factors | 2395 | Hair/balding pattern | No | No |
| **100070** | Male-specific factors | 2405 | Number of children fathered | No | No |
| **100047** | Operations | 2415 | Had major operations | No | No |
| **100044** | Medical conditions | 2443 | Diabetes diagnosed by doctor | Yes | Yes |
| **100044** | Medical conditions | 2453 | Cancer diagnosed by doctor | Yes | Yes |
| **100044** | Medical conditions | 2463 | Fractured/broken bones in last 5 years | Yes | Yes |
| **100044** | Medical conditions | 2473 | Other serious medical condition/disability diagnosed by doctor | Yes | Yes |
| **100045** | Medication | 2492 | Taking other prescription medications | Yes | Yes |
| **100054** | Physical activity | 2624 | Frequency of heavy DIY in last 4 weeks | No | No |
| **100054** | Physical activity | 2634 | Duration of heavy DIY | No | No |
| **100058** | Smoking | 2644 | Light smokers, at least 100 smokes in lifetime | No | No |
| **100052** | Diet | 2654 | Non-butter spread type details | No | No |
| **100051** | Alcohol | 2664 | Reason for reducing amount of alcohol drunk | No | No |
| **100069** | Female-specific factors | 2674 | Ever had breast cancer screening / mammogram | No | No |
| **100069** | Female-specific factors | 2684 | Years since last breast cancer screening / mammogram | No | No |
| **100069** | Female-specific factors | 2694 | Ever had cervical smear test | No | No |
| **100069** | Female-specific factors | 2704 | Years since last cervical smear test | No | No |
| **100069** | Female-specific factors | 2714 | Age when periods started (menarche) | No | No |
| **100069** | Female-specific factors | 2724 | Had menopause | Yes | Yes |
| **100069** | Female-specific factors | 2734 | Number of live births | Yes | Yes |
| **100069** | Female-specific factors | 2744 | Birth weight of first child | Yes | Yes |
| **100069** | Female-specific factors | 2754 | Age at first live birth | Yes | Yes |
| **100069** | Female-specific factors | 2764 | Age at last live birth | Yes | Yes |
| **100069** | Female-specific factors | 2774 | Ever had stillbirth, spontaneous miscarriage or termination | No | Yes |
| **100069** | Female-specific factors | 2784 | Ever taken oral contraceptive pill | Yes | Yes |
| **100069** | Female-specific factors | 2794 | Age started oral contraceptive pill | Yes | Yes |
| **100069** | Female-specific factors | 2804 | Age when last used oral contraceptive pill | Yes | Yes |
| **100069** | Female-specific factors | 2814 | Ever used hormone-replacement therapy (HRT) | Yes | Yes |
| **100069** | Female-specific factors | 2824 | Age at hysterectomy | Yes | Yes |
| **100069** | Female-specific factors | 2834 | Bilateral oophorectomy (both ovaries removed) | Yes | Yes |
| **100047** | Operations | 2844 | Had other major operations | Yes | Yes |
| **100058** | Smoking | 2867 | Age started smoking in former smokers | No | No |
| **100058** | Smoking | 2877 | Type of tobacco previously smoked | No | No |
| **100058** | Smoking | 2887 | Number of cigarettes previously smoked daily | No | No |
| **100058** | Smoking | 2897 | Age stopped smoking | No | No |
| **100058** | Smoking | 2907 | Ever stopped smoking for 6+ months | No | No |
| **100058** | Smoking | 2926 | Number of unsuccessful stop-smoking attempts | No | No |
| **100058** | Smoking | 2936 | Likelihood of resuming smoking | No | No |
| **100034** | Family history | 2946 | Father's age | No | No |
| **100048** | Pain | 2956 | General pain for 3+ months | No | No |
| **100044** | Medical conditions | 2966 | Age high blood pressure diagnosed | Yes | Yes |
| **100044** | Medical conditions | 2976 | Age diabetes diagnosed | Yes | Yes |
| **100044** | Medical conditions | 2986 | Started insulin within one year diagnosis of diabetes | Yes | Yes |
| **100044** | Medical conditions | 3005 | Fracture resulting from simple fall | Yes | Yes |
| **100020** | Spirometry | 3059 | Result ranking | No | No |
| **100020** | Spirometry | 3060 | Time of blow measurement | No | No |
| **100020** | Spirometry | 3061 | Acceptability of each blow result | No | No |
| **100020** | Spirometry | 3062 | Forced vital capacity (FVC) | Yes | Yes |
| **100020** | Spirometry | 3063 | Forced expiratory volume in 1-second (FEV1) | Yes | Yes |
| **100020** | Spirometry | 3064 | Peak expiratory flow (PEF) | Yes | Yes |
| **100020** | Spirometry | 3065 | Ordering of blows | No | No |
| **100020** | Spirometry | 3066 | Data points for blow | No | No |
| **100010** | Body size measures | 3077 | Seating box height | No | No |
| **100076** | Operations | 3079 | Pace-maker | No | No |
| **100018** | Bone-densitometry of heel | 3081 | Foot measured for bone density | No | No |
| **100018** | Bone-densitometry of heel | 3082 | Fractured heel | No | No |
| **100018** | Bone-densitometry of heel | 3083 | Heel quantitative ultrasound index (QUI), manual entry | No | No |
| **100018** | Bone-densitometry of heel | 3084 | Heel bone mineral density (BMD), manual entry | No | No |
| **100018** | Bone-densitometry of heel | 3085 | Heel Broadband ultrasound attenuation (BUA), manual entry | No | No |
| **100018** | Bone-densitometry of heel | 3086 | Speed of sound through heel, manual entry | No | No |
| **100020** | Spirometry | 3088 | Contra-indications for spirometry | No | No |
| **100020** | Spirometry | 3089 | Caffeine drink within last hour | No | No |
| **100020** | Spirometry | 3090 | Used an inhaler for chest within last hour | No | No |
| **100020** | Spirometry | 3132 | Spirometry device serial number | No | No |
| **100020** | Spirometry | 3137 | Number of measurements made | No | No |
| **100074** | Medical conditions | 3140 | Pregnant | No | No |
| **100018** | Bone-densitometry of heel | 3143 | Ankle spacing width | No | No |
| **100018** | Bone-densitometry of heel | 3144 | Heel Broadband ultrasound attenuation, direct entry | No | No |
| **100018** | Bone-densitometry of heel | 3146 | Speed of sound through heel | No | No |
| **100018** | Bone-densitometry of heel | 3147 | Heel quantitative ultrasound index (QUI), direct entry | No | No |
| **100018** | Bone-densitometry of heel | 3148 | Heel bone mineral density (BMD) | No | No |
| **100020** | Spirometry | 3159 | Smoked cigarette or pipe within last hour | No | No |
| **100010** | Body size measures | 3160 | Weight, manual entry | No | Yes |
| **100002** | Blood sample collection | 3166 | Time blood sample collected | No | No |
| **100043** | Hearing | 3393 | Hearing aid user | No | No |
| **100048** | Pain | 3404 | Neck/shoulder pain for 3+ months | No | No |
| **100048** | Pain | 3414 | Hip pain for 3+ months | No | No |
| **100064** | Employment | 3426 | Job involves night shift work | No | No |
| **100058** | Smoking | 3436 | Age started smoking in current smokers | No | No |
| **100058** | Smoking | 3446 | Type of tobacco currently smoked | No | No |
| **100058** | Smoking | 3456 | Number of cigarettes currently smoked daily (current cigarette smokers) | No | No |
| **100058** | Smoking | 3466 | Time from waking to first cigarette | No | No |
| **100058** | Smoking | 3476 | Difficulty not smoking for 1 day | No | No |
| **100058** | Smoking | 3486 | Ever tried to stop smoking | No | No |
| **100058** | Smoking | 3496 | Wants to stop smoking | No | No |
| **100058** | Smoking | 3506 | Smoking compared to 10 years previous | No | No |
| **100034** | Family history | 3526 | Mother's age at death | No | No |
| **100069** | Female-specific factors | 3536 | Age started hormone-replacement therapy (HRT) | Yes | Yes |
| **100069** | Female-specific factors | 3546 | Age last used hormone-replacement therapy (HRT) | Yes | Yes |
| **100048** | Pain | 3571 | Back pain for 3+ months | No | No |
| **100069** | Female-specific factors | 3581 | Age at menopause (last menstrual period) | No | No |
| **100069** | Female-specific factors | 3591 | Ever had hysterectomy (womb removed) | No | No |
| **100039** | Chest pain | 3606 | Chest pain or discomfort walking normally | No | No |
| **100039** | Chest pain | 3616 | Chest pain due to walking ceases when standing still | No | No |
| **100044** | Medical conditions | 3627 | Age angina diagnosed | Yes | Yes |
| **100054** | Physical activity | 3637 | Frequency of other exercises in last 4 weeks | No | No |
| **100054** | Physical activity | 3647 | Duration of other exercises | No | No |
| **100065** | Ethnicity | 3659 | Year immigrated to UK (United Kingdom) | No | No |
| **100056** | Sexual factors | 3669 | Lifetime number of same-sex sexual partners | No | No |
| **100052** | Diet | 3680 | Age when last ate meat | No | No |
| **100069** | Female-specific factors | 3700 | Time since last menstrual period | No | No |
| **100069** | Female-specific factors | 3710 | Length of menstrual cycle | No | No |
| **100069** | Female-specific factors | 3720 | Menstruating today | No | No |
| **100051** | Alcohol | 3731 | Former alcohol drinker | No | No |
| **100048** | Pain | 3741 | Stomach/abdominal pain for 3+ months | No | No |
| **100039** | Chest pain | 3751 | Chest pain or discomfort when walking uphill or hurrying | No | No |
| **100044** | Medical conditions | 3761 | Age hay fever, rhinitis or eczema diagnosed | No | No |
| **100048** | Pain | 3773 | Knee pain for 3+ months | No | No |
| **100044** | Medical conditions | 3786 | Age asthma diagnosed | Yes | Yes |
| **100048** | Pain | 3799 | Headaches for 3+ months | No | No |
| **100040** | Cancer screening | 3809 | Time since last prostate specific antigen (PSA) test | No | No |
| **100069** | Female-specific factors | 3829 | Number of stillbirths | No | No |
| **100069** | Female-specific factors | 3839 | Number of spontaneous miscarriages | No | No |
| **100069** | Female-specific factors | 3849 | Number of pregnancy terminations | No | No |
| **100051** | Alcohol | 3859 | Reason former drinker stopped drinking alcohol | No | No |
| **100069** | Female-specific factors | 3872 | Age of primiparous women at birth of child | No | No |
| **100069** | Female-specific factors | 3882 | Age at bilateral oophorectomy (both ovaries removed) | No | No |
| **100044** | Medical conditions | 3894 | Age heart attack diagnosed | Yes | Yes |
| **100034** | Family history | 3912 | Adopted father still alive | No | No |
| **100034** | Family history | 3942 | Adopted mother still alive | No | No |
| **100034** | Family history | 3972 | Number of adopted brothers | No | No |
| **100034** | Family history | 3982 | Number of adopted sisters | No | No |
| **100044** | Medical conditions | 3992 | Age emphysema/chronic bronchitis diagnosed | Yes | Yes |
| **100044** | Medical conditions | 4012 | Age deep-vein thrombosis (DVT, blood clot in leg) diagnosed | No | Yes |
| **100044** | Medical conditions | 4022 | Age pulmonary embolism (blood clot in lung) diagnosed | Yes | Yes |
| **100044** | Medical conditions | 4041 | Gestational diabetes only | Yes | Yes |
| **100044** | Medical conditions | 4056 | Age stroke diagnosed | Yes | Yes |
| **100048** | Pain | 4067 | Facial pains for 3+ months | No | No |
| **100011** | Blood pressure | 4079 | Diastolic blood pressure, automated reading | No | Yes |
| **100011** | Blood pressure | 4080 | Systolic blood pressure, automated reading | No | Yes |
| **100011** | Blood pressure | 4081 | Method of measuring blood pressure | No | No |
| **100018** | Bone-densitometry of heel | 4092 | Heel ultrasound method (left) | No | No |
| **100018** | Bone-densitometry of heel | 4093 | Fractured heel (left) | No | No |
| **100018** | Bone-densitometry of heel | 4095 | Heel ultrasound method (right) | No | No |
| **100018** | Bone-densitometry of heel | 4096 | Fractured heel (right) | No | No |
| **100018** | Bone-densitometry of heel | 4100 | Ankle spacing width (left) | No | No |
| **100018** | Bone-densitometry of heel | 4101 | Heel broadband ultrasound attenuation (left) | No | No |
| **100018** | Bone-densitometry of heel | 4103 | Speed of sound through heel (left) | No | No |
| **100018** | Bone-densitometry of heel | 4104 | Heel quantitative ultrasound index (QUI), direct entry (left) | No | No |
| **100018** | Bone-densitometry of heel | 4105 | Heel bone mineral density (BMD) (left) | No | No |
| **100018** | Bone-densitometry of heel | 4106 | Heel bone mineral density (BMD) T-score, automated (left) | No | Yes |
| **100018** | Bone-densitometry of heel | 4119 | Ankle spacing width (right) | No | No |
| **100018** | Bone-densitometry of heel | 4120 | Heel broadband ultrasound attenuation (right) | No | No |
| **100018** | Bone-densitometry of heel | 4122 | Speed of sound through heel (right) | No | No |
| **100018** | Bone-densitometry of heel | 4123 | Heel quantitative ultrasound index (QUI), direct entry (right) | No | No |
| **100018** | Bone-densitometry of heel | 4124 | Heel bone mineral density (BMD) (right) | No | No |
| **100018** | Bone-densitometry of heel | 4125 | Heel bone mineral density (BMD) T-score, automated (right) | No | Yes |
| **100007** | Arterial stiffness | 4136 | Arterial pulse-wave stiffness device ID | Yes | Yes |
| **100018** | Bone-densitometry of heel | 4138 | Heel bone mineral density (BMD) T-score, manual entry (left) | No | Yes |
| **100018** | Bone-densitometry of heel | 4139 | Heel quantitative ultrasound index (QUI), manual entry (left) | No | No |
| **100018** | Bone-densitometry of heel | 4140 | Heel bone mineral density (BMD), manual entry (left) | No | No |
| **100018** | Bone-densitometry of heel | 4141 | Heel broadband ultrasound attenuation (BUA), manual entry (left) | No | No |
| **100018** | Bone-densitometry of heel | 4142 | Speed of sound through heel, manual entry (left) | No | No |
| **100018** | Bone-densitometry of heel | 4143 | Heel bone mineral density (BMD) T-score, manual entry (right) | No | Yes |
| **100018** | Bone-densitometry of heel | 4144 | Heel quantitative ultrasound index (QUI), manual entry (right) | No | No |
| **100018** | Bone-densitometry of heel | 4145 | Heel bone mineral density (BMD), manual entry (right) | No | No |
| **100018** | Bone-densitometry of heel | 4146 | Heel broadband ultrasound attenuation (BUA), manual entry (right) | No | No |
| **100018** | Bone-densitometry of heel | 4147 | Speed of sound through heel, manual entry (right) | No | No |
| **100007** | Arterial stiffness | 4186 | Stiffness method | No | No |
| **100007** | Arterial stiffness | 4194 | Pulse rate | Yes | Yes |
| **100007** | Arterial stiffness | 4195 | Pulse wave reflection index | No | No |
| **100007** | Arterial stiffness | 4196 | Pulse wave peak to peak time | No | No |
| **100007** | Arterial stiffness | 4198 | Position of the pulse wave peak | No | No |
| **100007** | Arterial stiffness | 4199 | Position of pulse wave notch | No | No |
| **100007** | Arterial stiffness | 4200 | Position of the shoulder on the pulse waveform | No | No |
| **100007** | Arterial stiffness | 4204 | Absence of notch position in the pulse waveform | No | No |
| **100007** | Arterial stiffness | 4205 | Pulse wave pressure versus time response curve | No | No |
| **100007** | Arterial stiffness | 4206 | Arterial stiffness device ID | Yes | Yes |
| **100007** | Arterial stiffness | 4207 | Pulse wave velocity (manual entry) | Yes | Yes |
| **100049** | Hearing test | 4229 | Triplet played (left) | No | No |
| **100049** | Hearing test | 4230 | Signal-to-noise-ratio (SNR) of triplet (left) | No | No |
| **100049** | Hearing test | 4232 | Triplet correct (left) | No | No |
| **100049** | Hearing test | 4233 | Mean signal-to-noise ratio (SNR), (left) | No | No |
| **100049** | Hearing test | 4234 | Time to press first digit (left) | No | No |
| **100049** | Hearing test | 4235 | Time to press last digit (left) | No | No |
| **100049** | Hearing test | 4236 | Triplet entered (left) | No | No |
| **100049** | Hearing test | 4237 | Time to press 'next' (left) | No | No |
| **100049** | Hearing test | 4238 | Keystroke history (left) | No | No |
| **100049** | Hearing test | 4239 | Number of times 'clear' was pressed (left) | No | No |
| **100049** | Hearing test | 4240 | Triplet played (right) | No | No |
| **100049** | Hearing test | 4241 | Signal-to-noise-ratio (SNR) of triplet (right) | No | No |
| **100049** | Hearing test | 4242 | Triplet entered (right) | No | No |
| **100049** | Hearing test | 4243 | Triplet correct (right) | No | No |
| **100049** | Hearing test | 4244 | Mean signal-to-noise ratio (SNR), (right) | No | No |
| **100049** | Hearing test | 4245 | Time to press first digit (right) | No | No |
| **100049** | Hearing test | 4246 | Time to press last digit (right) | No | No |
| **100049** | Hearing test | 4247 | Time to press 'next' (right) | No | No |
| **100049** | Hearing test | 4248 | Keystroke history (right) | No | No |
| **100049** | Hearing test | 4249 | Number of times 'clear' was pressed (right) | No | No |
| **100029** | Numeric memory | 4250 | Number of digits to be memorised/recalled | No | No |
| **100029** | Numeric memory | 4251 | Target number to be memorised | No | No |
| **100029** | Numeric memory | 4252 | Target number to be entered | No | No |
| **100029** | Numeric memory | 4253 | Time number displayed for | No | No |
| **100029** | Numeric memory | 4254 | Time first key touched | No | No |
| **100029** | Numeric memory | 4255 | Time last key touched | No | No |
| **100029** | Numeric memory | 4256 | Time elapsed | No | No |
| **100029** | Numeric memory | 4257 | Keystroke history | No | No |
| **100029** | Numeric memory | 4258 | Number entered by participant | No | No |
| **100029** | Numeric memory | 4259 | Digits entered correctly | No | No |
| **100029** | Numeric memory | 4260 | Round of numeric memory test | No | No |
| **100049** | Hearing test | 4268 | Completion status (left) | No | No |
| **100049** | Hearing test | 4269 | Number of triplets attempted (left) | No | No |
| **100049** | Hearing test | 4270 | Volume level set by participant (left) | No | No |
| **100049** | Hearing test | 4272 | Duration of hearing test (left) | No | No |
| **100049** | Hearing test | 4275 | Completion status (right) | No | No |
| **100049** | Hearing test | 4276 | Number of triplets attempted (right) | No | No |
| **100049** | Hearing test | 4277 | Volume level set by participant (right) | No | No |
| **100049** | Hearing test | 4279 | Duration of hearing test (right) | No | No |
| **100029** | Numeric memory | 4281 | Completion status of numeric memory test | No | No |
| **100029** | Numeric memory | 4282 | Maximum digits remembered correctly | No | No |
| **100029** | Numeric memory | 4283 | Number of rounds of numeric memory test performed | No | No |
| **100029** | Numeric memory | 4285 | Time to complete test | No | No |
| **100031** | Prospective memory | 4286 | Time when initial screen shown | No | No |
| **100031** | Prospective memory | 4287 | Test completion status | No | No |
| **100031** | Prospective memory | 4288 | Time to answer | No | No |
| **100031** | Prospective memory | 4289 | Time screen exited | No | No |
| **100031** | Prospective memory | 4290 | Duration screen displayed | No | No |
| **100031** | Prospective memory | 4291 | Number of attempts | No | No |
| **100031** | Prospective memory | 4292 | PM: initial answer | No | No |
| **100031** | Prospective memory | 4293 | PM: final answer | No | No |
| **100031** | Prospective memory | 4294 | Final attempt correct | No | No |
| **100031** | Prospective memory | 4295 | History of attempts | No | No |
| **100051** | Alcohol | 4407 | Average monthly red wine intake | No | No |
| **100051** | Alcohol | 4418 | Average monthly champagne plus white wine intake | No | No |
| **100051** | Alcohol | 4429 | Average monthly beer plus cider intake | No | No |
| **100051** | Alcohol | 4440 | Average monthly spirits intake | No | No |
| **100051** | Alcohol | 4451 | Average monthly fortified wine intake | No | No |
| **100051** | Alcohol | 4462 | Average monthly intake of other alcoholic drinks | No | No |
| **100034** | Family history | 4501 | Non-accidental death in close genetic family | No | No |
| **100060** | Mental health | 4526 | Happiness | No | No |
| **100060** | Mental health | 4537 | Work/job satisfaction | No | No |
| **100060** | Mental health | 4548 | Health satisfaction | No | No |
| **100060** | Mental health | 4559 | Family relationship satisfaction | No | No |
| **100060** | Mental health | 4570 | Friendships satisfaction | No | No |
| **100060** | Mental health | 4581 | Financial situation satisfaction | No | No |
| **100060** | Mental health | 4598 | Ever depressed for a whole week | No | No |
| **100060** | Mental health | 4609 | Longest period of depression | No | No |
| **100060** | Mental health | 4620 | Number of depression episodes | No | No |
| **100060** | Mental health | 4631 | Ever unenthusiastic/disinterested for a whole week | No | No |
| **100060** | Mental health | 4642 | Ever manic/hyper for 2 days | No | No |
| **100060** | Mental health | 4653 | Ever highly irritable/argumentative for 2 days | No | No |
| **100067** | Other sociodemographic factors | 4674 | Private healthcare | No | No |
| **100041** | Eyesight | 4689 | Age glaucoma diagnosed | Yes | Yes |
| **100041** | Eyesight | 4700 | Age cataract diagnosed | Yes | Yes |
| **100037** | Breathing | 4717 | Shortness of breath walking on level ground | No | No |
| **100038** | Claudication and peripheral artery disease | 4728 | Leg pain on walking | No | No |
| **100043** | Hearing | 4792 | Cochlear implant | No | No |
| **100043** | Hearing | 4803 | Tinnitus | No | No |
| **100043** | Hearing | 4814 | Tinnitus severity/nuisance | No | No |
| **100043** | Hearing | 4825 | Noisy workplace | No | No |
| **100043** | Hearing | 4836 | Loud music exposure frequency | No | No |
| **100049** | Hearing test | 4849 | Hearing test done | No | No |
| **100027** | Fluid intelligence / reasoning | 4924 | Attempted fluid intelligence (FI) test. | No | No |
| **100027** | Fluid intelligence / reasoning | 4935 | FI1 : numeric addition test | No | No |
| **100027** | Fluid intelligence / reasoning | 4946 | FI2 : identify largest number | No | No |
| **100027** | Fluid intelligence / reasoning | 4957 | FI3 : word interpolation | No | No |
| **100027** | Fluid intelligence / reasoning | 4968 | FI4 : positional arithmetic | No | No |
| **100027** | Fluid intelligence / reasoning | 4979 | FI5 : family relationship calculation | No | No |
| **100027** | Fluid intelligence / reasoning | 4990 | FI6 : conditional arithmetic | No | No |
| **100027** | Fluid intelligence / reasoning | 5001 | FI7 : synonym | No | No |
| **100027** | Fluid intelligence / reasoning | 5012 | FI8 : chained arithmetic | No | No |
| **100034** | Family history | 5057 | Number of older siblings | No | No |
| **100017** | Visual acuity | 5074 | Number of letters shown in round (left) | No | No |
| **100017** | Visual acuity | 5075 | Number of letters shown in round (right) | No | No |
| **100017** | Visual acuity | 5076 | Number of letters correct in round (right) | No | No |
| **100017** | Visual acuity | 5077 | Number of letters correct in round (left) | No | No |
| **100017** | Visual acuity | 5078 | logMAR in round (left) | Yes | Yes |
| **100017** | Visual acuity | 5079 | logMAR in round (right) | Yes | Yes |
| **100017** | Visual acuity | 5080 | Displayed letters in round (right) | No | No |
| **100017** | Visual acuity | 5081 | Displayed letters in round (left) | No | No |
| **100017** | Visual acuity | 5082 | Visual acuity result in round (left) | No | No |
| **100017** | Visual acuity | 5083 | Visual acuity result in round (right) | No | No |
| **100014** | Autorefraction | 5084 | Spherical power (right) | No | No |
| **100014** | Autorefraction | 5085 | Spherical power (left) | No | No |
| **100014** | Autorefraction | 5086 | Cylindrical power (left) | No | No |
| **100014** | Autorefraction | 5087 | Cylindrical power (right) | No | No |
| **100014** | Autorefraction | 5088 | Astigmatism angle (right) | No | No |
| **100014** | Autorefraction | 5089 | Astigmatism angle (left) | No | No |
| **100014** | Autorefraction | 5090 | Refractometry result unreliable (left) | No | No |
| **100014** | Autorefraction | 5091 | Refractometry result unreliable (right) | No | No |
| **100014** | Autorefraction | 5096 | 3mm weak meridian (left) | No | No |
| **100014** | Autorefraction | 5097 | 6mm weak meridian (left) | No | No |
| **100014** | Autorefraction | 5098 | 6mm weak meridian (right) | No | No |
| **100014** | Autorefraction | 5099 | 3mm weak meridian (right) | No | No |
| **100014** | Autorefraction | 5100 | 3mm weak meridian angle (right) | No | No |
| **100014** | Autorefraction | 5101 | 6mm weak meridian angle (right) | No | No |
| **100014** | Autorefraction | 5102 | 6mm weak meridian angle (left) | No | No |
| **100014** | Autorefraction | 5103 | 3mm weak meridian angle (left) | No | No |
| **100014** | Autorefraction | 5104 | 3mm strong meridian angle (left) | No | No |
| **100014** | Autorefraction | 5105 | 6mm strong meridian angle (left) | No | No |
| **100014** | Autorefraction | 5106 | 6mm strong meridian angle (right) | No | No |
| **100014** | Autorefraction | 5107 | 3mm strong meridian angle (right) | No | No |
| **100014** | Autorefraction | 5108 | 3mm asymmetry angle (right) | No | No |
| **100014** | Autorefraction | 5109 | 6mm asymmetry angle (right) | No | No |
| **100014** | Autorefraction | 5110 | 6mm asymmetry angle (left) | No | No |
| **100014** | Autorefraction | 5111 | 3mm asymmetry angle (left) | No | No |
| **100014** | Autorefraction | 5112 | 3mm cylindrical power angle (left) | No | No |
| **100014** | Autorefraction | 5113 | 6mm cylindrical power angle (left) | No | No |
| **100014** | Autorefraction | 5114 | 6mm cylindrical power angle (right) | No | No |
| **100014** | Autorefraction | 5115 | 3mm cylindrical power angle (right) | No | No |
| **100014** | Autorefraction | 5116 | 3mm cylindrical power (right) | No | No |
| **100014** | Autorefraction | 5117 | 6mm cylindrical power (right) | No | No |
| **100014** | Autorefraction | 5118 | 6mm cylindrical power (left) | No | No |
| **100014** | Autorefraction | 5119 | 3mm cylindrical power (left) | No | No |
| **100014** | Autorefraction | 5132 | 3mm strong meridian (right) | No | No |
| **100014** | Autorefraction | 5133 | 6mm strong meridian (right) | No | No |
| **100014** | Autorefraction | 5134 | 6mm strong meridian (left) | No | No |
| **100014** | Autorefraction | 5135 | 3mm strong meridian (left) | No | No |
| **100014** | Autorefraction | 5136 | 3mm keratometry result unreliable (left) | No | No |
| **100014** | Autorefraction | 5138 | 6mm keratometry result unreliable (left) | No | No |
| **100014** | Autorefraction | 5139 | 6mm keratometry result unreliable (right) | No | No |
| **100014** | Autorefraction | 5140 | 3mm keratometry result unreliable (right) | No | No |
| **100014** | Autorefraction | 5141 | 3mm asymmetry index unreliable (left) | No | No |
| **100014** | Autorefraction | 5142 | 6mm asymmetry index unreliable (left) | No | No |
| **100014** | Autorefraction | 5143 | 6mm asymmetry index unreliable (right) | No | No |
| **100014** | Autorefraction | 5144 | 3mm asymmetry index unreliable (right) | No | No |
| **100014** | Autorefraction | 5145 | 3mm regularity index unreliable (right) | No | No |
| **100014** | Autorefraction | 5146 | 6mm regularity index unreliable (right) | No | No |
| **100014** | Autorefraction | 5147 | 6mm regularity index unreliable (left) | No | No |
| **100014** | Autorefraction | 5148 | 3mm regularity index unreliable (left) | No | No |
| **100014** | Autorefraction | 5149 | 3mm regularity index for irregular astigmatism level (left) | No | No |
| **100014** | Autorefraction | 5152 | 3mm asymmetry index for irregular astigmatism level (right) | No | No |
| **100014** | Autorefraction | 5155 | 3mm asymmetry index for irregular astigmatism level (left) | No | No |
| **100014** | Autorefraction | 5156 | 3mm asymmetry index (left) | No | No |
| **100014** | Autorefraction | 5157 | 6mm asymmetry index (left) | No | No |
| **100014** | Autorefraction | 5158 | 6mm asymmetry index (right) | No | No |
| **100014** | Autorefraction | 5159 | 3mm asymmetry index (right) | No | No |
| **100014** | Autorefraction | 5160 | 3mm regularity index (right) | No | No |
| **100014** | Autorefraction | 5161 | 6mm regularity index (right) | No | No |
| **100014** | Autorefraction | 5162 | 6mm regularity index (left) | No | No |
| **100014** | Autorefraction | 5163 | 3mm regularity index (left) | No | No |
| **100014** | Autorefraction | 5164 | 3mm regularity index for irregular astigmatism level (right) | No | No |
| **100099** | Eye surgery/complications | 5181 | Ever had eye surgery | No | No |
| **100099** | Eye surgery/complications | 5182 | Both eyes present | No | No |
| **100099** | Eye surgery/complications | 5183 | Current eye infection | No | No |
| **100017** | Visual acuity | 5185 | Visual acuity measured (right) | No | No |
| **100017** | Visual acuity | 5186 | Duration visual-acuity screen displayed (right) | No | No |
| **100017** | Visual acuity | 5187 | Visual acuity measured (left) | No | No |
| **100017** | Visual acuity | 5188 | Duration visual-acuity screen displayed (left) | No | No |
| **100014** | Autorefraction | 5189 | Auto-refraction method (right) | No | No |
| **100014** | Autorefraction | 5190 | Duration at which refractometer first shown (right) | No | No |
| **100014** | Autorefraction | 5191 | Auto-refraction method (left) | No | No |
| **100014** | Autorefraction | 5193 | Duration at which refractometer first shown (left) | No | No |
| **100015** | Intraocular pressure | 5194 | Intra-ocular pressure (IOP) method (right) | No | No |
| **100015** | Intraocular pressure | 5196 | Intra-ocular pressure (IOP) method (left) | No | No |
| **152** | Process durations | 5198 | Eye measures duration | No | No |
| **100017** | Visual acuity | 5199 | logMAR, initial (right) | No | Yes |
| **100017** | Visual acuity | 5200 | Final number of letters displayed (right) | No | No |
| **100017** | Visual acuity | 5201 | logMAR, final (right) | No | Yes |
| **100017** | Visual acuity | 5202 | Number of rounds to result (right) | No | No |
| **100017** | Visual acuity | 5204 | Distance of viewer to screen (right) | Yes | Yes |
| **100017** | Visual acuity | 5205 | Direct or mirror view (right) | No | No |
| **100017** | Visual acuity | 5206 | logMAR, initial (left) | No | Yes |
| **100017** | Visual acuity | 5207 | Final number of letters displayed (left) | No | No |
| **100017** | Visual acuity | 5208 | logMAR, final (left) | No | Yes |
| **100017** | Visual acuity | 5209 | Number of rounds to result (left) | No | No |
| **100017** | Visual acuity | 5211 | Distance of viewer to screen (left) | Yes | Yes |
| **100017** | Visual acuity | 5212 | Direct or mirror view (left) | No | No |
| **100014** | Autorefraction | 5214 | Auto-refractor device ID (right) | Yes | Yes |
| **100014** | Autorefraction | 5215 | Vertex distance (right) | No | No |
| **100014** | Autorefraction | 5221 | Index of best refractometry result (right) | No | No |
| **100014** | Autorefraction | 5237 | 3mm index of best keratometry results (right) | No | No |
| **100014** | Autorefraction | 5251 | 6mm index of best keratometry results (right) | No | No |
| **100015** | Intraocular pressure | 5253 | Intra-ocular pressure device ID (right) | Yes | Yes |
| **100015** | Intraocular pressure | 5254 | Intra-ocular pressure, corneal-compensated (right) | No | Yes |
| **100015** | Intraocular pressure | 5255 | Intra-ocular pressure, Goldmann-correlated (right) | No | Yes |
| **100015** | Intraocular pressure | 5256 | Corneal hysteresis (right) | No | No |
| **100015** | Intraocular pressure | 5257 | Corneal resistance factor (right) | No | No |
| **100015** | Intraocular pressure | 5258 | Applanation curve (right) | No | No |
| **100015** | Intraocular pressure | 5259 | Pressure curve (right) | No | No |
| **100015** | Intraocular pressure | 5261 | Intra-ocular pressure device ID (left) | Yes | Yes |
| **100015** | Intraocular pressure | 5262 | Intra-ocular pressure, corneal-compensated (left) | No | Yes |
| **100015** | Intraocular pressure | 5263 | Intra-ocular pressure, Goldmann-correlated (left) | No | Yes |
| **100015** | Intraocular pressure | 5264 | Corneal hysteresis (left) | No | No |
| **100015** | Intraocular pressure | 5265 | Corneal resistance factor (left) | No | No |
| **100015** | Intraocular pressure | 5266 | Applanation curve (left) | No | No |
| **100015** | Intraocular pressure | 5267 | Pressure curve (left) | No | No |
| **100016** | Retinal optical coherence tomography | 5270 | Optical-coherence tomography device ID | Yes | Yes |
| **100014** | Autorefraction | 5273 | Auto-refractor device ID (left) | Yes | Yes |
| **100014** | Autorefraction | 5274 | Vertex distance (left) | No | No |
| **100014** | Autorefraction | 5276 | Index of best refractometry result (left) | No | No |
| **100014** | Autorefraction | 5292 | 3mm index of best keratometry results (left) | No | No |
| **100014** | Autorefraction | 5306 | 6mm index of best keratometry results (left) | No | No |
| **100099** | Eye surgery/complications | 5324 | Ever had cataract surgery | No | No |
| **100099** | Eye surgery/complications | 5325 | Ever had refractive laser eye surgery | No | No |
| **100099** | Eye surgery/complications | 5326 | Ever had surgery for glaucoma or high eye pressure | No | No |
| **100099** | Eye surgery/complications | 5327 | Ever had laser treatment for glaucoma or high eye pressure | No | No |
| **100099** | Eye surgery/complications | 5328 | Ever had corneal graft surgery | No | No |
| **100051** | Alcohol | 5364 | Average weekly intake of other alcoholic drinks | No | No |
| **100060** | Mental health | 5375 | Longest period of unenthusiasm / disinterest | No | No |
| **100060** | Mental health | 5386 | Number of unenthusiastic/disinterested episodes | No | No |
| **100041** | Eyesight | 5408 | Which eye(s) affected by amblyopia (lazy eye) | No | No |
| **100041** | Eyesight | 5419 | Which eye(s) affected by injury or trauma resulting in loss of vision | No | No |
| **100041** | Eyesight | 5430 | Age when loss of vision due to injury or trauma diagnosed | No | No |
| **100041** | Eyesight | 5441 | Which eye(s) are affected by cataract | No | No |
| **100038** | Claudication and peripheral artery disease | 5452 | Leg pain when standing still or sitting | No | No |
| **100038** | Claudication and peripheral artery disease | 5463 | Leg pain in calf/calves | No | No |
| **100038** | Claudication and peripheral artery disease | 5474 | Leg pain when walking uphill or hurrying | No | No |
| **100038** | Claudication and peripheral artery disease | 5485 | Leg pain when walking normally | No | No |
| **100038** | Claudication and peripheral artery disease | 5496 | Leg pain when walking ever disappears while walking | No | No |
| **100038** | Claudication and peripheral artery disease | 5507 | Leg pain on walking : action taken | No | No |
| **100038** | Claudication and peripheral artery disease | 5518 | Leg pain on walking : effect of standing still | No | No |
| **100038** | Claudication and peripheral artery disease | 5529 | Surgery on leg arteries (other than for varicose veins) | No | No |
| **100038** | Claudication and peripheral artery disease | 5540 | Surgery/amputation of toe or leg | No | No |
| **100027** | Fluid intelligence / reasoning | 5556 | FI9 : concept interpolation | No | No |
| **100041** | Eyesight | 5610 | Which eye(s) affected by presbyopia | No | No |
| **100060** | Mental health | 5663 | Length of longest manic/irritable episode | No | No |
| **100060** | Mental health | 5674 | Severity of manic/irritable episodes | No | No |
| **100027** | Fluid intelligence / reasoning | 5699 | FI10 : arithmetic sequence recognition | No | No |
| **100027** | Fluid intelligence / reasoning | 5779 | FI11 : antonym | No | No |
| **100027** | Fluid intelligence / reasoning | 5790 | FI12 : square sequence recognition | No | No |
| **100041** | Eyesight | 5832 | Which eye(s) affected by hypermetropia (long sight) | No | No |
| **100041** | Eyesight | 5843 | Which eye(s) affected by myopia (short sight) | No | No |
| **100041** | Eyesight | 5855 | Which eye(s) affected by astigmatism | No | No |
| **100027** | Fluid intelligence / reasoning | 5866 | FI13 : subset inclusion logic | No | No |
| **100041** | Eyesight | 5877 | Which eye(s) affected by other eye condition | No | No |
| **100041** | Eyesight | 5890 | Which eye(s) affected by diabetes-related eye disease | No | No |
| **100041** | Eyesight | 5901 | Age when diabetes-related eye disease diagnosed | No | No |
| **100041** | Eyesight | 5912 | Which eye(s) affected by macular degeneration | No | No |
| **100041** | Eyesight | 5923 | Age macular degeneration diagnosed | No | No |
| **100041** | Eyesight | 5934 | Which eye(s) affected by other serious eye condition | No | No |
| **100041** | Eyesight | 5945 | Age other serious eye condition diagnosed | No | No |
| **100058** | Smoking | 5959 | Previously smoked cigarettes on most/all days | No | No |
| **100012** | ECG during exercise | 5983 | ECG, heart rate | No | Yes |
| **100012** | ECG during exercise | 5984 | ECG, load | No | No |
| **100012** | ECG during exercise | 5985 | Bicycle speed | No | No |
| **100012** | ECG during exercise | 5986 | ECG, phase time | No | No |
| **100012** | ECG during exercise | 5987 | ECG, trend phase name | No | No |
| **100012** | ECG during exercise | 5988 | ECG, stage name | No | No |
| **100012** | ECG during exercise | 5990 | ECG, stage duration | No | No |
| **100012** | ECG during exercise | 5991 | ECG, phase name | No | No |
| **100012** | ECG during exercise | 5992 | ECG, phase duration | No | No |
| **100012** | ECG during exercise | 5993 | ECG, number of stages in a phase | No | No |
| **100012** | ECG during exercise | 6014 | Doctor restricts physical activity due to heart condition | No | No |
| **100012** | ECG during exercise | 6015 | Chest pain felt during physical activity | No | No |
| **100012** | ECG during exercise | 6016 | Chest pain felt outside physical activity | No | No |
| **100012** | ECG during exercise | 6017 | Able to walk or cycle unaided for 10 minutes | No | No |
| **100012** | ECG during exercise | 6019 | ECG/bike method for fitness test | No | No |
| **100012** | ECG during exercise | 6020 | Completion status of test | No | No |
| **152** | Process durations | 6022 | ECG during exercise duration | No | No |
| **100012** | ECG during exercise | 6023 | Description of exercise protocol recommended | No | No |
| **100012** | ECG during exercise | 6024 | Program category | No | No |
| **100012** | ECG during exercise | 6025 | Fitness test results, including ECG data | No | No |
| **100012** | ECG during exercise | 6032 | Maximum workload during fitness test | No | No |
| **100012** | ECG during exercise | 6033 | Maximum heart rate during fitness test | Yes | Yes |
| **100012** | ECG during exercise | 6034 | Target heart rate achieved | No | No |
| **100012** | ECG during exercise | 6038 | Number of trend entries | No | No |
| **100012** | ECG during exercise | 6039 | Duration of fitness test | Yes | Yes |
| **100016** | Retinal optical coherence tomography | 6070 | OCT measured (right) | No | No |
| **100016** | Retinal optical coherence tomography | 6071 | Duration at which OCT screen shown (right) | No | No |
| **100016** | Retinal optical coherence tomography | 6072 | OCT measured (left) | No | No |
| **100016** | Retinal optical coherence tomography | 6073 | Duration at which OCT screen shown (left) | No | No |
| **100017** | Visual acuity | 6074 | Glasses worn/required (right) | No | No |
| **100017** | Visual acuity | 6075 | Glasses worn/required (left) | No | No |
| **100041** | Eyesight | 6119 | Which eye(s) affected by glaucoma | No | No |
| **100063** | Education | 6138 | Qualifications | No | No |
| **100066** | Household | 6139 | Gas or solid-fuel cooking/heating | No | No |
| **100066** | Household | 6140 | Heating type(s) in home | No | No |
| **100066** | Household | 6141 | How are people in household related to participant | No | No |
| **100064** | Employment | 6142 | Current employment status | No | No |
| **100064** | Employment | 6143 | Transport type for commuting to job workplace | No | No |
| **100052** | Diet | 6144 | Never eat eggs, dairy, wheat, sugar | No | No |
| **100060** | Mental health | 6145 | Illness, injury, bereavement, stress in last 2 years | No | No |
| **100067** | Other sociodemographic factors | 6146 | Attendance/disability/mobility allowance | No | No |
| **100041** | Eyesight | 6147 | Reason for glasses/contact lenses | No | No |
| **100041** | Eyesight | 6148 | Eye problems/disorders | No | No |
| **100046** | Mouth | 6149 | Mouth/teeth dental problems | No | No |
| **100044** | Medical conditions | 6150 | Vascular/heart problems diagnosed by doctor | Yes | Yes |
| **100044** | Medical conditions | 6151 | Fractured bone site(s) | Yes | Yes |
| **100044** | Medical conditions | 6152 | Blood clot, DVT, bronchitis, emphysema, asthma, rhinitis, eczema, allergy diagnosed by doctor | No | Yes |
| **100045** | Medication | 6153 | Medication for cholesterol, blood pressure, diabetes, or take exogenous hormones | No | No |
| **100045** | Medication | 6154 | Medication for pain relief, constipation, heartburn | No | No |
| **100045** | Medication | 6155 | Vitamin and mineral supplements | No | No |
| **100060** | Mental health | 6156 | Manic/hyper symptoms | No | No |
| **100058** | Smoking | 6157 | Why stopped smoking | No | No |
| **100058** | Smoking | 6158 | Why reduced smoking | No | No |
| **100048** | Pain | 6159 | Pain type(s) experienced in last month | No | No |
| **100061** | Social support | 6160 | Leisure/social activities | No | No |
| **100054** | Physical activity | 6162 | Types of transport used (excluding work) | No | No |
| **100054** | Physical activity | 6164 | Types of physical activity in last 4 weeks | No | No |
| **100045** | Medication | 6177 | Medication for cholesterol, blood pressure or diabetes | No | No |
| **100045** | Medication | 6179 | Mineral and other dietary supplements | No | No |
| **100058** | Smoking | 6183 | Number of cigarettes previously smoked daily (current cigar/pipe smokers) | No | No |
| **100058** | Smoking | 6194 | Age stopped smoking cigarettes (current cigar/pipe or previous cigarette smoker) | No | No |
| **100041** | Eyesight | 6205 | Which eye(s) affected by strabismus (squint) | No | No |
| **100009** | Impedance measures | 6218 | Impedance of whole body, manual entry | No | No |
| **100009** | Impedance measures | 6219 | Impedance of leg, manual entry (right) | No | No |
| **100009** | Impedance measures | 6220 | Impedance of leg, manual entry (left) | No | No |
| **100009** | Impedance measures | 6221 | Impedance of arm, manual entry (right) | No | No |
| **100009** | Impedance measures | 6222 | Impedance of arm, manual entry (left) | No | No |
| **503** | Tower rearranging | 6312 | Value entered | No | No |
| **503** | Tower rearranging | 6313 | Duration to entering selection | No | No |
| **504** | Picture vocabulary | 6314 | Word/picture group used for each round | No | No |
| **504** | Picture vocabulary | 6315 | Picture selected for each round | No | No |
| **504** | Picture vocabulary | 6317 | Duration of selection in each round | No | No |
| **502** | Symbol digit substitution | 6325 | Duration to entering symbol choice | No | No |
| **501** | Matrix pattern completion | 6332 | Item selected for each puzzle | No | No |
| **501** | Matrix pattern completion | 6333 | Duration spent answering each puzzle | No | No |
| **100030** | Pairs matching | 6334 | Screen layout | No | No |
| **505** | Trail making | 6348 | Duration to complete numeric path (trail #1) | No | No |
| **505** | Trail making | 6349 | Total errors traversing numeric path (trail #1) | No | No |
| **505** | Trail making | 6350 | Duration to complete alphanumeric path (trail #2) | No | No |
| **505** | Trail making | 6351 | Total errors traversing alphanumeric path (trail #2) | No | No |
| **502** | Symbol digit substitution | 6362 | First code array presented | No | No |
| **504** | Picture vocabulary | 6364 | Vocabulary level | No | No |
| **504** | Picture vocabulary | 6365 | Uncertainty in vocabulary level | No | No |
| **501** | Matrix pattern completion | 6373 | Number of puzzles correctly solved | No | No |
| **501** | Matrix pattern completion | 6374 | Number of puzzles viewed | No | No |
| **503** | Tower rearranging | 6383 | Number of puzzles attempted | No | No |
| **506** | Paired associate learning | 6448 | Word associated with "huge" | No | No |
| **506** | Paired associate learning | 6459 | Word associated with "happy" | No | No |
| **506** | Paired associate learning | 6470 | Word associated with "tattered" | No | No |
| **506** | Paired associate learning | 6481 | Word associated with "old" | No | No |
| **506** | Paired associate learning | 6492 | Word associated with "long" | No | No |
| **506** | Paired associate learning | 6503 | Word associated with "red" | No | No |
| **506** | Paired associate learning | 6514 | Word associated with "sulking" | No | No |
| **506** | Paired associate learning | 6525 | Word associated with "pretty" | No | No |
| **506** | Paired associate learning | 6536 | Word associated with "tiny" | No | No |
| **506** | Paired associate learning | 6547 | Word associated with "new" | No | No |
| **100075** | Medications | 6671 | Number of antibiotics taken in last 3 months | No | No |
| **505** | Trail making | 6770 | Errors before selecting correct item in numeric path (trail #1) | No | No |
| **505** | Trail making | 6771 | Errors before selecting correct item in alphanumeric path (trail #2) | No | No |
| **505** | Trail making | 6772 | Interval between previous point and current one in numeric path (trail #1) | No | No |
| **505** | Trail making | 6773 | Interval between previous point and current one in alphanumeric path (trail #2) | No | No |
| **100045** | Medication | 10004 | Medication for pain relief, constipation, heartburn (pilot) | No | No |
| **100045** | Medication | 10005 | Medication for smoking cessation, constipation, heartburn, allergies (pilot) | No | No |
| **100046** | Mouth | 10006 | Mouth/teeth dental problems (pilot) | No | No |
| **100045** | Medication | 10007 | Vitamin and mineral supplements (pilot) | No | No |
| **100053** | Electronic device use | 10016 | Regular use of hands-free device/speakerphone with mobile phone (pilot) | No | No |
| **100053** | Electronic device use | 10105 | Internet user (pilot) | No | No |
| **100053** | Electronic device use | 10114 | Willing to be contacted by email (pilot) | No | No |
| **100058** | Smoking | 10115 | Why stopped smoking (pilot) | No | No |
| **100069** | Female-specific factors | 10132 | Type of progestan-only oral contraceptive used (pilot) | No | No |
| **100030** | Pairs matching | 10133 | Number of columns displayed in round (pilot) | No | No |
| **100030** | Pairs matching | 10134 | Number of rows displayed in round (pilot) | No | No |
| **100030** | Pairs matching | 10136 | Number of correct matches in round (pilot) | No | No |
| **100030** | Pairs matching | 10137 | Number of incorrect matches in round (pilot) | No | No |
| **100030** | Pairs matching | 10138 | Time to complete round (pilot) | No | No |
| **100032** | Reaction time | 10139 | Index for card A in round (pilot) | No | No |
| **100032** | Reaction time | 10140 | Index for card B in round (pilot) | No | No |
| **100032** | Reaction time | 10141 | Number of times snap-button pressed (pilot) | No | No |
| **100028** | Lights pattern memory | 10142 | Number of columns displayed (pilot) | No | No |
| **100028** | Lights pattern memory | 10143 | Number of rows displayed (pilot) | No | No |
| **100028** | Lights pattern memory | 10144 | Time taken to complete lights test (pilot) | No | No |
| **100028** | Lights pattern memory | 10145 | Pattern of lights displayed (pilot) | No | No |
| **100028** | Lights pattern memory | 10146 | Pattern of lights as remembered (pilot) | No | No |
| **100032** | Reaction time | 10147 | Duration to first press of snap-button in each round (pilot) | No | No |
| **152** | Process durations | 10241 | Touchscreen duration (pilot) | No | No |
| **100077** | Word production | 10609 | Words answer array (pilot) | No | No |
| **100077** | Word production | 10610 | Word count (pilot) | No | No |
| **100077** | Word production | 10612 | Number of words beginning with 'S' (pilot) | No | No |
| **100020** | Spirometry | 10691 | Result ranking (pilot) | No | No |
| **100020** | Spirometry | 10693 | Acceptability of each blow result (pilot) | No | No |
| **100020** | Spirometry | 10694 | Forced vital capacity (FVC) (pilot) | Yes | Yes |
| **100020** | Spirometry | 10695 | Forced expiratory volume in 1-second (FEV1) (pilot) | Yes | Yes |
| **100020** | Spirometry | 10696 | Peak expiratory flow (PEF) (pilot) | Yes | Yes |
| **100020** | Spirometry | 10697 | Data points for blow (pilot) | No | No |
| **100020** | Spirometry | 10711 | Spirometry method (pilot) | No | No |
| **100020** | Spirometry | 10714 | Spirometry device serial number (pilot) | No | No |
| **100020** | Spirometry | 10717 | Number of measurements made (pilot) | Yes | Yes |
| **100060** | Mental health | 10721 | Illness, injury, bereavement, stress in last 2 years (pilot) | No | No |
| **100063** | Education | 10722 | Qualifications (pilot) | No | No |
| **100045** | Medication | 10723 | Vitamin supplements (pilot) | No | No |
| **100061** | Social support | 10740 | Frequency of friend/family visits (pilot) | No | No |
| **100053** | Electronic device use | 10749 | Time using mobile phone in last 3 months (pilot) | No | No |
| **100052** | Diet | 10767 | Spread type (pilot) | No | No |
| **100052** | Diet | 10776 | Bread type/intake (pilot) | No | No |
| **100043** | Hearing | 10793 | Hearing difficulty/problems (pilot) | No | No |
| **100051** | Alcohol | 10818 | Reason for reducing amount of alcohol drunk (pilot) | No | No |
| **100058** | Smoking | 10827 | Ever stopped smoking for 6+ months (pilot) | No | No |
| **100044** | Medical conditions | 10844 | Gestational diabetes only (pilot) | Yes | Yes |
| **100051** | Alcohol | 10853 | Reason former drinker stopped drinking alcohol (pilot) | No | No |
| **100045** | Medication | 10854 | Other dietary supplements (pilot) | No | No |
| **100052** | Diet | 10855 | Never eat eggs, dairy, wheat, sugar (pilot) | No | No |
| **100066** | Household | 10860 | Gas or solid-fuel cooking/heating (pilot) | No | No |
| **100066** | Household | 10877 | Average total household income before tax (pilot) | No | No |
| **100053** | Electronic device use | 10886 | Difference in mobile phone use compared to one year previously (pilot) | No | No |
| **100058** | Smoking | 10895 | Light smokers, at least 100 smokes in lifetime (pilot) | No | No |
| **100052** | Diet | 10912 | Variation in diet (pilot) | No | No |
| **100054** | Physical activity | 10953 | Duration of walks (pilot) | No | No |
| **100054** | Physical activity | 10962 | Duration of moderate physical activity (pilot) | No | No |
| **100054** | Physical activity | 10971 | Duration of vigorous physical activity (pilot) | No | No |
| **100** | Brain MRI | 12139 | Believed safe to perform brain MRI scan | No | No |
| **105** | Abdominal MRI | 12140 | Believed safe to perform abdominal MRI scan | No | No |
| **103** | DXA assessment | 12141 | Believed safe to perform DXA scan | No | No |
| **100010** | Body size measures | 12143 | Weight (pre-imaging) | No | Yes |
| **100010** | Body size measures | 12144 | Height | No | Yes |
| **100004** | Procedural metrics | 12148 | Number of imaging stages blocked at screening | No | No |
| **100** | Brain MRI | 12187 | Brain MRI measuring method | No | No |
| **100** | Brain MRI | 12188 | Operator indicated brain MRI measurement completed | No | No |
| **105** | Abdominal MRI | 12223 | Abdominal MRI measuring method | No | No |
| **105** | Abdominal MRI | 12224 | Abdominal MRI measurement completed | No | No |
| **103** | DXA assessment | 12253 | DXA measuring method | No | No |
| **103** | DXA assessment | 12254 | DXA measurement completed | No | No |
| **101** | Carotid ultrasound | 12291 | Carotid ultrasound measuring method | No | No |
| **101** | Carotid ultrasound | 12292 | Carotid ultrasound measurement completed | No | No |
| **104** | ECG at rest, 12-lead | 12323 | 12-lead ECG measuring method | No | No |
| **104** | ECG at rest, 12-lead | 12336 | Ventricular rate | No | No |
| **104** | ECG at rest, 12-lead | 12338 | P duration | No | No |
| **104** | ECG at rest, 12-lead | 12340 | QRS duration | No | No |
| **102** | Heart MRI | 12624 | Identifier for blood pressure device | No | No |
| **106** | Task functional brain MRI | 12651 | Duration of eprime test | No | No |
| **100** | Brain MRI | 12652 | Reason brain MRI not performed | No | No |
| **104** | ECG at rest, 12-lead | 12653 | ECG automated diagnoses | No | No |
| **104** | ECG at rest, 12-lead | 12654 | Number of automated diagnostic comments recorded during 12-lead ECG | No | No |
| **104** | ECG at rest, 12-lead | 12657 | Suspicious flag for 12-lead ECG | No | No |
| **104** | ECG at rest, 12-lead | 12658 | Identifier for 12-lead ECG device | No | No |
| **100** | Brain MRI | 12663 | Reason believed unsafe to perform brain MRI | No | No |
| **128** | Pulse wave analysis | 12671 | PWA start time | No | No |
| **128** | Pulse wave analysis | 12673 | Heart rate during PWA | No | No |
| **128** | Pulse wave analysis | 12674 | Systolic brachial blood pressure during PWA | No | No |
| **128** | Pulse wave analysis | 12675 | Diastolic brachial blood pressure during PWA | No | No |
| **128** | Pulse wave analysis | 12676 | Peripheral pulse pressure during PWA | No | No |
| **128** | Pulse wave analysis | 12677 | Central systolic blood pressure during PWA | No | No |
| **128** | Pulse wave analysis | 12678 | Central pulse pressure during PWA | No | No |
| **128** | Pulse wave analysis | 12679 | Number of beats in waveform average for PWA | No | No |
| **128** | Pulse wave analysis | 12680 | Central augmentation pressure during PWA | No | No |
| **128** | Pulse wave analysis | 12681 | Augmentation index for PWA | No | No |
| **128** | Pulse wave analysis | 12682 | Cardiac output during PWA | No | No |
| **128** | Pulse wave analysis | 12683 | End systolic pressure during PWA | No | No |
| **128** | Pulse wave analysis | 12684 | End systolic pressure index during PWA | No | No |
| **128** | Pulse wave analysis | 12685 | Total peripheral resistance during PWA | No | No |
| **128** | Pulse wave analysis | 12686 | Stroke volume during PWA | No | No |
| **128** | Pulse wave analysis | 12687 | Mean arterial pressure during PWA | No | No |
| **128** | Pulse wave analysis | 12688 | Vicorder trace | No | No |
| **128** | Pulse wave analysis | 12695 | Blood pressure test start time | No | No |
| **128** | Pulse wave analysis | 12697 | Systolic brachial blood pressure | No | No |
| **128** | Pulse wave analysis | 12698 | Diastolic brachial blood pressure | No | No |
| **128** | Pulse wave analysis | 12699 | Number of PWA tests performed | No | No |
| **128** | Pulse wave analysis | 12700 | Vicorder results plausible | No | No |
| **128** | Pulse wave analysis | 12702 | Cardiac index during PWA | No | No |
| **100** | Brain MRI | 12704 | Reason brain MRI not completed | No | No |
| **106** | Task functional brain MRI | 12706 | Eprime error flag | No | No |
| **100074** | Medical conditions | 20001 | Cancer code, self-reported | No | Yes |
| **100074** | Medical conditions | 20002 | Non-cancer illness code, self-reported | No | Yes |
| **100075** | Medications | 20003 | Treatment/medication code | No | Yes |
| **100076** | Operations | 20004 | Operation code | No | Yes |
| **2** | Ongoing characteristics | 20005 | Email access | No | No |
| **100074** | Medical conditions | 20006 | Interpolated Year when cancer first diagnosed | No | No |
| **100074** | Medical conditions | 20007 | Interpolated Age of participant when cancer first diagnosed | Yes | Yes |
| **100074** | Medical conditions | 20008 | Interpolated Year when non-cancer illness first diagnosed | Yes | Yes |
| **100074** | Medical conditions | 20009 | Interpolated Age of participant when non-cancer illness first diagnosed | Yes | Yes |
| **100076** | Operations | 20010 | Interpolated Year when operation took place | Yes | Yes |
| **100076** | Operations | 20011 | Interpolated Age of participant when operation took place | No | No |
| **100074** | Medical conditions | 20012 | Method of recording time when cancer first diagnosed | No | No |
| **100074** | Medical conditions | 20013 | Method of recording time when non-cancer illness first diagnosed | No | No |
| **100076** | Operations | 20014 | Method of recording time when operation occurred | No | No |
| **100010** | Body size measures | 20015 | Sitting height | Yes | Yes |
| **100027** | Fluid intelligence / reasoning | 20016 | Fluid intelligence score | No | No |
| **100031** | Prospective memory | 20018 | Prospective memory result | No | No |
| **100049** | Hearing test | 20019 | Speech-reception-threshold (SRT) estimate (left) | No | No |
| **100049** | Hearing test | 20021 | Speech-reception-threshold (SRT) estimate (right) | No | No |
| **100072** | Early life factors | 20022 | Birth weight | No | No |
| **100032** | Reaction time | 20023 | Mean time to correctly identify matches | No | No |
| **100073** | Employment | 20024 | Job code at visit - deduced | Yes | Yes |
| **100096** | Saliva sample collection | 20025 | Time saliva sample collected | No | No |
| **100020** | Spirometry | 20031 | Acceptability of each blow result (text) | No | No |
| **100020** | Spirometry | 20032 | Acceptability of each blow result (text) (pilot) | No | No |
| **100024** | Reception | 20033 | Home location at assessment - east co-ordinate | No | No |
| **100024** | Reception | 20034 | Home location at assessment - north co-ordinate | No | No |
| **100095** | Urine sample collection | 20035 | Time urine sample collected | No | No |
| **100010** | Body size measures | 20041 | Reason for skipping weight | No | No |
| **100020** | Spirometry | 20042 | Reason for skipping spirometry | No | No |
| **100019** | Hand grip strength | 20043 | Reason for skipping grip strength (right) | No | No |
| **100019** | Hand grip strength | 20044 | Reason for skipping grip strength (left) | No | No |
| **100010** | Body size measures | 20045 | Reason for skipping waist | No | No |
| **100010** | Body size measures | 20046 | Reason for skipping hip measurement | No | No |
| **100010** | Body size measures | 20047 | Reason for skipping standing height | No | No |
| **100010** | Body size measures | 20048 | Reason for skipping sitting height | No | No |
| **100002** | Blood sample collection | 20049 | Blood sample #, note contents | No | No |
| **100002** | Blood sample collection | 20050 | Reason blood sampling not attempted | No | No |
| **100007** | Arterial stiffness | 20051 | Reason for skipping arterial stiffness | No | No |
| **100014** | Autorefraction | 20052 | Reason for skipping refractometry (left) | No | No |
| **100015** | Intraocular pressure | 20053 | Reason for skipping IOP (right) | No | No |
| **100015** | Intraocular pressure | 20054 | Reason for skipping IOP (left) | No | No |
| **100014** | Autorefraction | 20055 | Reason for skipping refractometry (right) | No | No |
| **100017** | Visual acuity | 20056 | Reason for skipping visual acuity (right) | No | No |
| **100017** | Visual acuity | 20057 | Reason for skipping visual acuity (left) | No | No |
| **100012** | ECG during exercise | 20058 | Reason for skipping ECG | No | No |
| **100012** | ECG during exercise | 20059 | Reason ECG not completed | No | No |
| **100012** | ECG during exercise | 20060 | Reason at-rest ECG performed without bicycle | No | No |
| **100016** | Retinal optical coherence tomography | 20061 | Reason for skipping OCT (right) | No | No |
| **100016** | Retinal optical coherence tomography | 20062 | Reason for skipping OCT (left) | No | No |
| **100096** | Saliva sample collection | 20071 | Reason no saliva sample collected | No | No |
| **100095** | Urine sample collection | 20072 | Reason no urine sample collected | No | No |
| **100024** | Reception | 20074 | Home location at assessment - east co-ordinate (rounded) | No | No |
| **100024** | Reception | 20075 | Home location at assessment - north co-ordinate (rounded) | No | No |
| **100075** | Medications | 20076 | Treatment/medication READ-code | No | No |
| **100114** | Diet questionnaire performance | 20077 | Number of diet questionnaires completed | No | No |
| **100114** | Diet questionnaire performance | 20078 | When diet questionnaire completion requested | No | No |
| **100114** | Diet questionnaire performance | 20079 | Day-of-week questionnaire completion requested | No | No |
| **100114** | Diet questionnaire performance | 20080 | Day-of-week questionnaire completed | No | No |
| **100114** | Diet questionnaire performance | 20081 | Hour-of-day questionnaire completed | No | No |
| **100114** | Diet questionnaire performance | 20082 | Duration of questionnaire | No | No |
| **100114** | Diet questionnaire performance | 20083 | Delay between questionnaire request and completion | No | No |
| **100112** | Vitamin/mineral supplements yesterday | 20084 | Vitamin and/or mineral supplement use | No | No |
| **100097** | Typical diet yesterday | 20085 | Reason for not eating or drinking normally | No | No |
| **100097** | Typical diet yesterday | 20086 | Type of special diet followed | No | No |
| **100101** | Bread/pasta/rice yesterday | 20087 | Types of spread used on bread/crackers | No | No |
| **100110** | Spreads/sauces/cooking oils yesterday | 20088 | Types of spreads/sauces consumed | No | No |
| **100105** | Meal type yesterday | 20089 | Type of meals eaten | No | No |
| **100110** | Spreads/sauces/cooking oils yesterday | 20090 | Type of fat/oil used in cooking | No | No |
| **100101** | Bread/pasta/rice yesterday | 20091 | Type of sliced bread eaten | No | No |
| **100101** | Bread/pasta/rice yesterday | 20092 | Type of baguette eaten | No | No |
| **100101** | Bread/pasta/rice yesterday | 20093 | Type of large bap eaten | No | No |
| **100101** | Bread/pasta/rice yesterday | 20094 | Type of bread roll eaten | No | No |
| **100100** | Alcoholic beverages yesterday | 20095 | Size of white wine glass drunk | No | No |
| **100100** | Alcoholic beverages yesterday | 20096 | Size of red wine glass drunk | No | No |
| **100100** | Alcoholic beverages yesterday | 20097 | Size of rose wine glass drunk | No | No |
| **100101** | Bread/pasta/rice yesterday | 20098 | Thickness of butter/margarine spread on sliced bread | No | No |
| **100101** | Bread/pasta/rice yesterday | 20099 | Thickness of butter/margarine spread on baguettes | No | No |
| **100101** | Bread/pasta/rice yesterday | 20100 | Thickness of butter/margarine spread on large baps | No | No |
| **100101** | Bread/pasta/rice yesterday | 20101 | Thickness of butter/margarine spread on bread rolls | No | No |
| **100101** | Bread/pasta/rice yesterday | 20102 | Thickness of butter/margarine spread on crackers/crispbreads | No | No |
| **100101** | Bread/pasta/rice yesterday | 20103 | Thickness of butter/margarine spread on oatcakes | No | No |
| **100101** | Bread/pasta/rice yesterday | 20104 | Thickness of butter/margarine spread on other bread | No | No |
| **100102** | Cereal yesterday | 20105 | Liquid used to make porridge | No | No |
| **100109** | Soup/snacks/pastries yesterday | 20106 | Type of yogurt eaten | No | No |
| **100034** | Family history | 20107 | Illnesses of father | No | No |
| **100109** | Soup/snacks/pastries yesterday | 20108 | Ingredients in canned soup | No | No |
| **100109** | Soup/snacks/pastries yesterday | 20109 | Ingredients in homemade soup | No | No |
| **100034** | Family history | 20110 | Illnesses of mother | No | No |
| **100034** | Family history | 20111 | Illnesses of siblings | No | No |
| **100034** | Family history | 20112 | Illnesses of adopted father | No | No |
| **100034** | Family history | 20113 | Illnesses of adopted mother | No | No |
| **100034** | Family history | 20114 | Illnesses of adopted siblings | No | No |
| **100072** | Early life factors | 20115 | Country of Birth (non-UK origin) | No | No |
| **100058** | Smoking | 20116 | Smoking status | Yes | Yes |
| **100051** | Alcohol | 20117 | Alcohol drinker status | Yes | Yes |
| **100024** | Reception | 20118 | Home area population density - urban or rural | No | No |
| **100064** | Employment | 20119 | Current employment status - corrected | No | No |
| **100073** | Employment | 20121 | Cascot confidence score | No | No |
| **100060** | Mental health | 20122 | Bipolar disorder status | No | No |
| **100060** | Mental health | 20123 | Single episode of probable major depression | No | No |
| **100060** | Mental health | 20124 | Probable recurrent major depression (moderate) | No | No |
| **100060** | Mental health | 20125 | Probable recurrent major depression (severe) | No | No |
| **100060** | Mental health | 20126 | Bipolar and major depression status | No | No |
| **100060** | Mental health | 20127 | Neuroticism score | Yes | Yes |
| **100027** | Fluid intelligence / reasoning | 20128 | Number of fluid intelligence questions attempted within time limit | No | No |
| **117** | Pairs matching | 20129 | Number of columns displayed in round | No | No |
| **117** | Pairs matching | 20130 | Number of rows displayed in round | No | No |
| **117** | Pairs matching | 20131 | Number of correct matches in round | No | No |
| **117** | Pairs matching | 20132 | Number of incorrect matches in round | No | No |
| **117** | Pairs matching | 20133 | Time to complete round | No | No |
| **117** | Pairs matching | 20134 | When pairs test completed | No | No |
| **118** | Fluid intelligence / reasoning | 20135 | When fluid intelligence test completed | No | No |
| **121** | Trail making | 20136 | When trail making test completed | No | No |
| **122** | Symbol digit substitution | 20137 | When symbol digit substitution test completed | No | No |
| **120** | Numeric memory | 20138 | When numeric memory test completed | No | No |
| **116** | Cognitive function online | 20140 | When device described | No | No |
| **121** | Trail making | 20147 | Errors before selecting correct item in numeric path (trail #1) | No | No |
| **121** | Trail making | 20148 | Errors before selecting correct item in alphanumeric path (trail #2) | No | No |
| **121** | Trail making | 20149 | Interval between previous point and current one in numeric path (trail #1) | No | No |
| **100020** | Spirometry | 20150 | Forced expiratory volume in 1-second (FEV1), Best measure | No | Yes |
| **100020** | Spirometry | 20151 | Forced vital capacity (FVC), Best measure | No | Yes |
| **100020** | Spirometry | 20152 | Reproduciblity of spirometry measurement using ERS/ATS criteria | No | No |
| **100020** | Spirometry | 20153 | Forced expiratory volume in 1-second (FEV1), predicted | No | Yes |
| **100020** | Spirometry | 20154 | Forced expiratory volume in 1-second (FEV1), predicted percentage | No | Yes |
| **121** | Trail making | 20155 | Interval between previous point and current one in alphanumeric path (trail #2) | No | No |
| **121** | Trail making | 20156 | Duration to complete numeric path (trail #1) | No | No |
| **121** | Trail making | 20157 | Duration to complete alphanumeric path (trail #2) | No | No |
| **103** | DXA assessment | 20158 | DXA images | No | No |
| **122** | Symbol digit substitution | 20159 | Number of symbol digit matches made correctly | No | No |
| **100058** | Smoking | 20160 | Ever smoked | No | No |
| **100058** | Smoking | 20161 | Pack years of smoking | No | No |
| **100058** | Smoking | 20162 | Pack years adult smoking as proportion of life span exposed to smoking | No | No |
| **118** | Fluid intelligence / reasoning | 20165 | FI1 : numeric addition test | No | No |
| **118** | Fluid intelligence / reasoning | 20167 | FI2 : identify largest number | No | No |
| **118** | Fluid intelligence / reasoning | 20169 | FI3 : word interpolation | No | No |
| **118** | Fluid intelligence / reasoning | 20171 | FI4 : positional arithmetic | No | No |
| **118** | Fluid intelligence / reasoning | 20173 | FI5 : family relationship calculation | No | No |
| **118** | Fluid intelligence / reasoning | 20175 | FI6 : conditional arithmetic | No | No |
| **118** | Fluid intelligence / reasoning | 20177 | FI7 : synonym | No | No |
| **118** | Fluid intelligence / reasoning | 20179 | FI8 : chained arithmetic | No | No |
| **118** | Fluid intelligence / reasoning | 20181 | FI9 : concept interpolation | No | No |
| **118** | Fluid intelligence / reasoning | 20183 | FI10 : arithmetic sequence recognition | No | No |
| **118** | Fluid intelligence / reasoning | 20185 | FI11 : antonym | No | No |
| **118** | Fluid intelligence / reasoning | 20187 | FI12 : square sequence recognition | No | No |
| **118** | Fluid intelligence / reasoning | 20189 | FI13 : subset inclusion logic | No | No |
| **118** | Fluid intelligence / reasoning | 20191 | Fluid intelligence score | No | No |
| **118** | Fluid intelligence / reasoning | 20192 | Number of fluid intelligence questions attempted within time limit | No | No |
| **118** | Fluid intelligence / reasoning | 20193 | FI14 : alphanumeric substitution | No | No |
| **122** | Symbol digit substitution | 20195 | Number of symbol digit matches attempted | No | No |
| **122** | Symbol digit substitution | 20196 | First code array presented | No | No |
| **506** | Paired associate learning | 20197 | Number of word pairs correctly associated | No | No |
| **122** | Symbol digit substitution | 20198 | Test array presented | No | No |
| **100075** | Medications | 20199 | Antibiotic codes for last 3 months | No | No |
| **122** | Symbol digit substitution | 20200 | Values wanted | No | No |
| **105** | Abdominal MRI | 20201 | Dixon technique for internal fat - DICOM | No | No |
| **131** | Pancreas MRI | 20202 | Pancreatic fat - DICOM | No | No |
| **126** | Liver MRI | 20203 | Liver imaging - gradient echo - DICOM | No | No |
| **126** | Liver MRI | 20204 | Liver Imaging - T1 ShMoLLI - DICOM | No | No |
| **104** | ECG at rest, 12-lead | 20205 | ECG datasets | No | No |
| **131** | Pancreas MRI | 20206 | Measurements of pancreas volume - DICOM | No | No |
| **102** | Heart MRI | 20207 | Scout images for heart MRI - DICOM | No | No |
| **102** | Heart MRI | 20208 | Long axis heart images - DICOM | No | No |
| **102** | Heart MRI | 20209 | Short axis heart images - DICOM | No | No |
| **102** | Heart MRI | 20210 | Aortic distensibilty images - DICOM | No | No |
| **102** | Heart MRI | 20211 | Cine tagging images - DICOM | No | No |
| **102** | Heart MRI | 20212 | Left ventricular outflow tract images - DICOM | No | No |
| **102** | Heart MRI | 20213 | Blood flow images - DICOM | No | No |
| **102** | Heart MRI | 20214 | Experimental shMOLLI sequence images - DICOM | No | No |
| **108** | Scout images and configuration for brain MRI | 20215 | Scout images for brain scans - DICOM | No | No |
| **110** | T1 structural brain MRI | 20216 | T1 structural brain images - DICOM | No | No |
| **106** | Task functional brain MRI | 20217 | Functional brain images - task - DICOM | No | No |
| **107** | Diffusion brain MRI | 20218 | Multiband diffusion brain images - DICOM | No | No |
| **109** | Susceptibility weighted brain MRI | 20219 | Susceptibility weighted brain images - DICOM | No | No |
| **112** | T2-weighted brain MRI | 20220 | T2 FLAIR structural brain images - DICOM | No | No |
| **112** | T2-weighted brain MRI | 20221 | T2/PD brain images - DICOM | No | No |
| **101** | Carotid ultrasound | 20222 | Carotid artery ultrasound image (left) | No | No |
| **101** | Carotid ultrasound | 20223 | Carotid artery ultrasound image (right) | No | No |
| **108** | Scout images and configuration for brain MRI | 20224 | Phoenix - DICOM | No | No |
| **111** | Resting functional brain MRI | 20225 | Functional brain images - resting - DICOM | No | No |
| **101** | Carotid ultrasound | 20226 | Carotid artery ultrasound report | No | No |
| **111** | Resting functional brain MRI | 20227 | Functional brain images - resting - NIFTI | No | No |
| **122** | Symbol digit substitution | 20229 | Values entered | No | No |
| **122** | Symbol digit substitution | 20230 | Duration to entering value | No | No |
| **120** | Numeric memory | 20240 | Maximum digits remembered correctly | No | No |
| **101** | Carotid ultrasound | 20241 | Raw carotid device data | No | No |
| **118** | Fluid intelligence / reasoning | 20242 | Fluid intelligence completion status | No | No |
| **156** |  | 20243 | Kidney Imaging - T1 ShMOLLI - DICOM | No | No |
| **117** | Pairs matching | 20244 | Pairs matching completion status | No | No |
| **122** | Symbol digit substitution | 20245 | Symbol digit completion status | No | No |
| **121** | Trail making | 20246 | Trail making completion status | No | No |
| **121** | Trail making | 20247 | Total errors traversing numeric path (trail #1) | No | No |
| **121** | Trail making | 20248 | Total errors traversing alphanumeric path (trail #2) | No | No |
| **106** | Task functional brain MRI | 20249 | Functional brain images - task - NIFTI | No | No |
| **107** | Diffusion brain MRI | 20250 | Multiband diffusion brain images - NIFTI | No | No |
| **109** | Susceptibility weighted brain MRI | 20251 | Susceptibility weighted brain images - NIFTI | No | No |
| **110** | T1 structural brain MRI | 20252 | T1 structural brain images - NIFTI | No | No |
| **112** | T2-weighted brain MRI | 20253 | T2 FLAIR structural brain images - NIFTI | No | No |
| **126** | Liver MRI | 20254 | Liver imaging - IDEAL protocol - DICOM | No | No |
| **100020** | Spirometry | 20255 | Spirometry QC measure | No | No |
| **100020** | Spirometry | 20256 | Forced expiratory volume in 1-second (FEV1) Z-score | Yes | Yes |
| **100020** | Spirometry | 20257 | Forced vital capacity (FVC) Z-score | Yes | Yes |
| **100020** | Spirometry | 20258 | FEV1/ FVC ratio Z-score | Yes | Yes |
| **131** | Pancreas MRI | 20259 | Pancreas Images - ShMoLLI - DICOM | No | No |
| **131** | Pancreas MRI | 20260 | Pancreas Images - gradient echo - DICOM | No | No |
| **100017** | Visual acuity | 20261 | avMSE | No | No |
| **100017** | Visual acuity | 20262 | Myopia diagnosis | No | No |
| **110** | T1 structural brain MRI | 20263 | T1 surface model files and additional structural segmentations | No | No |
| **156** |  | 20264 | Kidney Imaging - gradient echo - DICOM | No | No |
| **156** |  | 20265 | Kidney Imaging - T2 haste - DICOM | No | No |
| **119** |  | 20266 | Arterial spin labelling brain images - DICOM | No | No |
| **156** |  | 20267 | Kidney imaging - T2 Vibe - DICOM | No | No |
| **100024** | Reception | 20270 | Home location - census output area (2001 Census) | No | No |
| **100024** | Reception | 20271 | Home location - lower layer super output area (2001 Census) | No | No |
| **100024** | Reception | 20272 | Home location - local authority district (2001 boundaries) | No | No |
| **100024** | Reception | 20273 | Home location - census output area (2011 Census) | No | No |
| **100024** | Reception | 20274 | Home location - lower layer super output area (2011 Census) | No | No |
| **100024** | Reception | 20275 | Home location - middle layer super output area (2011 Census) | No | No |
| **100024** | Reception | 20276 | Home location - local authority district (2011 boundaries) | No | No |
| **100073** | Employment | 20277 | Job code at visit | Yes | Yes |
| **136** | Mental health | 20400 | Date of completing mental health questionnaire | No | No |
| **141** | Addictions | 20401 | Ever addicted to any substance or behaviour | No | No |
| **142** | Alcohol use | 20403 | Amount of alcohol drunk on a typical drinking day | Yes | Yes |
| **141** | Addictions | 20404 | Ever physically dependent on alcohol | No | No |
| **142** | Alcohol use | 20405 | Ever had known person concerned about, or recommend reduction of, alcohol consumption | No | No |
| **141** | Addictions | 20406 | Ever addicted to alcohol | No | No |
| **142** | Alcohol use | 20407 | Frequency of failure to fulfil normal expectations due to drinking alcohol in last year | No | No |
| **142** | Alcohol use | 20408 | Frequency of memory loss due to drinking alcohol in last year | No | No |
| **142** | Alcohol use | 20409 | Frequency of feeling guilt or remorse after drinking alcohol in last year | No | No |
| **142** | Alcohol use | 20410 | Age when known person last commented about drinking habits | No | No |
| **142** | Alcohol use | 20411 | Ever been injured or injured someone else through drinking alcohol | No | No |
| **142** | Alcohol use | 20412 | Frequency of needing morning drink of alcohol after heavy drinking session in last year | No | No |
| **142** | Alcohol use | 20413 | Frequency of inability to cease drinking in last year | No | No |
| **142** | Alcohol use | 20414 | Frequency of drinking alcohol | No | No |
| **141** | Addictions | 20415 | Ongoing addiction to alcohol | No | No |
| **142** | Alcohol use | 20416 | Frequency of consuming six or more units of alcohol | No | No |
| **140** | Anxiety | 20417 | Tense, sore, or aching muscles during worst period of anxiety | No | No |
| **140** | Anxiety | 20418 | Impact on normal roles during worst period of anxiety | No | No |
| **140** | Anxiety | 20419 | Difficulty concentrating during worst period of anxiety | No | No |
| **140** | Anxiety | 20420 | Longest period spent worried or anxious | No | No |
| **140** | Anxiety | 20421 | Ever felt worried, tense, or anxious for most of a month or longer | No | No |
| **140** | Anxiety | 20422 | More irritable than usual during worst period of anxiety | No | No |
| **140** | Anxiety | 20423 | Keyed up or on edge during worst period of anxiety | No | No |
| **140** | Anxiety | 20425 | Ever worried more than most people would in similar situation | No | No |
| **140** | Anxiety | 20426 | Restless during period of worst anxiety | No | No |
| **140** | Anxiety | 20427 | Frequent trouble falling or staying asleep during worst period of anxiety | No | No |
| **140** | Anxiety | 20428 | Professional informed about anxiety | No | No |
| **140** | Anxiety | 20429 | Easily tired during worst period of anxiety | No | No |
| **141** | Addictions | 20431 | Ever addicted to a behaviour or miscellanous | No | No |
| **141** | Addictions | 20432 | Ongoing behavioural or miscellanous addiction | No | No |
| **138** | Depression | 20433 | Age at first episode of depression | No | No |
| **138** | Depression | 20434 | Age at last episode of depression | No | No |
| **138** | Depression | 20435 | Difficulty concentrating during worst depression | No | No |
| **138** | Depression | 20436 | Fraction of day affected during worst episode of depression | No | No |
| **138** | Depression | 20437 | Thoughts of death during worst depression | No | No |
| **138** | Depression | 20438 | Duration of worst depression | No | No |
| **138** | Depression | 20439 | Frequency of depressed days during worst episode of depression | No | No |
| **138** | Depression | 20440 | Impact on normal roles during worst period of depression | No | No |
| **138** | Depression | 20441 | Ever had prolonged loss of interest in normal activities | No | No |
| **138** | Depression | 20442 | Lifetime number of depressed periods | No | No |
| **138** | Depression | 20445 | Depression possibly related to childbirth | No | No |
| **138** | Depression | 20446 | Ever had prolonged feelings of sadness or depression | No | No |
| **138** | Depression | 20447 | Depression possibly related to stressful or traumatic event | No | No |
| **138** | Depression | 20448 | Professional informed about depression | No | No |
| **138** | Depression | 20449 | Feelings of tiredness during worst episode of depression | No | No |
| **138** | Depression | 20450 | Feelings of worthlessness during worst period of depression | No | No |
| **143** | Cannabis use | 20453 | Ever taken cannabis | No | No |
| **143** | Cannabis use | 20454 | Maximum frequency of taking cannabis | No | No |
| **143** | Cannabis use | 20455 | Age when last took cannabis | No | No |
| **141** | Addictions | 20456 | Ever addicted to illicit or recreational drugs | No | No |
| **141** | Addictions | 20457 | Ongoing addiction or dependence on illicit or recreational drugs | No | No |
| **147** | Happiness and subjective well-being | 20458 | General happiness | No | No |
| **147** | Happiness and subjective well-being | 20459 | General happiness with own health | No | No |
| **147** | Happiness and subjective well-being | 20460 | Belief that own life is meaningful | No | No |
| **144** | Unusual and psychotic experiences | 20461 | Age when first had unusual or psychotic experience | No | No |
| **144** | Unusual and psychotic experiences | 20462 | Distress caused by unusual or psychotic experiences | No | No |
| **144** | Unusual and psychotic experiences | 20463 | Ever heard an un-real voice | No | No |
| **144** | Unusual and psychotic experiences | 20465 | Number of times heard an un-real voice | No | No |
| **144** | Unusual and psychotic experiences | 20466 | Ever prescribed a medication for unusual or psychotic experiences | No | No |
| **144** | Unusual and psychotic experiences | 20467 | Frequency of unusual or psychotic experiences in past year | No | No |
| **144** | Unusual and psychotic experiences | 20468 | Ever believed in an un-real conspiracy against self | No | No |
| **144** | Unusual and psychotic experiences | 20470 | Number of times believed in an un-real conspiracy against self | No | No |
| **144** | Unusual and psychotic experiences | 20471 | Ever seen an un-real vision | No | No |
| **144** | Unusual and psychotic experiences | 20473 | Number of times seen an un-real vision | No | No |
| **144** | Unusual and psychotic experiences | 20474 | Ever believed in un-real communications or signs | No | No |
| **144** | Unusual and psychotic experiences | 20476 | Number of times believed in un-real communications or signs | No | No |
| **144** | Unusual and psychotic experiences | 20477 | Ever talked to a health professional about unusual or psychotic experiences | No | No |
| **146** | Self-harm behaviours | 20479 | Ever thought that life not worth living | No | No |
| **146** | Self-harm behaviours | 20480 | Ever self-harmed | No | No |
| **146** | Self-harm behaviours | 20481 | Self-harmed in past year | No | No |
| **146** | Self-harm behaviours | 20482 | Number of times self-harmed | No | No |
| **146** | Self-harm behaviours | 20483 | Ever attempted suicide | No | No |
| **146** | Self-harm behaviours | 20484 | Attempted suicide in past year | No | No |
| **146** | Self-harm behaviours | 20485 | Ever contemplated self-harm | No | No |
| **146** | Self-harm behaviours | 20486 | Contemplated self-harm in past year | No | No |
| **145** | Traumatic events | 20487 | Felt hated by family member as a child | No | No |
| **145** | Traumatic events | 20488 | Physically abused by family as a child | No | No |
| **145** | Traumatic events | 20489 | Felt loved as a child | No | No |
| **145** | Traumatic events | 20490 | Sexually molested as a child | No | No |
| **145** | Traumatic events | 20491 | Someone to take to doctor when needed as a child | No | No |
| **139** | Mania | 20492 | Longest period of mania or irritability | No | No |
| **139** | Mania | 20493 | Severity of problems due to mania or irritability | No | No |
| **145** | Traumatic events | 20494 | Felt irritable or had angry outbursts in past month | No | No |
| **145** | Traumatic events | 20495 | Avoided activities or situations because of previous stressful experience in past month | No | No |
| **145** | Traumatic events | 20496 | Felt distant from other people in past month | No | No |
| **145** | Traumatic events | 20497 | Repeated disturbing thoughts of stressful experience in past month | No | No |
| **145** | Traumatic events | 20498 | Felt very upset when reminded of stressful experience in past month | No | No |
| **137** | Mental distress | 20499 | Ever sought or received professional help for mental distress | No | No |
| **137** | Mental distress | 20500 | Ever suffered mental distress preventing usual activities | No | No |
| **139** | Mania | 20501 | Ever had period of mania / excitability | No | No |
| **139** | Mania | 20502 | Ever had period extreme irritability | No | No |
| **141** | Addictions | 20503 | Ever addicted to prescription or over-the-counter medication | No | No |
| **141** | Addictions | 20504 | Ongoing addiction or dependence to over-the-counter medication | No | No |
| **140** | Anxiety | 20505 | Recent easy annoyance or irritability | No | No |
| **140** | Anxiety | 20506 | Recent feelings or nervousness or anxiety | No | No |
| **138** | Depression | 20507 | Recent feelings of inadequacy | No | No |
| **138** | Depression | 20508 | Recent trouble concentrating on things | No | No |
| **140** | Anxiety | 20509 | Recent inability to stop or control worrying | No | No |
| **138** | Depression | 20510 | Recent feelings of depression | No | No |
| **138** | Depression | 20511 | Recent poor appetite or overeating | No | No |
| **140** | Anxiety | 20512 | Recent feelings of foreboding | No | No |
| **138** | Depression | 20513 | Recent thoughts of suicide or self-harm | No | No |
| **138** | Depression | 20514 | Recent lack of interest or pleasure in doing things | No | No |
| **140** | Anxiety | 20515 | Recent trouble relaxing | No | No |
| **140** | Anxiety | 20516 | Recent restlessness | No | No |
| **138** | Depression | 20517 | Trouble falling or staying asleep, or sleeping too much | No | No |
| **138** | Depression | 20518 | Recent changes in speed/amount of moving or speaking | No | No |
| **138** | Depression | 20519 | Recent feelings of tiredness or low energy | No | No |
| **140** | Anxiety | 20520 | Recent worrying too much about different things | No | No |
| **145** | Traumatic events | 20521 | Belittlement by partner or ex-partner as an adult | No | No |
| **145** | Traumatic events | 20522 | Been in a confiding relationship as an adult | No | No |
| **145** | Traumatic events | 20523 | Physical violence by partner or ex-partner as an adult | No | No |
| **145** | Traumatic events | 20524 | Sexual interference by partner or ex-partner without consent as an adult | No | No |
| **145** | Traumatic events | 20525 | Able to pay rent/mortgage as an adult | No | No |
| **145** | Traumatic events | 20526 | Been in serious accident believed to be life-threatening | No | No |
| **145** | Traumatic events | 20527 | Been involved in combat or exposed to war-zone | No | No |
| **145** | Traumatic events | 20528 | Diagnosed with life-threatening illness | No | No |
| **145** | Traumatic events | 20529 | Victim of physically violent crime | No | No |
| **145** | Traumatic events | 20530 | Witnessed sudden violent death | No | No |
| **145** | Traumatic events | 20531 | Victim of sexual assault | No | No |
| **138** | Depression | 20532 | Did your sleep change? | No | No |
| **138** | Depression | 20533 | Trouble falling asleep | No | No |
| **138** | Depression | 20534 | Sleeping too much | No | No |
| **138** | Depression | 20535 | Waking too early | No | No |
| **138** | Depression | 20536 | Weight change during worst episode of depression | No | No |
| **140** | Anxiety | 20537 | Frequency of difficulty controlling worry during worst period of anxiety | No | No |
| **140** | Anxiety | 20538 | Worried most days during period of worst anxiety | No | No |
| **140** | Anxiety | 20539 | Frequency of inability to stop worrying during worst period of anxiety | No | No |
| **140** | Anxiety | 20540 | Multiple worries during worst period of anxiety | No | No |
| **140** | Anxiety | 20541 | Difficulty stopping worrying during worst period of anxiety | No | No |
| **140** | Anxiety | 20542 | Stronger worrying (than other people) during period of worst anxiety | No | No |
| **140** | Anxiety | 20543 | Number of things worried about during worst period of anxiety | No | No |
| **137** | Mental distress | 20544 | Mental health problems ever diagnosed by a professional | Yes | Yes |
| **138** | Depression | 20546 | Substances taken for depression | No | No |
| **138** | Depression | 20547 | Activities undertaken to treat depression | No | No |
| **139** | Mania | 20548 | Manifestations of mania or irritability | No | No |
| **140** | Anxiety | 20549 | Substances taken for anxiety | No | No |
| **140** | Anxiety | 20550 | Activities undertaken to treat anxiety | No | No |
| **141** | Addictions | 20551 | Substance of prescription or over-the-counter medication addiction | No | No |
| **141** | Addictions | 20552 | Behavioural and miscellaneous addictions | No | No |
| **146** | Self-harm behaviours | 20553 | Methods of self-harm used | No | No |
| **146** | Self-harm behaviours | 20554 | Actions taken following self-harm | No | No |
| **1039** | Food (and other) preferences | 20599 | Order of asking questions | No | No |
| **1039** | Food (and other) preferences | 20600 | Liking for adding salt to foods | No | No |
| **1039** | Food (and other) preferences | 20601 | Liking for aniseed | No | No |
| **1039** | Food (and other) preferences | 20602 | Liking for apple juice | No | No |
| **1039** | Food (and other) preferences | 20603 | Liking for apples | No | No |
| **1039** | Food (and other) preferences | 20604 | Liking for asparagus | No | No |
| **1039** | Food (and other) preferences | 20605 | Liking for aubergine | No | No |
| **1039** | Food (and other) preferences | 20606 | Liking for avocados | No | No |
| **1039** | Food (and other) preferences | 20607 | Liking for bacon | No | No |
| **1039** | Food (and other) preferences | 20608 | Liking for baked/steamed fish | No | No |
| **1039** | Food (and other) preferences | 20609 | Liking for bananas | No | No |
| **1039** | Food (and other) preferences | 20610 | Liking for barbequed or grilled meat | No | No |
| **1039** | Food (and other) preferences | 20611 | Liking for beef steak | No | No |
| **1039** | Food (and other) preferences | 20612 | Liking for beetroot | No | No |
| **1039** | Food (and other) preferences | 20613 | Liking for bell pepper | No | No |
| **1039** | Food (and other) preferences | 20614 | Liking for bicycling | No | No |
| **1039** | Food (and other) preferences | 20615 | Liking for biscuits | No | No |
| **1039** | Food (and other) preferences | 20616 | Liking for bitter foods | No | No |
| **1039** | Food (and other) preferences | 20617 | Liking for bitter/ale | No | No |
| **1039** | Food (and other) preferences | 20618 | Liking for black olives | No | No |
| **1039** | Food (and other) preferences | 20619 | Liking for black pepper | No | No |
| **1039** | Food (and other) preferences | 20620 | Liking for blue cheese | No | No |
| **1039** | Food (and other) preferences | 20621 | Liking for bolognese sauce | No | No |
| **1039** | Food (and other) preferences | 20622 | Liking for broad beans | No | No |
| **1039** | Food (and other) preferences | 20623 | Liking for broccoli | No | No |
| **1039** | Food (and other) preferences | 20624 | Liking for brown rice | No | No |
| **1039** | Food (and other) preferences | 20625 | Liking for brussel sprouts | No | No |
| **1039** | Food (and other) preferences | 20626 | Liking for burgers (meat) | No | No |
| **1039** | Food (and other) preferences | 20627 | Liking for burn of spicy foods | No | No |
| **1039** | Food (and other) preferences | 20628 | Liking for butter on bread | No | No |
| **1039** | Food (and other) preferences | 20629 | Liking for butternut squash | No | No |
| **1039** | Food (and other) preferences | 20630 | Liking for cabbage | No | No |
| **1039** | Food (and other) preferences | 20631 | Liking for cake | No | No |
| **1039** | Food (and other) preferences | 20632 | Liking for cake icing | No | No |
| **1039** | Food (and other) preferences | 20633 | Liking for capers | No | No |
| **1039** | Food (and other) preferences | 20634 | Liking for cauliflower | No | No |
| **1039** | Food (and other) preferences | 20635 | Liking for cereal/granola bar | No | No |
| **1039** | Food (and other) preferences | 20636 | Liking for cheesecake | No | No |
| **1039** | Food (and other) preferences | 20637 | Liking for cherries | No | No |
| **1039** | Food (and other) preferences | 20638 | Liking for chicken | No | No |
| **1039** | Food (and other) preferences | 20639 | Liking for chilli pepper | No | No |
| **1039** | Food (and other) preferences | 20640 | Liking for chips/french fries | No | No |
| **1039** | Food (and other) preferences | 20641 | Liking for cigarette smoking | No | No |
| **1039** | Food (and other) preferences | 20642 | Liking for cod | No | No |
| **1039** | Food (and other) preferences | 20643 | Liking for coffee with sugar | No | No |
| **1039** | Food (and other) preferences | 20644 | Liking for coffee without sugar | No | No |
| **1039** | Food (and other) preferences | 20645 | Liking for coriander | No | No |
| **1039** | Food (and other) preferences | 20646 | Liking for corn flakes | No | No |
| **1039** | Food (and other) preferences | 20647 | Liking for cream | No | No |
| **1039** | Food (and other) preferences | 20648 | Liking for croissant | No | No |
| **1039** | Food (and other) preferences | 20649 | Liking for cucumber | No | No |
| **1039** | Food (and other) preferences | 20650 | Liking for curry | No | No |
| **1039** | Food (and other) preferences | 20651 | Liking for dairy products | No | No |
| **1039** | Food (and other) preferences | 20652 | Liking for dark chocolate | No | No |
| **1039** | Food (and other) preferences | 20653 | Liking for diet fizzy drinks | No | No |
| **1039** | Food (and other) preferences | 20654 | Liking for dried fruit | No | No |
| **1039** | Food (and other) preferences | 20655 | Liking for eggs | No | No |
| **1039** | Food (and other) preferences | 20656 | Liking for exercising alone | No | No |
| **1039** | Food (and other) preferences | 20657 | Liking for exercising with others | No | No |
| **1039** | Food (and other) preferences | 20658 | Liking for extra virgin olive oil | No | No |
| **1039** | Food (and other) preferences | 20659 | Liking for fatty foods | No | No |
| **1039** | Food (and other) preferences | 20660 | Liking for fresh tomatoes | No | No |
| **1039** | Food (and other) preferences | 20661 | Liking for fried chicken | No | No |
| **1039** | Food (and other) preferences | 20662 | Liking for fried/battered fish | No | No |
| **1039** | Food (and other) preferences | 20663 | Liking for fruit | No | No |
| **1039** | Food (and other) preferences | 20664 | Liking for garlic | No | No |
| **1039** | Food (and other) preferences | 20665 | Liking for gherkins | No | No |
| **1039** | Food (and other) preferences | 20666 | Liking for globe artichoke | No | No |
| **1039** | Food (and other) preferences | 20667 | Liking for goat's cheese | No | No |
| **1039** | Food (and other) preferences | 20668 | Liking for going to a cafe | No | No |
| **1039** | Food (and other) preferences | 20669 | Liking for going to the gym | No | No |
| **1039** | Food (and other) preferences | 20670 | Liking for going to the pub | No | No |
| **1039** | Food (and other) preferences | 20671 | Liking for grapefruit | No | No |
| **1039** | Food (and other) preferences | 20672 | Liking for green olives | No | No |
| **1039** | Food (and other) preferences | 20673 | Liking for haddock | No | No |
| **1039** | Food (and other) preferences | 20674 | Liking for ham | No | No |
| **1039** | Food (and other) preferences | 20675 | Liking for hard cheese | No | No |
| **1039** | Food (and other) preferences | 20676 | Liking for herring | No | No |
| **1039** | Food (and other) preferences | 20677 | Liking for honey | No | No |
| **1039** | Food (and other) preferences | 20678 | Liking for horseradish/wasabi | No | No |
| **1039** | Food (and other) preferences | 20679 | Liking for ice cream | No | No |
| **1039** | Food (and other) preferences | 20680 | Liking for jam | No | No |
| **1039** | Food (and other) preferences | 20681 | Liking for kiwi fruit | No | No |
| **1039** | Food (and other) preferences | 20682 | Liking for lager | No | No |
| **1039** | Food (and other) preferences | 20683 | Liking for lamb | No | No |
| **1039** | Food (and other) preferences | 20684 | Liking for lemons | No | No |
| **1039** | Food (and other) preferences | 20685 | Liking for lentils/beans | No | No |
| **1039** | Food (and other) preferences | 20686 | Liking for liver | No | No |
| **1039** | Food (and other) preferences | 20687 | Liking for mackerel | No | No |
| **1039** | Food (and other) preferences | 20688 | Liking for marzipan | No | No |
| **1039** | Food (and other) preferences | 20689 | Liking for mayonnaise | No | No |
| **1039** | Food (and other) preferences | 20690 | Liking for melon | No | No |
| **1039** | Food (and other) preferences | 20691 | Liking for milk chocolate | No | No |
| **1039** | Food (and other) preferences | 20692 | Liking for mushrooms | No | No |
| **1039** | Food (and other) preferences | 20693 | Liking for onions | No | No |
| **1039** | Food (and other) preferences | 20694 | Liking for orange juice | No | No |
| **1039** | Food (and other) preferences | 20695 | Liking for oranges | No | No |
| **1039** | Food (and other) preferences | 20696 | Liking for pasta | No | No |
| **1039** | Food (and other) preferences | 20697 | Liking for pears | No | No |
| **1039** | Food (and other) preferences | 20698 | Liking for pizza | No | No |
| **1039** | Food (and other) preferences | 20699 | Liking for plain yogurt | No | No |
| **1039** | Food (and other) preferences | 20700 | Liking for plums | No | No |
| **1039** | Food (and other) preferences | 20701 | Liking for pollock | No | No |
| **1039** | Food (and other) preferences | 20702 | Liking for pork chop | No | No |
| **1039** | Food (and other) preferences | 20703 | Liking for porridge | No | No |
| **1039** | Food (and other) preferences | 20704 | Liking for potato crisps | No | No |
| **1039** | Food (and other) preferences | 20705 | Liking for potatoes | No | No |
| **1039** | Food (and other) preferences | 20706 | Liking for prawns | No | No |
| **1039** | Food (and other) preferences | 20707 | Liking for raw carrots | No | No |
| **1039** | Food (and other) preferences | 20708 | Liking for red meat | No | No |
| **1039** | Food (and other) preferences | 20709 | Liking for red wine | No | No |
| **1039** | Food (and other) preferences | 20710 | Liking for regular (non-diet) fizzy drinks | No | No |
| **1039** | Food (and other) preferences | 20711 | Liking for roast chicken | No | No |
| **1039** | Food (and other) preferences | 20712 | Liking for salad dressing | No | No |
| **1039** | Food (and other) preferences | 20713 | Liking for salad leaves | No | No |
| **1039** | Food (and other) preferences | 20714 | Liking for salami | No | No |
| **1039** | Food (and other) preferences | 20715 | Liking for salmon | No | No |
| **1039** | Food (and other) preferences | 20716 | Liking for salty foods | No | No |
| **1039** | Food (and other) preferences | 20717 | Liking for salty pretzels | No | No |
| **1039** | Food (and other) preferences | 20718 | Liking for sardines | No | No |
| **1039** | Food (and other) preferences | 20719 | Liking for sausages (meat) | No | No |
| **1039** | Food (and other) preferences | 20720 | Liking for savoury biscuits | No | No |
| **1039** | Food (and other) preferences | 20721 | Liking for shellfish (other than prawns) | No | No |
| **1039** | Food (and other) preferences | 20722 | Liking for skimmed milk | No | No |
| **1039** | Food (and other) preferences | 20723 | Liking for smoked fish | No | No |
| **1039** | Food (and other) preferences | 20724 | Liking for soft cheese | No | No |
| **1039** | Food (and other) preferences | 20725 | Liking for soy sauce | No | No |
| **1039** | Food (and other) preferences | 20726 | Liking for soya milk | No | No |
| **1039** | Food (and other) preferences | 20727 | Liking for spicy foods | No | No |
| **1039** | Food (and other) preferences | 20728 | Liking for spinach | No | No |
| **1039** | Food (and other) preferences | 20729 | Liking for spirits | No | No |
| **1039** | Food (and other) preferences | 20730 | Liking for strawberries | No | No |
| **1039** | Food (and other) preferences | 20731 | Liking for sweet coffee house drinks | No | No |
| **1039** | Food (and other) preferences | 20732 | Liking for sweet foods | No | No |
| **1039** | Food (and other) preferences | 20733 | Liking for taking the stairs | No | No |
| **1039** | Food (and other) preferences | 20734 | Liking for tea with sugar | No | No |
| **1039** | Food (and other) preferences | 20735 | Liking for tea without sugar | No | No |
| **1039** | Food (and other) preferences | 20736 | Liking for tomato ketchup | No | No |
| **1039** | Food (and other) preferences | 20737 | Liking for tinned tuna | No | No |
| **1039** | Food (and other) preferences | 20738 | Liking for turnip (white) | No | No |
| **1039** | Food (and other) preferences | 20739 | Liking for vegetables | No | No |
| **1039** | Food (and other) preferences | 20740 | Liking for vinegar | No | No |
| **1039** | Food (and other) preferences | 20741 | Liking for watching television | No | No |
| **1039** | Food (and other) preferences | 20742 | Liking for whisky | No | No |
| **1039** | Food (and other) preferences | 20743 | Liking for white bread | No | No |
| **1039** | Food (and other) preferences | 20744 | Liking for white rice | No | No |
| **1039** | Food (and other) preferences | 20745 | Liking for white wine | No | No |
| **1039** | Food (and other) preferences | 20746 | Liking for whole grain breakfast cereal | No | No |
| **1039** | Food (and other) preferences | 20747 | Liking for whole milk | No | No |
| **1039** | Food (and other) preferences | 20748 | Liking for wholemeal bread | No | No |
| **1039** | Food (and other) preferences | 20749 | Liking for working up a sweat | No | No |
| **1039** | Food (and other) preferences | 20750 | When food preferences questionnaire completed | No | No |
| **1039** | Food (and other) preferences | 20751 | Duration of questionnaire | No | No |
| **100065** | Ethnicity | 21000 | Ethnic background | No | Yes |
| **100010** | Body size measures | 21001 | Body mass index (BMI) | Yes | Yes |
| **100010** | Body size measures | 21002 | Weight | Yes | Yes |
| **100024** | Reception | 21003 | Age when attended assessment centre | No | No |
| **503** | Tower rearranging | 21004 | Number of puzzles correct | No | No |
| **100016** | Retinal optical coherence tomography | 21011 | FDA data file (left) | No | No |
| **100016** | Retinal optical coherence tomography | 21012 | FDS data file (left) | No | No |
| **100016** | Retinal optical coherence tomography | 21013 | FDA data file (right) | No | No |
| **100016** | Retinal optical coherence tomography | 21014 | FDS data file (right) | No | No |
| **100016** | Retinal optical coherence tomography | 21015 | Fundus retinal eye image (left) | No | No |
| **100016** | Retinal optical coherence tomography | 21016 | Fundus retinal eye image (right) | No | No |
| **100016** | Retinal optical coherence tomography | 21017 | OCT image slices (left) | No | No |
| **100016** | Retinal optical coherence tomography | 21018 | OCT image slices (right) | No | No |
| **100007** | Arterial stiffness | 21021 | Pulse wave Arterial Stiffness index | No | No |
| **100094** | Baseline characteristics | 21022 | Age at recruitment | No | No |
| **153** | Digestive health | 21023 | When digestive health questionnaire completed | No | No |
| **153** | Digestive health | 21024 | Ever diagnosed with IBS | Yes | Yes |
| **153** | Digestive health | 21025 | Frequency of discomfort/pain in abdomen in last 3 months | No | No |
| **153** | Digestive health | 21026 | Discomfort/pain occurring only during menstrual bleed | No | No |
| **153** | Digestive health | 21027 | Abdominal discomfort/pain for 6 months or longer | No | No |
| **153** | Digestive health | 21028 | Frequency of discomfort/pain getting better or stopping after a bowel movement | No | No |
| **153** | Digestive health | 21029 | More frequent bowel movements when abdominal discomfort/pain started | No | No |
| **153** | Digestive health | 21030 | Less frequent bowel movements when abdominal discomfort/pain started | No | No |
| **153** | Digestive health | 21031 | Stools looser when abdominal discomfort/pain started | No | No |
| **153** | Digestive health | 21032 | Frequency of harder stools when abdominal discomfort/pain started | No | No |
| **153** | Digestive health | 21033 | Frequency of hard/lumpy stools in the last 3 months | No | No |
| **153** | Digestive health | 21034 | Frequency of loose/mushy/watery stools in the last 3 months | No | No |
| **153** | Digestive health | 21035 | Currently (in last 3 months) suffer from abdominal pain | No | No |
| **153** | Digestive health | 21036 | Severity of current abdominal pain | No | No |
| **153** | Digestive health | 21037 | Number of days (out of 10) with abdominal pain | No | No |
| **153** | Digestive health | 21038 | Currently suffer from abdominal distension | No | No |
| **153** | Digestive health | 21039 | Severity of current abdominal distension/tightness | No | No |
| **153** | Digestive health | 21040 | Satisfaction with bowel habits | No | No |
| **153** | Digestive health | 21041 | Degree to which abdominal pain/discomfort/altered bowel habits affect/interfere with life in general | No | No |
| **153** | Digestive health | 21042 | Greatest number of times bowels opened per day | No | No |
| **153** | Digestive health | 21043 | Least number of times bowels opened per week | No | No |
| **153** | Digestive health | 21044 | Average number of times bowels opened per day | No | No |
| **153** | Digestive health | 21045 | Number of weeks absent from work due to IBS, in the last year | No | No |
| **153** | Digestive health | 21046 | When gave up work due to IBS | No | No |
| **153** | Digestive health | 21047 | Number of weeks (in the last year) at work suffering because of IBS | No | No |
| **153** | Digestive health | 21048 | Degree bothered by back pain in the past 3 months | No | No |
| **153** | Digestive health | 21049 | Degree bothered by pain in arms/legs/joints in the past 3 months | No | No |
| **153** | Digestive health | 21050 | Degree bothered by menstrual cramps or other problems with period in the last 3 months | No | No |
| **153** | Digestive health | 21051 | Degree bothered by headaches in the last 3 months | No | No |
| **153** | Digestive health | 21052 | Degree bothered by chest pain in the last 3 months | No | No |
| **153** | Digestive health | 21053 | Degree bothered by dizziness in the last 3 months | No | No |
| **153** | Digestive health | 21054 | Degree bothered by fainting spells in the last 3 months | No | No |
| **153** | Digestive health | 21055 | Degree bothered by feeling heart pound/race in the last 3 months | No | No |
| **153** | Digestive health | 21056 | Degree bothered by shortness of breath in the last 3 months | No | No |
| **153** | Digestive health | 21057 | Degree bothered by pain/problems during intercourse in the last 3 months | No | No |
| **153** | Digestive health | 21058 | Degree bothered by urinary frequency/bladder irritability in the last 3 months | No | No |
| **153** | Digestive health | 21059 | Degree bothered by nausea in the last 3 months | No | No |
| **153** | Digestive health | 21060 | Degree bothered by feeling tired all the time in the last 3 months | No | No |
| **153** | Digestive health | 21061 | Degree bothered by trouble sleeping in the last 3 months | No | No |
| **153** | Digestive health | 21062 | Ever been offered/sought treatment for anxiety | No | No |
| **153** | Digestive health | 21063 | Ever been offered/sought treatment for depression | No | No |
| **153** | Digestive health | 21064 | Sensitive stomach | No | No |
| **153** | Digestive health | 21065 | Family history of IBS | No | No |
| **153** | Digestive health | 21066 | Born by caesarian section | No | No |
| **153** | Digestive health | 21067 | Long-term/recurrent antibiotics as child or teenager | No | No |
| **153** | Digestive health | 21068 | Diagnosed with coeliac disease or gluten sensitivity | No | No |
| **153** | Digestive health | 21069 | Method of coeliac disease/gluten sensitivity diagnosis | No | No |
| **153** | Digestive health | 21070 | Speed of onset of IBS symptoms | No | No |
| **153** | Digestive health | 21071 | Infectious illness diagnosis status when IBS symptoms first began | No | No |
| **153** | Digestive health | 21072 | Infection diagnosed alongside IBS when IBS symptoms began | No | No |
| **153** | Digestive health | 21073 | Fever when IBS symptoms first began | No | No |
| **153** | Digestive health | 21074 | Diarrhoea when IBS symptoms first began | No | No |
| **153** | Digestive health | 21075 | Bloody diarrhoea when IBS symptoms first began | No | No |
| **153** | Digestive health | 21076 | Vomiting when IBS symptoms first began | No | No |
| **152** | Process durations | 21611 | Reception duration | No | No |
| **152** | Process durations | 21621 | Consent duration | No | No |
| **152** | Process durations | 21622 | Touchscreen duration | No | No |
| **152** | Process durations | 21623 | Touchscreen cognitive duration (pilot) | No | No |
| **152** | Process durations | 21625 | Touchscreen cognitive duration | No | No |
| **152** | Process durations | 21631 | Verbal interview duration | No | No |
| **152** | Process durations | 21632 | Pilot Spirometry duration | No | No |
| **152** | Process durations | 21633 | Measurement/Impedance duration (pilot) | No | No |
| **152** | Process durations | 21634 | Biometrics duration | No | No |
| **152** | Process durations | 21636 | Eye measures duration | No | No |
| **152** | Process durations | 21638 | ECG during exercise duration | No | No |
| **152** | Process durations | 21641 | Urine collection duration | No | No |
| **152** | Process durations | 21642 | Sample collection duration | No | No |
| **152** | Process durations | 21651 | Conclusion duration | No | No |
| **152** | Process durations | 21661 | Imaging screening duration | No | No |
| **152** | Process durations | 21662 | Brain MRI duration | No | No |
| **152** | Process durations | 21663 | Chest MRI duration | No | No |
| **152** | Process durations | 21664 | DXA assessment duration | No | No |
| **152** | Process durations | 21665 | Carotid ultrasound duration | No | No |
| **152** | Process durations | 21666 | ECG at rest duration | No | No |
| **152** | Process durations | 21671 | Cardiac monitor duration | No | No |
| **127** | Process authorisation | 21711 | Reception authorisation | Yes | Yes |
| **127** | Process authorisation | 21721 | Consent authorisation | No | No |
| **127** | Process authorisation | 21722 | Touchscreen authorisation | Yes | Yes |
| **127** | Process authorisation | 21723 | Touchscreen cognitive authorisation (pilot) | Yes | Yes |
| **127** | Process authorisation | 21725 | Touchscreen cognitive authorisation | Yes | Yes |
| **127** | Process authorisation | 21731 | Verbal interview authorisation | Yes | Yes |
| **127** | Process authorisation | 21732 | Pilot Spirometry authorisation | Yes | Yes |
| **127** | Process authorisation | 21733 | Measurement/Impedance authorisation (pilot) | Yes | Yes |
| **127** | Process authorisation | 21734 | Biometrics authorisation | No | No |
| **127** | Process authorisation | 21736 | Eye measures authorisation | No | No |
| **127** | Process authorisation | 21738 | ECG during exercise authorisation | No | No |
| **127** | Process authorisation | 21741 | Urine collection authorisation | Yes | Yes |
| **127** | Process authorisation | 21742 | Sample collection authorisation | Yes | Yes |
| **127** | Process authorisation | 21751 | Conclusion authorisation | No | No |
| **127** | Process authorisation | 21761 | Imaging screening authorisation | Yes | Yes |
| **127** | Process authorisation | 21762 | Brain MRI authorisation | No | No |
| **127** | Process authorisation | 21763 | Chest MRI authorisation | No | No |
| **127** | Process authorisation | 21764 | DXA assessment authorisation | No | No |
| **127** | Process authorisation | 21765 | Carotid ultrasound authorisation | No | No |
| **127** | Process authorisation | 21766 | ECG at rest authorisation | No | No |
| **127** | Process authorisation | 21771 | Cardiac monitor authorisation | No | No |
| **129** | Process completion times | 21811 | Reception sign-off timestamp | No | No |
| **129** | Process completion times | 21821 | Consent sign-off timestamp | No | No |
| **129** | Process completion times | 21822 | Touchscreen sign-off timestamp | No | No |
| **129** | Process completion times | 21823 | Touchscreen cognitive sign-off timestamp (pilot) | No | No |
| **129** | Process completion times | 21825 | Touchscreen cognitive sign-off timestamp | No | No |
| **129** | Process completion times | 21831 | Verbal nterview sign-off timestamp | No | No |
| **129** | Process completion times | 21832 | Pilot Spirometry sign-off timestamp | No | No |
| **129** | Process completion times | 21833 | Measurement/Impedance sign-off timestamp (pilot) | No | No |
| **129** | Process completion times | 21834 | Biometrics sign-off timestamp | No | No |
| **129** | Process completion times | 21836 | Eye measures sign-off timestamp | No | No |
| **129** | Process completion times | 21838 | ECG during exercise sign-off timestamp | No | No |
| **129** | Process completion times | 21841 | Urine collection sign-off timestamp | No | No |
| **129** | Process completion times | 21842 | Sample collection sign-off timestamp | No | No |
| **129** | Process completion times | 21851 | Conclusion sign-off timestamp | No | No |
| **129** | Process completion times | 21861 | Imaging screening sign-off timestamp | No | No |
| **129** | Process completion times | 21862 | Brain MRI sign-off timestamp | No | No |
| **129** | Process completion times | 21863 | Chest MRI sign-off timestamp | No | No |
| **129** | Process completion times | 21864 | DXA assessment sign-off timestamp | No | No |
| **129** | Process completion times | 21865 | Carotid ultrasound sign-off timestamp | No | No |
| **129** | Process completion times | 21866 | ECG at rest sign-off timestamp | No | No |
| **129** | Process completion times | 21871 | Cardiac monitor sign-off timestamp | No | No |
| **100313** | Genotyping process and sample QC | 22000 | Genotype measurement batch | No | No |
| **100313** | Genotyping process and sample QC | 22001 | Genetic sex | No | No |
| **100313** | Genotyping process and sample QC | 22002 | CEL files | No | No |
| **100313** | Genotyping process and sample QC | 22003 | Heterozygosity | No | No |
| **100313** | Genotyping process and sample QC | 22004 | Heterozygosity, PCA corrected | No | No |
| **100313** | Genotyping process and sample QC | 22005 | Missingness | No | No |
| **100313** | Genotyping process and sample QC | 22006 | Genetic ethnic grouping | No | No |
| **100313** | Genotyping process and sample QC | 22007 | Genotype measurement plate | No | No |
| **100313** | Genotyping process and sample QC | 22008 | Genotype measurement well | No | No |
| **100313** | Genotyping process and sample QC | 22009 | Genetic principal components | No | No |
| **199001** | Interim genotype release | 22010 | Recommended genomic analysis exclusions | No | No |
| **199001** | Interim genotype release | 22011 | Genetic relatedness pairing | No | No |
| **199001** | Interim genotype release | 22012 | Genetic relatedness factor | No | No |
| **199001** | Interim genotype release | 22013 | Genetic relatedness IBS0 | No | No |
| **123** | Work environment | 22016 | Invitation to complete online occupational questionnaire, date sent | No | No |
| **116** | Cognitive function online | 22017 | Invitation to complete online cognitive function questionnaire, date sent | No | No |
| **199001** | Interim genotype release | 22018 | Genetic relatedness exclusions | No | No |
| **100313** | Genotyping process and sample QC | 22019 | Sex chromosome aneuploidy | No | No |
| **100313** | Genotyping process and sample QC | 22020 | Used in genetic principal components | No | No |
| **100313** | Genotyping process and sample QC | 22021 | Genetic kinship to other participants | No | No |
| **100313** | Genotyping process and sample QC | 22022 | Sex inference X probe-intensity | No | No |
| **100313** | Genotyping process and sample QC | 22023 | Sex inference Y probe-intensity | No | No |
| **100313** | Genotyping process and sample QC | 22024 | DNA concentration | No | No |
| **100313** | Genotyping process and sample QC | 22025 | Affymetrix quality control metric "Cluster.CR" | No | No |
| **100313** | Genotyping process and sample QC | 22026 | Affymetrix quality control metric "dQC" | No | No |
| **100313** | Genotyping process and sample QC | 22027 | Outliers for heterozygosity or missing rate | No | No |
| **100313** | Genotyping process and sample QC | 22028 | Use in phasing Chromosomes 1-22 | No | No |
| **100313** | Genotyping process and sample QC | 22029 | Use in phasing Chromosome X | No | No |
| **100313** | Genotyping process and sample QC | 22030 | Use in phasing Chromosome XY | No | No |
| **54** | MET Scores | 22032 | IPAQ activity group | No | No |
| **54** | MET Scores | 22033 | Summed days activity | No | No |
| **54** | MET Scores | 22034 | Summed minutes activity | No | No |
| **54** | MET Scores | 22035 | Above moderate/vigorous recommendation | No | No |
| **54** | MET Scores | 22036 | Above moderate/vigorous/walking recommendation | No | No |
| **54** | MET Scores | 22037 | MET minutes per week for walking | No | No |
| **54** | MET Scores | 22038 | MET minutes per week for moderate activity | No | No |
| **54** | MET Scores | 22039 | MET minutes per week for vigorous activity | No | No |
| **54** | MET Scores | 22040 | Summed MET minutes per week for all activity | No | No |
| **199001** | Interim genotype release | 22051 | UKBiLEVE genotype quality control for samples | No | No |
| **199001** | Interim genotype release | 22052 | UKBiLEVE unrelatedness indicator | No | No |
| **132** | Medical information | 22126 | Doctor diagnosed hayfever or allergic rhinitis | Yes | Yes |
| **132** | Medical information | 22127 | Doctor diagnosed asthma | Yes | Yes |
| **132** | Medical information | 22128 | Doctor diagnosed emphysema | Yes | Yes |
| **132** | Medical information | 22129 | Doctor diagnosed chronic bronchitis | Yes | Yes |
| **132** | Medical information | 22130 | Doctor diagnosed COPD (chronic obstructive pulmonary disease) | Yes | Yes |
| **132** | Medical information | 22131 | Doctor diagnosed cystic fibrosis | Yes | Yes |
| **132** | Medical information | 22132 | Doctor diagnosed alpha-1 antitrypsin deficiency | Yes | Yes |
| **132** | Medical information | 22133 | Doctor diagnosed sarcoidosis | Yes | Yes |
| **132** | Medical information | 22134 | Doctor diagnosed bronchiectasis | Yes | Yes |
| **132** | Medical information | 22135 | Doctor diagnosed idiopathic pulmonary fibrosis | Yes | Yes |
| **132** | Medical information | 22136 | Doctor diagnosed fibrosing alveolitis/unspecified alveolitis | Yes | Yes |
| **132** | Medical information | 22137 | Doctor diagnosed tuberculosis | Yes | Yes |
| **132** | Medical information | 22138 | Doctor diagnosed silicosis | Yes | Yes |
| **132** | Medical information | 22139 | Doctor diagnosed asbestosis | Yes | Yes |
| **132** | Medical information | 22140 | Doctor diagnosed lung cancer (not mesothelioma) | Yes | Yes |
| **132** | Medical information | 22141 | Doctor diagnosed mesothelioma of the lung | Yes | Yes |
| **132** | Medical information | 22146 | Age hayfever or allergic rhinitis diagnosed by doctor | No | No |
| **132** | Medical information | 22147 | Age asthma diagnosed by doctor | No | No |
| **132** | Medical information | 22148 | Age emphysema diagnosed by doctor | No | No |
| **132** | Medical information | 22149 | Age chronic bronchitis diagnosed by doctor | No | No |
| **132** | Medical information | 22150 | Age COPD (Chronic Obstructive Pulmonary Disease) diagnosed by doctor | No | No |
| **132** | Medical information | 22151 | Age cystic fibrosis diagnosed by doctor | No | No |
| **132** | Medical information | 22152 | Age alpha-1 antitrypsin deficiency diagnosed by doctor | No | No |
| **132** | Medical information | 22153 | Age sarcoidosis diagnosed by doctor | No | No |
| **132** | Medical information | 22154 | Age bronchiectasis diagnosed by doctor | No | No |
| **132** | Medical information | 22155 | Age idiopathic pulmonary fibrosis diagnosed by doctor | No | No |
| **132** | Medical information | 22156 | Age fibrosing alveolitis/unspecified alveolitis diagnosed by doctor | No | No |
| **132** | Medical information | 22157 | Age tuberculosis diagnosed by doctor | No | No |
| **132** | Medical information | 22158 | Age silicosis diagnosed by doctor | No | No |
| **132** | Medical information | 22159 | Age asbestosis diagnosed by doctor | No | No |
| **132** | Medical information | 22160 | Age lung cancer (not mesothelioma) diagnosed by doctor | No | No |
| **132** | Medical information | 22161 | Age mesothelioma of the lung diagnosed by doctor | No | No |
| **132** | Medical information | 22166 | Recent medication for hayfever or allergic rhinitis | Yes | Yes |
| **132** | Medical information | 22167 | Recent medication for asthma | Yes | Yes |
| **132** | Medical information | 22168 | Recent medication for emphysema | Yes | Yes |
| **132** | Medical information | 22169 | Recent medication for chronic bronchitis | Yes | Yes |
| **132** | Medical information | 22170 | Recent medication for COPD (Chronic Obstructive Pulmonary Disease) | Yes | Yes |
| **132** | Medical information | 22171 | Recent medication for cystic fibrosis | Yes | Yes |
| **132** | Medical information | 22172 | Recent medication for alpha-1 antitrypsin deficiency | Yes | Yes |
| **132** | Medical information | 22173 | Recent medication for sarcoidosis | Yes | Yes |
| **132** | Medical information | 22174 | Recent medication for bronchiectasis | Yes | Yes |
| **132** | Medical information | 22175 | Recent medication for idiopathic pulmonary fibrosis | Yes | Yes |
| **132** | Medical information | 22176 | Recent medication for fibrosing alveolitis/unspecified alveolitis | Yes | Yes |
| **132** | Medical information | 22177 | Recent medication for tuberculosis | Yes | Yes |
| **132** | Medical information | 22178 | Recent medication for silicosis | Yes | Yes |
| **132** | Medical information | 22179 | Recent medication for asbestosis | Yes | Yes |
| **132** | Medical information | 22180 | Recent medication for lung cancer (not mesothelioma) | Yes | Yes |
| **132** | Medical information | 22181 | Recent medication for mesothelioma of the lung | Yes | Yes |
| **100035** | HLA | 22182 | HLA imputation values | No | No |
| **265** | Telomeres | 22190 | Unadjusted T/S ratio | No | No |
| **265** | Telomeres | 22191 | Adjusted T/S ratio | No | No |
| **265** | Telomeres | 22192 | Z-adjusted T/S log | No | No |
| **265** | Telomeres | 22193 | Telomere measurement plate | No | No |
| **265** | Telomeres | 22194 | T/S ratio for regression dilution bias | No | No |
| **130** | Employment history | 22200 | Year of birth | No | No |
| **104** | ECG at rest, 12-lead | 22330 | PQ interval | No | No |
| **104** | ECG at rest, 12-lead | 22331 | QT interval | No | No |
| **104** | ECG at rest, 12-lead | 22332 | QTC interval | No | No |
| **104** | ECG at rest, 12-lead | 22333 | RR interval | No | No |
| **104** | ECG at rest, 12-lead | 22334 | PP interval | No | No |
| **104** | ECG at rest, 12-lead | 22335 | P axis | No | No |
| **104** | ECG at rest, 12-lead | 22336 | R axis | No | No |
| **104** | ECG at rest, 12-lead | 22337 | T axis | No | No |
| **104** | ECG at rest, 12-lead | 22338 | QRS num | No | No |
| **126** | Liver MRI | 22400 | Liver iron (Fe) | Yes | Yes |
| **126** | Liver MRI | 22401 | Liver inflammation factor (LIF) | No | No |
| **126** | Liver MRI | 22402 | Proton density fat fraction (PDFF) | No | No |
| **149** | Abdominal composition | 22403 | Anterior thigh lean muscle volume (right) | No | No |
| **149** | Abdominal composition | 22404 | Posterior thigh lean muscle volume (right) | No | No |
| **149** | Abdominal composition | 22405 | Anterior thigh lean muscle volume (left) | No | No |
| **149** | Abdominal composition | 22406 | Posterior thigh lean muscle volume (left) | No | No |
| **149** | Abdominal composition | 22407 | Visceral adipose tissue volume (VAT) | No | No |
| **149** | Abdominal composition | 22408 | Abdominal subcutaneous adipose tissue volume (ASAT) | No | No |
| **149** | Abdominal composition | 22409 | Total thigh muscle volume | No | No |
| **149** | Abdominal composition | 22410 | Total trunk fat volume | No | No |
| **149** | Abdominal composition | 22411 | VAT/ASAT error indicator | No | No |
| **149** | Abdominal composition | 22412 | Thigh error indicator (left) | No | No |
| **149** | Abdominal composition | 22413 | Thigh error indicator (right) | No | No |
| **149** | Abdominal composition | 22414 | Image quality indicator | No | No |
| **149** | Abdominal composition | 22415 | Total adipose tissue volume | No | No |
| **149** | Abdominal composition | 22416 | Total lean tissue volume | No | No |
| **126** | Liver MRI | 22417 | Liver iron corrected T1 (ct1) | Yes | Yes |
| **100315** | Genotype Results | 22418 | Genotype calls | No | No |
| **100315** | Genotype Results | 22419 | Genotype confidences | No | No |
| **133** | Left ventricular size and function | 22420 | LV ejection fraction | Yes | Yes |
| **133** | Left ventricular size and function | 22421 | LV end diastolic volume | Yes | Yes |
| **133** | Left ventricular size and function | 22422 | LV end systolic volume | Yes | Yes |
| **133** | Left ventricular size and function | 22423 | LV stroke volume | Yes | Yes |
| **133** | Left ventricular size and function | 22424 | Cardiac output | Yes | Yes |
| **133** | Left ventricular size and function | 22425 | Cardiac index | Yes | Yes |
| **133** | Left ventricular size and function | 22426 | Average heart rate | Yes | Yes |
| **133** | Left ventricular size and function | 22427 | Body surface area | Yes | Yes |
| **100315** | Genotype Results | 22430 | Genotype intensities | No | No |
| **100315** | Genotype Results | 22431 | Genotype copy number variants, log2ratios | No | No |
| **149** | Abdominal composition | 22432 | Total abdominal adipose tissue index | No | No |
| **149** | Abdominal composition | 22433 | Weight-to-muscle ratio | No | No |
| **149** | Abdominal composition | 22434 | Abdominal fat ratio | No | No |
| **149** | Abdominal composition | 22435 | Muscle fat infiltration | No | No |
| **149** | Abdominal composition | 22436 | Liver proton density fat fraction (AMRA) | No | No |
| **100315** | Genotype Results | 22437 | Genotype copy number variants B-allele frequencies | No | No |
| **100319** | Imputation | 22438 | Haplotypes (WTCHG) | No | No |
| **123** | Work environment | 22499 | Device used to enter occupational data | No | No |
| **123** | Work environment | 22500 | When occupational data entered | No | No |
| **123** | Work environment | 22501 | Year ended full time education | No | No |
| **132** | Medical information | 22502 | Cough on most days | No | No |
| **132** | Medical information | 22503 | Years of cough on most days | No | No |
| **132** | Medical information | 22504 | Bring up phlegm/sputum/mucus on most days | No | No |
| **132** | Medical information | 22505 | Years of bringing up phlegm/sputum/mucus on most days | No | No |
| **132** | Medical information | 22506 | Tobacco smoking | Yes | Yes |
| **132** | Medical information | 22507 | Age of stopping smoking | No | No |
| **132** | Medical information | 22508 | Amount of tobacco currently smoked | No | No |
| **130** | Employment history | 22599 | Number of jobs held | No | No |
| **130** | Employment history | 22600 | Job title | Yes | Yes |
| **130** | Employment history | 22601 | Job coding | No | No |
| **130** | Employment history | 22602 | Year job started | No | No |
| **130** | Employment history | 22603 | Year job ended | No | No |
| **130** | Employment history | 22604 | Work hours - lumped category | No | No |
| **130** | Employment history | 22605 | Work hours per week - exact value | Yes | Yes |
| **130** | Employment history | 22606 | Workplace very noisy | No | No |
| **130** | Employment history | 22607 | Workplace very cold | No | No |
| **130** | Employment history | 22608 | Workplace very hot | No | No |
| **130** | Employment history | 22609 | Workplace very dusty | No | No |
| **130** | Employment history | 22610 | Workplace full of chemical or other fumes | No | No |
| **130** | Employment history | 22611 | Workplace had a lot of cigarette smoke from other people smoking | No | No |
| **130** | Employment history | 22612 | Worked with materials containing asbestos | No | No |
| **130** | Employment history | 22613 | Worked with paints, thinners or glues | No | No |
| **130** | Employment history | 22614 | Worked with pesticides | No | No |
| **130** | Employment history | 22615 | Workplace had a lot of diesel exhaust | No | No |
| **130** | Employment history | 22616 | Breathing problems during period of job | No | No |
| **130** | Employment history | 22617 | Job code - historical | Yes | Yes |
| **130** | Employment history | 22618 | Breathing problems improved/stopped away from workplace or on holiday | No | No |
| **130** | Employment history | 22619 | Breathing problems responsible for leaving job | No | No |
| **130** | Employment history | 22620 | Job involved shift work | No | No |
| **130** | Employment history | 22630 | Day shifts worked | No | No |
| **130** | Employment history | 22631 | Period spent working day shifts | No | No |
| **130** | Employment history | 22640 | Mixture of day and night shifts worked | No | No |
| **130** | Employment history | 22641 | Period spent working mix of day and night shifts | No | No |
| **130** | Employment history | 22642 | Usual length of each night shift during mixed shift periods | No | No |
| **130** | Employment history | 22643 | Number of night shifts worked monthly during mixed shift periods | No | No |
| **130** | Employment history | 22644 | Consecutive night shifts during mixed shift periods | No | No |
| **130** | Employment history | 22645 | Rest days during mixed shift periods | No | No |
| **130** | Employment history | 22650 | Night shifts worked | No | No |
| **130** | Employment history | 22651 | Period spent working night shifts | No | No |
| **130** | Employment history | 22652 | Usual length of each night shift during night shift periods | No | No |
| **130** | Employment history | 22653 | Number of night shifts worked monthly during night shift periods | No | No |
| **130** | Employment history | 22654 | Consecutive night shifts during night shift periods | No | No |
| **130** | Employment history | 22655 | Rest days during night shift periods | No | No |
| **130** | Employment history | 22660 | Gap coding | No | No |
| **130** | Employment history | 22661 | Number of gap periods | No | No |
| **130** | Employment history | 22662 | Title for other gap | No | No |
| **130** | Employment history | 22663 | Year gap started | No | No |
| **130** | Employment history | 22664 | Year gap ended | No | No |
| **101** | Carotid ultrasound | 22670 | Minimum carotid IMT (intima-medial thickness) at 120 degrees | No | No |
| **101** | Carotid ultrasound | 22671 | Mean carotid IMT (intima-medial thickness) at 120 degrees | No | No |
| **101** | Carotid ultrasound | 22672 | Maximum carotid IMT (intima-medial thickness) at 120 degrees | No | No |
| **101** | Carotid ultrasound | 22673 | Minimum carotid IMT (intima-medial thickness) at 150 degrees | No | No |
| **101** | Carotid ultrasound | 22674 | Mean carotid IMT (intima-medial thickness) at 150 degrees | No | No |
| **101** | Carotid ultrasound | 22675 | Maximum carotid IMT (intima-medial thickness) at 150 degrees | No | No |
| **101** | Carotid ultrasound | 22676 | Minimum carotid IMT (intima-medial thickness) at 210 degrees | No | No |
| **101** | Carotid ultrasound | 22677 | Mean carotid IMT (intima-medial thickness) at 210 degrees | No | No |
| **101** | Carotid ultrasound | 22678 | Maximum carotid IMT (intima-medial thickness) at 210 degrees | No | No |
| **101** | Carotid ultrasound | 22679 | Minimum carotid IMT (intima-medial thickness) at 240 degrees | No | No |
| **101** | Carotid ultrasound | 22680 | Mean carotid IMT (intima-medial thickness) at 240 degrees | No | No |
| **101** | Carotid ultrasound | 22681 | Maximum carotid IMT (intima-medial thickness) at 240 degrees | No | No |
| **101** | Carotid ultrasound | 22682 | Quality control indicator for IMT at 120 degrees | No | No |
| **101** | Carotid ultrasound | 22683 | Quality control indicator for IMT at 150 degrees | No | No |
| **101** | Carotid ultrasound | 22684 | Quality control indicator for IMT at 210 degrees | No | No |
| **101** | Carotid ultrasound | 22685 | Quality control indicator for IMT at 240 degrees | No | No |
| **150** | Home locations | 22700 | Date first recorded at location | No | No |
| **150** | Home locations | 22701 | Home location - east co-ordinate | No | No |
| **150** | Home locations | 22702 | Home location - east co-ordinate (rounded) | No | No |
| **150** | Home locations | 22703 | Home location - north co-ordinate | No | No |
| **150** | Home locations | 22704 | Home location - north co-ordinate (rounded) | No | No |
| **100319** | Imputation | 22828 | Imputation from genotype (WTCHG) | No | No |
| **1307** | Infectious Disease Antigens | 23000 | 1gG antigen for Herpes Simplex virus-1 | Yes | Yes |
| **1307** | Infectious Disease Antigens | 23001 | 2mgG unique antigen for Herpes Simplex virus-2 | Yes | Yes |
| **1307** | Infectious Disease Antigens | 23002 | gE / gI antigen for Varicella Zoster Virus | Yes | Yes |
| **1307** | Infectious Disease Antigens | 23003 | VCA p18 antigen for Epstein-Barr Virus | Yes | Yes |
| **1307** | Infectious Disease Antigens | 23004 | EBNA-1 antigen for Epstein-Barr Virus | Yes | Yes |
| **1307** | Infectious Disease Antigens | 23005 | ZEBRA antigen for Epstein-Barr Virus | Yes | Yes |
| **1307** | Infectious Disease Antigens | 23006 | EA-D antigen for Epstein-Barr Virus | Yes | Yes |
| **1307** | Infectious Disease Antigens | 23007 | pp150 Nter antigen for Human Cytomegalovirus | Yes | Yes |
| **1307** | Infectious Disease Antigens | 23008 | pp 52 antigen for Human Cytomegalovirus | Yes | Yes |
| **1307** | Infectious Disease Antigens | 23009 | pp 28 antigen for Human Cytomegalovirus | Yes | Yes |
| **1307** | Infectious Disease Antigens | 23010 | IE1A antigen for Human Herpesvirus-6 | Yes | Yes |
| **1307** | Infectious Disease Antigens | 23011 | IE1B antigen for Human Herpesvirus-6 | Yes | Yes |
| **1307** | Infectious Disease Antigens | 23012 | p101 k antigen for Human Herpesvirus-6 | Yes | Yes |
| **1307** | Infectious Disease Antigens | 23013 | U14 antigen for Human Herpesvirus-7 | Yes | Yes |
| **1307** | Infectious Disease Antigens | 23014 | LANA antigen for Kaposi's Sarcoma-Associated Herpesvirus | Yes | Yes |
| **1307** | Infectious Disease Antigens | 23015 | K8.1 antigen for Kaposi's Sarcoma-Associated Herpesvirus | No | Yes |
| **1307** | Infectious Disease Antigens | 23016 | HBc antigen for Hepatitis B Virus | Yes | Yes |
| **1307** | Infectious Disease Antigens | 23017 | HBe antigen for Hepatitis B Virus | Yes | Yes |
| **1307** | Infectious Disease Antigens | 23018 | Core antigen for Hepatitis C Virus | Yes | Yes |
| **1307** | Infectious Disease Antigens | 23019 | NS3 antigen for Hepatitis C Virus | Yes | Yes |
| **1307** | Infectious Disease Antigens | 23020 | p22 antigen for Toxoplasma gondii | Yes | Yes |
| **1307** | Infectious Disease Antigens | 23021 | sag1 antigen for Toxoplasma gondii | Yes | Yes |
| **1307** | Infectious Disease Antigens | 23022 | HTLV-1 gag antigen for Human T-Lymphotropic Virus 1 | Yes | Yes |
| **1307** | Infectious Disease Antigens | 23023 | HTLV-1 env antigen for Human T-Lymphotropic Virus 1 | Yes | Yes |
| **1307** | Infectious Disease Antigens | 23024 | HIV-1 gag antigen for Human Immunodeficiency Virus | Yes | Yes |
| **1307** | Infectious Disease Antigens | 23025 | HIV-1 env antigen for Human Immunodeficiency Virus | Yes | Yes |
| **1307** | Infectious Disease Antigens | 23026 | BK VP1 antigen for Human Polyomavirus BKV | Yes | Yes |
| **1307** | Infectious Disease Antigens | 23027 | JC VP1 antigen for Human Polyomavirus JCV | Yes | Yes |
| **1307** | Infectious Disease Antigens | 23028 | MC VP1 antigen for Merkel Cell Polyomavirus | Yes | Yes |
| **1307** | Infectious Disease Antigens | 23029 | L1 antigen for Human Papillomavirus type-16 | Yes | Yes |
| **1307** | Infectious Disease Antigens | 23030 | E6 antigen for Human Papillomavirus type-16 | Yes | Yes |
| **1307** | Infectious Disease Antigens | 23031 | E7 antigen for Human Papillomavirus type-16 | Yes | Yes |
| **1307** | Infectious Disease Antigens | 23032 | L1 antigen for Human Papillomavirus type-18 | Yes | Yes |
| **1307** | Infectious Disease Antigens | 23033 | momp D antigen for Chlamydia trachomatis | Yes | Yes |
| **1307** | Infectious Disease Antigens | 23034 | momp A antigen for Chlamydia trachomatis | Yes | Yes |
| **1307** | Infectious Disease Antigens | 23035 | tarp-D F1 antigen for Chlamydia trachomatis | Yes | Yes |
| **1307** | Infectious Disease Antigens | 23036 | tarp-D F2 antigen for Chlamydia trachomatis | Yes | Yes |
| **1307** | Infectious Disease Antigens | 23037 | PorB antigen for Chlamydia trachomatis | Yes | Yes |
| **1307** | Infectious Disease Antigens | 23038 | pGP3 antigen for Chlamydia trachomatis | Yes | Yes |
| **1307** | Infectious Disease Antigens | 23039 | CagA antigen for Helicobacter pylori | Yes | Yes |
| **1307** | Infectious Disease Antigens | 23040 | VacA antigen for Helicobacter pylori | Yes | Yes |
| **1307** | Infectious Disease Antigens | 23041 | OMP antigen for Helicobacter pylori | Yes | Yes |
| **1307** | Infectious Disease Antigens | 23042 | GroEL antigen for Helicobacter pylori | Yes | Yes |
| **1307** | Infectious Disease Antigens | 23043 | Catalase antigen for Helicobacter pylori | Yes | Yes |
| **1307** | Infectious Disease Antigens | 23044 | UreA antigen for Helicobacter pylori | Yes | Yes |
| **155** | Mood | 23045 | Very nervous mood over last week | No | No |
| **155** | Mood | 23046 | Down in dumps over last week | No | No |
| **155** | Mood | 23047 | Felt calm over last week | No | No |
| **1307** | Infectious Disease Antigens | 23048 | Antigen assay date | No | No |
| **1307** | Infectious Disease Antigens | 23049 | Antigen assay QC indicator | No | No |
| **51428** | Infectious Diseases | 23050 | HSV-1 seropositivity for Herpes Simplex virus-1 | No | No |
| **51428** | Infectious Diseases | 23051 | HSV-2 seropositivity for Herpes Simplex virus-2 | No | No |
| **51428** | Infectious Diseases | 23052 | VZV seropositivity for Varicella Zoster Virus | No | No |
| **51428** | Infectious Diseases | 23053 | EBV seropositivity for Epstein-Barr Virus | No | No |
| **51428** | Infectious Diseases | 23054 | CMV seropositivity for Human Cytomegalovirus | No | No |
| **51428** | Infectious Diseases | 23055 | HHV-6 overall seropositivity for Human Herpesvirus-6 | No | No |
| **51428** | Infectious Diseases | 23056 | HHV-6A seropositivity for Human Herpesvirus-6 | No | No |
| **51428** | Infectious Diseases | 23057 | HHV-6B seropositivity for Human Herpesvirus-6 | No | No |
| **51428** | Infectious Diseases | 23058 | HHV-7 seropositivity for Human Herpesvirus-7 | No | No |
| **51428** | Infectious Diseases | 23059 | KSHV seropositivity for Kaposi's Sarcoma-Associated Herpesvirus | No | No |
| **51428** | Infectious Diseases | 23060 | HBV seropositivity for Hepatitis B Virus | No | No |
| **51428** | Infectious Diseases | 23061 | HCV seropositivity for Hepatitis C Virus | No | No |
| **51428** | Infectious Diseases | 23062 | T. gondii seropositivity for Toxoplasma gondii | No | No |
| **51428** | Infectious Diseases | 23063 | HTLV-1 seropositivity for Human T-Lymphotropic Virus 1 | No | No |
| **51428** | Infectious Diseases | 23064 | HIV-1 seropositivity for Human Immunodeficiency Virus | No | No |
| **51428** | Infectious Diseases | 23065 | BKV seropositivity for Human Polyomavirus BKV | No | No |
| **51428** | Infectious Diseases | 23066 | JCV seropositivity for Human Polyomavirus JCV | No | No |
| **51428** | Infectious Diseases | 23067 | MCV seropositivity for Merkel Cell Polyomavirus | No | No |
| **51428** | Infectious Diseases | 23068 | HPV 16 Definition I seropositivity for Human Papillomavirus type-16 | No | No |
| **51428** | Infectious Diseases | 23069 | HPV 18 seropositivity for Human Papillomavirus type-18 | No | No |
| **51428** | Infectious Diseases | 23070 | C. trachomatis Definition I seropositivity for Chlamydia trachomatis | No | No |
| **51428** | Infectious Diseases | 23071 | C. trachomatis Definition II seropositivity for Chlamydia trachomatis | No | No |
| **155** | Mood | 23072 | Downhearted and depressed over last week | No | No |
| **51428** | Infectious Diseases | 23073 | H. pylori Definition I seropositivity for Helicobacter pylori | No | No |
| **51428** | Infectious Diseases | 23074 | H. pylori Definition II seropositivity for Helicobacter pylori | No | No |
| **51428** | Infectious Diseases | 23075 | HPV 16 Definition II seropositivity for Human Papillomavirus type-16 | No | No |
| **155** | Mood | 23076 | Happy over last week | No | No |
| **116** | Cognitive function online | 23077 | Device used for typing | No | No |
| **116** | Cognitive function online | 23078 | Device used for pointing | No | No |
| **155** | Mood | 23079 | When mood described | No | No |
| **187** |  | 23080 | Sequencing Provider | No | No |
| **187** |  | 23081 | Sample plate ID | No | No |
| **187** |  | 23082 | Shipment batch number | No | No |
| **187** |  | 23083 | Sample quant reading (UKB) | No | No |
| **187** |  | 23084 | Sample quant reading (sequence provider) | No | No |
| **187** |  | 23085 | Library prep plate barcode | No | No |
| **187** |  | 23086 | Library prep plate position (well) | No | No |
| **187** |  | 23087 | Yield | No | No |
| **187** |  | 23088 | Proportion of mapped read pairs | No | No |
| **187** |  | 23089 | Coverage | No | No |
| **187** |  | 23090 | Average batch coverage | No | No |
| **187** |  | 23091 | Freemix verify BAM ID | No | No |
| **187** |  | 23092 | NRD genotyping | No | No |
| **187** |  | 23093 | Quality requirements achieved | No | No |
| **187** |  | 23094 | Read haps | No | No |
| **100009** | Impedance measures | 23098 | Weight | Yes | Yes |
| **100009** | Impedance measures | 23099 | Body fat percentage | Yes | Yes |
| **100009** | Impedance measures | 23100 | Whole body fat mass | Yes | Yes |
| **100009** | Impedance measures | 23101 | Whole body fat-free mass | Yes | Yes |
| **100009** | Impedance measures | 23102 | Whole body water mass | Yes | Yes |
| **100009** | Impedance measures | 23104 | Body mass index (BMI) | Yes | Yes |
| **100009** | Impedance measures | 23105 | Basal metabolic rate | Yes | Yes |
| **100009** | Impedance measures | 23106 | Impedance of whole body | No | No |
| **100009** | Impedance measures | 23107 | Impedance of leg (right) | No | No |
| **100009** | Impedance measures | 23108 | Impedance of leg (left) | No | No |
| **100009** | Impedance measures | 23109 | Impedance of arm (right) | No | No |
| **100009** | Impedance measures | 23110 | Impedance of arm (left) | No | No |
| **100009** | Impedance measures | 23111 | Leg fat percentage (right) | No | No |
| **100009** | Impedance measures | 23112 | Leg fat mass (right) | No | No |
| **100009** | Impedance measures | 23113 | Leg fat-free mass (right) | No | No |
| **100009** | Impedance measures | 23114 | Leg predicted mass (right) | No | No |
| **100009** | Impedance measures | 23115 | Leg fat percentage (left) | No | No |
| **100009** | Impedance measures | 23116 | Leg fat mass (left) | No | No |
| **100009** | Impedance measures | 23117 | Leg fat-free mass (left) | No | No |
| **100009** | Impedance measures | 23118 | Leg predicted mass (left) | No | No |
| **100009** | Impedance measures | 23119 | Arm fat percentage (right) | No | No |
| **100009** | Impedance measures | 23120 | Arm fat mass (right) | No | No |
| **100009** | Impedance measures | 23121 | Arm fat-free mass (right) | No | No |
| **100009** | Impedance measures | 23122 | Arm predicted mass (right) | No | No |
| **100009** | Impedance measures | 23123 | Arm fat percentage (left) | No | No |
| **100009** | Impedance measures | 23124 | Arm fat mass (left) | No | No |
| **100009** | Impedance measures | 23125 | Arm fat-free mass (left) | No | No |
| **100009** | Impedance measures | 23126 | Arm predicted mass (left) | No | No |
| **100009** | Impedance measures | 23127 | Trunk fat percentage | No | No |
| **100009** | Impedance measures | 23128 | Trunk fat mass | No | No |
| **100009** | Impedance measures | 23129 | Trunk fat-free mass | No | No |
| **100009** | Impedance measures | 23130 | Trunk predicted mass | No | No |
| **170** | Exome sequences | 23141 | Exome OQFE variant call files (VCFs) | No | No |
| **170** | Exome sequences | 23142 | Exome OQFE variant call file (VCF) indices | No | No |
| **170** | Exome sequences | 23143 | Exome OQFE CRAM files | No | No |
| **170** | Exome sequences | 23144 | Exome OQFE CRAM indices | No | No |
| **171** |  | 23145 | Population level exome OQFE variants, PLINK format - interim 300k release | No | No |
| **171** |  | 23146 | Population level exome OQFE variants, pVCF format - interim 300k release | No | No |
| **171** |  | 23147 | Population level exome OQFE variants, BGEN format - interim 300k release | No | No |
| **170** | Exome sequences | 23148 | Population level exome OQFE variants, pVCF format - interim 450k release | No | No |
| **170** | Exome sequences | 23149 | Population level exome OQFE variants, PLINK format - interim 450k release | No | No |
| **170** | Exome sequences | 23150 | Population level exome OQFE variants, BGEN format - interim 450k release | No | No |
| **171** |  | 23151 | Exome OQFE variant call files (VCFs) - interim 200k release | No | No |
| **171** |  | 23152 | Exome OQFE variant call file (VCF) indices - interim 200k release | No | No |
| **171** |  | 23153 | Exome OQFE CRAM files - interim 200k release | No | No |
| **171** |  | 23154 | Exome OQFE CRAM indices - interim 200k release | No | No |
| **171** |  | 23155 | Population level exome OQFE variants, PLINK format - interim 200k release | No | No |
| **171** |  | 23156 | Population level exome OQFE variants, pVCF format - interim 200k release | No | No |
| **170** | Exome sequences | 23157 | Population level exome OQFE variants, pVCF format - 500k release | No | No |
| **170** | Exome sequences | 23158 | Population level exome OQFE variants, PLINK format - 500k release | No | No |
| **170** | Exome sequences | 23159 | Population level exome OQFE variants, BGEN format - 500k release | No | No |
| **171** |  | 23160 | Population-level FE variants, PLINK format - initial 50k release | No | No |
| **171** |  | 23161 | Exome FE variant call files (VCFs) - initial 50k release | No | No |
| **171** |  | 23162 | Exome FE variant calls indices - initial 50k release | No | No |
| **171** |  | 23163 | Exome FE CRAM files - initial 50k release | No | No |
| **171** |  | 23164 | Exome FE CRAM indices - initial 50k release | No | No |
| **264** | Genetically deduced phenotypes | 23165 | Blood-type haplotype | No | No |
| **170** | Exome sequences | 23170 | INVALID Population-level SPB variants, PLINK format | No | No |
| **171** |  | 23176 | Exome SPB variant call files (VCFs) - initial 50k release | No | No |
| **171** |  | 23177 | Exome SPB variant calls indices - initial 50k release | No | No |
| **171** |  | 23178 | Exome SPB CRAM files - initial 50k release | No | No |
| **171** |  | 23179 | Exome SPB CRAM indices - initial 50k release | No | No |
| **181** | BGI WGS pilot | 23181 | BGI WGS CRAM files | No | No |
| **181** | BGI WGS pilot | 23182 | BGI WGS CRAM indices | No | No |
| **182** | Broad WGS pilot | 23183 | Broad WGS CRAM files | No | No |
| **182** | Broad WGS pilot | 23184 | Broad WGS CRAM indices | No | No |
| **180** | Whole genome sequences | 23191 | Whole genome variant call files (VCFs) | No | No |
| **180** | Whole genome sequences | 23192 | Whole genome variant calls indices | No | No |
| **180** | Whole genome sequences | 23193 | Whole genome CRAM files | No | No |
| **180** | Whole genome sequences | 23194 | Whole genome CRAM indices | No | No |
| **186** |  | 23195 | Whole genome GraphTyper joint call pVCF (deprecated) | No | No |
| **186** |  | 23196 | Whole genome GATK joint call pVCF | No | No |
| **125** | Bone size, mineral and density by DXA | 23200 | L1-L4 area | No | No |
| **125** | Bone size, mineral and density by DXA | 23201 | L1-L4 average height | No | No |
| **125** | Bone size, mineral and density by DXA | 23202 | L1-L4 average width | No | No |
| **125** | Bone size, mineral and density by DXA | 23203 | L1-L4 BMC (bone mineral content) | No | No |
| **125** | Bone size, mineral and density by DXA | 23204 | L1-L4 BMD (bone mineral density) | Yes | Yes |
| **125** | Bone size, mineral and density by DXA | 23205 | L1-L4 BMD (bone mineral density) T-score | Yes | Yes |
| **125** | Bone size, mineral and density by DXA | 23206 | Femur lower neck BMD (bone mineral density) (right) | Yes | Yes |
| **125** | Bone size, mineral and density by DXA | 23207 | Femur lower neck BMD (bone mineral density) T-score (right) | Yes | Yes |
| **125** | Bone size, mineral and density by DXA | 23208 | Femur neck BMD (bone mineral density) (right) | Yes | Yes |
| **125** | Bone size, mineral and density by DXA | 23209 | Femur neck BMD (bone mineral density) T-score (right) | Yes | Yes |
| **125** | Bone size, mineral and density by DXA | 23210 | Femur shaft BMD (bone mineral density) (right) | Yes | Yes |
| **125** | Bone size, mineral and density by DXA | 23211 | Femur shaft BMD (bone mineral density) T-score (right) | Yes | Yes |
| **125** | Bone size, mineral and density by DXA | 23212 | Femur total BMD (bone mineral density) (right) | Yes | Yes |
| **125** | Bone size, mineral and density by DXA | 23213 | Femur total BMD (bone mineral density) T-score (right) | Yes | Yes |
| **125** | Bone size, mineral and density by DXA | 23214 | Femur troch BMD (bone mineral density) (right) | Yes | Yes |
| **125** | Bone size, mineral and density by DXA | 23215 | Femur troch BMD (bone mineral density) T-score (right) | Yes | Yes |
| **125** | Bone size, mineral and density by DXA | 23216 | Femur upper neck BMD (bone mineral density) (right) | Yes | Yes |
| **125** | Bone size, mineral and density by DXA | 23217 | Femur upper neck BMD (bone mineral density) T-score (right) | Yes | Yes |
| **125** | Bone size, mineral and density by DXA | 23218 | Femur wards BMD (bone mineral density) (right) | Yes | Yes |
| **125** | Bone size, mineral and density by DXA | 23219 | Femur wards BMD (bone mineral density) T-score (right) | Yes | Yes |
| **125** | Bone size, mineral and density by DXA | 23220 | Arm BMC (bone mineral content) (left) | No | No |
| **125** | Bone size, mineral and density by DXA | 23221 | Arm BMD (bone mineral density) (left) | Yes | Yes |
| **125** | Bone size, mineral and density by DXA | 23222 | Arm BMC (bone mineral content) (right) | No | No |
| **125** | Bone size, mineral and density by DXA | 23223 | Arm BMD (bone mineral density) (right) | Yes | Yes |
| **125** | Bone size, mineral and density by DXA | 23224 | Arms BMC (bone mineral content) | No | No |
| **125** | Bone size, mineral and density by DXA | 23225 | Arms BMD (bone mineral density) | Yes | Yes |
| **125** | Bone size, mineral and density by DXA | 23226 | Head BMD (bone mineral density) | No | No |
| **125** | Bone size, mineral and density by DXA | 23227 | Leg BMD (bone mineral density) (left) | Yes | Yes |
| **125** | Bone size, mineral and density by DXA | 23228 | Leg BMC (bone mineral content) (right) | No | No |
| **125** | Bone size, mineral and density by DXA | 23229 | Leg BMD (bone mineral density) (right) | Yes | Yes |
| **125** | Bone size, mineral and density by DXA | 23230 | Legs BMC (bone mineral content) | No | No |
| **125** | Bone size, mineral and density by DXA | 23231 | Legs BMD (bone mineral density) | Yes | Yes |
| **125** | Bone size, mineral and density by DXA | 23232 | Pelvis BMD (bone mineral density) | No | No |
| **125** | Bone size, mineral and density by DXA | 23233 | Ribs BMD (bone mineral density) | No | No |
| **125** | Bone size, mineral and density by DXA | 23234 | Spine BMD (bone mineral density) | No | No |
| **125** | Bone size, mineral and density by DXA | 23235 | Total BMC (bone mineral content) | No | No |
| **125** | Bone size, mineral and density by DXA | 23236 | Total BMD (bone mineral density) | Yes | Yes |
| **125** | Bone size, mineral and density by DXA | 23237 | Total BMD (bone mineral density) (left) | Yes | Yes |
| **125** | Bone size, mineral and density by DXA | 23238 | Total BMD (bone mineral density) (right) | Yes | Yes |
| **125** | Bone size, mineral and density by DXA | 23239 | Total BMD (bone mineral density) T-score | No | No |
| **125** | Bone size, mineral and density by DXA | 23240 | Trunk BMC (bone mineral content) | No | No |
| **125** | Bone size, mineral and density by DXA | 23241 | Trunk BMD (bone mineral density) | No | No |
| **125** | Bone size, mineral and density by DXA | 23242 | Trunk BMD (bone mineral density) (left) | No | No |
| **125** | Bone size, mineral and density by DXA | 23243 | Trunk BMD (bone mineral density) (right) | No | No |
| **124** | Body composition by DXA | 23244 | Android bone mass | No | No |
| **124** | Body composition by DXA | 23245 | Android fat mass | No | No |
| **124** | Body composition by DXA | 23246 | Android lean mass | No | No |
| **124** | Body composition by DXA | 23247 | Android tissue fat percentage | No | No |
| **124** | Body composition by DXA | 23248 | Android total mass | No | No |
| **124** | Body composition by DXA | 23249 | Arm fat mass (left) | No | No |
| **124** | Body composition by DXA | 23250 | Arm lean mass (left) | No | No |
| **124** | Body composition by DXA | 23251 | Arm tissue fat percentage (left) | No | No |
| **124** | Body composition by DXA | 23252 | Arm total mass (left) | No | No |
| **124** | Body composition by DXA | 23253 | Arm fat mass (right) | No | No |
| **124** | Body composition by DXA | 23254 | Arm lean mass (right) | No | No |
| **124** | Body composition by DXA | 23255 | Arm tissue fat percentage (right) | No | No |
| **124** | Body composition by DXA | 23256 | Arm total mass (right) | No | No |
| **124** | Body composition by DXA | 23257 | Arms fat mass | No | No |
| **124** | Body composition by DXA | 23258 | Arms lean mass | No | No |
| **124** | Body composition by DXA | 23259 | Arms tissue fat percentage | No | No |
| **124** | Body composition by DXA | 23260 | Arms total mass | No | No |
| **124** | Body composition by DXA | 23261 | Gynoid bone mass | No | No |
| **124** | Body composition by DXA | 23262 | Gynoid fat mass | No | No |
| **124** | Body composition by DXA | 23263 | Gynoid lean mass | No | No |
| **124** | Body composition by DXA | 23264 | Gynoid tissue fat percentage | No | No |
| **124** | Body composition by DXA | 23265 | Gynoid total mass | No | No |
| **124** | Body composition by DXA | 23266 | Leg fat mass (left) | No | No |
| **124** | Body composition by DXA | 23267 | Leg lean mass (left) | No | No |
| **124** | Body composition by DXA | 23268 | Leg tissue fat percentage (left) | No | No |
| **124** | Body composition by DXA | 23269 | Leg total mass (left) | No | No |
| **124** | Body composition by DXA | 23270 | Leg fat mass (right) | No | No |
| **124** | Body composition by DXA | 23271 | Leg lean mass (right) | No | No |
| **124** | Body composition by DXA | 23272 | Leg tissue fat percentage (right) | No | No |
| **124** | Body composition by DXA | 23273 | Leg total mass (right) | No | No |
| **124** | Body composition by DXA | 23274 | Legs fat mass | No | No |
| **124** | Body composition by DXA | 23275 | Legs lean mass | No | No |
| **124** | Body composition by DXA | 23276 | Legs tissue fat percentage | No | No |
| **124** | Body composition by DXA | 23277 | Legs total mass | No | No |
| **124** | Body composition by DXA | 23278 | Total fat mass | No | No |
| **124** | Body composition by DXA | 23279 | Total fat-free mass | No | No |
| **124** | Body composition by DXA | 23280 | Total lean mass | No | No |
| **124** | Body composition by DXA | 23281 | Total tissue fat percentage | No | No |
| **124** | Body composition by DXA | 23282 | Total tissue mass | No | No |
| **124** | Body composition by DXA | 23283 | Total mass | No | No |
| **124** | Body composition by DXA | 23284 | Trunk fat mass | No | No |
| **124** | Body composition by DXA | 23285 | Trunk lean mass | No | No |
| **124** | Body composition by DXA | 23286 | Trunk tissue fat percentage | No | No |
| **124** | Body composition by DXA | 23287 | Trunk total mass | No | No |
| **124** | Body composition by DXA | 23288 | VAT (visceral adipose tissue) mass | No | No |
| **124** | Body composition by DXA | 23289 | VAT (visceral adipose tissue) volume | No | No |
| **125** | Bone size, mineral and density by DXA | 23290 | Femur shaft BMD (bone mineral density) (left) | Yes | Yes |
| **125** | Bone size, mineral and density by DXA | 23291 | Femur total BMD (bone mineral density) (left) | Yes | Yes |
| **125** | Bone size, mineral and density by DXA | 23292 | Femur upper neck BMD (bone mineral density) (left) | Yes | Yes |
| **125** | Bone size, mineral and density by DXA | 23293 | Femur total BMD (bone mineral density) T-score (left) | Yes | Yes |
| **125** | Bone size, mineral and density by DXA | 23294 | Femur lower neck BMD (bone mineral density) T-score (left) | Yes | Yes |
| **125** | Bone size, mineral and density by DXA | 23295 | Femur troch BMD (bone mineral density) (left) | Yes | Yes |
| **125** | Bone size, mineral and density by DXA | 23296 | Femur upper neck BMD (bone mineral density) T-score (left) | Yes | Yes |
| **125** | Bone size, mineral and density by DXA | 23297 | Femur wards BMD (bone mineral density) (left) | Yes | Yes |
| **125** | Bone size, mineral and density by DXA | 23298 | Femur troch BMD (bone mineral density) T-score (left) | Yes | Yes |
| **125** | Bone size, mineral and density by DXA | 23299 | Femur neck BMD (bone mineral density) (left) | Yes | Yes |
| **125** | Bone size, mineral and density by DXA | 23300 | Femur neck BMD (bone mineral density) T-score (left) | Yes | Yes |
| **125** | Bone size, mineral and density by DXA | 23301 | Femur wards BMD (bone mineral density) T-score (left) | Yes | Yes |
| **125** | Bone size, mineral and density by DXA | 23302 | Femur lower neck BMD (bone mineral density) (left) | Yes | Yes |
| **125** | Bone size, mineral and density by DXA | 23303 | Femur shaft BMD (bone mineral density) T-score (left) | Yes | Yes |
| **125** | Bone size, mineral and density by DXA | 23304 | Trunk bone area | No | No |
| **125** | Bone size, mineral and density by DXA | 23305 | Head bone area | No | No |
| **125** | Bone size, mineral and density by DXA | 23306 | Head BMC (bone mineral content) | No | No |
| **125** | Bone size, mineral and density by DXA | 23307 | Pelvis bone area | No | No |
| **125** | Bone size, mineral and density by DXA | 23308 | Pelvis BMC (bone mineral content) | No | No |
| **125** | Bone size, mineral and density by DXA | 23309 | Ribs bone area | No | No |
| **125** | Bone size, mineral and density by DXA | 23310 | Ribs BMC (bone mineral content) | No | No |
| **125** | Bone size, mineral and density by DXA | 23311 | Spine bone area | No | No |
| **125** | Bone size, mineral and density by DXA | 23312 | Spine BMC (bone mineral content) | No | No |
| **125** | Bone size, mineral and density by DXA | 23313 | Arm bone area (left) | No | No |
| **125** | Bone size, mineral and density by DXA | 23314 | Arm bone area (right) | No | No |
| **125** | Bone size, mineral and density by DXA | 23315 | Leg bone area (left) | No | No |
| **125** | Bone size, mineral and density by DXA | 23316 | Leg bone area (right) | No | No |
| **125** | Bone size, mineral and density by DXA | 23317 | Arms combined bone area | No | No |
| **125** | Bone size, mineral and density by DXA | 23318 | Legs combined bone area | No | No |
| **125** | Bone size, mineral and density by DXA | 23320 | Leg BMC (bone mineral content) (left) | No | No |
| **502** | Symbol digit substitution | 23321 | Values wanted | No | No |
| **502** | Symbol digit substitution | 23322 | Values entered | No | No |
| **502** | Symbol digit substitution | 23323 | Number of symbol digit matches attempted | No | No |
| **502** | Symbol digit substitution | 23324 | Number of symbol digit matches made correctly | No | No |
| **125** | Bone size, mineral and density by DXA | 23325 | Femur neck bone area (left) | No | No |
| **125** | Bone size, mineral and density by DXA | 23326 | Femur neck bone area (right) | No | No |
| **125** | Bone size, mineral and density by DXA | 23327 | Femur neck BMC (bone mineral content) (left) | No | No |
| **125** | Bone size, mineral and density by DXA | 23328 | Femur neck BMC (bone mineral content) (right) | No | No |
| **125** | Bone size, mineral and density by DXA | 23329 | Femur shaft bone area (left) | No | No |
| **125** | Bone size, mineral and density by DXA | 23330 | Femur shaft bone area (right) | No | No |
| **125** | Bone size, mineral and density by DXA | 23331 | Femur shaft BMC (bone mineral content) (left) | No | No |
| **125** | Bone size, mineral and density by DXA | 23332 | Femur shaft BMC (bone mineral content) (right) | No | No |
| **125** | Bone size, mineral and density by DXA | 23333 | Femur total area (left) | No | No |
| **125** | Bone size, mineral and density by DXA | 23334 | Femur total area (right) | No | No |
| **125** | Bone size, mineral and density by DXA | 23335 | Femur total BMC (bone mineral content) (left) | No | No |
| **125** | Bone size, mineral and density by DXA | 23336 | Femur total BMC (bone mineral content) (right) | No | No |
| **125** | Bone size, mineral and density by DXA | 23337 | Femur troch bone area (left) | No | No |
| **125** | Bone size, mineral and density by DXA | 23338 | Femur troch bone area (right) | No | No |
| **125** | Bone size, mineral and density by DXA | 23339 | Femur troch BMC (bone mineral content) (left) | No | No |
| **125** | Bone size, mineral and density by DXA | 23340 | Femur troch BMC (bone mineral content) (right) | No | No |
| **125** | Bone size, mineral and density by DXA | 23341 | Femur wards bone area (left) | No | No |
| **125** | Bone size, mineral and density by DXA | 23342 | Femur wards bone area (left) | No | No |
| **125** | Bone size, mineral and density by DXA | 23343 | Femur wards BMC (bone mineral content) (left) | No | No |
| **125** | Bone size, mineral and density by DXA | 23344 | Femur wards BMC (bone mineral content) (right) | No | No |
| **180** | Whole genome sequences | 23350 | Manta-called scored structural variant and indel candidates | No | No |
| **180** | Whole genome sequences | 23351 | Manta-called unscored structural variant and indel candidates | No | No |
| **149** | Abdominal composition | 23355 | Posterior thigh muscle fat infiltration (MFI) (left) | No | No |
| **149** | Abdominal composition | 23356 | Posterior thigh muscle fat infiltration (MFI) (right) | No | No |
| **149** | Abdominal composition | 23357 | VAT error indicator | No | No |
| **149** | Abdominal composition | 23358 | ASAT error indicator | No | No |
| **149** | Abdominal composition | 23359 | Anterior thigh error indicator (left) | No | No |
| **149** | Abdominal composition | 23360 | Posterior thigh error indicator (left) | No | No |
| **149** | Abdominal composition | 23361 | Anterior thigh error indicator (right) | No | No |
| **149** | Abdominal composition | 23362 | Posterior thigh error indicator (right) | No | No |
| **149** | Abdominal composition | 23363 | 10P liver PDFF mean error indicator | No | No |
| **149** | Abdominal composition | 23364 | FR liver PDFF mean error indicator | No | No |
| **180** | Whole genome sequences | 23370 | Whole genome variant call files (VCFs) (reserved) | No | No |
| **180** | Whole genome sequences | 23371 | Whole genome variant calls indices (reserved) | No | No |
| **180** | Whole genome sequences | 23372 | Whole genome CRAM files (reserved) | No | No |
| **180** | Whole genome sequences | 23373 | Whole genome CRAM indices (reserved) | No | No |
| **180** | Whole genome sequences | 23374 | Population level WGS variants, pVCF format - 500k release | No | No |
| **186** |  | 23375 | Whole genome GATK joint call pVCF (reserved) | No | No |
| **220** |  | 23400 | Total Cholesterol | No | No |
| **220** |  | 23401 | Total Cholesterol Minus HDL-C | No | No |
| **220** |  | 23402 | Remnant Cholesterol (Non-HDL, Non-LDL -Cholesterol) | No | No |
| **220** |  | 23403 | VLDL Cholesterol | No | No |
| **220** |  | 23404 | Clinical LDL Cholesterol | No | No |
| **220** |  | 23405 | LDL Cholesterol | No | No |
| **220** |  | 23406 | HDL Cholesterol | No | No |
| **220** |  | 23407 | Total Triglycerides | No | No |
| **220** |  | 23408 | Triglycerides in VLDL | No | No |
| **220** |  | 23409 | Triglycerides in LDL | No | No |
| **220** |  | 23410 | Triglycerides in HDL | No | No |
| **220** |  | 23411 | Total Phospholipids in Lipoprotein Particles | No | No |
| **220** |  | 23412 | Phospholipids in VLDL | No | No |
| **220** |  | 23413 | Phospholipids in LDL | No | No |
| **220** |  | 23414 | Phospholipids in HDL | No | No |
| **220** |  | 23415 | Total Esterified Cholesterol | No | No |
| **220** |  | 23416 | Cholesteryl Esters in VLDL | No | No |
| **220** |  | 23417 | Cholesteryl Esters in LDL | No | No |
| **220** |  | 23418 | Cholesteryl Esters in HDL | No | No |
| **220** |  | 23419 | Total Free Cholesterol | No | No |
| **220** |  | 23420 | Free Cholesterol in VLDL | No | No |
| **220** |  | 23421 | Free Cholesterol in LDL | No | No |
| **220** |  | 23422 | Free Cholesterol in HDL | No | No |
| **220** |  | 23423 | Total Lipids in Lipoprotein Particles | No | No |
| **220** |  | 23424 | Total Lipids in VLDL | No | No |
| **220** |  | 23425 | Total Lipids in LDL | No | No |
| **220** |  | 23426 | Total Lipids in HDL | No | No |
| **220** |  | 23427 | Total Concentration of Lipoprotein Particles | No | No |
| **220** |  | 23428 | Concentration of VLDL Particles | No | No |
| **220** |  | 23429 | Concentration of LDL Particles | No | No |
| **220** |  | 23430 | Concentration of HDL Particles | No | No |
| **220** |  | 23431 | Average Diameter for VLDL Particles | No | No |
| **220** |  | 23432 | Average Diameter for LDL Particles | No | No |
| **220** |  | 23433 | Average Diameter for HDL Particles | No | No |
| **220** |  | 23434 | Phosphoglycerides | No | No |
| **220** |  | 23435 | Triglycerides to Phosphoglycerides ratio | No | No |
| **220** |  | 23436 | Total Cholines | No | No |
| **220** |  | 23437 | Phosphatidylcholines | No | No |
| **220** |  | 23438 | Sphingomyelins | No | No |
| **220** |  | 23439 | Apolipoprotein B | No | No |
| **220** |  | 23440 | Apolipoprotein A1 | No | No |
| **220** |  | 23441 | Apolipoprotein B to Apolipoprotein A1 ratio | No | No |
| **220** |  | 23442 | Total Fatty Acids | No | No |
| **220** |  | 23443 | Degree of Unsaturation | No | No |
| **220** |  | 23444 | Omega-3 Fatty Acids | No | No |
| **220** |  | 23445 | Omega-6 Fatty Acids | No | No |
| **220** |  | 23446 | Polyunsaturated Fatty Acids | No | No |
| **220** |  | 23447 | Monounsaturated Fatty Acids | No | No |
| **220** |  | 23448 | Saturated Fatty Acids | No | No |
| **220** |  | 23449 | Linoleic Acid | No | No |
| **220** |  | 23450 | Docosahexaenoic Acid | No | No |
| **220** |  | 23451 | Omega-3 Fatty Acids to Total Fatty Acids percentage | No | No |
| **220** |  | 23452 | Omega-6 Fatty Acids to Total Fatty Acids percentage | No | No |
| **220** |  | 23453 | Polyunsaturated Fatty Acids to Total Fatty Acids percentage | No | No |
| **220** |  | 23454 | Monounsaturated Fatty Acids to Total Fatty Acids percentage | No | No |
| **220** |  | 23455 | Saturated Fatty Acids to Total Fatty Acids percentage | No | No |
| **220** |  | 23456 | Linoleic Acid to Total Fatty Acids percentage | No | No |
| **220** |  | 23457 | Docosahexaenoic Acid to Total Fatty Acids percentage | No | No |
| **220** |  | 23458 | Polyunsaturated Fatty Acids to Monounsaturated Fatty Acids ratio | No | No |
| **220** |  | 23459 | Omega-6 Fatty Acids to Omega-3 Fatty Acids ratio | No | No |
| **220** |  | 23460 | Alanine | No | No |
| **220** |  | 23461 | Glutamine | No | No |
| **220** |  | 23462 | Glycine | No | No |
| **220** |  | 23463 | Histidine | No | No |
| **220** |  | 23464 | Total Concentration of Branched-Chain Amino Acids (Leucine + Isoleucine + Valine) | No | No |
| **220** |  | 23465 | Isoleucine | No | No |
| **220** |  | 23466 | Leucine | No | No |
| **220** |  | 23467 | Valine | No | No |
| **220** |  | 23468 | Phenylalanine | No | No |
| **220** |  | 23469 | Tyrosine | No | No |
| **220** |  | 23470 | Glucose | No | No |
| **220** |  | 23471 | Lactate | No | No |
| **220** |  | 23472 | Pyruvate | No | No |
| **220** |  | 23473 | Citrate | No | No |
| **220** |  | 23474 | 3-Hydroxybutyrate | No | No |
| **220** |  | 23475 | Acetate | No | No |
| **220** |  | 23476 | Acetoacetate | No | No |
| **220** |  | 23477 | Acetone | No | No |
| **220** |  | 23478 | Creatinine | No | No |
| **220** |  | 23479 | Albumin | No | No |
| **220** |  | 23480 | Glycoprotein Acetyls | No | No |
| **220** |  | 23481 | Concentration of Chylomicrons and Extremely Large VLDL Particles | No | No |
| **220** |  | 23482 | Total Lipids in Chylomicrons and Extremely Large VLDL | No | No |
| **220** |  | 23483 | Phospholipids in Chylomicrons and Extremely Large VLDL | No | No |
| **220** |  | 23484 | Cholesterol in Chylomicrons and Extremely Large VLDL | No | No |
| **220** |  | 23485 | Cholesteryl Esters in Chylomicrons and Extremely Large VLDL | No | No |
| **220** |  | 23486 | Free Cholesterol in Chylomicrons and Extremely Large VLDL | No | No |
| **220** |  | 23487 | Triglycerides in Chylomicrons and Extremely Large VLDL | No | No |
| **220** |  | 23488 | Concentration of Very Large VLDL Particles | No | No |
| **220** |  | 23489 | Total Lipids in Very Large VLDL | No | No |
| **220** |  | 23490 | Phospholipids in Very Large VLDL | No | No |
| **220** |  | 23491 | Cholesterol in Very Large VLDL | No | No |
| **220** |  | 23492 | Cholesteryl Esters in Very Large VLDL | No | No |
| **220** |  | 23493 | Free Cholesterol in Very Large VLDL | No | No |
| **220** |  | 23494 | Triglycerides in Very Large VLDL | No | No |
| **220** |  | 23495 | Concentration of Large VLDL Particles | No | No |
| **220** |  | 23496 | Total Lipids in Large VLDL | No | No |
| **220** |  | 23497 | Phospholipids in Large VLDL | No | No |
| **220** |  | 23498 | Cholesterol in Large VLDL | No | No |
| **220** |  | 23499 | Cholesteryl Esters in Large VLDL | No | No |
| **220** |  | 23500 | Free Cholesterol in Large VLDL | No | No |
| **220** |  | 23501 | Triglycerides in Large VLDL | No | No |
| **220** |  | 23502 | Concentration of Medium VLDL Particles | No | No |
| **220** |  | 23503 | Total Lipids in Medium VLDL | No | No |
| **220** |  | 23504 | Phospholipids in Medium VLDL | No | No |
| **220** |  | 23505 | Cholesterol in Medium VLDL | No | No |
| **220** |  | 23506 | Cholesteryl Esters in Medium VLDL | No | No |
| **220** |  | 23507 | Free Cholesterol in Medium VLDL | No | No |
| **220** |  | 23508 | Triglycerides in Medium VLDL | No | No |
| **220** |  | 23509 | Concentration of Small VLDL Particles | No | No |
| **220** |  | 23510 | Total Lipids in Small VLDL | No | No |
| **220** |  | 23511 | Phospholipids in Small VLDL | No | No |
| **220** |  | 23512 | Cholesterol in Small VLDL | No | No |
| **220** |  | 23513 | Cholesteryl Esters in Small VLDL | No | No |
| **220** |  | 23514 | Free Cholesterol in Small VLDL | No | No |
| **220** |  | 23515 | Triglycerides in Small VLDL | No | No |
| **220** |  | 23516 | Concentration of Very Small VLDL Particles | No | No |
| **220** |  | 23517 | Total Lipids in Very Small VLDL | No | No |
| **220** |  | 23518 | Phospholipids in Very Small VLDL | No | No |
| **220** |  | 23519 | Cholesterol in Very Small VLDL | No | No |
| **220** |  | 23520 | Cholesteryl Esters in Very Small VLDL | No | No |
| **220** |  | 23521 | Free Cholesterol in Very Small VLDL | No | No |
| **220** |  | 23522 | Triglycerides in Very Small VLDL | No | No |
| **220** |  | 23523 | Concentration of IDL Particles | No | No |
| **220** |  | 23524 | Total Lipids in IDL | No | No |
| **220** |  | 23525 | Phospholipids in IDL | No | No |
| **220** |  | 23526 | Cholesterol in IDL | No | No |
| **220** |  | 23527 | Cholesteryl Esters in IDL | No | No |
| **220** |  | 23528 | Free Cholesterol in IDL | No | No |
| **220** |  | 23529 | Triglycerides in IDL | No | No |
| **220** |  | 23530 | Concentration of Large LDL Particles | No | No |
| **220** |  | 23531 | Total Lipids in Large LDL | No | No |
| **220** |  | 23532 | Phospholipids in Large LDL | No | No |
| **220** |  | 23533 | Cholesterol in Large LDL | No | No |
| **220** |  | 23534 | Cholesteryl Esters in Large LDL | No | No |
| **220** |  | 23535 | Free Cholesterol in Large LDL | No | No |
| **220** |  | 23536 | Triglycerides in Large LDL | No | No |
| **220** |  | 23537 | Concentration of Medium LDL Particles | No | No |
| **220** |  | 23538 | Total Lipids in Medium LDL | No | No |
| **220** |  | 23539 | Phospholipids in Medium LDL | No | No |
| **220** |  | 23540 | Cholesterol in Medium LDL | No | No |
| **220** |  | 23541 | Cholesteryl Esters in Medium LDL | No | No |
| **220** |  | 23542 | Free Cholesterol in Medium LDL | No | No |
| **220** |  | 23543 | Triglycerides in Medium LDL | No | No |
| **220** |  | 23544 | Concentration of Small LDL Particles | No | No |
| **220** |  | 23545 | Total Lipids in Small LDL | No | No |
| **220** |  | 23546 | Phospholipids in Small LDL | No | No |
| **220** |  | 23547 | Cholesterol in Small LDL | No | No |
| **220** |  | 23548 | Cholesteryl Esters in Small LDL | No | No |
| **220** |  | 23549 | Free Cholesterol in Small LDL | No | No |
| **220** |  | 23550 | Triglycerides in Small LDL | No | No |
| **220** |  | 23551 | Concentration of Very Large HDL Particles | No | No |
| **220** |  | 23552 | Total Lipids in Very Large HDL | No | No |
| **220** |  | 23553 | Phospholipids in Very Large HDL | No | No |
| **220** |  | 23554 | Cholesterol in Very Large HDL | No | No |
| **220** |  | 23555 | Cholesteryl Esters in Very Large HDL | No | No |
| **220** |  | 23556 | Free Cholesterol in Very Large HDL | No | No |
| **220** |  | 23557 | Triglycerides in Very Large HDL | No | No |
| **220** |  | 23558 | Concentration of Large HDL Particles | No | No |
| **220** |  | 23559 | Total Lipids in Large HDL | No | No |
| **220** |  | 23560 | Phospholipids in Large HDL | No | No |
| **220** |  | 23561 | Cholesterol in Large HDL | No | No |
| **220** |  | 23562 | Cholesteryl Esters in Large HDL | No | No |
| **220** |  | 23563 | Free Cholesterol in Large HDL | No | No |
| **220** |  | 23564 | Triglycerides in Large HDL | No | No |
| **220** |  | 23565 | Concentration of Medium HDL Particles | No | No |
| **220** |  | 23566 | Total Lipids in Medium HDL | No | No |
| **220** |  | 23567 | Phospholipids in Medium HDL | No | No |
| **220** |  | 23568 | Cholesterol in Medium HDL | No | No |
| **220** |  | 23569 | Cholesteryl Esters in Medium HDL | No | No |
| **220** |  | 23570 | Free Cholesterol in Medium HDL | No | No |
| **220** |  | 23571 | Triglycerides in Medium HDL | No | No |
| **220** |  | 23572 | Concentration of Small HDL Particles | No | No |
| **220** |  | 23573 | Total Lipids in Small HDL | No | No |
| **220** |  | 23574 | Phospholipids in Small HDL | No | No |
| **220** |  | 23575 | Cholesterol in Small HDL | No | No |
| **220** |  | 23576 | Cholesteryl Esters in Small HDL | No | No |
| **220** |  | 23577 | Free Cholesterol in Small HDL | No | No |
| **220** |  | 23578 | Triglycerides in Small HDL | No | No |
| **220** |  | 23579 | Phospholipids to Total Lipids in Chylomicrons and Extremely Large VLDL percentage | No | No |
| **220** |  | 23580 | Cholesterol to Total Lipids in Chylomicrons and Extremely Large VLDL percentage | No | No |
| **220** |  | 23581 | Cholesteryl Esters to Total Lipids in Chylomicrons and Extremely Large VLDL percentage | No | No |
| **220** |  | 23582 | Free Cholesterol to Total Lipids in Chylomicrons and Extremely Large VLDL percentage | No | No |
| **220** |  | 23583 | Triglycerides to Total Lipids in Chylomicrons and Extremely Large VLDL percentage | No | No |
| **220** |  | 23584 | Phospholipids to Total Lipids in Very Large VLDL percentage | No | No |
| **220** |  | 23585 | Cholesterol to Total Lipids in Very Large VLDL percentage | No | No |
| **220** |  | 23586 | Cholesteryl Esters to Total Lipids in Very Large VLDL percentage | No | No |
| **220** |  | 23587 | Free Cholesterol to Total Lipids in Very Large VLDL percentage | No | No |
| **220** |  | 23588 | Triglycerides to Total Lipids in Very Large VLDL percentage | No | No |
| **220** |  | 23589 | Phospholipids to Total Lipids in Large VLDL percentage | No | No |
| **220** |  | 23590 | Cholesterol to Total Lipids in Large VLDL percentage | No | No |
| **220** |  | 23591 | Cholesteryl Esters to Total Lipids in Large VLDL percentage | No | No |
| **220** |  | 23592 | Free Cholesterol to Total Lipids in Large VLDL percentage | No | No |
| **220** |  | 23593 | Triglycerides to Total Lipids in Large VLDL percentage | No | No |
| **220** |  | 23594 | Phospholipids to Total Lipids in Medium VLDL percentage | No | No |
| **220** |  | 23595 | Cholesterol to Total Lipids in Medium VLDL percentage | No | No |
| **220** |  | 23596 | Cholesteryl Esters to Total Lipids in Medium VLDL percentage | No | No |
| **220** |  | 23597 | Free Cholesterol to Total Lipids in Medium VLDL percentage | No | No |
| **220** |  | 23598 | Triglycerides to Total Lipids in Medium VLDL percentage | No | No |
| **220** |  | 23599 | Phospholipids to Total Lipids in Small VLDL percentage | No | No |
| **220** |  | 23600 | Cholesterol to Total Lipids in Small VLDL percentage | No | No |
| **220** |  | 23601 | Cholesteryl Esters to Total Lipids in Small VLDL percentage | No | No |
| **220** |  | 23602 | Free Cholesterol to Total Lipids in Small VLDL percentage | No | No |
| **220** |  | 23603 | Triglycerides to Total Lipids in Small VLDL percentage | No | No |
| **220** |  | 23604 | Phospholipids to Total Lipids in Very Small VLDL percentage | No | No |
| **220** |  | 23605 | Cholesterol to Total Lipids in Very Small VLDL percentage | No | No |
| **220** |  | 23606 | Cholesteryl Esters to Total Lipids in Very Small VLDL percentage | No | No |
| **220** |  | 23607 | Free Cholesterol to Total Lipids in Very Small VLDL percentage | No | No |
| **220** |  | 23608 | Triglycerides to Total Lipids in Very Small VLDL percentage | No | No |
| **220** |  | 23609 | Phospholipids to Total Lipids in IDL percentage | No | No |
| **220** |  | 23610 | Cholesterol to Total Lipids in IDL percentage | No | No |
| **220** |  | 23611 | Cholesteryl Esters to Total Lipids in IDL percentage | No | No |
| **220** |  | 23612 | Free Cholesterol to Total Lipids in IDL percentage | No | No |
| **220** |  | 23613 | Triglycerides to Total Lipids in IDL percentage | No | No |
| **220** |  | 23614 | Phospholipids to Total Lipids in Large LDL percentage | No | No |
| **220** |  | 23615 | Cholesterol to Total Lipids in Large LDL percentage | No | No |
| **220** |  | 23616 | Cholesteryl Esters to Total Lipids in Large LDL percentage | No | No |
| **220** |  | 23617 | Free Cholesterol to Total Lipids in Large LDL percentage | No | No |
| **220** |  | 23618 | Triglycerides to Total Lipids in Large LDL percentage | No | No |
| **220** |  | 23619 | Phospholipids to Total Lipids in Medium LDL percentage | No | No |
| **220** |  | 23620 | Cholesterol to Total Lipids in Medium LDL percentage | No | No |
| **220** |  | 23621 | Cholesteryl Esters to Total Lipids in Medium LDL percentage | No | No |
| **220** |  | 23622 | Free Cholesterol to Total Lipids in Medium LDL percentage | No | No |
| **220** |  | 23623 | Triglycerides to Total Lipids in Medium LDL percentage | No | No |
| **220** |  | 23624 | Phospholipids to Total Lipids in Small LDL percentage | No | No |
| **220** |  | 23625 | Cholesterol to Total Lipids in Small LDL percentage | No | No |
| **220** |  | 23626 | Cholesteryl Esters to Total Lipids in Small LDL percentage | No | No |
| **220** |  | 23627 | Free Cholesterol to Total Lipids in Small LDL percentage | No | No |
| **220** |  | 23628 | Triglycerides to Total Lipids in Small LDL percentage | No | No |
| **220** |  | 23629 | Phospholipids to Total Lipids in Very Large HDL percentage | No | No |
| **220** |  | 23630 | Cholesterol to Total Lipids in Very Large HDL percentage | No | No |
| **220** |  | 23631 | Cholesteryl Esters to Total Lipids in Very Large HDL percentage | No | No |
| **220** |  | 23632 | Free Cholesterol to Total Lipids in Very Large HDL percentage | No | No |
| **220** |  | 23633 | Triglycerides to Total Lipids in Very Large HDL percentage | No | No |
| **220** |  | 23634 | Phospholipids to Total Lipids in Large HDL percentage | No | No |
| **220** |  | 23635 | Cholesterol to Total Lipids in Large HDL percentage | No | No |
| **220** |  | 23636 | Cholesteryl Esters to Total Lipids in Large HDL percentage | No | No |
| **220** |  | 23637 | Free Cholesterol to Total Lipids in Large HDL percentage | No | No |
| **220** |  | 23638 | Triglycerides to Total Lipids in Large HDL percentage | No | No |
| **220** |  | 23639 | Phospholipids to Total Lipids in Medium HDL percentage | No | No |
| **220** |  | 23640 | Cholesterol to Total Lipids in Medium HDL percentage | No | No |
| **220** |  | 23641 | Cholesteryl Esters to Total Lipids in Medium HDL percentage | No | No |
| **220** |  | 23642 | Free Cholesterol to Total Lipids in Medium HDL percentage | No | No |
| **220** |  | 23643 | Triglycerides to Total Lipids in Medium HDL percentage | No | No |
| **220** |  | 23644 | Phospholipids to Total Lipids in Small HDL percentage | No | No |
| **220** |  | 23645 | Cholesterol to Total Lipids in Small HDL percentage | No | No |
| **220** |  | 23646 | Cholesteryl Esters to Total Lipids in Small HDL percentage | No | No |
| **220** |  | 23647 | Free Cholesterol to Total Lipids in Small HDL percentage | No | No |
| **220** |  | 23648 | Triglycerides to Total Lipids in Small HDL percentage | No | No |
| **222** |  | 23649 | Shipment Plate | No | No |
| **222** |  | 23650 | Spectrometer | No | No |
| **222** |  | 23652 | High Lactate | No | No |
| **222** |  | 23653 | High Pyruvate | No | No |
| **222** |  | 23654 | Low Glucose | No | No |
| **222** |  | 23655 | Low Protein | No | No |
| **222** |  | 23658 | Sample Measured Date and Time | No | No |
| **222** |  | 23659 | Sample Prepared Date and Time | No | No |
| **222** |  | 23660 | Well position within plate | No | No |
| **221** |  | 23704 | Clinical LDL Cholesterol, QC Flag | No | No |
| **221** |  | 23705 | LDL Cholesterol, QC Flag | No | No |
| **221** |  | 23717 | Cholesteryl Esters in LDL, QC Flag | No | No |
| **221** |  | 23721 | Free Cholesterol in LDL, QC Flag | No | No |
| **221** |  | 23734 | Phosphoglycerides, QC Flag | No | No |
| **221** |  | 23735 | Triglycerides to Phosphoglycerides ratio, QC Flag | No | No |
| **221** |  | 23736 | Total Cholines, QC Flag | No | No |
| **221** |  | 23737 | Phosphatidylcholines, QC Flag | No | No |
| **221** |  | 23738 | Sphingomyelins, QC Flag | No | No |
| **221** |  | 23742 | Total Fatty Acids, QC Flag | No | No |
| **221** |  | 23743 | Degree of Unsaturation, QC Flag | No | No |
| **221** |  | 23744 | Omega-3 Fatty Acids, QC Flag | No | No |
| **221** |  | 23745 | Omega-6 Fatty Acids, QC Flag | No | No |
| **221** |  | 23746 | Polyunsaturated Fatty Acids, QC Flag | No | No |
| **221** |  | 23747 | Monounsaturated Fatty Acids, QC Flag | No | No |
| **221** |  | 23748 | Saturated Fatty Acids, QC Flag | No | No |
| **221** |  | 23749 | Linoleic Acid, QC Flag | No | No |
| **221** |  | 23750 | Docosahexaenoic Acid, QC Flag | No | No |
| **221** |  | 23751 | Omega-3 Fatty Acids to Total Fatty Acids percentage, QC Flag | No | No |
| **221** |  | 23752 | Omega-6 Fatty Acids to Total Fatty Acids percentage, QC Flag | No | No |
| **221** |  | 23753 | Polyunsaturated Fatty Acids to Total Fatty Acids percentage, QC Flag | No | No |
| **221** |  | 23754 | Monounsaturated Fatty Acids to Total Fatty Acids percentage, QC Flag | No | No |
| **221** |  | 23755 | Saturated Fatty Acids to Total Fatty Acids percentage, QC Flag | No | No |
| **221** |  | 23756 | Linoleic Acid to Total Fatty Acids percentage, QC Flag | No | No |
| **221** |  | 23757 | Docosahexaenoic Acid to Total Fatty Acids percentage, QC Flag | No | No |
| **221** |  | 23758 | Polyunsaturated Fatty Acids to Monounsaturated Fatty Acids ratio, QC Flag | No | No |
| **221** |  | 23759 | Omega-6 Fatty Acids to Omega-3 Fatty Acids ratio, QC Flag | No | No |
| **221** |  | 23760 | Alanine, QC Flag | No | No |
| **221** |  | 23761 | Glutamine, QC Flag | No | No |
| **221** |  | 23762 | Glycine, QC Flag | No | No |
| **221** |  | 23763 | Histidine, QC Flag | No | No |
| **221** |  | 23764 | Total Concentration of Branched-Chain Amino Acids (Leucine + Isoleucine + Valine), QC Flag | No | No |
| **221** |  | 23766 | Leucine, QC Flag | No | No |
| **221** |  | 23767 | Valine, QC Flag | No | No |
| **221** |  | 23768 | Phenylalanine, QC Flag | No | No |
| **221** |  | 23769 | Tyrosine, QC Flag | No | No |
| **221** |  | 23770 | Glucose, QC Flag | No | No |
| **221** |  | 23772 | Pyruvate, QC Flag | No | No |
| **221** |  | 23773 | Citrate, QC Flag | No | No |
| **221** |  | 23774 | 3-Hydroxybutyrate, QC Flag | No | No |
| **221** |  | 23775 | Acetate, QC Flag | No | No |
| **221** |  | 23776 | Acetoacetate, QC Flag | No | No |
| **221** |  | 23778 | Creatinine, QC Flag | No | No |
| **221** |  | 23782 | Total Lipids in Chylomicrons and Extremely Large VLDL, QC Flag | No | No |
| **221** |  | 23783 | Phospholipids in Chylomicrons and Extremely Large VLDL, QC Flag | No | No |
| **221** |  | 23784 | Cholesterol in Chylomicrons and Extremely Large VLDL, QC Flag | No | No |
| **221** |  | 23785 | Cholesteryl Esters in Chylomicrons and Extremely Large VLDL, QC Flag | No | No |
| **221** |  | 23786 | Free Cholesterol in Chylomicrons and Extremely Large VLDL, QC Flag | No | No |
| **221** |  | 23787 | Triglycerides in Chylomicrons and Extremely Large VLDL, QC Flag | No | No |
| **221** |  | 23789 | Total Lipids in Very Large VLDL, QC Flag | No | No |
| **221** |  | 23790 | Phospholipids in Very Large VLDL, QC Flag | No | No |
| **221** |  | 23791 | Cholesterol in Very Large VLDL, QC Flag | No | No |
| **221** |  | 23792 | Cholesteryl Esters in Very Large VLDL, QC Flag | No | No |
| **221** |  | 23793 | Free Cholesterol in Very Large VLDL, QC Flag | No | No |
| **221** |  | 23794 | Triglycerides in Very Large VLDL, QC Flag | No | No |
| **221** |  | 23796 | Total Lipids in Large VLDL, QC Flag | No | No |
| **221** |  | 23797 | Phospholipids in Large VLDL, QC Flag | No | No |
| **221** |  | 23798 | Cholesterol in Large VLDL, QC Flag | No | No |
| **221** |  | 23799 | Cholesteryl Esters in Large VLDL, QC Flag | No | No |
| **221** |  | 23800 | Free Cholesterol in Large VLDL, QC Flag | No | No |
| **221** |  | 23801 | Triglycerides in Large VLDL, QC Flag | No | No |
| **221** |  | 23804 | Phospholipids in Medium VLDL, QC Flag | No | No |
| **221** |  | 23805 | Cholesterol in Medium VLDL, QC Flag | No | No |
| **221** |  | 23806 | Cholesteryl Esters in Medium VLDL, QC Flag | No | No |
| **221** |  | 23807 | Free Cholesterol in Medium VLDL, QC Flag | No | No |
| **221** |  | 23808 | Triglycerides in Medium VLDL, QC Flag | No | No |
| **221** |  | 23813 | Cholesteryl Esters in Small VLDL, QC Flag | No | No |
| **221** |  | 23814 | Free Cholesterol in Small VLDL, QC Flag | No | No |
| **221** |  | 23833 | Cholesterol in Large LDL, QC Flag | No | No |
| **221** |  | 23834 | Cholesteryl Esters in Large LDL, QC Flag | No | No |
| **221** |  | 23835 | Free Cholesterol in Large LDL, QC Flag | No | No |
| **221** |  | 23840 | Cholesterol in Medium LDL, QC Flag | No | No |
| **221** |  | 23841 | Cholesteryl Esters in Medium LDL, QC Flag | No | No |
| **221** |  | 23842 | Free Cholesterol in Medium LDL, QC Flag | No | No |
| **221** |  | 23843 | Triglycerides in Medium LDL, QC Flag | No | No |
| **221** |  | 23847 | Cholesterol in Small LDL, QC Flag | No | No |
| **221** |  | 23848 | Cholesteryl Esters in Small LDL, QC Flag | No | No |
| **221** |  | 23849 | Free Cholesterol in Small LDL, QC Flag | No | No |
| **221** |  | 23850 | Triglycerides in Small LDL, QC Flag | No | No |
| **221** |  | 23852 | Total Lipids in Very Large HDL, QC Flag | No | No |
| **221** |  | 23853 | Phospholipids in Very Large HDL, QC Flag | No | No |
| **221** |  | 23854 | Cholesterol in Very Large HDL, QC Flag | No | No |
| **221** |  | 23855 | Cholesteryl Esters in Very Large HDL, QC Flag | No | No |
| **221** |  | 23856 | Free Cholesterol in Very Large HDL, QC Flag | No | No |
| **221** |  | 23857 | Triglycerides in Very Large HDL, QC Flag | No | No |
| **221** |  | 23860 | Phospholipids in Large HDL, QC Flag | No | No |
| **221** |  | 23861 | Cholesterol in Large HDL, QC Flag | No | No |
| **221** |  | 23862 | Cholesteryl Esters in Large HDL, QC Flag | No | No |
| **221** |  | 23863 | Free Cholesterol in Large HDL, QC Flag | No | No |
| **221** |  | 23864 | Triglycerides in Large HDL, QC Flag | No | No |
| **221** |  | 23867 | Phospholipids in Medium HDL, QC Flag | No | No |
| **221** |  | 23868 | Cholesterol in Medium HDL, QC Flag | No | No |
| **221** |  | 23869 | Cholesteryl Esters in Medium HDL, QC Flag | No | No |
| **221** |  | 23870 | Free Cholesterol in Medium HDL, QC Flag | No | No |
| **221** |  | 23871 | Triglycerides in Medium HDL, QC Flag | No | No |
| **221** |  | 23876 | Cholesteryl Esters in Small HDL, QC Flag | No | No |
| **221** |  | 23878 | Triglycerides in Small HDL, QC Flag | No | No |
| **221** |  | 23879 | Phospholipids to Total Lipids in Chylomicrons and Extremely Large VLDL percentage, QC Flag | No | No |
| **221** |  | 23880 | Cholesterol to Total Lipids in Chylomicrons and Extremely Large VLDL percentage, QC Flag | No | No |
| **221** |  | 23881 | Cholesteryl Esters to Total Lipids in Chylomicrons and Extremely Large VLDL percentage, QC Flag | No | No |
| **221** |  | 23882 | Free Cholesterol to Total Lipids in Chylomicrons and Extremely Large VLDL percentage, QC Flag | No | No |
| **221** |  | 23883 | Triglycerides to Total Lipids in Chylomicrons and Extremely Large VLDL percentage, QC Flag | No | No |
| **221** |  | 23884 | Phospholipids to Total Lipids in Very Large VLDL percentage, QC Flag | No | No |
| **221** |  | 23885 | Cholesterol to Total Lipids in Very Large VLDL percentage, QC Flag | No | No |
| **221** |  | 23886 | Cholesteryl Esters to Total Lipids in Very Large VLDL percentage, QC Flag | No | No |
| **221** |  | 23887 | Free Cholesterol to Total Lipids in Very Large VLDL percentage, QC Flag | No | No |
| **221** |  | 23888 | Triglycerides to Total Lipids in Very Large VLDL percentage, QC Flag | No | No |
| **221** |  | 23889 | Phospholipids to Total Lipids in Large VLDL percentage, QC Flag | No | No |
| **221** |  | 23890 | Cholesterol to Total Lipids in Large VLDL percentage, QC Flag | No | No |
| **221** |  | 23891 | Cholesteryl Esters to Total Lipids in Large VLDL percentage, QC Flag | No | No |
| **221** |  | 23892 | Free Cholesterol to Total Lipids in Large VLDL percentage, QC Flag | No | No |
| **221** |  | 23893 | Triglycerides to Total Lipids in Large VLDL percentage, QC Flag | No | No |
| **221** |  | 23894 | Phospholipids to Total Lipids in Medium VLDL percentage, QC Flag | No | No |
| **221** |  | 23895 | Cholesterol to Total Lipids in Medium VLDL percentage, QC Flag | No | No |
| **221** |  | 23896 | Cholesteryl Esters to Total Lipids in Medium VLDL percentage, QC Flag | No | No |
| **221** |  | 23897 | Free Cholesterol to Total Lipids in Medium VLDL percentage, QC Flag | No | No |
| **221** |  | 23898 | Triglycerides to Total Lipids in Medium VLDL percentage, QC Flag | No | No |
| **221** |  | 23901 | Cholesteryl Esters to Total Lipids in Small VLDL percentage, QC Flag | No | No |
| **221** |  | 23902 | Free Cholesterol to Total Lipids in Small VLDL percentage, QC Flag | No | No |
| **221** |  | 23915 | Cholesterol to Total Lipids in Large LDL percentage, QC Flag | No | No |
| **221** |  | 23916 | Cholesteryl Esters to Total Lipids in Large LDL percentage, QC Flag | No | No |
| **221** |  | 23917 | Free Cholesterol to Total Lipids in Large LDL percentage, QC Flag | No | No |
| **221** |  | 23920 | Cholesterol to Total Lipids in Medium LDL percentage, QC Flag | No | No |
| **221** |  | 23921 | Cholesteryl Esters to Total Lipids in Medium LDL percentage, QC Flag | No | No |
| **221** |  | 23922 | Free Cholesterol to Total Lipids in Medium LDL percentage, QC Flag | No | No |
| **221** |  | 23923 | Triglycerides to Total Lipids in Medium LDL percentage, QC Flag | No | No |
| **221** |  | 23925 | Cholesterol to Total Lipids in Small LDL percentage, QC Flag | No | No |
| **221** |  | 23926 | Cholesteryl Esters to Total Lipids in Small LDL percentage, QC Flag | No | No |
| **221** |  | 23927 | Free Cholesterol to Total Lipids in Small LDL percentage, QC Flag | No | No |
| **221** |  | 23928 | Triglycerides to Total Lipids in Small LDL percentage, QC Flag | No | No |
| **221** |  | 23929 | Phospholipids to Total Lipids in Very Large HDL percentage, QC Flag | No | No |
| **221** |  | 23930 | Cholesterol to Total Lipids in Very Large HDL percentage, QC Flag | No | No |
| **221** |  | 23931 | Cholesteryl Esters to Total Lipids in Very Large HDL percentage, QC Flag | No | No |
| **221** |  | 23932 | Free Cholesterol to Total Lipids in Very Large HDL percentage, QC Flag | No | No |
| **221** |  | 23933 | Triglycerides to Total Lipids in Very Large HDL percentage, QC Flag | No | No |
| **221** |  | 23934 | Phospholipids to Total Lipids in Large HDL percentage, QC Flag | No | No |
| **221** |  | 23935 | Cholesterol to Total Lipids in Large HDL percentage, QC Flag | No | No |
| **221** |  | 23936 | Cholesteryl Esters to Total Lipids in Large HDL percentage, QC Flag | No | No |
| **221** |  | 23937 | Free Cholesterol to Total Lipids in Large HDL percentage, QC Flag | No | No |
| **221** |  | 23938 | Triglycerides to Total Lipids in Large HDL percentage, QC Flag | No | No |
| **221** |  | 23939 | Phospholipids to Total Lipids in Medium HDL percentage, QC Flag | No | No |
| **221** |  | 23940 | Cholesterol to Total Lipids in Medium HDL percentage, QC Flag | No | No |
| **221** |  | 23941 | Cholesteryl Esters to Total Lipids in Medium HDL percentage, QC Flag | No | No |
| **221** |  | 23942 | Free Cholesterol to Total Lipids in Medium HDL percentage, QC Flag | No | No |
| **221** |  | 23943 | Triglycerides to Total Lipids in Medium HDL percentage, QC Flag | No | No |
| **221** |  | 23946 | Cholesteryl Esters to Total Lipids in Small HDL percentage, QC Flag | No | No |
| **221** |  | 23948 | Triglycerides to Total Lipids in Small HDL percentage, QC Flag | No | No |
| **114** | Residential air pollution | 24003 | Nitrogen dioxide air pollution; 2010 | No | No |
| **114** | Residential air pollution | 24004 | Nitrogen oxides air pollution; 2010 | No | No |
| **114** | Residential air pollution | 24005 | Particulate matter air pollution (pm10); 2010 | No | No |
| **114** | Residential air pollution | 24006 | Particulate matter air pollution (pm2.5); 2010 | No | No |
| **114** | Residential air pollution | 24007 | Particulate matter air pollution (pm2.5) absorbance; 2010 | No | No |
| **114** | Residential air pollution | 24008 | Particulate matter air pollution 2.5-10um; 2010 | No | No |
| **114** | Residential air pollution | 24009 | Traffic intensity on the nearest road | No | No |
| **114** | Residential air pollution | 24010 | Inverse distance to the nearest road | No | No |
| **114** | Residential air pollution | 24011 | Traffic intensity on the nearest major road | No | No |
| **114** | Residential air pollution | 24012 | Inverse distance to the nearest major road | No | No |
| **114** | Residential air pollution | 24013 | Total traffic load on major roads | No | No |
| **114** | Residential air pollution | 24014 | Close to major road | No | No |
| **114** | Residential air pollution | 24015 | Sum of road length of major roads within 100m | No | No |
| **114** | Residential air pollution | 24016 | Nitrogen dioxide air pollution; 2005 | No | No |
| **114** | Residential air pollution | 24017 | Nitrogen dioxide air pollution; 2006 | No | No |
| **114** | Residential air pollution | 24018 | Nitrogen dioxide air pollution; 2007 | No | No |
| **114** | Residential air pollution | 24019 | Particulate matter air pollution (pm10); 2007 | No | No |
| **115** | Residential noise pollution | 24020 | Average daytime sound level of noise pollution | No | No |
| **115** | Residential noise pollution | 24021 | Average evening sound level of noise pollution | No | No |
| **115** | Residential noise pollution | 24022 | Average night-time sound level of noise pollution | No | No |
| **115** | Residential noise pollution | 24023 | Average 16-hour sound level of noise pollution | No | No |
| **115** | Residential noise pollution | 24024 | Average 24-hour sound level of noise pollution | No | No |
| **113** | Local environment | 24025 | Included in pollution study | No | No |
| **149** | Abdominal composition | 24352 | FR liver PDFF mean | No | No |
| **149** | Abdominal composition | 24353 | Anterior thigh muscle fat infiltration (MFI) (left) | No | No |
| **149** | Abdominal composition | 24354 | Anterior thigh muscle fat infiltration (MFI) (right) | No | No |
| **151** | Greenspace and coastal proximity | 24500 | Greenspace percentage, buffer 1000m | No | No |
| **151** | Greenspace and coastal proximity | 24501 | Domestic garden percentage, buffer 1000m | No | No |
| **151** | Greenspace and coastal proximity | 24502 | Water percentage, buffer 1000m | No | No |
| **151** | Greenspace and coastal proximity | 24503 | Greenspace percentage, buffer 300m | No | No |
| **151** | Greenspace and coastal proximity | 24504 | Domestic garden percentage, buffer 300m | No | No |
| **151** | Greenspace and coastal proximity | 24505 | Water percentage, buffer 300m | No | No |
| **151** | Greenspace and coastal proximity | 24506 | Natural environment percentage, buffer 1000m | No | No |
| **151** | Greenspace and coastal proximity | 24507 | Natural environment percentage, buffer 300m | No | No |
| **151** | Greenspace and coastal proximity | 24508 | Distance (Euclidean) to coast | No | No |
| **110** | T1 structural brain MRI | 25000 | Volumetric scaling from T1 head image to standard space | No | No |
| **110** | T1 structural brain MRI | 25001 | Volume of peripheral cortical grey matter (normalised for head size) | No | No |
| **110** | T1 structural brain MRI | 25002 | Volume of peripheral cortical grey matter | No | No |
| **110** | T1 structural brain MRI | 25003 | Volume of ventricular cerebrospinal fluid (normalised for head size) | No | No |
| **110** | T1 structural brain MRI | 25004 | Volume of ventricular cerebrospinal fluid | No | No |
| **110** | T1 structural brain MRI | 25005 | Volume of grey matter (normalised for head size) | No | No |
| **110** | T1 structural brain MRI | 25006 | Volume of grey matter | No | No |
| **110** | T1 structural brain MRI | 25007 | Volume of white matter (normalised for head size) | No | No |
| **110** | T1 structural brain MRI | 25008 | Volume of white matter | No | No |
| **110** | T1 structural brain MRI | 25009 | Volume of brain, grey+white matter (normalised for head size) | No | No |
| **110** | T1 structural brain MRI | 25010 | Volume of brain, grey+white matter | No | No |
| **1102** | Subcortical volumes (FIRST) | 25011 | Volume of thalamus (left) | No | No |
| **1102** | Subcortical volumes (FIRST) | 25012 | Volume of thalamus (right) | No | No |
| **1102** | Subcortical volumes (FIRST) | 25013 | Volume of caudate (left) | No | No |
| **1102** | Subcortical volumes (FIRST) | 25014 | Volume of caudate (right) | No | No |
| **1102** | Subcortical volumes (FIRST) | 25015 | Volume of putamen (left) | No | No |
| **1102** | Subcortical volumes (FIRST) | 25016 | Volume of putamen (right) | No | No |
| **1102** | Subcortical volumes (FIRST) | 25017 | Volume of pallidum (left) | No | No |
| **1102** | Subcortical volumes (FIRST) | 25018 | Volume of pallidum (right) | No | No |
| **1102** | Subcortical volumes (FIRST) | 25019 | Volume of hippocampus (left) | No | No |
| **1102** | Subcortical volumes (FIRST) | 25020 | Volume of hippocampus (right) | No | No |
| **1102** | Subcortical volumes (FIRST) | 25021 | Volume of amygdala (left) | No | No |
| **1102** | Subcortical volumes (FIRST) | 25022 | Volume of amygdala (right) | No | No |
| **1102** | Subcortical volumes (FIRST) | 25023 | Volume of accumbens (left) | No | No |
| **1102** | Subcortical volumes (FIRST) | 25024 | Volume of accumbens (right) | No | No |
| **110** | T1 structural brain MRI | 25025 | Volume of brain stem + 4th ventricle | No | No |
| **109** | Susceptibility weighted brain MRI | 25026 | Median T2star in thalamus (left) | No | No |
| **109** | Susceptibility weighted brain MRI | 25027 | Median T2star in thalamus (right) | No | No |
| **109** | Susceptibility weighted brain MRI | 25028 | Median T2star in caudate (left) | No | No |
| **109** | Susceptibility weighted brain MRI | 25029 | Median T2star in caudate (right) | No | No |
| **109** | Susceptibility weighted brain MRI | 25030 | Median T2star in putamen (left) | No | No |
| **109** | Susceptibility weighted brain MRI | 25031 | Median T2star in putamen (right) | No | No |
| **109** | Susceptibility weighted brain MRI | 25032 | Median T2star in pallidum (left) | No | No |
| **109** | Susceptibility weighted brain MRI | 25033 | Median T2star in pallidum (right) | No | No |
| **109** | Susceptibility weighted brain MRI | 25034 | Median T2star in hippocampus (left) | No | No |
| **109** | Susceptibility weighted brain MRI | 25035 | Median T2star in hippocampus (right) | No | No |
| **109** | Susceptibility weighted brain MRI | 25036 | Median T2star in amygdala (left) | No | No |
| **109** | Susceptibility weighted brain MRI | 25037 | Median T2star in amygdala (right) | No | No |
| **109** | Susceptibility weighted brain MRI | 25038 | Median T2star in accumbens (left) | No | No |
| **109** | Susceptibility weighted brain MRI | 25039 | Median T2star in accumbens (right) | No | No |
| **106** | Task functional brain MRI | 25040 | Median BOLD effect (in group-defined mask) for shapes activation | No | No |
| **106** | Task functional brain MRI | 25042 | Median z-statistic (in group-defined mask) for shapes activation | No | No |
| **106** | Task functional brain MRI | 25044 | Median BOLD effect (in group-defined mask) for faces activation | No | No |
| **106** | Task functional brain MRI | 25046 | Median z-statistic (in group-defined mask) for faces activation | No | No |
| **106** | Task functional brain MRI | 25048 | Median BOLD effect (in group-defined mask) for faces-shapes contrast | No | No |
| **106** | Task functional brain MRI | 25050 | Median z-statistic (in group-defined mask) for faces-shapes contrast | No | No |
| **106** | Task functional brain MRI | 25052 | Median BOLD effect (in group-defined amygdala activation mask) for faces-shapes contrast | No | No |
| **106** | Task functional brain MRI | 25054 | Median z-statistic (in group-defined amygdala activation mask) for faces-shapes contrast | No | No |
| **134** | dMRI skeleton | 25056 | Mean FA in middle cerebellar peduncle on FA skeleton | No | No |
| **134** | dMRI skeleton | 25057 | Mean FA in pontine crossing tract on FA skeleton | No | No |
| **134** | dMRI skeleton | 25058 | Mean FA in genu of corpus callosum on FA skeleton | No | No |
| **134** | dMRI skeleton | 25059 | Mean FA in body of corpus callosum on FA skeleton | No | No |
| **134** | dMRI skeleton | 25060 | Mean FA in splenium of corpus callosum on FA skeleton | No | No |
| **134** | dMRI skeleton | 25061 | Mean FA in fornix on FA skeleton | No | No |
| **134** | dMRI skeleton | 25062 | Mean FA in corticospinal tract on FA skeleton (right) | No | No |
| **134** | dMRI skeleton | 25063 | Mean FA in corticospinal tract on FA skeleton (left) | No | No |
| **134** | dMRI skeleton | 25064 | Mean FA in medial lemniscus on FA skeleton (right) | No | No |
| **134** | dMRI skeleton | 25065 | Mean FA in medial lemniscus on FA skeleton (left) | No | No |
| **134** | dMRI skeleton | 25066 | Mean FA in inferior cerebellar peduncle on FA skeleton (right) | No | No |
| **134** | dMRI skeleton | 25067 | Mean FA in inferior cerebellar peduncle on FA skeleton (left) | No | No |
| **134** | dMRI skeleton | 25068 | Mean FA in superior cerebellar peduncle on FA skeleton (right) | No | No |
| **134** | dMRI skeleton | 25069 | Mean FA in superior cerebellar peduncle on FA skeleton (left) | No | No |
| **134** | dMRI skeleton | 25070 | Mean FA in cerebral peduncle on FA skeleton (right) | No | No |
| **134** | dMRI skeleton | 25071 | Mean FA in cerebral peduncle on FA skeleton (left) | No | No |
| **134** | dMRI skeleton | 25072 | Mean FA in anterior limb of internal capsule on FA skeleton (right) | No | No |
| **134** | dMRI skeleton | 25073 | Mean FA in anterior limb of internal capsule on FA skeleton (left) | No | No |
| **134** | dMRI skeleton | 25074 | Mean FA in posterior limb of internal capsule on FA skeleton (right) | No | No |
| **134** | dMRI skeleton | 25075 | Mean FA in posterior limb of internal capsule on FA skeleton (left) | No | No |
| **134** | dMRI skeleton | 25076 | Mean FA in retrolenticular part of internal capsule on FA skeleton (right) | No | No |
| **134** | dMRI skeleton | 25077 | Mean FA in retrolenticular part of internal capsule on FA skeleton (left) | No | No |
| **134** | dMRI skeleton | 25078 | Mean FA in anterior corona radiata on FA skeleton (right) | No | No |
| **134** | dMRI skeleton | 25079 | Mean FA in anterior corona radiata on FA skeleton (left) | No | No |
| **134** | dMRI skeleton | 25080 | Mean FA in superior corona radiata on FA skeleton (right) | No | No |
| **134** | dMRI skeleton | 25081 | Mean FA in superior corona radiata on FA skeleton (left) | No | No |
| **134** | dMRI skeleton | 25082 | Mean FA in posterior corona radiata on FA skeleton (right) | No | No |
| **134** | dMRI skeleton | 25083 | Mean FA in posterior corona radiata on FA skeleton (left) | No | No |
| **134** | dMRI skeleton | 25084 | Mean FA in posterior thalamic radiation on FA skeleton (right) | No | No |
| **134** | dMRI skeleton | 25085 | Mean FA in posterior thalamic radiation on FA skeleton (left) | No | No |
| **134** | dMRI skeleton | 25086 | Mean FA in sagittal stratum on FA skeleton (right) | No | No |
| **134** | dMRI skeleton | 25087 | Mean FA in sagittal stratum on FA skeleton (left) | No | No |
| **134** | dMRI skeleton | 25088 | Mean FA in external capsule on FA skeleton (right) | No | No |
| **134** | dMRI skeleton | 25089 | Mean FA in external capsule on FA skeleton (left) | No | No |
| **134** | dMRI skeleton | 25090 | Mean FA in cingulum cingulate gyrus on FA skeleton (right) | No | No |
| **134** | dMRI skeleton | 25091 | Mean FA in cingulum cingulate gyrus on FA skeleton (left) | No | No |
| **134** | dMRI skeleton | 25092 | Mean FA in cingulum hippocampus on FA skeleton (right) | No | No |
| **134** | dMRI skeleton | 25093 | Mean FA in cingulum hippocampus on FA skeleton (left) | No | No |
| **134** | dMRI skeleton | 25094 | Mean FA in fornix cres+stria terminalis on FA skeleton (right) | No | No |
| **134** | dMRI skeleton | 25095 | Mean FA in fornix cres+stria terminalis on FA skeleton (left) | No | No |
| **134** | dMRI skeleton | 25096 | Mean FA in superior longitudinal fasciculus on FA skeleton (right) | No | No |
| **134** | dMRI skeleton | 25097 | Mean FA in superior longitudinal fasciculus on FA skeleton (left) | No | No |
| **134** | dMRI skeleton | 25098 | Mean FA in superior fronto-occipital fasciculus on FA skeleton (right) | No | No |
| **134** | dMRI skeleton | 25099 | Mean FA in superior fronto-occipital fasciculus on FA skeleton (left) | No | No |
| **134** | dMRI skeleton | 25100 | Mean FA in uncinate fasciculus on FA skeleton (right) | No | No |
| **134** | dMRI skeleton | 25101 | Mean FA in uncinate fasciculus on FA skeleton (left) | No | No |
| **134** | dMRI skeleton | 25102 | Mean FA in tapetum on FA skeleton (right) | No | No |
| **134** | dMRI skeleton | 25103 | Mean FA in tapetum on FA skeleton (left) | No | No |
| **134** | dMRI skeleton | 25104 | Mean MD in middle cerebellar peduncle on FA skeleton | No | No |
| **134** | dMRI skeleton | 25105 | Mean MD in pontine crossing tract on FA skeleton | No | No |
| **134** | dMRI skeleton | 25106 | Mean MD in genu of corpus callosum on FA skeleton | No | No |
| **134** | dMRI skeleton | 25107 | Mean MD in body of corpus callosum on FA skeleton | No | No |
| **134** | dMRI skeleton | 25108 | Mean MD in splenium of corpus callosum on FA skeleton | No | No |
| **134** | dMRI skeleton | 25109 | Mean MD in fornix on FA skeleton | No | No |
| **134** | dMRI skeleton | 25110 | Mean MD in corticospinal tract on FA skeleton (right) | No | No |
| **134** | dMRI skeleton | 25111 | Mean MD in corticospinal tract on FA skeleton (left) | No | No |
| **134** | dMRI skeleton | 25112 | Mean MD in medial lemniscus on FA skeleton (right) | No | No |
| **134** | dMRI skeleton | 25113 | Mean MD in medial lemniscus on FA skeleton (left) | No | No |
| **134** | dMRI skeleton | 25114 | Mean MD in inferior cerebellar peduncle on FA skeleton (right) | No | No |
| **134** | dMRI skeleton | 25115 | Mean MD in inferior cerebellar peduncle on FA skeleton (left) | No | No |
| **134** | dMRI skeleton | 25116 | Mean MD in superior cerebellar peduncle on FA skeleton (right) | No | No |
| **134** | dMRI skeleton | 25117 | Mean MD in superior cerebellar peduncle on FA skeleton (left) | No | No |
| **134** | dMRI skeleton | 25118 | Mean MD in cerebral peduncle on FA skeleton (right) | No | No |
| **134** | dMRI skeleton | 25119 | Mean MD in cerebral peduncle on FA skeleton (left) | No | No |
| **134** | dMRI skeleton | 25120 | Mean MD in anterior limb of internal capsule on FA skeleton (right) | No | No |
| **134** | dMRI skeleton | 25121 | Mean MD in anterior limb of internal capsule on FA skeleton (left) | No | No |
| **134** | dMRI skeleton | 25122 | Mean MD in posterior limb of internal capsule on FA skeleton (right) | No | No |
| **134** | dMRI skeleton | 25123 | Mean MD in posterior limb of internal capsule on FA skeleton (left) | No | No |
| **134** | dMRI skeleton | 25124 | Mean MD in retrolenticular part of internal capsule on FA skeleton (right) | No | No |
| **134** | dMRI skeleton | 25125 | Mean MD in retrolenticular part of internal capsule on FA skeleton (left) | No | No |
| **134** | dMRI skeleton | 25126 | Mean MD in anterior corona radiata on FA skeleton (right) | No | No |
| **134** | dMRI skeleton | 25127 | Mean MD in anterior corona radiata on FA skeleton (left) | No | No |
| **134** | dMRI skeleton | 25128 | Mean MD in superior corona radiata on FA skeleton (right) | No | No |
| **134** | dMRI skeleton | 25129 | Mean MD in superior corona radiata on FA skeleton (left) | No | No |
| **134** | dMRI skeleton | 25130 | Mean MD in posterior corona radiata on FA skeleton (right) | No | No |
| **134** | dMRI skeleton | 25131 | Mean MD in posterior corona radiata on FA skeleton (left) | No | No |
| **134** | dMRI skeleton | 25132 | Mean MD in posterior thalamic radiation on FA skeleton (right) | No | No |
| **134** | dMRI skeleton | 25133 | Mean MD in posterior thalamic radiation on FA skeleton (left) | No | No |
| **134** | dMRI skeleton | 25134 | Mean MD in sagittal stratum on FA skeleton (right) | No | No |
| **134** | dMRI skeleton | 25135 | Mean MD in sagittal stratum on FA skeleton (left) | No | No |
| **134** | dMRI skeleton | 25136 | Mean MD in external capsule on FA skeleton (right) | No | No |
| **134** | dMRI skeleton | 25137 | Mean MD in external capsule on FA skeleton (left) | No | No |
| **134** | dMRI skeleton | 25138 | Mean MD in cingulum cingulate gyrus on FA skeleton (right) | No | No |
| **134** | dMRI skeleton | 25139 | Mean MD in cingulum cingulate gyrus on FA skeleton (left) | No | No |
| **134** | dMRI skeleton | 25140 | Mean MD in cingulum hippocampus on FA skeleton (right) | No | No |
| **134** | dMRI skeleton | 25141 | Mean MD in cingulum hippocampus on FA skeleton (left) | No | No |
| **134** | dMRI skeleton | 25142 | Mean MD in fornix cres+stria terminalis on FA skeleton (right) | No | No |
| **134** | dMRI skeleton | 25143 | Mean MD in fornix cres+stria terminalis on FA skeleton (left) | No | No |
| **134** | dMRI skeleton | 25144 | Mean MD in superior longitudinal fasciculus on FA skeleton (right) | No | No |
| **134** | dMRI skeleton | 25145 | Mean MD in superior longitudinal fasciculus on FA skeleton (left) | No | No |
| **134** | dMRI skeleton | 25146 | Mean MD in superior fronto-occipital fasciculus on FA skeleton (right) | No | No |
| **134** | dMRI skeleton | 25147 | Mean MD in superior fronto-occipital fasciculus on FA skeleton (left) | No | No |
| **134** | dMRI skeleton | 25148 | Mean MD in uncinate fasciculus on FA skeleton (right) | No | No |
| **134** | dMRI skeleton | 25149 | Mean MD in uncinate fasciculus on FA skeleton (left) | No | No |
| **134** | dMRI skeleton | 25150 | Mean MD in tapetum on FA skeleton (right) | No | No |
| **134** | dMRI skeleton | 25151 | Mean MD in tapetum on FA skeleton (left) | No | No |
| **134** | dMRI skeleton | 25152 | Mean MO in middle cerebellar peduncle on FA skeleton | No | No |
| **134** | dMRI skeleton | 25153 | Mean MO in pontine crossing tract on FA skeleton | No | No |
| **134** | dMRI skeleton | 25154 | Mean MO in genu of corpus callosum on FA skeleton | No | No |
| **134** | dMRI skeleton | 25155 | Mean MO in body of corpus callosum on FA skeleton | No | No |
| **134** | dMRI skeleton | 25156 | Mean MO in splenium of corpus callosum on FA skeleton | No | No |
| **134** | dMRI skeleton | 25157 | Mean MO in fornix on FA skeleton | No | No |
| **134** | dMRI skeleton | 25158 | Mean MO in corticospinal tract on FA skeleton (right) | No | No |
| **134** | dMRI skeleton | 25159 | Mean MO in corticospinal tract on FA skeleton (left) | No | No |
| **134** | dMRI skeleton | 25160 | Mean MO in medial lemniscus on FA skeleton (right) | No | No |
| **134** | dMRI skeleton | 25161 | Mean MO in medial lemniscus on FA skeleton (left) | No | No |
| **134** | dMRI skeleton | 25162 | Mean MO in inferior cerebellar peduncle on FA skeleton (right) | No | No |
| **134** | dMRI skeleton | 25163 | Mean MO in inferior cerebellar peduncle on FA skeleton (left) | No | No |
| **134** | dMRI skeleton | 25164 | Mean MO in superior cerebellar peduncle on FA skeleton (right) | No | No |
| **134** | dMRI skeleton | 25165 | Mean MO in superior cerebellar peduncle on FA skeleton (left) | No | No |
| **134** | dMRI skeleton | 25166 | Mean MO in cerebral peduncle on FA skeleton (right) | No | No |
| **134** | dMRI skeleton | 25167 | Mean MO in cerebral peduncle on FA skeleton (left) | No | No |
| **134** | dMRI skeleton | 25168 | Mean MO in anterior limb of internal capsule on FA skeleton (right) | No | No |
| **134** | dMRI skeleton | 25169 | Mean MO in anterior limb of internal capsule on FA skeleton (left) | No | No |
| **134** | dMRI skeleton | 25170 | Mean MO in posterior limb of internal capsule on FA skeleton (right) | No | No |
| **134** | dMRI skeleton | 25171 | Mean MO in posterior limb of internal capsule on FA skeleton (left) | No | No |
| **134** | dMRI skeleton | 25172 | Mean MO in retrolenticular part of internal capsule on FA skeleton (right) | No | No |
| **134** | dMRI skeleton | 25173 | Mean MO in retrolenticular part of internal capsule on FA skeleton (left) | No | No |
| **134** | dMRI skeleton | 25174 | Mean MO in anterior corona radiata on FA skeleton (right) | No | No |
| **134** | dMRI skeleton | 25175 | Mean MO in anterior corona radiata on FA skeleton (left) | No | No |
| **134** | dMRI skeleton | 25176 | Mean MO in superior corona radiata on FA skeleton (right) | No | No |
| **134** | dMRI skeleton | 25177 | Mean MO in superior corona radiata on FA skeleton (left) | No | No |
| **134** | dMRI skeleton | 25178 | Mean MO in posterior corona radiata on FA skeleton (right) | No | No |
| **134** | dMRI skeleton | 25179 | Mean MO in posterior corona radiata on FA skeleton (left) | No | No |
| **134** | dMRI skeleton | 25180 | Mean MO in posterior thalamic radiation on FA skeleton (right) | No | No |
| **134** | dMRI skeleton | 25181 | Mean MO in posterior thalamic radiation on FA skeleton (left) | No | No |
| **134** | dMRI skeleton | 25182 | Mean MO in sagittal stratum on FA skeleton (right) | No | No |
| **134** | dMRI skeleton | 25183 | Mean MO in sagittal stratum on FA skeleton (left) | No | No |
| **134** | dMRI skeleton | 25184 | Mean MO in external capsule on FA skeleton (right) | No | No |
| **134** | dMRI skeleton | 25185 | Mean MO in external capsule on FA skeleton (left) | No | No |
| **134** | dMRI skeleton | 25186 | Mean MO in cingulum cingulate gyrus on FA skeleton (right) | No | No |
| **134** | dMRI skeleton | 25187 | Mean MO in cingulum cingulate gyrus on FA skeleton (left) | No | No |
| **134** | dMRI skeleton | 25188 | Mean MO in cingulum hippocampus on FA skeleton (right) | No | No |
| **134** | dMRI skeleton | 25189 | Mean MO in cingulum hippocampus on FA skeleton (left) | No | No |
| **134** | dMRI skeleton | 25190 | Mean MO in fornix cres+stria terminalis on FA skeleton (right) | No | No |
| **134** | dMRI skeleton | 25191 | Mean MO in fornix cres+stria terminalis on FA skeleton (left) | No | No |
| **134** | dMRI skeleton | 25192 | Mean MO in superior longitudinal fasciculus on FA skeleton (right) | No | No |
| **134** | dMRI skeleton | 25193 | Mean MO in superior longitudinal fasciculus on FA skeleton (left) | No | No |
| **134** | dMRI skeleton | 25194 | Mean MO in superior fronto-occipital fasciculus on FA skeleton (right) | No | No |
| **134** | dMRI skeleton | 25195 | Mean MO in superior fronto-occipital fasciculus on FA skeleton (left) | No | No |
| **134** | dMRI skeleton | 25196 | Mean MO in uncinate fasciculus on FA skeleton (right) | No | No |
| **134** | dMRI skeleton | 25197 | Mean MO in uncinate fasciculus on FA skeleton (left) | No | No |
| **134** | dMRI skeleton | 25198 | Mean MO in tapetum on FA skeleton (right) | No | No |
| **134** | dMRI skeleton | 25199 | Mean MO in tapetum on FA skeleton (left) | No | No |
| **134** | dMRI skeleton | 25200 | Mean L1 in middle cerebellar peduncle on FA skeleton | No | No |
| **134** | dMRI skeleton | 25201 | Mean L1 in pontine crossing tract on FA skeleton | No | No |
| **134** | dMRI skeleton | 25202 | Mean L1 in genu of corpus callosum on FA skeleton | No | No |
| **134** | dMRI skeleton | 25203 | Mean L1 in body of corpus callosum on FA skeleton | No | No |
| **134** | dMRI skeleton | 25204 | Mean L1 in splenium of corpus callosum on FA skeleton | No | No |
| **134** | dMRI skeleton | 25205 | Mean L1 in fornix on FA skeleton | No | No |
| **134** | dMRI skeleton | 25206 | Mean L1 in corticospinal tract on FA skeleton (right) | No | No |
| **134** | dMRI skeleton | 25207 | Mean L1 in corticospinal tract on FA skeleton (left) | No | No |
| **134** | dMRI skeleton | 25208 | Mean L1 in medial lemniscus on FA skeleton (right) | No | No |
| **134** | dMRI skeleton | 25209 | Mean L1 in medial lemniscus on FA skeleton (left) | No | No |
| **134** | dMRI skeleton | 25210 | Mean L1 in inferior cerebellar peduncle on FA skeleton (right) | No | No |
| **134** | dMRI skeleton | 25211 | Mean L1 in inferior cerebellar peduncle on FA skeleton (left) | No | No |
| **134** | dMRI skeleton | 25212 | Mean L1 in superior cerebellar peduncle on FA skeleton (right) | No | No |
| **134** | dMRI skeleton | 25213 | Mean L1 in superior cerebellar peduncle on FA skeleton (left) | No | No |
| **134** | dMRI skeleton | 25214 | Mean L1 in cerebral peduncle on FA skeleton (right) | No | No |
| **134** | dMRI skeleton | 25215 | Mean L1 in cerebral peduncle on FA skeleton (left) | No | No |
| **134** | dMRI skeleton | 25216 | Mean L1 in anterior limb of internal capsule on FA skeleton (right) | No | No |
| **134** | dMRI skeleton | 25217 | Mean L1 in anterior limb of internal capsule on FA skeleton (left) | No | No |
| **134** | dMRI skeleton | 25218 | Mean L1 in posterior limb of internal capsule on FA skeleton (right) | No | No |
| **134** | dMRI skeleton | 25219 | Mean L1 in posterior limb of internal capsule on FA skeleton (left) | No | No |
| **134** | dMRI skeleton | 25220 | Mean L1 in retrolenticular part of internal capsule on FA skeleton (right) | No | No |
| **134** | dMRI skeleton | 25221 | Mean L1 in retrolenticular part of internal capsule on FA skeleton (left) | No | No |
| **134** | dMRI skeleton | 25222 | Mean L1 in anterior corona radiata on FA skeleton (right) | No | No |
| **134** | dMRI skeleton | 25223 | Mean L1 in anterior corona radiata on FA skeleton (left) | No | No |
| **134** | dMRI skeleton | 25224 | Mean L1 in superior corona radiata on FA skeleton (right) | No | No |
| **134** | dMRI skeleton | 25225 | Mean L1 in superior corona radiata on FA skeleton (left) | No | No |
| **134** | dMRI skeleton | 25226 | Mean L1 in posterior corona radiata on FA skeleton (right) | No | No |
| **134** | dMRI skeleton | 25227 | Mean L1 in posterior corona radiata on FA skeleton (left) | No | No |
| **134** | dMRI skeleton | 25228 | Mean L1 in posterior thalamic radiation on FA skeleton (right) | No | No |
| **134** | dMRI skeleton | 25229 | Mean L1 in posterior thalamic radiation on FA skeleton (left) | No | No |
| **134** | dMRI skeleton | 25230 | Mean L1 in sagittal stratum on FA skeleton (right) | No | No |
| **134** | dMRI skeleton | 25231 | Mean L1 in sagittal stratum on FA skeleton (left) | No | No |
| **134** | dMRI skeleton | 25232 | Mean L1 in external capsule on FA skeleton (right) | No | No |
| **134** | dMRI skeleton | 25233 | Mean L1 in external capsule on FA skeleton (left) | No | No |
| **134** | dMRI skeleton | 25234 | Mean L1 in cingulum cingulate gyrus on FA skeleton (right) | No | No |
| **134** | dMRI skeleton | 25235 | Mean L1 in cingulum cingulate gyrus on FA skeleton (left) | No | No |
| **134** | dMRI skeleton | 25236 | Mean L1 in cingulum hippocampus on FA skeleton (right) | No | No |
| **134** | dMRI skeleton | 25237 | Mean L1 in cingulum hippocampus on FA skeleton (left) | No | No |
| **134** | dMRI skeleton | 25238 | Mean L1 in fornix cres+stria terminalis on FA skeleton (right) | No | No |
| **134** | dMRI skeleton | 25239 | Mean L1 in fornix cres+stria terminalis on FA skeleton (left) | No | No |
| **134** | dMRI skeleton | 25240 | Mean L1 in superior longitudinal fasciculus on FA skeleton (right) | No | No |
| **134** | dMRI skeleton | 25241 | Mean L1 in superior longitudinal fasciculus on FA skeleton (left) | No | No |
| **134** | dMRI skeleton | 25242 | Mean L1 in superior fronto-occipital fasciculus on FA skeleton (right) | No | No |
| **134** | dMRI skeleton | 25243 | Mean L1 in superior fronto-occipital fasciculus on FA skeleton (left) | No | No |
| **134** | dMRI skeleton | 25244 | Mean L1 in uncinate fasciculus on FA skeleton (right) | No | No |
| **134** | dMRI skeleton | 25245 | Mean L1 in uncinate fasciculus on FA skeleton (left) | No | No |
| **134** | dMRI skeleton | 25246 | Mean L1 in tapetum on FA skeleton (right) | No | No |
| **134** | dMRI skeleton | 25247 | Mean L1 in tapetum on FA skeleton (left) | No | No |
| **134** | dMRI skeleton | 25248 | Mean L2 in middle cerebellar peduncle on FA skeleton | No | No |
| **134** | dMRI skeleton | 25249 | Mean L2 in pontine crossing tract on FA skeleton | No | No |
| **134** | dMRI skeleton | 25250 | Mean L2 in genu of corpus callosum on FA skeleton | No | No |
| **134** | dMRI skeleton | 25251 | Mean L2 in body of corpus callosum on FA skeleton | No | No |
| **134** | dMRI skeleton | 25252 | Mean L2 in splenium of corpus callosum on FA skeleton | No | No |
| **134** | dMRI skeleton | 25253 | Mean L2 in fornix on FA skeleton | No | No |
| **134** | dMRI skeleton | 25254 | Mean L2 in corticospinal tract on FA skeleton (right) | No | No |
| **134** | dMRI skeleton | 25255 | Mean L2 in corticospinal tract on FA skeleton (left) | No | No |
| **134** | dMRI skeleton | 25256 | Mean L2 in medial lemniscus on FA skeleton (right) | No | No |
| **134** | dMRI skeleton | 25257 | Mean L2 in medial lemniscus on FA skeleton (left) | No | No |
| **134** | dMRI skeleton | 25258 | Mean L2 in inferior cerebellar peduncle on FA skeleton (right) | No | No |
| **134** | dMRI skeleton | 25259 | Mean L2 in inferior cerebellar peduncle on FA skeleton (left) | No | No |
| **134** | dMRI skeleton | 25260 | Mean L2 in superior cerebellar peduncle on FA skeleton (right) | No | No |
| **134** | dMRI skeleton | 25261 | Mean L2 in superior cerebellar peduncle on FA skeleton (left) | No | No |
| **134** | dMRI skeleton | 25262 | Mean L2 in cerebral peduncle on FA skeleton (right) | No | No |
| **134** | dMRI skeleton | 25263 | Mean L2 in cerebral peduncle on FA skeleton (left) | No | No |
| **134** | dMRI skeleton | 25264 | Mean L2 in anterior limb of internal capsule on FA skeleton (right) | No | No |
| **134** | dMRI skeleton | 25265 | Mean L2 in anterior limb of internal capsule on FA skeleton (left) | No | No |
| **134** | dMRI skeleton | 25266 | Mean L2 in posterior limb of internal capsule on FA skeleton (right) | No | No |
| **134** | dMRI skeleton | 25267 | Mean L2 in posterior limb of internal capsule on FA skeleton (left) | No | No |
| **134** | dMRI skeleton | 25268 | Mean L2 in retrolenticular part of internal capsule on FA skeleton (right) | No | No |
| **134** | dMRI skeleton | 25269 | Mean L2 in retrolenticular part of internal capsule on FA skeleton (left) | No | No |
| **134** | dMRI skeleton | 25270 | Mean L2 in anterior corona radiata on FA skeleton (right) | No | No |
| **134** | dMRI skeleton | 25271 | Mean L2 in anterior corona radiata on FA skeleton (left) | No | No |
| **134** | dMRI skeleton | 25272 | Mean L2 in superior corona radiata on FA skeleton (right) | No | No |
| **134** | dMRI skeleton | 25273 | Mean L2 in superior corona radiata on FA skeleton (left) | No | No |
| **134** | dMRI skeleton | 25274 | Mean L2 in posterior corona radiata on FA skeleton (right) | No | No |
| **134** | dMRI skeleton | 25275 | Mean L2 in posterior corona radiata on FA skeleton (left) | No | No |
| **134** | dMRI skeleton | 25276 | Mean L2 in posterior thalamic radiation on FA skeleton (right) | No | No |
| **134** | dMRI skeleton | 25277 | Mean L2 in posterior thalamic radiation on FA skeleton (left) | No | No |
| **134** | dMRI skeleton | 25278 | Mean L2 in sagittal stratum on FA skeleton (right) | No | No |
| **134** | dMRI skeleton | 25279 | Mean L2 in sagittal stratum on FA skeleton (left) | No | No |
| **134** | dMRI skeleton | 25280 | Mean L2 in external capsule on FA skeleton (right) | No | No |
| **134** | dMRI skeleton | 25281 | Mean L2 in external capsule on FA skeleton (left) | No | No |
| **134** | dMRI skeleton | 25282 | Mean L2 in cingulum cingulate gyrus on FA skeleton (right) | No | No |
| **134** | dMRI skeleton | 25283 | Mean L2 in cingulum cingulate gyrus on FA skeleton (left) | No | No |
| **134** | dMRI skeleton | 25284 | Mean L2 in cingulum hippocampus on FA skeleton (right) | No | No |
| **134** | dMRI skeleton | 25285 | Mean L2 in cingulum hippocampus on FA skeleton (left) | No | No |
| **134** | dMRI skeleton | 25286 | Mean L2 in fornix cres+stria terminalis on FA skeleton (right) | No | No |
| **134** | dMRI skeleton | 25287 | Mean L2 in fornix cres+stria terminalis on FA skeleton (left) | No | No |
| **134** | dMRI skeleton | 25288 | Mean L2 in superior longitudinal fasciculus on FA skeleton (right) | No | No |
| **134** | dMRI skeleton | 25289 | Mean L2 in superior longitudinal fasciculus on FA skeleton (left) | No | No |
| **134** | dMRI skeleton | 25290 | Mean L2 in superior fronto-occipital fasciculus on FA skeleton (right) | No | No |
| **134** | dMRI skeleton | 25291 | Mean L2 in superior fronto-occipital fasciculus on FA skeleton (left) | No | No |
| **134** | dMRI skeleton | 25292 | Mean L2 in uncinate fasciculus on FA skeleton (right) | No | No |
| **134** | dMRI skeleton | 25293 | Mean L2 in uncinate fasciculus on FA skeleton (left) | No | No |
| **134** | dMRI skeleton | 25294 | Mean L2 in tapetum on FA skeleton (right) | No | No |
| **134** | dMRI skeleton | 25295 | Mean L2 in tapetum on FA skeleton (left) | No | No |
| **134** | dMRI skeleton | 25296 | Mean L3 in middle cerebellar peduncle on FA skeleton | No | No |
| **134** | dMRI skeleton | 25297 | Mean L3 in pontine crossing tract on FA skeleton | No | No |
| **134** | dMRI skeleton | 25298 | Mean L3 in genu of corpus callosum on FA skeleton | No | No |
| **134** | dMRI skeleton | 25299 | Mean L3 in body of corpus callosum on FA skeleton | No | No |
| **134** | dMRI skeleton | 25300 | Mean L3 in splenium of corpus callosum on FA skeleton | No | No |
| **134** | dMRI skeleton | 25301 | Mean L3 in fornix on FA skeleton | No | No |
| **134** | dMRI skeleton | 25302 | Mean L3 in corticospinal tract on FA skeleton (right) | No | No |
| **134** | dMRI skeleton | 25303 | Mean L3 in corticospinal tract on FA skeleton (left) | No | No |
| **134** | dMRI skeleton | 25304 | Mean L3 in medial lemniscus on FA skeleton (right) | No | No |
| **134** | dMRI skeleton | 25305 | Mean L3 in medial lemniscus on FA skeleton (left) | No | No |
| **134** | dMRI skeleton | 25306 | Mean L3 in inferior cerebellar peduncle on FA skeleton (right) | No | No |
| **134** | dMRI skeleton | 25307 | Mean L3 in inferior cerebellar peduncle on FA skeleton (left) | No | No |
| **134** | dMRI skeleton | 25308 | Mean L3 in superior cerebellar peduncle on FA skeleton (right) | No | No |
| **134** | dMRI skeleton | 25309 | Mean L3 in superior cerebellar peduncle on FA skeleton (left) | No | No |
| **134** | dMRI skeleton | 25310 | Mean L3 in cerebral peduncle on FA skeleton (right) | No | No |
| **134** | dMRI skeleton | 25311 | Mean L3 in cerebral peduncle on FA skeleton (left) | No | No |
| **134** | dMRI skeleton | 25312 | Mean L3 in anterior limb of internal capsule on FA skeleton (right) | No | No |
| **134** | dMRI skeleton | 25313 | Mean L3 in anterior limb of internal capsule on FA skeleton (left) | No | No |
| **134** | dMRI skeleton | 25314 | Mean L3 in posterior limb of internal capsule on FA skeleton (right) | No | No |
| **134** | dMRI skeleton | 25315 | Mean L3 in posterior limb of internal capsule on FA skeleton (left) | No | No |
| **134** | dMRI skeleton | 25316 | Mean L3 in retrolenticular part of internal capsule on FA skeleton (right) | No | No |
| **134** | dMRI skeleton | 25317 | Mean L3 in retrolenticular part of internal capsule on FA skeleton (left) | No | No |
| **134** | dMRI skeleton | 25318 | Mean L3 in anterior corona radiata on FA skeleton (right) | No | No |
| **134** | dMRI skeleton | 25319 | Mean L3 in anterior corona radiata on FA skeleton (left) | No | No |
| **134** | dMRI skeleton | 25320 | Mean L3 in superior corona radiata on FA skeleton (right) | No | No |
| **134** | dMRI skeleton | 25321 | Mean L3 in superior corona radiata on FA skeleton (left) | No | No |
| **134** | dMRI skeleton | 25322 | Mean L3 in posterior corona radiata on FA skeleton (right) | No | No |
| **134** | dMRI skeleton | 25323 | Mean L3 in posterior corona radiata on FA skeleton (left) | No | No |
| **134** | dMRI skeleton | 25324 | Mean L3 in posterior thalamic radiation on FA skeleton (right) | No | No |
| **134** | dMRI skeleton | 25325 | Mean L3 in posterior thalamic radiation on FA skeleton (left) | No | No |
| **134** | dMRI skeleton | 25326 | Mean L3 in sagittal stratum on FA skeleton (right) | No | No |
| **134** | dMRI skeleton | 25327 | Mean L3 in sagittal stratum on FA skeleton (left) | No | No |
| **134** | dMRI skeleton | 25328 | Mean L3 in external capsule on FA skeleton (right) | No | No |
| **134** | dMRI skeleton | 25329 | Mean L3 in external capsule on FA skeleton (left) | No | No |
| **134** | dMRI skeleton | 25330 | Mean L3 in cingulum cingulate gyrus on FA skeleton (right) | No | No |
| **134** | dMRI skeleton | 25331 | Mean L3 in cingulum cingulate gyrus on FA skeleton (left) | No | No |
| **134** | dMRI skeleton | 25332 | Mean L3 in cingulum hippocampus on FA skeleton (right) | No | No |
| **134** | dMRI skeleton | 25333 | Mean L3 in cingulum hippocampus on FA skeleton (left) | No | No |
| **134** | dMRI skeleton | 25334 | Mean L3 in fornix cres+stria terminalis on FA skeleton (right) | No | No |
| **134** | dMRI skeleton | 25335 | Mean L3 in fornix cres+stria terminalis on FA skeleton (left) | No | No |
| **134** | dMRI skeleton | 25336 | Mean L3 in superior longitudinal fasciculus on FA skeleton (right) | No | No |
| **134** | dMRI skeleton | 25337 | Mean L3 in superior longitudinal fasciculus on FA skeleton (left) | No | No |
| **134** | dMRI skeleton | 25338 | Mean L3 in superior fronto-occipital fasciculus on FA skeleton (right) | No | No |
| **134** | dMRI skeleton | 25339 | Mean L3 in superior fronto-occipital fasciculus on FA skeleton (left) | No | No |
| **134** | dMRI skeleton | 25340 | Mean L3 in uncinate fasciculus on FA skeleton (right) | No | No |
| **134** | dMRI skeleton | 25341 | Mean L3 in uncinate fasciculus on FA skeleton (left) | No | No |
| **134** | dMRI skeleton | 25342 | Mean L3 in tapetum on FA skeleton (right) | No | No |
| **134** | dMRI skeleton | 25343 | Mean L3 in tapetum on FA skeleton (left) | No | No |
| **134** | dMRI skeleton | 25344 | Mean ICVF in middle cerebellar peduncle on FA skeleton | No | No |
| **134** | dMRI skeleton | 25345 | Mean ICVF in pontine crossing tract on FA skeleton | No | No |
| **134** | dMRI skeleton | 25346 | Mean ICVF in genu of corpus callosum on FA skeleton | No | No |
| **134** | dMRI skeleton | 25347 | Mean ICVF in body of corpus callosum on FA skeleton | No | No |
| **134** | dMRI skeleton | 25348 | Mean ICVF in splenium of corpus callosum on FA skeleton | No | No |
| **134** | dMRI skeleton | 25349 | Mean ICVF in fornix on FA skeleton | No | No |
| **134** | dMRI skeleton | 25350 | Mean ICVF in corticospinal tract on FA skeleton (right) | No | No |
| **134** | dMRI skeleton | 25351 | Mean ICVF in corticospinal tract on FA skeleton (left) | No | No |
| **134** | dMRI skeleton | 25352 | Mean ICVF in medial lemniscus on FA skeleton (right) | No | No |
| **134** | dMRI skeleton | 25353 | Mean ICVF in medial lemniscus on FA skeleton (left) | No | No |
| **134** | dMRI skeleton | 25354 | Mean ICVF in inferior cerebellar peduncle on FA skeleton (right) | No | No |
| **134** | dMRI skeleton | 25355 | Mean ICVF in inferior cerebellar peduncle on FA skeleton (left) | No | No |
| **134** | dMRI skeleton | 25356 | Mean ICVF in superior cerebellar peduncle on FA skeleton (right) | No | No |
| **134** | dMRI skeleton | 25357 | Mean ICVF in superior cerebellar peduncle on FA skeleton (left) | No | No |
| **134** | dMRI skeleton | 25358 | Mean ICVF in cerebral peduncle on FA skeleton (right) | No | No |
| **134** | dMRI skeleton | 25359 | Mean ICVF in cerebral peduncle on FA skeleton (left) | No | No |
| **134** | dMRI skeleton | 25360 | Mean ICVF in anterior limb of internal capsule on FA skeleton (right) | No | No |
| **134** | dMRI skeleton | 25361 | Mean ICVF in anterior limb of internal capsule on FA skeleton (left) | No | No |
| **134** | dMRI skeleton | 25362 | Mean ICVF in posterior limb of internal capsule on FA skeleton (right) | No | No |
| **134** | dMRI skeleton | 25363 | Mean ICVF in posterior limb of internal capsule on FA skeleton (left) | No | No |
| **134** | dMRI skeleton | 25364 | Mean ICVF in retrolenticular part of internal capsule on FA skeleton (right) | No | No |
| **134** | dMRI skeleton | 25365 | Mean ICVF in retrolenticular part of internal capsule on FA skeleton (left) | No | No |
| **134** | dMRI skeleton | 25366 | Mean ICVF in anterior corona radiata on FA skeleton (right) | No | No |
| **134** | dMRI skeleton | 25367 | Mean ICVF in anterior corona radiata on FA skeleton (left) | No | No |
| **134** | dMRI skeleton | 25368 | Mean ICVF in superior corona radiata on FA skeleton (right) | No | No |
| **134** | dMRI skeleton | 25369 | Mean ICVF in superior corona radiata on FA skeleton (left) | No | No |
| **134** | dMRI skeleton | 25370 | Mean ICVF in posterior corona radiata on FA skeleton (right) | No | No |
| **134** | dMRI skeleton | 25371 | Mean ICVF in posterior corona radiata on FA skeleton (left) | No | No |
| **134** | dMRI skeleton | 25372 | Mean ICVF in posterior thalamic radiation on FA skeleton (right) | No | No |
| **134** | dMRI skeleton | 25373 | Mean ICVF in posterior thalamic radiation on FA skeleton (left) | No | No |
| **134** | dMRI skeleton | 25374 | Mean ICVF in sagittal stratum on FA skeleton (right) | No | No |
| **134** | dMRI skeleton | 25375 | Mean ICVF in sagittal stratum on FA skeleton (left) | No | No |
| **134** | dMRI skeleton | 25376 | Mean ICVF in external capsule on FA skeleton (right) | No | No |
| **134** | dMRI skeleton | 25377 | Mean ICVF in external capsule on FA skeleton (left) | No | No |
| **134** | dMRI skeleton | 25378 | Mean ICVF in cingulum cingulate gyrus on FA skeleton (right) | No | No |
| **134** | dMRI skeleton | 25379 | Mean ICVF in cingulum cingulate gyrus on FA skeleton (left) | No | No |
| **134** | dMRI skeleton | 25380 | Mean ICVF in cingulum hippocampus on FA skeleton (right) | No | No |
| **134** | dMRI skeleton | 25381 | Mean ICVF in cingulum hippocampus on FA skeleton (left) | No | No |
| **134** | dMRI skeleton | 25382 | Mean ICVF in fornix cres+stria terminalis on FA skeleton (right) | No | No |
| **134** | dMRI skeleton | 25383 | Mean ICVF in fornix cres+stria terminalis on FA skeleton (left) | No | No |
| **134** | dMRI skeleton | 25384 | Mean ICVF in superior longitudinal fasciculus on FA skeleton (right) | No | No |
| **134** | dMRI skeleton | 25385 | Mean ICVF in superior longitudinal fasciculus on FA skeleton (left) | No | No |
| **134** | dMRI skeleton | 25386 | Mean ICVF in superior fronto-occipital fasciculus on FA skeleton (right) | No | No |
| **134** | dMRI skeleton | 25387 | Mean ICVF in superior fronto-occipital fasciculus on FA skeleton (left) | No | No |
| **134** | dMRI skeleton | 25388 | Mean ICVF in uncinate fasciculus on FA skeleton (right) | No | No |
| **134** | dMRI skeleton | 25389 | Mean ICVF in uncinate fasciculus on FA skeleton (left) | No | No |
| **134** | dMRI skeleton | 25390 | Mean ICVF in tapetum on FA skeleton (right) | No | No |
| **134** | dMRI skeleton | 25391 | Mean ICVF in tapetum on FA skeleton (left) | No | No |
| **134** | dMRI skeleton | 25392 | Mean OD in middle cerebellar peduncle on FA skeleton | No | No |
| **134** | dMRI skeleton | 25393 | Mean OD in pontine crossing tract on FA skeleton | No | No |
| **134** | dMRI skeleton | 25394 | Mean OD in genu of corpus callosum on FA skeleton | No | No |
| **134** | dMRI skeleton | 25395 | Mean OD in body of corpus callosum on FA skeleton | No | No |
| **134** | dMRI skeleton | 25396 | Mean OD in splenium of corpus callosum on FA skeleton | No | No |
| **134** | dMRI skeleton | 25397 | Mean OD in fornix on FA skeleton | No | No |
| **134** | dMRI skeleton | 25398 | Mean OD in corticospinal tract on FA skeleton (right) | No | No |
| **134** | dMRI skeleton | 25399 | Mean OD in corticospinal tract on FA skeleton (left) | No | No |
| **134** | dMRI skeleton | 25400 | Mean OD in medial lemniscus on FA skeleton (right) | No | No |
| **134** | dMRI skeleton | 25401 | Mean OD in medial lemniscus on FA skeleton (left) | No | No |
| **134** | dMRI skeleton | 25402 | Mean OD in inferior cerebellar peduncle on FA skeleton (right) | No | No |
| **134** | dMRI skeleton | 25403 | Mean OD in inferior cerebellar peduncle on FA skeleton (left) | No | No |
| **134** | dMRI skeleton | 25404 | Mean OD in superior cerebellar peduncle on FA skeleton (right) | No | No |
| **134** | dMRI skeleton | 25405 | Mean OD in superior cerebellar peduncle on FA skeleton (left) | No | No |
| **134** | dMRI skeleton | 25406 | Mean OD in cerebral peduncle on FA skeleton (right) | No | No |
| **134** | dMRI skeleton | 25407 | Mean OD in cerebral peduncle on FA skeleton (left) | No | No |
| **134** | dMRI skeleton | 25408 | Mean OD in anterior limb of internal capsule on FA skeleton (right) | No | No |
| **134** | dMRI skeleton | 25409 | Mean OD in anterior limb of internal capsule on FA skeleton (left) | No | No |
| **134** | dMRI skeleton | 25410 | Mean OD in posterior limb of internal capsule on FA skeleton (right) | No | No |
| **134** | dMRI skeleton | 25411 | Mean OD in posterior limb of internal capsule on FA skeleton (left) | No | No |
| **134** | dMRI skeleton | 25412 | Mean OD in retrolenticular part of internal capsule on FA skeleton (right) | No | No |
| **134** | dMRI skeleton | 25413 | Mean OD in retrolenticular part of internal capsule on FA skeleton (left) | No | No |
| **134** | dMRI skeleton | 25414 | Mean OD in anterior corona radiata on FA skeleton (right) | No | No |
| **134** | dMRI skeleton | 25415 | Mean OD in anterior corona radiata on FA skeleton (left) | No | No |
| **134** | dMRI skeleton | 25416 | Mean OD in superior corona radiata on FA skeleton (right) | No | No |
| **134** | dMRI skeleton | 25417 | Mean OD in superior corona radiata on FA skeleton (left) | No | No |
| **134** | dMRI skeleton | 25418 | Mean OD in posterior corona radiata on FA skeleton (right) | No | No |
| **134** | dMRI skeleton | 25419 | Mean OD in posterior corona radiata on FA skeleton (left) | No | No |
| **134** | dMRI skeleton | 25420 | Mean OD in posterior thalamic radiation on FA skeleton (right) | No | No |
| **134** | dMRI skeleton | 25421 | Mean OD in posterior thalamic radiation on FA skeleton (left) | No | No |
| **134** | dMRI skeleton | 25422 | Mean OD in sagittal stratum on FA skeleton (right) | No | No |
| **134** | dMRI skeleton | 25423 | Mean OD in sagittal stratum on FA skeleton (left) | No | No |
| **134** | dMRI skeleton | 25424 | Mean OD in external capsule on FA skeleton (right) | No | No |
| **134** | dMRI skeleton | 25425 | Mean OD in external capsule on FA skeleton (left) | No | No |
| **134** | dMRI skeleton | 25426 | Mean OD in cingulum cingulate gyrus on FA skeleton (right) | No | No |
| **134** | dMRI skeleton | 25427 | Mean OD in cingulum cingulate gyrus on FA skeleton (left) | No | No |
| **134** | dMRI skeleton | 25428 | Mean OD in cingulum hippocampus on FA skeleton (right) | No | No |
| **134** | dMRI skeleton | 25429 | Mean OD in cingulum hippocampus on FA skeleton (left) | No | No |
| **134** | dMRI skeleton | 25430 | Mean OD in fornix cres+stria terminalis on FA skeleton (right) | No | No |
| **134** | dMRI skeleton | 25431 | Mean OD in fornix cres+stria terminalis on FA skeleton (left) | No | No |
| **134** | dMRI skeleton | 25432 | Mean OD in superior longitudinal fasciculus on FA skeleton (right) | No | No |
| **134** | dMRI skeleton | 25433 | Mean OD in superior longitudinal fasciculus on FA skeleton (left) | No | No |
| **134** | dMRI skeleton | 25434 | Mean OD in superior fronto-occipital fasciculus on FA skeleton (right) | No | No |
| **134** | dMRI skeleton | 25435 | Mean OD in superior fronto-occipital fasciculus on FA skeleton (left) | No | No |
| **134** | dMRI skeleton | 25436 | Mean OD in uncinate fasciculus on FA skeleton (right) | No | No |
| **134** | dMRI skeleton | 25437 | Mean OD in uncinate fasciculus on FA skeleton (left) | No | No |
| **134** | dMRI skeleton | 25438 | Mean OD in tapetum on FA skeleton (right) | No | No |
| **134** | dMRI skeleton | 25439 | Mean OD in tapetum on FA skeleton (left) | No | No |
| **134** | dMRI skeleton | 25440 | Mean ISOVF in middle cerebellar peduncle on FA skeleton | No | No |
| **134** | dMRI skeleton | 25441 | Mean ISOVF in pontine crossing tract on FA skeleton | No | No |
| **134** | dMRI skeleton | 25442 | Mean ISOVF in genu of corpus callosum on FA skeleton | No | No |
| **134** | dMRI skeleton | 25443 | Mean ISOVF in body of corpus callosum on FA skeleton | No | No |
| **134** | dMRI skeleton | 25444 | Mean ISOVF in splenium of corpus callosum on FA skeleton | No | No |
| **134** | dMRI skeleton | 25445 | Mean ISOVF in fornix on FA skeleton | No | No |
| **134** | dMRI skeleton | 25446 | Mean ISOVF in corticospinal tract on FA skeleton (right) | No | No |
| **134** | dMRI skeleton | 25447 | Mean ISOVF in corticospinal tract on FA skeleton (left) | No | No |
| **134** | dMRI skeleton | 25448 | Mean ISOVF in medial lemniscus on FA skeleton (right) | No | No |
| **134** | dMRI skeleton | 25449 | Mean ISOVF in medial lemniscus on FA skeleton (left) | No | No |
| **134** | dMRI skeleton | 25450 | Mean ISOVF in inferior cerebellar peduncle on FA skeleton (right) | No | No |
| **134** | dMRI skeleton | 25451 | Mean ISOVF in inferior cerebellar peduncle on FA skeleton (left) | No | No |
| **134** | dMRI skeleton | 25452 | Mean ISOVF in superior cerebellar peduncle on FA skeleton (right) | No | No |
| **134** | dMRI skeleton | 25453 | Mean ISOVF in superior cerebellar peduncle on FA skeleton (left) | No | No |
| **134** | dMRI skeleton | 25454 | Mean ISOVF in cerebral peduncle on FA skeleton (right) | No | No |
| **134** | dMRI skeleton | 25455 | Mean ISOVF in cerebral peduncle on FA skeleton (left) | No | No |
| **134** | dMRI skeleton | 25456 | Mean ISOVF in anterior limb of internal capsule on FA skeleton (right) | No | No |
| **134** | dMRI skeleton | 25457 | Mean ISOVF in anterior limb of internal capsule on FA skeleton (left) | No | No |
| **134** | dMRI skeleton | 25458 | Mean ISOVF in posterior limb of internal capsule on FA skeleton (right) | No | No |
| **134** | dMRI skeleton | 25459 | Mean ISOVF in posterior limb of internal capsule on FA skeleton (left) | No | No |
| **134** | dMRI skeleton | 25460 | Mean ISOVF in retrolenticular part of internal capsule on FA skeleton (right) | No | No |
| **134** | dMRI skeleton | 25461 | Mean ISOVF in retrolenticular part of internal capsule on FA skeleton (left) | No | No |
| **134** | dMRI skeleton | 25462 | Mean ISOVF in anterior corona radiata on FA skeleton (right) | No | No |
| **134** | dMRI skeleton | 25463 | Mean ISOVF in anterior corona radiata on FA skeleton (left) | No | No |
| **134** | dMRI skeleton | 25464 | Mean ISOVF in superior corona radiata on FA skeleton (right) | No | No |
| **134** | dMRI skeleton | 25465 | Mean ISOVF in superior corona radiata on FA skeleton (left) | No | No |
| **134** | dMRI skeleton | 25466 | Mean ISOVF in posterior corona radiata on FA skeleton (right) | No | No |
| **134** | dMRI skeleton | 25467 | Mean ISOVF in posterior corona radiata on FA skeleton (left) | No | No |
| **134** | dMRI skeleton | 25468 | Mean ISOVF in posterior thalamic radiation on FA skeleton (right) | No | No |
| **134** | dMRI skeleton | 25469 | Mean ISOVF in posterior thalamic radiation on FA skeleton (left) | No | No |
| **134** | dMRI skeleton | 25470 | Mean ISOVF in sagittal stratum on FA skeleton (right) | No | No |
| **134** | dMRI skeleton | 25471 | Mean ISOVF in sagittal stratum on FA skeleton (left) | No | No |
| **134** | dMRI skeleton | 25472 | Mean ISOVF in external capsule on FA skeleton (right) | No | No |
| **134** | dMRI skeleton | 25473 | Mean ISOVF in external capsule on FA skeleton (left) | No | No |
| **134** | dMRI skeleton | 25474 | Mean ISOVF in cingulum cingulate gyrus on FA skeleton (right) | No | No |
| **134** | dMRI skeleton | 25475 | Mean ISOVF in cingulum cingulate gyrus on FA skeleton (left) | No | No |
| **134** | dMRI skeleton | 25476 | Mean ISOVF in cingulum hippocampus on FA skeleton (right) | No | No |
| **134** | dMRI skeleton | 25477 | Mean ISOVF in cingulum hippocampus on FA skeleton (left) | No | No |
| **134** | dMRI skeleton | 25478 | Mean ISOVF in fornix cres+stria terminalis on FA skeleton (right) | No | No |
| **134** | dMRI skeleton | 25479 | Mean ISOVF in fornix cres+stria terminalis on FA skeleton (left) | No | No |
| **134** | dMRI skeleton | 25480 | Mean ISOVF in superior longitudinal fasciculus on FA skeleton (right) | No | No |
| **134** | dMRI skeleton | 25481 | Mean ISOVF in superior longitudinal fasciculus on FA skeleton (left) | No | No |
| **134** | dMRI skeleton | 25482 | Mean ISOVF in superior fronto-occipital fasciculus on FA skeleton (right) | No | No |
| **134** | dMRI skeleton | 25483 | Mean ISOVF in superior fronto-occipital fasciculus on FA skeleton (left) | No | No |
| **134** | dMRI skeleton | 25484 | Mean ISOVF in uncinate fasciculus on FA skeleton (right) | No | No |
| **134** | dMRI skeleton | 25485 | Mean ISOVF in uncinate fasciculus on FA skeleton (left) | No | No |
| **134** | dMRI skeleton | 25486 | Mean ISOVF in tapetum on FA skeleton (right) | No | No |
| **134** | dMRI skeleton | 25487 | Mean ISOVF in tapetum on FA skeleton (left) | No | No |
| **135** | dMRI weighted means | 25488 | Weighted-mean FA in tract acoustic radiation (left) | No | No |
| **135** | dMRI weighted means | 25489 | Weighted-mean FA in tract acoustic radiation (right) | No | No |
| **135** | dMRI weighted means | 25490 | Weighted-mean FA in tract anterior thalamic radiation (left) | No | No |
| **135** | dMRI weighted means | 25491 | Weighted-mean FA in tract anterior thalamic radiation (right) | No | No |
| **135** | dMRI weighted means | 25492 | Weighted-mean FA in tract cingulate gyrus part of cingulum (left) | No | No |
| **135** | dMRI weighted means | 25493 | Weighted-mean FA in tract cingulate gyrus part of cingulum (right) | No | No |
| **135** | dMRI weighted means | 25494 | Weighted-mean FA in tract parahippocampal part of cingulum (left) | No | No |
| **135** | dMRI weighted means | 25495 | Weighted-mean FA in tract parahippocampal part of cingulum (right) | No | No |
| **135** | dMRI weighted means | 25496 | Weighted-mean FA in tract corticospinal tract (left) | No | No |
| **135** | dMRI weighted means | 25497 | Weighted-mean FA in tract corticospinal tract (right) | No | No |
| **135** | dMRI weighted means | 25498 | Weighted-mean FA in tract forceps major | No | No |
| **135** | dMRI weighted means | 25499 | Weighted-mean FA in tract forceps minor | No | No |
| **135** | dMRI weighted means | 25500 | Weighted-mean FA in tract inferior fronto-occipital fasciculus (left) | No | No |
| **135** | dMRI weighted means | 25501 | Weighted-mean FA in tract inferior fronto-occipital fasciculus (right) | No | No |
| **135** | dMRI weighted means | 25502 | Weighted-mean FA in tract inferior longitudinal fasciculus (left) | No | No |
| **135** | dMRI weighted means | 25503 | Weighted-mean FA in tract inferior longitudinal fasciculus (right) | No | No |
| **135** | dMRI weighted means | 25504 | Weighted-mean FA in tract middle cerebellar peduncle | No | No |
| **135** | dMRI weighted means | 25505 | Weighted-mean FA in tract medial lemniscus (left) | No | No |
| **135** | dMRI weighted means | 25506 | Weighted-mean FA in tract medial lemniscus (right) | No | No |
| **135** | dMRI weighted means | 25507 | Weighted-mean FA in tract posterior thalamic radiation (left) | No | No |
| **135** | dMRI weighted means | 25508 | Weighted-mean FA in tract posterior thalamic radiation (right) | No | No |
| **135** | dMRI weighted means | 25509 | Weighted-mean FA in tract superior longitudinal fasciculus (left) | No | No |
| **135** | dMRI weighted means | 25510 | Weighted-mean FA in tract superior longitudinal fasciculus (right) | No | No |
| **135** | dMRI weighted means | 25511 | Weighted-mean FA in tract superior thalamic radiation (left) | No | No |
| **135** | dMRI weighted means | 25512 | Weighted-mean FA in tract superior thalamic radiation (right) | No | No |
| **135** | dMRI weighted means | 25513 | Weighted-mean FA in tract uncinate fasciculus (left) | No | No |
| **135** | dMRI weighted means | 25514 | Weighted-mean FA in tract uncinate fasciculus (right) | No | No |
| **135** | dMRI weighted means | 25515 | Weighted-mean MD in tract acoustic radiation (left) | No | No |
| **135** | dMRI weighted means | 25516 | Weighted-mean MD in tract acoustic radiation (right) | No | No |
| **135** | dMRI weighted means | 25517 | Weighted-mean MD in tract anterior thalamic radiation (left) | No | No |
| **135** | dMRI weighted means | 25518 | Weighted-mean MD in tract anterior thalamic radiation (right) | No | No |
| **135** | dMRI weighted means | 25519 | Weighted-mean MD in tract cingulate gyrus part of cingulum (left) | No | No |
| **135** | dMRI weighted means | 25520 | Weighted-mean MD in tract cingulate gyrus part of cingulum (right) | No | No |
| **135** | dMRI weighted means | 25521 | Weighted-mean MD in tract parahippocampal part of cingulum (left) | No | No |
| **135** | dMRI weighted means | 25522 | Weighted-mean MD in tract parahippocampal part of cingulum (right) | No | No |
| **135** | dMRI weighted means | 25523 | Weighted-mean MD in tract corticospinal tract (left) | No | No |
| **135** | dMRI weighted means | 25524 | Weighted-mean MD in tract corticospinal tract (right) | No | No |
| **135** | dMRI weighted means | 25525 | Weighted-mean MD in tract forceps major | No | No |
| **135** | dMRI weighted means | 25526 | Weighted-mean MD in tract forceps minor | No | No |
| **135** | dMRI weighted means | 25527 | Weighted-mean MD in tract inferior fronto-occipital fasciculus (left) | No | No |
| **135** | dMRI weighted means | 25528 | Weighted-mean MD in tract inferior fronto-occipital fasciculus (right) | No | No |
| **135** | dMRI weighted means | 25529 | Weighted-mean MD in tract inferior longitudinal fasciculus (left) | No | No |
| **135** | dMRI weighted means | 25530 | Weighted-mean MD in tract inferior longitudinal fasciculus (right) | No | No |
| **135** | dMRI weighted means | 25531 | Weighted-mean MD in tract middle cerebellar peduncle | No | No |
| **135** | dMRI weighted means | 25532 | Weighted-mean MD in tract medial lemniscus (left) | No | No |
| **135** | dMRI weighted means | 25533 | Weighted-mean MD in tract medial lemniscus (right) | No | No |
| **135** | dMRI weighted means | 25534 | Weighted-mean MD in tract posterior thalamic radiation (left) | No | No |
| **135** | dMRI weighted means | 25535 | Weighted-mean MD in tract posterior thalamic radiation (right) | No | No |
| **135** | dMRI weighted means | 25536 | Weighted-mean MD in tract superior longitudinal fasciculus (left) | No | No |
| **135** | dMRI weighted means | 25537 | Weighted-mean MD in tract superior longitudinal fasciculus (right) | No | No |
| **135** | dMRI weighted means | 25538 | Weighted-mean MD in tract superior thalamic radiation (left) | No | No |
| **135** | dMRI weighted means | 25539 | Weighted-mean MD in tract superior thalamic radiation (right) | No | No |
| **135** | dMRI weighted means | 25540 | Weighted-mean MD in tract uncinate fasciculus (left) | No | No |
| **135** | dMRI weighted means | 25541 | Weighted-mean MD in tract uncinate fasciculus (right) | No | No |
| **135** | dMRI weighted means | 25542 | Weighted-mean MO in tract acoustic radiation (left) | No | No |
| **135** | dMRI weighted means | 25543 | Weighted-mean MO in tract acoustic radiation (right) | No | No |
| **135** | dMRI weighted means | 25544 | Weighted-mean MO in tract anterior thalamic radiation (left) | No | No |
| **135** | dMRI weighted means | 25545 | Weighted-mean MO in tract anterior thalamic radiation (right) | No | No |
| **135** | dMRI weighted means | 25546 | Weighted-mean MO in tract cingulate gyrus part of cingulum (left) | No | No |
| **135** | dMRI weighted means | 25547 | Weighted-mean MO in tract cingulate gyrus part of cingulum (right) | No | No |
| **135** | dMRI weighted means | 25548 | Weighted-mean MO in tract parahippocampal part of cingulum (left) | No | No |
| **135** | dMRI weighted means | 25549 | Weighted-mean MO in tract parahippocampal part of cingulum (right) | No | No |
| **135** | dMRI weighted means | 25550 | Weighted-mean MO in tract corticospinal tract (left) | No | No |
| **135** | dMRI weighted means | 25551 | Weighted-mean MO in tract corticospinal tract (right) | No | No |
| **135** | dMRI weighted means | 25552 | Weighted-mean MO in tract forceps major | No | No |
| **135** | dMRI weighted means | 25553 | Weighted-mean MO in tract forceps minor | No | No |
| **135** | dMRI weighted means | 25554 | Weighted-mean MO in tract inferior fronto-occipital fasciculus (left) | No | No |
| **135** | dMRI weighted means | 25555 | Weighted-mean MO in tract inferior fronto-occipital fasciculus (right) | No | No |
| **135** | dMRI weighted means | 25556 | Weighted-mean MO in tract inferior longitudinal fasciculus (left) | No | No |
| **135** | dMRI weighted means | 25557 | Weighted-mean MO in tract inferior longitudinal fasciculus (right) | No | No |
| **135** | dMRI weighted means | 25558 | Weighted-mean MO in tract middle cerebellar peduncle | No | No |
| **135** | dMRI weighted means | 25559 | Weighted-mean MO in tract medial lemniscus (left) | No | No |
| **135** | dMRI weighted means | 25560 | Weighted-mean MO in tract medial lemniscus (right) | No | No |
| **135** | dMRI weighted means | 25561 | Weighted-mean MO in tract posterior thalamic radiation (left) | No | No |
| **135** | dMRI weighted means | 25562 | Weighted-mean MO in tract posterior thalamic radiation (right) | No | No |
| **135** | dMRI weighted means | 25563 | Weighted-mean MO in tract superior longitudinal fasciculus (left) | No | No |
| **135** | dMRI weighted means | 25564 | Weighted-mean MO in tract superior longitudinal fasciculus (right) | No | No |
| **135** | dMRI weighted means | 25565 | Weighted-mean MO in tract superior thalamic radiation (left) | No | No |
| **135** | dMRI weighted means | 25566 | Weighted-mean MO in tract superior thalamic radiation (right) | No | No |
| **135** | dMRI weighted means | 25567 | Weighted-mean MO in tract uncinate fasciculus (left) | No | No |
| **135** | dMRI weighted means | 25568 | Weighted-mean MO in tract uncinate fasciculus (right) | No | No |
| **135** | dMRI weighted means | 25569 | Weighted-mean L1 in tract acoustic radiation (left) | No | No |
| **135** | dMRI weighted means | 25570 | Weighted-mean L1 in tract acoustic radiation (right) | No | No |
| **135** | dMRI weighted means | 25571 | Weighted-mean L1 in tract anterior thalamic radiation (left) | No | No |
| **135** | dMRI weighted means | 25572 | Weighted-mean L1 in tract anterior thalamic radiation (right) | No | No |
| **135** | dMRI weighted means | 25573 | Weighted-mean L1 in tract cingulate gyrus part of cingulum (left) | No | No |
| **135** | dMRI weighted means | 25574 | Weighted-mean L1 in tract cingulate gyrus part of cingulum (right) | No | No |
| **135** | dMRI weighted means | 25575 | Weighted-mean L1 in tract parahippocampal part of cingulum (left) | No | No |
| **135** | dMRI weighted means | 25576 | Weighted-mean L1 in tract parahippocampal part of cingulum (right) | No | No |
| **135** | dMRI weighted means | 25577 | Weighted-mean L1 in tract corticospinal tract (left) | No | No |
| **135** | dMRI weighted means | 25578 | Weighted-mean L1 in tract corticospinal tract (right) | No | No |
| **135** | dMRI weighted means | 25579 | Weighted-mean L1 in tract forceps major | No | No |
| **135** | dMRI weighted means | 25580 | Weighted-mean L1 in tract forceps minor | No | No |
| **135** | dMRI weighted means | 25581 | Weighted-mean L1 in tract inferior fronto-occipital fasciculus (left) | No | No |
| **135** | dMRI weighted means | 25582 | Weighted-mean L1 in tract inferior fronto-occipital fasciculus (right) | No | No |
| **135** | dMRI weighted means | 25583 | Weighted-mean L1 in tract inferior longitudinal fasciculus (left) | No | No |
| **135** | dMRI weighted means | 25584 | Weighted-mean L1 in tract inferior longitudinal fasciculus (right) | No | No |
| **135** | dMRI weighted means | 25585 | Weighted-mean L1 in tract middle cerebellar peduncle | No | No |
| **135** | dMRI weighted means | 25586 | Weighted-mean L1 in tract medial lemniscus (left) | No | No |
| **135** | dMRI weighted means | 25587 | Weighted-mean L1 in tract medial lemniscus (right) | No | No |
| **135** | dMRI weighted means | 25588 | Weighted-mean L1 in tract posterior thalamic radiation (left) | No | No |
| **135** | dMRI weighted means | 25589 | Weighted-mean L1 in tract posterior thalamic radiation (right) | No | No |
| **135** | dMRI weighted means | 25590 | Weighted-mean L1 in tract superior longitudinal fasciculus (left) | No | No |
| **135** | dMRI weighted means | 25591 | Weighted-mean L1 in tract superior longitudinal fasciculus (right) | No | No |
| **135** | dMRI weighted means | 25592 | Weighted-mean L1 in tract superior thalamic radiation (left) | No | No |
| **135** | dMRI weighted means | 25593 | Weighted-mean L1 in tract superior thalamic radiation (right) | No | No |
| **135** | dMRI weighted means | 25594 | Weighted-mean L1 in tract uncinate fasciculus (left) | No | No |
| **135** | dMRI weighted means | 25595 | Weighted-mean L1 in tract uncinate fasciculus (right) | No | No |
| **135** | dMRI weighted means | 25596 | Weighted-mean L2 in tract acoustic radiation (left) | No | No |
| **135** | dMRI weighted means | 25597 | Weighted-mean L2 in tract acoustic radiation (right) | No | No |
| **135** | dMRI weighted means | 25598 | Weighted-mean L2 in tract anterior thalamic radiation (left) | No | No |
| **135** | dMRI weighted means | 25599 | Weighted-mean L2 in tract anterior thalamic radiation (right) | No | No |
| **135** | dMRI weighted means | 25600 | Weighted-mean L2 in tract cingulate gyrus part of cingulum (left) | No | No |
| **135** | dMRI weighted means | 25601 | Weighted-mean L2 in tract cingulate gyrus part of cingulum (right) | No | No |
| **135** | dMRI weighted means | 25602 | Weighted-mean L2 in tract parahippocampal part of cingulum (left) | No | No |
| **135** | dMRI weighted means | 25603 | Weighted-mean L2 in tract parahippocampal part of cingulum (right) | No | No |
| **135** | dMRI weighted means | 25604 | Weighted-mean L2 in tract corticospinal tract (left) | No | No |
| **135** | dMRI weighted means | 25605 | Weighted-mean L2 in tract corticospinal tract (right) | No | No |
| **135** | dMRI weighted means | 25606 | Weighted-mean L2 in tract forceps major | No | No |
| **135** | dMRI weighted means | 25607 | Weighted-mean L2 in tract forceps minor | No | No |
| **135** | dMRI weighted means | 25608 | Weighted-mean L2 in tract inferior fronto-occipital fasciculus (left) | No | No |
| **135** | dMRI weighted means | 25609 | Weighted-mean L2 in tract inferior fronto-occipital fasciculus (right) | No | No |
| **135** | dMRI weighted means | 25610 | Weighted-mean L2 in tract inferior longitudinal fasciculus (left) | No | No |
| **135** | dMRI weighted means | 25611 | Weighted-mean L2 in tract inferior longitudinal fasciculus (right) | No | No |
| **135** | dMRI weighted means | 25612 | Weighted-mean L2 in tract middle cerebellar peduncle | No | No |
| **135** | dMRI weighted means | 25613 | Weighted-mean L2 in tract medial lemniscus (left) | No | No |
| **135** | dMRI weighted means | 25614 | Weighted-mean L2 in tract medial lemniscus (right) | No | No |
| **135** | dMRI weighted means | 25615 | Weighted-mean L2 in tract posterior thalamic radiation (left) | No | No |
| **135** | dMRI weighted means | 25616 | Weighted-mean L2 in tract posterior thalamic radiation (right) | No | No |
| **135** | dMRI weighted means | 25617 | Weighted-mean L2 in tract superior longitudinal fasciculus (left) | No | No |
| **135** | dMRI weighted means | 25618 | Weighted-mean L2 in tract superior longitudinal fasciculus (right) | No | No |
| **135** | dMRI weighted means | 25619 | Weighted-mean L2 in tract superior thalamic radiation (left) | No | No |
| **135** | dMRI weighted means | 25620 | Weighted-mean L2 in tract superior thalamic radiation (right) | No | No |
| **135** | dMRI weighted means | 25621 | Weighted-mean L2 in tract uncinate fasciculus (left) | No | No |
| **135** | dMRI weighted means | 25622 | Weighted-mean L2 in tract uncinate fasciculus (right) | No | No |
| **135** | dMRI weighted means | 25623 | Weighted-mean L3 in tract acoustic radiation (left) | No | No |
| **135** | dMRI weighted means | 25624 | Weighted-mean L3 in tract acoustic radiation (right) | No | No |
| **135** | dMRI weighted means | 25625 | Weighted-mean L3 in tract anterior thalamic radiation (left) | No | No |
| **135** | dMRI weighted means | 25626 | Weighted-mean L3 in tract anterior thalamic radiation (right) | No | No |
| **135** | dMRI weighted means | 25627 | Weighted-mean L3 in tract cingulate gyrus part of cingulum (left) | No | No |
| **135** | dMRI weighted means | 25628 | Weighted-mean L3 in tract cingulate gyrus part of cingulum (right) | No | No |
| **135** | dMRI weighted means | 25629 | Weighted-mean L3 in tract parahippocampal part of cingulum (left) | No | No |
| **135** | dMRI weighted means | 25630 | Weighted-mean L3 in tract parahippocampal part of cingulum (right) | No | No |
| **135** | dMRI weighted means | 25631 | Weighted-mean L3 in tract corticospinal tract (left) | No | No |
| **135** | dMRI weighted means | 25632 | Weighted-mean L3 in tract corticospinal tract (right) | No | No |
| **135** | dMRI weighted means | 25633 | Weighted-mean L3 in tract forceps major | No | No |
| **135** | dMRI weighted means | 25634 | Weighted-mean L3 in tract forceps minor | No | No |
| **135** | dMRI weighted means | 25635 | Weighted-mean L3 in tract inferior fronto-occipital fasciculus (left) | No | No |
| **135** | dMRI weighted means | 25636 | Weighted-mean L3 in tract inferior fronto-occipital fasciculus (right) | No | No |
| **135** | dMRI weighted means | 25637 | Weighted-mean L3 in tract inferior longitudinal fasciculus (left) | No | No |
| **135** | dMRI weighted means | 25638 | Weighted-mean L3 in tract inferior longitudinal fasciculus (right) | No | No |
| **135** | dMRI weighted means | 25639 | Weighted-mean L3 in tract middle cerebellar peduncle | No | No |
| **135** | dMRI weighted means | 25640 | Weighted-mean L3 in tract medial lemniscus (left) | No | No |
| **135** | dMRI weighted means | 25641 | Weighted-mean L3 in tract medial lemniscus (right) | No | No |
| **135** | dMRI weighted means | 25642 | Weighted-mean L3 in tract posterior thalamic radiation (left) | No | No |
| **135** | dMRI weighted means | 25643 | Weighted-mean L3 in tract posterior thalamic radiation (right) | No | No |
| **135** | dMRI weighted means | 25644 | Weighted-mean L3 in tract superior longitudinal fasciculus (left) | No | No |
| **135** | dMRI weighted means | 25645 | Weighted-mean L3 in tract superior longitudinal fasciculus (right) | No | No |
| **135** | dMRI weighted means | 25646 | Weighted-mean L3 in tract superior thalamic radiation (left) | No | No |
| **135** | dMRI weighted means | 25647 | Weighted-mean L3 in tract superior thalamic radiation (right) | No | No |
| **135** | dMRI weighted means | 25648 | Weighted-mean L3 in tract uncinate fasciculus (left) | No | No |
| **135** | dMRI weighted means | 25649 | Weighted-mean L3 in tract uncinate fasciculus (right) | No | No |
| **135** | dMRI weighted means | 25650 | Weighted-mean ICVF in tract acoustic radiation (left) | No | No |
| **135** | dMRI weighted means | 25651 | Weighted-mean ICVF in tract acoustic radiation (right) | No | No |
| **135** | dMRI weighted means | 25652 | Weighted-mean ICVF in tract anterior thalamic radiation (left) | No | No |
| **135** | dMRI weighted means | 25653 | Weighted-mean ICVF in tract anterior thalamic radiation (right) | No | No |
| **135** | dMRI weighted means | 25654 | Weighted-mean ICVF in tract cingulate gyrus part of cingulum (left) | No | No |
| **135** | dMRI weighted means | 25655 | Weighted-mean ICVF in tract cingulate gyrus part of cingulum (right) | No | No |
| **135** | dMRI weighted means | 25656 | Weighted-mean ICVF in tract parahippocampal part of cingulum (left) | No | No |
| **135** | dMRI weighted means | 25657 | Weighted-mean ICVF in tract parahippocampal part of cingulum (right) | No | No |
| **135** | dMRI weighted means | 25658 | Weighted-mean ICVF in tract corticospinal tract (left) | No | No |
| **135** | dMRI weighted means | 25659 | Weighted-mean ICVF in tract corticospinal tract (right) | No | No |
| **135** | dMRI weighted means | 25660 | Weighted-mean ICVF in tract forceps major | No | No |
| **135** | dMRI weighted means | 25661 | Weighted-mean ICVF in tract forceps minor | No | No |
| **135** | dMRI weighted means | 25662 | Weighted-mean ICVF in tract inferior fronto-occipital fasciculus (left) | No | No |
| **135** | dMRI weighted means | 25663 | Weighted-mean ICVF in tract inferior fronto-occipital fasciculus (right) | No | No |
| **135** | dMRI weighted means | 25664 | Weighted-mean ICVF in tract inferior longitudinal fasciculus (left) | No | No |
| **135** | dMRI weighted means | 25665 | Weighted-mean ICVF in tract inferior longitudinal fasciculus (right) | No | No |
| **135** | dMRI weighted means | 25666 | Weighted-mean ICVF in tract middle cerebellar peduncle | No | No |
| **135** | dMRI weighted means | 25667 | Weighted-mean ICVF in tract medial lemniscus (left) | No | No |
| **135** | dMRI weighted means | 25668 | Weighted-mean ICVF in tract medial lemniscus (right) | No | No |
| **135** | dMRI weighted means | 25669 | Weighted-mean ICVF in tract posterior thalamic radiation (left) | No | No |
| **135** | dMRI weighted means | 25670 | Weighted-mean ICVF in tract posterior thalamic radiation (right) | No | No |
| **135** | dMRI weighted means | 25671 | Weighted-mean ICVF in tract superior longitudinal fasciculus (left) | No | No |
| **135** | dMRI weighted means | 25672 | Weighted-mean ICVF in tract superior longitudinal fasciculus (right) | No | No |
| **135** | dMRI weighted means | 25673 | Weighted-mean ICVF in tract superior thalamic radiation (left) | No | No |
| **135** | dMRI weighted means | 25674 | Weighted-mean ICVF in tract superior thalamic radiation (right) | No | No |
| **135** | dMRI weighted means | 25675 | Weighted-mean ICVF in tract uncinate fasciculus (left) | No | No |
| **135** | dMRI weighted means | 25676 | Weighted-mean ICVF in tract uncinate fasciculus (right) | No | No |
| **135** | dMRI weighted means | 25677 | Weighted-mean OD in tract acoustic radiation (left) | No | No |
| **135** | dMRI weighted means | 25678 | Weighted-mean OD in tract acoustic radiation (right) | No | No |
| **135** | dMRI weighted means | 25679 | Weighted-mean OD in tract anterior thalamic radiation (left) | No | No |
| **135** | dMRI weighted means | 25680 | Weighted-mean OD in tract anterior thalamic radiation (right) | No | No |
| **135** | dMRI weighted means | 25681 | Weighted-mean OD in tract cingulate gyrus part of cingulum (left) | No | No |
| **135** | dMRI weighted means | 25682 | Weighted-mean OD in tract cingulate gyrus part of cingulum (right) | No | No |
| **135** | dMRI weighted means | 25683 | Weighted-mean OD in tract parahippocampal part of cingulum (left) | No | No |
| **135** | dMRI weighted means | 25684 | Weighted-mean OD in tract parahippocampal part of cingulum (right) | No | No |
| **135** | dMRI weighted means | 25685 | Weighted-mean OD in tract corticospinal tract (left) | No | No |
| **135** | dMRI weighted means | 25686 | Weighted-mean OD in tract corticospinal tract (right) | No | No |
| **135** | dMRI weighted means | 25687 | Weighted-mean OD in tract forceps major | No | No |
| **135** | dMRI weighted means | 25688 | Weighted-mean OD in tract forceps minor | No | No |
| **135** | dMRI weighted means | 25689 | Weighted-mean OD in tract inferior fronto-occipital fasciculus (left) | No | No |
| **135** | dMRI weighted means | 25690 | Weighted-mean OD in tract inferior fronto-occipital fasciculus (right) | No | No |
| **135** | dMRI weighted means | 25691 | Weighted-mean OD in tract inferior longitudinal fasciculus (left) | No | No |
| **135** | dMRI weighted means | 25692 | Weighted-mean OD in tract inferior longitudinal fasciculus (right) | No | No |
| **135** | dMRI weighted means | 25693 | Weighted-mean OD in tract middle cerebellar peduncle | No | No |
| **135** | dMRI weighted means | 25694 | Weighted-mean OD in tract medial lemniscus (left) | No | No |
| **135** | dMRI weighted means | 25695 | Weighted-mean OD in tract medial lemniscus (right) | No | No |
| **135** | dMRI weighted means | 25696 | Weighted-mean OD in tract posterior thalamic radiation (left) | No | No |
| **135** | dMRI weighted means | 25697 | Weighted-mean OD in tract posterior thalamic radiation (right) | No | No |
| **135** | dMRI weighted means | 25698 | Weighted-mean OD in tract superior longitudinal fasciculus (left) | No | No |
| **135** | dMRI weighted means | 25699 | Weighted-mean OD in tract superior longitudinal fasciculus (right) | No | No |
| **135** | dMRI weighted means | 25700 | Weighted-mean OD in tract superior thalamic radiation (left) | No | No |
| **135** | dMRI weighted means | 25701 | Weighted-mean OD in tract superior thalamic radiation (right) | No | No |
| **135** | dMRI weighted means | 25702 | Weighted-mean OD in tract uncinate fasciculus (left) | No | No |
| **135** | dMRI weighted means | 25703 | Weighted-mean OD in tract uncinate fasciculus (right) | No | No |
| **135** | dMRI weighted means | 25704 | Weighted-mean ISOVF in tract acoustic radiation (left) | No | No |
| **135** | dMRI weighted means | 25705 | Weighted-mean ISOVF in tract acoustic radiation (right) | No | No |
| **135** | dMRI weighted means | 25706 | Weighted-mean ISOVF in tract anterior thalamic radiation (left) | No | No |
| **135** | dMRI weighted means | 25707 | Weighted-mean ISOVF in tract anterior thalamic radiation (right) | No | No |
| **135** | dMRI weighted means | 25708 | Weighted-mean ISOVF in tract cingulate gyrus part of cingulum (left) | No | No |
| **135** | dMRI weighted means | 25709 | Weighted-mean ISOVF in tract cingulate gyrus part of cingulum (right) | No | No |
| **135** | dMRI weighted means | 25710 | Weighted-mean ISOVF in tract parahippocampal part of cingulum (left) | No | No |
| **135** | dMRI weighted means | 25711 | Weighted-mean ISOVF in tract parahippocampal part of cingulum (right) | No | No |
| **135** | dMRI weighted means | 25712 | Weighted-mean ISOVF in tract corticospinal tract (left) | No | No |
| **135** | dMRI weighted means | 25713 | Weighted-mean ISOVF in tract corticospinal tract (right) | No | No |
| **135** | dMRI weighted means | 25714 | Weighted-mean ISOVF in tract forceps major | No | No |
| **135** | dMRI weighted means | 25715 | Weighted-mean ISOVF in tract forceps minor | No | No |
| **135** | dMRI weighted means | 25716 | Weighted-mean ISOVF in tract inferior fronto-occipital fasciculus (left) | No | No |
| **135** | dMRI weighted means | 25717 | Weighted-mean ISOVF in tract inferior fronto-occipital fasciculus (right) | No | No |
| **135** | dMRI weighted means | 25718 | Weighted-mean ISOVF in tract inferior longitudinal fasciculus (left) | No | No |
| **135** | dMRI weighted means | 25719 | Weighted-mean ISOVF in tract inferior longitudinal fasciculus (right) | No | No |
| **135** | dMRI weighted means | 25720 | Weighted-mean ISOVF in tract middle cerebellar peduncle | No | No |
| **135** | dMRI weighted means | 25721 | Weighted-mean ISOVF in tract medial lemniscus (left) | No | No |
| **135** | dMRI weighted means | 25722 | Weighted-mean ISOVF in tract medial lemniscus (right) | No | No |
| **135** | dMRI weighted means | 25723 | Weighted-mean ISOVF in tract posterior thalamic radiation (left) | No | No |
| **135** | dMRI weighted means | 25724 | Weighted-mean ISOVF in tract posterior thalamic radiation (right) | No | No |
| **135** | dMRI weighted means | 25725 | Weighted-mean ISOVF in tract superior longitudinal fasciculus (left) | No | No |
| **135** | dMRI weighted means | 25726 | Weighted-mean ISOVF in tract superior longitudinal fasciculus (right) | No | No |
| **135** | dMRI weighted means | 25727 | Weighted-mean ISOVF in tract superior thalamic radiation (left) | No | No |
| **135** | dMRI weighted means | 25728 | Weighted-mean ISOVF in tract superior thalamic radiation (right) | No | No |
| **135** | dMRI weighted means | 25729 | Weighted-mean ISOVF in tract uncinate fasciculus (left) | No | No |
| **135** | dMRI weighted means | 25730 | Weighted-mean ISOVF in tract uncinate fasciculus (right) | No | No |
| **110** | T1 structural brain MRI | 25731 | Discrepancy between T1 brain image and standard-space brain template (linearly-aligned) | No | No |
| **110** | T1 structural brain MRI | 25732 | Discrepancy between T1 brain image and standard-space brain template (nonlinearly-aligned) | No | No |
| **110** | T1 structural brain MRI | 25733 | Amount of warping applied to non-linearly align T1 brain image to standard-space | No | No |
| **110** | T1 structural brain MRI | 25734 | Inverted signal-to-noise ratio in T1 | No | No |
| **110** | T1 structural brain MRI | 25735 | Inverted contrast-to-noise ratio in T1 | No | No |
| **112** | T2-weighted brain MRI | 25736 | Discrepancy between T2 FLAIR brain image and T1 brain image | No | No |
| **107** | Diffusion brain MRI | 25737 | Discrepancy between dMRI brain image and T1 brain image | No | No |
| **109** | Susceptibility weighted brain MRI | 25738 | Discrepancy between SWI brain image and T1 brain image | No | No |
| **111** | Resting functional brain MRI | 25739 | Discrepancy between rfMRI brain image and T1 brain image | No | No |
| **106** | Task functional brain MRI | 25740 | Discrepancy between tfMRI brain image and T1 brain image | No | No |
| **111** | Resting functional brain MRI | 25741 | Mean rfMRI head motion, averaged across space and time points | No | No |
| **106** | Task functional brain MRI | 25742 | Mean tfMRI head motion, averaged across space and time points | No | No |
| **111** | Resting functional brain MRI | 25743 | Inverted temporal signal-to-noise ratio in pre-processed rfMRI | No | No |
| **111** | Resting functional brain MRI | 25744 | Inverted temporal signal-to-noise ratio in artefact-cleaned pre-processed rfMRI | No | No |
| **106** | Task functional brain MRI | 25745 | Inverted temporal signal-to-noise ratio in pre-processed tfMRI | No | No |
| **107** | Diffusion brain MRI | 25746 | Number of dMRI outlier slices detected and corrected | No | No |
| **106** | Task functional brain MRI | 25747 | Eprime advisor file | No | No |
| **106** | Task functional brain MRI | 25748 | Eprime txt file | No | No |
| **106** | Task functional brain MRI | 25749 | Eprime ed2 file | No | No |
| **111** | Resting functional brain MRI | 25750 | rfMRI full correlation matrix, dimension 25 | No | No |
| **111** | Resting functional brain MRI | 25751 | rfMRI full correlation matrix, dimension 100 | No | No |
| **111** | Resting functional brain MRI | 25752 | rfMRI partial correlation matrix, dimension 25 | No | No |
| **111** | Resting functional brain MRI | 25753 | rfMRI partial correlation matrix, dimension 100 | No | No |
| **111** | Resting functional brain MRI | 25754 | rfMRI component amplitudes, dimension 25 | No | No |
| **111** | Resting functional brain MRI | 25755 | rfMRI component amplitudes, dimension 100 | No | No |
| **110** | T1 structural brain MRI | 25756 | Scanner lateral (X) brain position | No | No |
| **110** | T1 structural brain MRI | 25757 | Scanner transverse (Y) brain position | No | No |
| **110** | T1 structural brain MRI | 25758 | Scanner longitudinal (Z) brain position | No | No |
| **110** | T1 structural brain MRI | 25759 | Scanner table position | No | No |
| **106** | Task functional brain MRI | 25761 | 90th percentile of BOLD effect (in group-defined mask) for shapes activation | No | No |
| **106** | Task functional brain MRI | 25762 | 90th percentile of z-statistic (in group-defined mask) for shapes activation | No | No |
| **106** | Task functional brain MRI | 25763 | 90th percentile of BOLD effect (in group-defined mask) for faces activation | No | No |
| **106** | Task functional brain MRI | 25764 | 90th percentile of z-statistic (in group-defined mask) for faces activation | No | No |
| **106** | Task functional brain MRI | 25765 | 90th percentile of BOLD effect (in group-defined mask) for faces-shapes contrast | No | No |
| **106** | Task functional brain MRI | 25766 | 90th percentile of z-statistic (in group-defined mask) for faces-shapes contrast | No | No |
| **106** | Task functional brain MRI | 25767 | 90th percentile of BOLD effect (in group-defined amygdala activation mask) for faces-shapes contrast | No | No |
| **106** | Task functional brain MRI | 25768 | 90th percentile of z-statistic (in group-defined amygdala activation mask) for faces-shapes contrast | No | No |
| **100** | Brain MRI | 25780 | Acquisition protocol phase. | No | No |
| **112** | T2-weighted brain MRI | 25781 | Total volume of white matter hyperintensities (from T1 and T2_FLAIR images) | No | No |
| **1101** | Regional grey matter volumes (FAST) | 25782 | Volume of grey matter in Frontal Pole (left) | No | No |
| **1101** | Regional grey matter volumes (FAST) | 25783 | Volume of grey matter in Frontal Pole (right) | No | No |
| **1101** | Regional grey matter volumes (FAST) | 25784 | Volume of grey matter in Insular Cortex (left) | No | No |
| **1101** | Regional grey matter volumes (FAST) | 25785 | Volume of grey matter in Insular Cortex (right) | No | No |
| **1101** | Regional grey matter volumes (FAST) | 25786 | Volume of grey matter in Superior Frontal Gyrus (left) | No | No |
| **1101** | Regional grey matter volumes (FAST) | 25787 | Volume of grey matter in Superior Frontal Gyrus (right) | No | No |
| **1101** | Regional grey matter volumes (FAST) | 25788 | Volume of grey matter in Middle Frontal Gyrus (left) | No | No |
| **1101** | Regional grey matter volumes (FAST) | 25789 | Volume of grey matter in Middle Frontal Gyrus (right) | No | No |
| **1101** | Regional grey matter volumes (FAST) | 25790 | Volume of grey matter in Inferior Frontal Gyrus, pars triangularis (left) | No | No |
| **1101** | Regional grey matter volumes (FAST) | 25791 | Volume of grey matter in Inferior Frontal Gyrus, pars triangularis (right) | No | No |
| **1101** | Regional grey matter volumes (FAST) | 25792 | Volume of grey matter in Inferior Frontal Gyrus, pars opercularis (left) | No | No |
| **1101** | Regional grey matter volumes (FAST) | 25793 | Volume of grey matter in Inferior Frontal Gyrus, pars opercularis (right) | No | No |
| **1101** | Regional grey matter volumes (FAST) | 25794 | Volume of grey matter in Precentral Gyrus (left) | No | No |
| **1101** | Regional grey matter volumes (FAST) | 25795 | Volume of grey matter in Precentral Gyrus (right) | No | No |
| **1101** | Regional grey matter volumes (FAST) | 25796 | Volume of grey matter in Temporal Pole (left) | No | No |
| **1101** | Regional grey matter volumes (FAST) | 25797 | Volume of grey matter in Temporal Pole (right) | No | No |
| **1101** | Regional grey matter volumes (FAST) | 25798 | Volume of grey matter in Superior Temporal Gyrus, anterior division (left) | No | No |
| **1101** | Regional grey matter volumes (FAST) | 25799 | Volume of grey matter in Superior Temporal Gyrus, anterior division (right) | No | No |
| **1101** | Regional grey matter volumes (FAST) | 25800 | Volume of grey matter in Superior Temporal Gyrus, posterior division (left) | No | No |
| **1101** | Regional grey matter volumes (FAST) | 25801 | Volume of grey matter in Superior Temporal Gyrus, posterior division (right) | No | No |
| **1101** | Regional grey matter volumes (FAST) | 25802 | Volume of grey matter in Middle Temporal Gyrus, anterior division (left) | No | No |
| **1101** | Regional grey matter volumes (FAST) | 25803 | Volume of grey matter in Middle Temporal Gyrus, anterior division (right) | No | No |
| **1101** | Regional grey matter volumes (FAST) | 25804 | Volume of grey matter in Middle Temporal Gyrus, posterior division (left) | No | No |
| **1101** | Regional grey matter volumes (FAST) | 25805 | Volume of grey matter in Middle Temporal Gyrus, posterior division (right) | No | No |
| **1101** | Regional grey matter volumes (FAST) | 25806 | Volume of grey matter in Middle Temporal Gyrus, temporooccipital part (left) | No | No |
| **1101** | Regional grey matter volumes (FAST) | 25807 | Volume of grey matter in Middle Temporal Gyrus, temporooccipital part (right) | No | No |
| **1101** | Regional grey matter volumes (FAST) | 25808 | Volume of grey matter in Inferior Temporal Gyrus, anterior division (left) | No | No |
| **1101** | Regional grey matter volumes (FAST) | 25809 | Volume of grey matter in Inferior Temporal Gyrus, anterior division (right) | No | No |
| **1101** | Regional grey matter volumes (FAST) | 25810 | Volume of grey matter in Inferior Temporal Gyrus, posterior division (left) | No | No |
| **1101** | Regional grey matter volumes (FAST) | 25811 | Volume of grey matter in Inferior Temporal Gyrus, posterior division (right) | No | No |
| **1101** | Regional grey matter volumes (FAST) | 25812 | Volume of grey matter in Inferior Temporal Gyrus, temporooccipital part (left) | No | No |
| **1101** | Regional grey matter volumes (FAST) | 25813 | Volume of grey matter in Inferior Temporal Gyrus, temporooccipital part (right) | No | No |
| **1101** | Regional grey matter volumes (FAST) | 25814 | Volume of grey matter in Postcentral Gyrus (left) | No | No |
| **1101** | Regional grey matter volumes (FAST) | 25815 | Volume of grey matter in Postcentral Gyrus (right) | No | No |
| **1101** | Regional grey matter volumes (FAST) | 25816 | Volume of grey matter in Superior Parietal Lobule (left) | No | No |
| **1101** | Regional grey matter volumes (FAST) | 25817 | Volume of grey matter in Superior Parietal Lobule (right) | No | No |
| **1101** | Regional grey matter volumes (FAST) | 25818 | Volume of grey matter in Supramarginal Gyrus, anterior division (left) | No | No |
| **1101** | Regional grey matter volumes (FAST) | 25819 | Volume of grey matter in Supramarginal Gyrus, anterior division (right) | No | No |
| **1101** | Regional grey matter volumes (FAST) | 25820 | Volume of grey matter in Supramarginal Gyrus, posterior division (left) | No | No |
| **1101** | Regional grey matter volumes (FAST) | 25821 | Volume of grey matter in Supramarginal Gyrus, posterior division (right) | No | No |
| **1101** | Regional grey matter volumes (FAST) | 25822 | Volume of grey matter in Angular Gyrus (left) | No | No |
| **1101** | Regional grey matter volumes (FAST) | 25823 | Volume of grey matter in Angular Gyrus (right) | No | No |
| **1101** | Regional grey matter volumes (FAST) | 25824 | Volume of grey matter in Lateral Occipital Cortex, superior division (left) | No | No |
| **1101** | Regional grey matter volumes (FAST) | 25825 | Volume of grey matter in Lateral Occipital Cortex, superior division (right) | No | No |
| **1101** | Regional grey matter volumes (FAST) | 25826 | Volume of grey matter in Lateral Occipital Cortex, inferior division (left) | No | No |
| **1101** | Regional grey matter volumes (FAST) | 25827 | Volume of grey matter in Lateral Occipital Cortex, inferior division (right) | No | No |
| **1101** | Regional grey matter volumes (FAST) | 25828 | Volume of grey matter in Intracalcarine Cortex (left) | No | No |
| **1101** | Regional grey matter volumes (FAST) | 25829 | Volume of grey matter in Intracalcarine Cortex (right) | No | No |
| **1101** | Regional grey matter volumes (FAST) | 25830 | Volume of grey matter in Frontal Medial Cortex (left) | No | No |
| **1101** | Regional grey matter volumes (FAST) | 25831 | Volume of grey matter in Frontal Medial Cortex (right) | No | No |
| **1101** | Regional grey matter volumes (FAST) | 25832 | Volume of grey matter in Juxtapositional Lobule Cortex (formerly Supplementary Motor Cortex) (left) | No | No |
| **1101** | Regional grey matter volumes (FAST) | 25833 | Volume of grey matter in Juxtapositional Lobule Cortex (formerly Supplementary Motor Cortex) (right) | No | No |
| **1101** | Regional grey matter volumes (FAST) | 25834 | Volume of grey matter in Subcallosal Cortex (left) | No | No |
| **1101** | Regional grey matter volumes (FAST) | 25835 | Volume of grey matter in Subcallosal Cortex (right) | No | No |
| **1101** | Regional grey matter volumes (FAST) | 25836 | Volume of grey matter in Paracingulate Gyrus (left) | No | No |
| **1101** | Regional grey matter volumes (FAST) | 25837 | Volume of grey matter in Paracingulate Gyrus (right) | No | No |
| **1101** | Regional grey matter volumes (FAST) | 25838 | Volume of grey matter in Cingulate Gyrus, anterior division (left) | No | No |
| **1101** | Regional grey matter volumes (FAST) | 25839 | Volume of grey matter in Cingulate Gyrus, anterior division (right) | No | No |
| **1101** | Regional grey matter volumes (FAST) | 25840 | Volume of grey matter in Cingulate Gyrus, posterior division (left) | No | No |
| **1101** | Regional grey matter volumes (FAST) | 25841 | Volume of grey matter in Cingulate Gyrus, posterior division (right) | No | No |
| **1101** | Regional grey matter volumes (FAST) | 25842 | Volume of grey matter in Precuneous Cortex (left) | No | No |
| **1101** | Regional grey matter volumes (FAST) | 25843 | Volume of grey matter in Precuneous Cortex (right) | No | No |
| **1101** | Regional grey matter volumes (FAST) | 25844 | Volume of grey matter in Cuneal Cortex (left) | No | No |
| **1101** | Regional grey matter volumes (FAST) | 25845 | Volume of grey matter in Cuneal Cortex (right) | No | No |
| **1101** | Regional grey matter volumes (FAST) | 25846 | Volume of grey matter in Frontal Orbital Cortex (left) | No | No |
| **1101** | Regional grey matter volumes (FAST) | 25847 | Volume of grey matter in Frontal Orbital Cortex (right) | No | No |
| **1101** | Regional grey matter volumes (FAST) | 25848 | Volume of grey matter in Parahippocampal Gyrus, anterior division (left) | No | No |
| **1101** | Regional grey matter volumes (FAST) | 25849 | Volume of grey matter in Parahippocampal Gyrus, anterior division (right) | No | No |
| **1101** | Regional grey matter volumes (FAST) | 25850 | Volume of grey matter in Parahippocampal Gyrus, posterior division (left) | No | No |
| **1101** | Regional grey matter volumes (FAST) | 25851 | Volume of grey matter in Parahippocampal Gyrus, posterior division (right) | No | No |
| **1101** | Regional grey matter volumes (FAST) | 25852 | Volume of grey matter in Lingual Gyrus (left) | No | No |
| **1101** | Regional grey matter volumes (FAST) | 25853 | Volume of grey matter in Lingual Gyrus (right) | No | No |
| **1101** | Regional grey matter volumes (FAST) | 25854 | Volume of grey matter in Temporal Fusiform Cortex, anterior division (left) | No | No |
| **1101** | Regional grey matter volumes (FAST) | 25855 | Volume of grey matter in Temporal Fusiform Cortex, anterior division (right) | No | No |
| **1101** | Regional grey matter volumes (FAST) | 25856 | Volume of grey matter in Temporal Fusiform Cortex, posterior division (left) | No | No |
| **1101** | Regional grey matter volumes (FAST) | 25857 | Volume of grey matter in Temporal Fusiform Cortex, posterior division (right) | No | No |
| **1101** | Regional grey matter volumes (FAST) | 25858 | Volume of grey matter in Temporal Occipital Fusiform Cortex (left) | No | No |
| **1101** | Regional grey matter volumes (FAST) | 25859 | Volume of grey matter in Temporal Occipital Fusiform Cortex (right) | No | No |
| **1101** | Regional grey matter volumes (FAST) | 25860 | Volume of grey matter in Occipital Fusiform Gyrus (left) | No | No |
| **1101** | Regional grey matter volumes (FAST) | 25861 | Volume of grey matter in Occipital Fusiform Gyrus (right) | No | No |
| **1101** | Regional grey matter volumes (FAST) | 25862 | Volume of grey matter in Frontal Operculum Cortex (left) | No | No |
| **1101** | Regional grey matter volumes (FAST) | 25863 | Volume of grey matter in Frontal Operculum Cortex (right) | No | No |
| **1101** | Regional grey matter volumes (FAST) | 25864 | Volume of grey matter in Central Opercular Cortex (left) | No | No |
| **1101** | Regional grey matter volumes (FAST) | 25865 | Volume of grey matter in Central Opercular Cortex (right) | No | No |
| **1101** | Regional grey matter volumes (FAST) | 25866 | Volume of grey matter in Parietal Operculum Cortex (left) | No | No |
| **1101** | Regional grey matter volumes (FAST) | 25867 | Volume of grey matter in Parietal Operculum Cortex (right) | No | No |
| **1101** | Regional grey matter volumes (FAST) | 25868 | Volume of grey matter in Planum Polare (left) | No | No |
| **1101** | Regional grey matter volumes (FAST) | 25869 | Volume of grey matter in Planum Polare (right) | No | No |
| **1101** | Regional grey matter volumes (FAST) | 25870 | Volume of grey matter in Heschl's Gyrus (includes H1 and H2) (left) | No | No |
| **1101** | Regional grey matter volumes (FAST) | 25871 | Volume of grey matter in Heschl's Gyrus (includes H1 and H2) (right) | No | No |
| **1101** | Regional grey matter volumes (FAST) | 25872 | Volume of grey matter in Planum Temporale (left) | No | No |
| **1101** | Regional grey matter volumes (FAST) | 25873 | Volume of grey matter in Planum Temporale (right) | No | No |
| **1101** | Regional grey matter volumes (FAST) | 25874 | Volume of grey matter in Supracalcarine Cortex (left) | No | No |
| **1101** | Regional grey matter volumes (FAST) | 25875 | Volume of grey matter in Supracalcarine Cortex (right) | No | No |
| **1101** | Regional grey matter volumes (FAST) | 25876 | Volume of grey matter in Occipital Pole (left) | No | No |
| **1101** | Regional grey matter volumes (FAST) | 25877 | Volume of grey matter in Occipital Pole (right) | No | No |
| **1101** | Regional grey matter volumes (FAST) | 25878 | Volume of grey matter in Thalamus (left) | No | No |
| **1101** | Regional grey matter volumes (FAST) | 25879 | Volume of grey matter in Thalamus (right) | No | No |
| **1101** | Regional grey matter volumes (FAST) | 25880 | Volume of grey matter in Caudate (left) | No | No |
| **1101** | Regional grey matter volumes (FAST) | 25881 | Volume of grey matter in Caudate (right) | No | No |
| **1101** | Regional grey matter volumes (FAST) | 25882 | Volume of grey matter in Putamen (left) | No | No |
| **1101** | Regional grey matter volumes (FAST) | 25883 | Volume of grey matter in Putamen (right) | No | No |
| **1101** | Regional grey matter volumes (FAST) | 25884 | Volume of grey matter in Pallidum (left) | No | No |
| **1101** | Regional grey matter volumes (FAST) | 25885 | Volume of grey matter in Pallidum (right) | No | No |
| **1101** | Regional grey matter volumes (FAST) | 25886 | Volume of grey matter in Hippocampus (left) | No | No |
| **1101** | Regional grey matter volumes (FAST) | 25887 | Volume of grey matter in Hippocampus (right) | No | No |
| **1101** | Regional grey matter volumes (FAST) | 25888 | Volume of grey matter in Amygdala (left) | No | No |
| **1101** | Regional grey matter volumes (FAST) | 25889 | Volume of grey matter in Amygdala (right) | No | No |
| **1101** | Regional grey matter volumes (FAST) | 25890 | Volume of grey matter in Ventral Striatum (left) | No | No |
| **1101** | Regional grey matter volumes (FAST) | 25891 | Volume of grey matter in Ventral Striatum (right) | No | No |
| **1101** | Regional grey matter volumes (FAST) | 25892 | Volume of grey matter in Brain-Stem | No | No |
| **1101** | Regional grey matter volumes (FAST) | 25893 | Volume of grey matter in I-IV Cerebellum (left) | No | No |
| **1101** | Regional grey matter volumes (FAST) | 25894 | Volume of grey matter in I-IV Cerebellum (right) | No | No |
| **1101** | Regional grey matter volumes (FAST) | 25895 | Volume of grey matter in V Cerebellum (left) | No | No |
| **1101** | Regional grey matter volumes (FAST) | 25896 | Volume of grey matter in V Cerebellum (right) | No | No |
| **1101** | Regional grey matter volumes (FAST) | 25897 | Volume of grey matter in VI Cerebellum (left) | No | No |
| **1101** | Regional grey matter volumes (FAST) | 25898 | Volume of grey matter in VI Cerebellum (vermis) | No | No |
| **1101** | Regional grey matter volumes (FAST) | 25899 | Volume of grey matter in VI Cerebellum (right) | No | No |
| **1101** | Regional grey matter volumes (FAST) | 25900 | Volume of grey matter in Crus I Cerebellum (left) | No | No |
| **1101** | Regional grey matter volumes (FAST) | 25901 | Volume of grey matter in Crus I Cerebellum (vermis) | No | No |
| **1101** | Regional grey matter volumes (FAST) | 25902 | Volume of grey matter in Crus I Cerebellum (right) | No | No |
| **1101** | Regional grey matter volumes (FAST) | 25903 | Volume of grey matter in Crus II Cerebellum (left) | No | No |
| **1101** | Regional grey matter volumes (FAST) | 25904 | Volume of grey matter in Crus II Cerebellum (vermis) | No | No |
| **1101** | Regional grey matter volumes (FAST) | 25905 | Volume of grey matter in Crus II Cerebellum (right) | No | No |
| **1101** | Regional grey matter volumes (FAST) | 25906 | Volume of grey matter in VIIb Cerebellum (left) | No | No |
| **1101** | Regional grey matter volumes (FAST) | 25907 | Volume of grey matter in VIIb Cerebellum (vermis) | No | No |
| **1101** | Regional grey matter volumes (FAST) | 25908 | Volume of grey matter in VIIb Cerebellum (right) | No | No |
| **1101** | Regional grey matter volumes (FAST) | 25909 | Volume of grey matter in VIIIa Cerebellum (left) | No | No |
| **1101** | Regional grey matter volumes (FAST) | 25910 | Volume of grey matter in VIIIa Cerebellum (vermis) | No | No |
| **1101** | Regional grey matter volumes (FAST) | 25911 | Volume of grey matter in VIIIa Cerebellum (right) | No | No |
| **1101** | Regional grey matter volumes (FAST) | 25912 | Volume of grey matter in VIIIb Cerebellum (left) | No | No |
| **1101** | Regional grey matter volumes (FAST) | 25913 | Volume of grey matter in VIIIb Cerebellum (vermis) | No | No |
| **1101** | Regional grey matter volumes (FAST) | 25914 | Volume of grey matter in VIIIb Cerebellum (right) | No | No |
| **1101** | Regional grey matter volumes (FAST) | 25915 | Volume of grey matter in IX Cerebellum (left) | No | No |
| **1101** | Regional grey matter volumes (FAST) | 25916 | Volume of grey matter in IX Cerebellum (vermis) | No | No |
| **1101** | Regional grey matter volumes (FAST) | 25917 | Volume of grey matter in IX Cerebellum (right) | No | No |
| **1101** | Regional grey matter volumes (FAST) | 25918 | Volume of grey matter in X Cerebellum (left) | No | No |
| **1101** | Regional grey matter volumes (FAST) | 25919 | Volume of grey matter in X Cerebellum (vermis) | No | No |
| **1101** | Regional grey matter volumes (FAST) | 25920 | Volume of grey matter in X Cerebellum (right) | No | No |
| **107** | Diffusion brain MRI | 25921 | Increased search space in eddy current estimation used for dMRI | No | No |
| **107** | Diffusion brain MRI | 25922 | Standard deviation of apparent translation in the Y axis as measured by eddy | No | No |
| **111** | Resting functional brain MRI | 25923 | Echo Time for rfMRI | No | No |
| **106** | Task functional brain MRI | 25924 | Echo Time for tfMRI | No | No |
| **110** | T1 structural brain MRI | 25925 | Intensity scaling for T1 | No | No |
| **112** | T2-weighted brain MRI | 25926 | Intensity scaling for T2_FLAIR | No | No |
| **109** | Susceptibility weighted brain MRI | 25927 | Intensity scaling for SWI | No | No |
| **107** | Diffusion brain MRI | 25928 | Intensity scaling for dMRI | No | No |
| **111** | Resting functional brain MRI | 25929 | Intensity scaling for rfMRI | No | No |
| **106** | Task functional brain MRI | 25930 | Intensity scaling for tfMRI | No | No |
| **76** | Indices of Multiple Deprivation | 26410 | Index of Multiple Deprivation (England) | No | No |
| **76** | Indices of Multiple Deprivation | 26411 | Income score (England) | No | No |
| **76** | Indices of Multiple Deprivation | 26412 | Employment score (England) | No | No |
| **76** | Indices of Multiple Deprivation | 26413 | Health score (England) | No | No |
| **76** | Indices of Multiple Deprivation | 26414 | Education score (England) | No | No |
| **76** | Indices of Multiple Deprivation | 26415 | Housing score (England) | No | No |
| **76** | Indices of Multiple Deprivation | 26416 | Crime score (England) | No | No |
| **76** | Indices of Multiple Deprivation | 26417 | Living environment score (England) | No | No |
| **76** | Indices of Multiple Deprivation | 26418 | Income score (Wales) | No | No |
| **76** | Indices of Multiple Deprivation | 26419 | Employment score (Wales) | No | No |
| **76** | Indices of Multiple Deprivation | 26420 | Health score (Wales) | No | No |
| **76** | Indices of Multiple Deprivation | 26421 | Education score (Wales) | No | No |
| **76** | Indices of Multiple Deprivation | 26422 | Access to services score (Wales) | No | No |
| **76** | Indices of Multiple Deprivation | 26423 | Housing score (Wales) | No | No |
| **76** | Indices of Multiple Deprivation | 26424 | Physical environment score (Wales) | No | No |
| **76** | Indices of Multiple Deprivation | 26425 | Community safety score (Wales) | No | No |
| **76** | Indices of Multiple Deprivation | 26426 | Index of Multiple Deprivation (Wales) | No | No |
| **76** | Indices of Multiple Deprivation | 26427 | Index of Multiple Deprivation (Scotland) | No | No |
| **76** | Indices of Multiple Deprivation | 26428 | Income score (Scotland) | No | No |
| **76** | Indices of Multiple Deprivation | 26429 | Employment score (Scotland) | No | No |
| **76** | Indices of Multiple Deprivation | 26430 | Health score (Scotland) | No | No |
| **76** | Indices of Multiple Deprivation | 26431 | Education score (Scotland) | No | No |
| **76** | Indices of Multiple Deprivation | 26432 | Housing score (Scotland) | No | No |
| **76** | Indices of Multiple Deprivation | 26433 | Access to services score (Scotland) | No | No |
| **76** | Indices of Multiple Deprivation | 26434 | Crime score (Scotland) | No | No |
| **110** | T1 structural brain MRI | 26500 | T2-FLAIR used (in addition to T1) to run FreeSurfer | No | No |
| **190** | Freesurfer ASEG | 26501 | Mean intensity of 3rd-Ventricle (whole brain) | No | No |
| **190** | Freesurfer ASEG | 26502 | Mean intensity of 4th-Ventricle (whole brain) | No | No |
| **190** | Freesurfer ASEG | 26503 | Mean intensity of 5th-Ventricle (whole brain) | No | No |
| **190** | Freesurfer ASEG | 26504 | Mean intensity of Brain-Stem (whole brain) | No | No |
| **190** | Freesurfer ASEG | 26505 | Mean intensity of CSF (whole brain) | No | No |
| **190** | Freesurfer ASEG | 26506 | Mean intensity of WM-hypointensities (whole brain) | No | No |
| **190** | Freesurfer ASEG | 26507 | Mean intensity of non-WM-hypointensities (whole brain) | No | No |
| **190** | Freesurfer ASEG | 26508 | Mean intensity of Optic-Chiasm (whole brain) | No | No |
| **190** | Freesurfer ASEG | 26509 | Mean intensity of CC-Posterior (whole brain) | No | No |
| **190** | Freesurfer ASEG | 26510 | Mean intensity of CC-Mid-Posterior (whole brain) | No | No |
| **190** | Freesurfer ASEG | 26511 | Mean intensity of CC-Central (whole brain) | No | No |
| **190** | Freesurfer ASEG | 26512 | Mean intensity of CC-Mid-Anterior (whole brain) | No | No |
| **190** | Freesurfer ASEG | 26513 | Mean intensity of CC-Anterior (whole brain) | No | No |
| **190** | Freesurfer ASEG | 26514 | Volume of BrainSeg (whole brain) | No | No |
| **190** | Freesurfer ASEG | 26515 | Volume of BrainSegNotVent (whole brain) | No | No |
| **190** | Freesurfer ASEG | 26516 | Volume of BrainSegNotVentSurf (whole brain) | No | No |
| **190** | Freesurfer ASEG | 26517 | Volume of SubCortGray (whole brain) | No | No |
| **190** | Freesurfer ASEG | 26518 | Volume of TotalGray (whole brain) | No | No |
| **190** | Freesurfer ASEG | 26519 | Volume of SupraTentorial (whole brain) | No | No |
| **190** | Freesurfer ASEG | 26520 | Volume of SupraTentorialNotVent (whole brain) | No | No |
| **190** | Freesurfer ASEG | 26521 | Volume of EstimatedTotalIntraCranial (whole brain) | No | No |
| **190** | Freesurfer ASEG | 26522 | Volume of VentricleChoroid (whole brain) | No | No |
| **190** | Freesurfer ASEG | 26523 | Volume of 3rd-Ventricle (whole brain) | No | No |
| **190** | Freesurfer ASEG | 26524 | Volume of 4th-Ventricle (whole brain) | No | No |
| **190** | Freesurfer ASEG | 26525 | Volume of 5th-Ventricle (whole brain) | No | No |
| **190** | Freesurfer ASEG | 26526 | Volume of Brain-Stem (whole brain) | No | No |
| **190** | Freesurfer ASEG | 26527 | Volume of CSF (whole brain) | No | No |
| **190** | Freesurfer ASEG | 26528 | Volume of WM-hypointensities (whole brain) | No | No |
| **190** | Freesurfer ASEG | 26529 | Volume of non-WM-hypointensities (whole brain) | No | No |
| **190** | Freesurfer ASEG | 26530 | Volume of Optic-Chiasm (whole brain) | No | No |
| **190** | Freesurfer ASEG | 26531 | Volume of CC-Posterior (whole brain) | No | No |
| **190** | Freesurfer ASEG | 26532 | Volume of CC-Mid-Posterior (whole brain) | No | No |
| **190** | Freesurfer ASEG | 26533 | Volume of CC-Central (whole brain) | No | No |
| **190** | Freesurfer ASEG | 26534 | Volume of CC-Mid-Anterior (whole brain) | No | No |
| **190** | Freesurfer ASEG | 26535 | Volume of CC-Anterior (whole brain) | No | No |
| **190** | Freesurfer ASEG | 26536 | Volume-ratio of BrainSegVol-to-eTIV (whole brain) | No | No |
| **190** | Freesurfer ASEG | 26537 | Volume-ratio of MaskVol-to-eTIV (whole brain) | No | No |
| **190** | Freesurfer ASEG | 26538 | Mean intensity of Lateral-Ventricle (left hemisphere) | No | No |
| **190** | Freesurfer ASEG | 26539 | Mean intensity of Inf-Lat-Vent (left hemisphere) | No | No |
| **190** | Freesurfer ASEG | 26540 | Mean intensity of Cerebellum-White-Matter (left hemisphere) | No | No |
| **190** | Freesurfer ASEG | 26541 | Mean intensity of Cerebellum-Cortex (left hemisphere) | No | No |
| **190** | Freesurfer ASEG | 26542 | Mean intensity of Thalamus-Proper (left hemisphere) | No | No |
| **190** | Freesurfer ASEG | 26543 | Mean intensity of Caudate (left hemisphere) | No | No |
| **190** | Freesurfer ASEG | 26544 | Mean intensity of Putamen (left hemisphere) | No | No |
| **190** | Freesurfer ASEG | 26545 | Mean intensity of Pallidum (left hemisphere) | No | No |
| **190** | Freesurfer ASEG | 26546 | Mean intensity of Hippocampus (left hemisphere) | No | No |
| **190** | Freesurfer ASEG | 26547 | Mean intensity of Amygdala (left hemisphere) | No | No |
| **190** | Freesurfer ASEG | 26548 | Mean intensity of Accumbens-area (left hemisphere) | No | No |
| **190** | Freesurfer ASEG | 26549 | Mean intensity of VentralDC (left hemisphere) | No | No |
| **190** | Freesurfer ASEG | 26550 | Mean intensity of vessel (left hemisphere) | No | No |
| **190** | Freesurfer ASEG | 26551 | Mean intensity of choroid-plexus (left hemisphere) | No | No |
| **190** | Freesurfer ASEG | 26552 | Volume of Cortex (left hemisphere) | No | No |
| **190** | Freesurfer ASEG | 26553 | Volume of CerebralWhiteMatter (left hemisphere) | No | No |
| **190** | Freesurfer ASEG | 26554 | Volume of Lateral-Ventricle (left hemisphere) | No | No |
| **190** | Freesurfer ASEG | 26555 | Volume of Inf-Lat-Vent (left hemisphere) | No | No |
| **190** | Freesurfer ASEG | 26556 | Volume of Cerebellum-White-Matter (left hemisphere) | No | No |
| **190** | Freesurfer ASEG | 26557 | Volume of Cerebellum-Cortex (left hemisphere) | No | No |
| **190** | Freesurfer ASEG | 26558 | Volume of Thalamus-Proper (left hemisphere) | No | No |
| **190** | Freesurfer ASEG | 26559 | Volume of Caudate (left hemisphere) | No | No |
| **190** | Freesurfer ASEG | 26560 | Volume of Putamen (left hemisphere) | No | No |
| **190** | Freesurfer ASEG | 26561 | Volume of Pallidum (left hemisphere) | No | No |
| **190** | Freesurfer ASEG | 26562 | Volume of Hippocampus (left hemisphere) | No | No |
| **190** | Freesurfer ASEG | 26563 | Volume of Amygdala (left hemisphere) | No | No |
| **190** | Freesurfer ASEG | 26564 | Volume of Accumbens-area (left hemisphere) | No | No |
| **190** | Freesurfer ASEG | 26565 | Volume of VentralDC (left hemisphere) | No | No |
| **190** | Freesurfer ASEG | 26566 | Volume of vessel (left hemisphere) | No | No |
| **190** | Freesurfer ASEG | 26567 | Volume of choroid-plexus (left hemisphere) | No | No |
| **190** | Freesurfer ASEG | 26568 | Number of HolesBeforeFixing (left hemisphere) | No | No |
| **190** | Freesurfer ASEG | 26569 | Mean intensity of Lateral-Ventricle (right hemisphere) | No | No |
| **190** | Freesurfer ASEG | 26570 | Mean intensity of Inf-Lat-Vent (right hemisphere) | No | No |
| **190** | Freesurfer ASEG | 26571 | Mean intensity of Cerebellum-White-Matter (right hemisphere) | No | No |
| **190** | Freesurfer ASEG | 26572 | Mean intensity of Cerebellum-Cortex (right hemisphere) | No | No |
| **190** | Freesurfer ASEG | 26573 | Mean intensity of Thalamus-Proper (right hemisphere) | No | No |
| **190** | Freesurfer ASEG | 26574 | Mean intensity of Caudate (right hemisphere) | No | No |
| **190** | Freesurfer ASEG | 26575 | Mean intensity of Putamen (right hemisphere) | No | No |
| **190** | Freesurfer ASEG | 26576 | Mean intensity of Pallidum (right hemisphere) | No | No |
| **190** | Freesurfer ASEG | 26577 | Mean intensity of Hippocampus (right hemisphere) | No | No |
| **190** | Freesurfer ASEG | 26578 | Mean intensity of Amygdala (right hemisphere) | No | No |
| **190** | Freesurfer ASEG | 26579 | Mean intensity of Accumbens-area (right hemisphere) | No | No |
| **190** | Freesurfer ASEG | 26580 | Mean intensity of VentralDC (right hemisphere) | No | No |
| **190** | Freesurfer ASEG | 26581 | Mean intensity of vessel (right hemisphere) | No | No |
| **190** | Freesurfer ASEG | 26582 | Mean intensity of choroid-plexus (right hemisphere) | No | No |
| **190** | Freesurfer ASEG | 26583 | Volume of Cortex (right hemisphere) | No | No |
| **190** | Freesurfer ASEG | 26584 | Volume of CerebralWhiteMatter (right hemisphere) | No | No |
| **190** | Freesurfer ASEG | 26585 | Volume of Lateral-Ventricle (right hemisphere) | No | No |
| **190** | Freesurfer ASEG | 26586 | Volume of Inf-Lat-Vent (right hemisphere) | No | No |
| **190** | Freesurfer ASEG | 26587 | Volume of Cerebellum-White-Matter (right hemisphere) | No | No |
| **190** | Freesurfer ASEG | 26588 | Volume of Cerebellum-Cortex (right hemisphere) | No | No |
| **190** | Freesurfer ASEG | 26589 | Volume of Thalamus-Proper (right hemisphere) | No | No |
| **190** | Freesurfer ASEG | 26590 | Volume of Caudate (right hemisphere) | No | No |
| **190** | Freesurfer ASEG | 26591 | Volume of Putamen (right hemisphere) | No | No |
| **190** | Freesurfer ASEG | 26592 | Volume of Pallidum (right hemisphere) | No | No |
| **190** | Freesurfer ASEG | 26593 | Volume of Hippocampus (right hemisphere) | No | No |
| **190** | Freesurfer ASEG | 26594 | Volume of Amygdala (right hemisphere) | No | No |
| **190** | Freesurfer ASEG | 26595 | Volume of Accumbens-area (right hemisphere) | No | No |
| **190** | Freesurfer ASEG | 26596 | Volume of VentralDC (right hemisphere) | No | No |
| **190** | Freesurfer ASEG | 26597 | Volume of vessel (right hemisphere) | No | No |
| **190** | Freesurfer ASEG | 26598 | Volume of choroid-plexus (right hemisphere) | No | No |
| **190** | Freesurfer ASEG | 26599 | Number of HolesBeforeFixing (right hemisphere) | No | No |
| **191** | Freesurfer subsegmentation | 26600 | Volume of Lateral-nucleus (left hemisphere) | No | No |
| **191** | Freesurfer subsegmentation | 26601 | Volume of Basal-nucleus (left hemisphere) | No | No |
| **191** | Freesurfer subsegmentation | 26602 | Volume of Accessory-Basal-nucleus (left hemisphere) | No | No |
| **191** | Freesurfer subsegmentation | 26603 | Volume of Anterior-amygdaloid-area-AAA (left hemisphere) | No | No |
| **191** | Freesurfer subsegmentation | 26604 | Volume of Central-nucleus (left hemisphere) | No | No |
| **191** | Freesurfer subsegmentation | 26605 | Volume of Medial-nucleus (left hemisphere) | No | No |
| **191** | Freesurfer subsegmentation | 26606 | Volume of Cortical-nucleus (left hemisphere) | No | No |
| **191** | Freesurfer subsegmentation | 26607 | Volume of Corticoamygdaloid-transitio (left hemisphere) | No | No |
| **191** | Freesurfer subsegmentation | 26608 | Volume of Paralaminar-nucleus (left hemisphere) | No | No |
| **191** | Freesurfer subsegmentation | 26609 | Volume of Whole-amygdala (left hemisphere) | No | No |
| **191** | Freesurfer subsegmentation | 26610 | Volume of Lateral-nucleus (right hemisphere) | No | No |
| **191** | Freesurfer subsegmentation | 26611 | Volume of Basal-nucleus (right hemisphere) | No | No |
| **191** | Freesurfer subsegmentation | 26612 | Volume of Accessory-Basal-nucleus (right hemisphere) | No | No |
| **191** | Freesurfer subsegmentation | 26613 | Volume of Anterior-amygdaloid-area-AAA (right hemisphere) | No | No |
| **191** | Freesurfer subsegmentation | 26614 | Volume of Central-nucleus (right hemisphere) | No | No |
| **191** | Freesurfer subsegmentation | 26615 | Volume of Medial-nucleus (right hemisphere) | No | No |
| **191** | Freesurfer subsegmentation | 26616 | Volume of Cortical-nucleus (right hemisphere) | No | No |
| **191** | Freesurfer subsegmentation | 26617 | Volume of Corticoamygdaloid-transitio (right hemisphere) | No | No |
| **191** | Freesurfer subsegmentation | 26618 | Volume of Paralaminar-nucleus (right hemisphere) | No | No |
| **191** | Freesurfer subsegmentation | 26619 | Volume of Whole-amygdala (right hemisphere) | No | No |
| **191** | Freesurfer subsegmentation | 26620 | Volume of Hippocampal-tail (left hemisphere) | No | No |
| **191** | Freesurfer subsegmentation | 26621 | Volume of subiculum-body (left hemisphere) | No | No |
| **191** | Freesurfer subsegmentation | 26622 | Volume of CA1-body (left hemisphere) | No | No |
| **191** | Freesurfer subsegmentation | 26623 | Volume of subiculum-head (left hemisphere) | No | No |
| **191** | Freesurfer subsegmentation | 26624 | Volume of hippocampal-fissure (left hemisphere) | No | No |
| **191** | Freesurfer subsegmentation | 26625 | Volume of presubiculum-head (left hemisphere) | No | No |
| **191** | Freesurfer subsegmentation | 26626 | Volume of CA1-head (left hemisphere) | No | No |
| **191** | Freesurfer subsegmentation | 26627 | Volume of presubiculum-body (left hemisphere) | No | No |
| **191** | Freesurfer subsegmentation | 26628 | Volume of parasubiculum (left hemisphere) | No | No |
| **191** | Freesurfer subsegmentation | 26629 | Volume of molecular-layer-HP-head (left hemisphere) | No | No |
| **191** | Freesurfer subsegmentation | 26630 | Volume of molecular-layer-HP-body (left hemisphere) | No | No |
| **191** | Freesurfer subsegmentation | 26631 | Volume of GC-ML-DG-head (left hemisphere) | No | No |
| **191** | Freesurfer subsegmentation | 26632 | Volume of CA3-body (left hemisphere) | No | No |
| **191** | Freesurfer subsegmentation | 26633 | Volume of GC-ML-DG-body (left hemisphere) | No | No |
| **191** | Freesurfer subsegmentation | 26634 | Volume of CA4-head (left hemisphere) | No | No |
| **191** | Freesurfer subsegmentation | 26635 | Volume of CA4-body (left hemisphere) | No | No |
| **191** | Freesurfer subsegmentation | 26636 | Volume of fimbria (left hemisphere) | No | No |
| **191** | Freesurfer subsegmentation | 26637 | Volume of CA3-head (left hemisphere) | No | No |
| **191** | Freesurfer subsegmentation | 26638 | Volume of HATA (left hemisphere) | No | No |
| **191** | Freesurfer subsegmentation | 26639 | Volume of Whole-hippocampal-body (left hemisphere) | No | No |
| **191** | Freesurfer subsegmentation | 26640 | Volume of Whole-hippocampal-head (left hemisphere) | No | No |
| **191** | Freesurfer subsegmentation | 26641 | Volume of Whole-hippocampus (left hemisphere) | No | No |
| **191** | Freesurfer subsegmentation | 26642 | Volume of Hippocampal-tail (right hemisphere) | No | No |
| **191** | Freesurfer subsegmentation | 26643 | Volume of subiculum-body (right hemisphere) | No | No |
| **191** | Freesurfer subsegmentation | 26644 | Volume of CA1-body (right hemisphere) | No | No |
| **191** | Freesurfer subsegmentation | 26645 | Volume of subiculum-head (right hemisphere) | No | No |
| **191** | Freesurfer subsegmentation | 26646 | Volume of hippocampal-fissure (right hemisphere) | No | No |
| **191** | Freesurfer subsegmentation | 26647 | Volume of presubiculum-head (right hemisphere) | No | No |
| **191** | Freesurfer subsegmentation | 26648 | Volume of CA1-head (right hemisphere) | No | No |
| **191** | Freesurfer subsegmentation | 26649 | Volume of presubiculum-body (right hemisphere) | No | No |
| **191** | Freesurfer subsegmentation | 26650 | Volume of parasubiculum (right hemisphere) | No | No |
| **191** | Freesurfer subsegmentation | 26651 | Volume of molecular-layer-HP-head (right hemisphere) | No | No |
| **191** | Freesurfer subsegmentation | 26652 | Volume of molecular-layer-HP-body (right hemisphere) | No | No |
| **191** | Freesurfer subsegmentation | 26653 | Volume of GC-ML-DG-head (right hemisphere) | No | No |
| **191** | Freesurfer subsegmentation | 26654 | Volume of CA3-body (right hemisphere) | No | No |
| **191** | Freesurfer subsegmentation | 26655 | Volume of GC-ML-DG-body (right hemisphere) | No | No |
| **191** | Freesurfer subsegmentation | 26656 | Volume of CA4-head (right hemisphere) | No | No |
| **191** | Freesurfer subsegmentation | 26657 | Volume of CA4-body (right hemisphere) | No | No |
| **191** | Freesurfer subsegmentation | 26658 | Volume of fimbria (right hemisphere) | No | No |
| **191** | Freesurfer subsegmentation | 26659 | Volume of CA3-head (right hemisphere) | No | No |
| **191** | Freesurfer subsegmentation | 26660 | Volume of HATA (right hemisphere) | No | No |
| **191** | Freesurfer subsegmentation | 26661 | Volume of Whole-hippocampal-body (right hemisphere) | No | No |
| **191** | Freesurfer subsegmentation | 26662 | Volume of Whole-hippocampal-head (right hemisphere) | No | No |
| **191** | Freesurfer subsegmentation | 26663 | Volume of Whole-hippocampus (right hemisphere) | No | No |
| **191** | Freesurfer subsegmentation | 26664 | Volume of MGN (left hemisphere) | No | No |
| **191** | Freesurfer subsegmentation | 26665 | Volume of LGN (left hemisphere) | No | No |
| **191** | Freesurfer subsegmentation | 26666 | Volume of PuI (left hemisphere) | No | No |
| **191** | Freesurfer subsegmentation | 26667 | Volume of PuM (left hemisphere) | No | No |
| **191** | Freesurfer subsegmentation | 26668 | Volume of L-Sg (left hemisphere) | No | No |
| **191** | Freesurfer subsegmentation | 26669 | Volume of VPL (left hemisphere) | No | No |
| **191** | Freesurfer subsegmentation | 26670 | Volume of CM (left hemisphere) | No | No |
| **191** | Freesurfer subsegmentation | 26671 | Volume of VLa (left hemisphere) | No | No |
| **191** | Freesurfer subsegmentation | 26672 | Volume of PuA (left hemisphere) | No | No |
| **191** | Freesurfer subsegmentation | 26673 | Volume of MDm (left hemisphere) | No | No |
| **191** | Freesurfer subsegmentation | 26674 | Volume of Pf (left hemisphere) | No | No |
| **191** | Freesurfer subsegmentation | 26675 | Volume of VAmc (left hemisphere) | No | No |
| **191** | Freesurfer subsegmentation | 26676 | Volume of MDl (left hemisphere) | No | No |
| **191** | Freesurfer subsegmentation | 26677 | Volume of CeM (left hemisphere) | No | No |
| **191** | Freesurfer subsegmentation | 26678 | Volume of VA (left hemisphere) | No | No |
| **191** | Freesurfer subsegmentation | 26679 | Volume of MV(Re) (left hemisphere) | No | No |
| **191** | Freesurfer subsegmentation | 26680 | Volume of VM (left hemisphere) | No | No |
| **191** | Freesurfer subsegmentation | 26681 | Volume of CL (left hemisphere) | No | No |
| **191** | Freesurfer subsegmentation | 26682 | Volume of PuL (left hemisphere) | No | No |
| **191** | Freesurfer subsegmentation | 26683 | Volume of Pt (left hemisphere) | No | No |
| **191** | Freesurfer subsegmentation | 26684 | Volume of AV (left hemisphere) | No | No |
| **191** | Freesurfer subsegmentation | 26685 | Volume of Pc (left hemisphere) | No | No |
| **191** | Freesurfer subsegmentation | 26686 | Volume of VLp (left hemisphere) | No | No |
| **191** | Freesurfer subsegmentation | 26687 | Volume of LP (left hemisphere) | No | No |
| **191** | Freesurfer subsegmentation | 26688 | Volume of LGN (right hemisphere) | No | No |
| **191** | Freesurfer subsegmentation | 26689 | Volume of MGN (right hemisphere) | No | No |
| **191** | Freesurfer subsegmentation | 26690 | Volume of PuI (right hemisphere) | No | No |
| **191** | Freesurfer subsegmentation | 26691 | Volume of PuM (right hemisphere) | No | No |
| **191** | Freesurfer subsegmentation | 26692 | Volume of L-Sg (right hemisphere) | No | No |
| **191** | Freesurfer subsegmentation | 26693 | Volume of VPL (right hemisphere) | No | No |
| **191** | Freesurfer subsegmentation | 26694 | Volume of CM (right hemisphere) | No | No |
| **191** | Freesurfer subsegmentation | 26695 | Volume of VLa (right hemisphere) | No | No |
| **191** | Freesurfer subsegmentation | 26696 | Volume of PuA (right hemisphere) | No | No |
| **191** | Freesurfer subsegmentation | 26697 | Volume of MDm (right hemisphere) | No | No |
| **191** | Freesurfer subsegmentation | 26698 | Volume of Pf (right hemisphere) | No | No |
| **191** | Freesurfer subsegmentation | 26699 | Volume of VAmc (right hemisphere) | No | No |
| **191** | Freesurfer subsegmentation | 26700 | Volume of MDl (right hemisphere) | No | No |
| **191** | Freesurfer subsegmentation | 26701 | Volume of VA (right hemisphere) | No | No |
| **191** | Freesurfer subsegmentation | 26702 | Volume of MV(Re) (right hemisphere) | No | No |
| **191** | Freesurfer subsegmentation | 26703 | Volume of CeM (right hemisphere) | No | No |
| **191** | Freesurfer subsegmentation | 26704 | Volume of VM (right hemisphere) | No | No |
| **191** | Freesurfer subsegmentation | 26705 | Volume of PuL (right hemisphere) | No | No |
| **191** | Freesurfer subsegmentation | 26706 | Volume of CL (right hemisphere) | No | No |
| **191** | Freesurfer subsegmentation | 26707 | Volume of VLp (right hemisphere) | No | No |
| **191** | Freesurfer subsegmentation | 26708 | Volume of Pc (right hemisphere) | No | No |
| **191** | Freesurfer subsegmentation | 26709 | Volume of Pt (right hemisphere) | No | No |
| **191** | Freesurfer subsegmentation | 26710 | Volume of AV (right hemisphere) | No | No |
| **191** | Freesurfer subsegmentation | 26711 | Volume of LP (right hemisphere) | No | No |
| **191** | Freesurfer subsegmentation | 26712 | Volume of LD (left hemisphere) | No | No |
| **191** | Freesurfer subsegmentation | 26713 | Volume of LD (right hemisphere) | No | No |
| **191** | Freesurfer subsegmentation | 26714 | Volume of Whole-thalamus (left hemisphere) | No | No |
| **191** | Freesurfer subsegmentation | 26715 | Volume of Whole-thalamus (right hemisphere) | No | No |
| **191** | Freesurfer subsegmentation | 26716 | Volume of Medulla (whole brain) | No | No |
| **191** | Freesurfer subsegmentation | 26717 | Volume of Pons (whole brain) | No | No |
| **191** | Freesurfer subsegmentation | 26718 | Volume of SCP (whole brain) | No | No |
| **191** | Freesurfer subsegmentation | 26719 | Volume of Midbrain (whole brain) | No | No |
| **191** | Freesurfer subsegmentation | 26720 | Volume of Whole-brainstem (whole brain) | No | No |
| **192** | Freesurfer desikan white | 26721 | Area of TotalSurface (left hemisphere) | No | No |
| **192** | Freesurfer desikan white | 26722 | Area of bankssts (left hemisphere) | No | No |
| **192** | Freesurfer desikan white | 26723 | Area of caudalanteriorcingulate (left hemisphere) | No | No |
| **192** | Freesurfer desikan white | 26724 | Area of caudalmiddlefrontal (left hemisphere) | No | No |
| **192** | Freesurfer desikan white | 26725 | Area of cuneus (left hemisphere) | No | No |
| **192** | Freesurfer desikan white | 26726 | Area of entorhinal (left hemisphere) | No | No |
| **192** | Freesurfer desikan white | 26727 | Area of fusiform (left hemisphere) | No | No |
| **192** | Freesurfer desikan white | 26728 | Area of inferiorparietal (left hemisphere) | No | No |
| **192** | Freesurfer desikan white | 26729 | Area of inferiortemporal (left hemisphere) | No | No |
| **192** | Freesurfer desikan white | 26730 | Area of isthmuscingulate (left hemisphere) | No | No |
| **192** | Freesurfer desikan white | 26731 | Area of lateraloccipital (left hemisphere) | No | No |
| **192** | Freesurfer desikan white | 26732 | Area of lateralorbitofrontal (left hemisphere) | No | No |
| **192** | Freesurfer desikan white | 26733 | Area of lingual (left hemisphere) | No | No |
| **192** | Freesurfer desikan white | 26734 | Area of medialorbitofrontal (left hemisphere) | No | No |
| **192** | Freesurfer desikan white | 26735 | Area of middletemporal (left hemisphere) | No | No |
| **192** | Freesurfer desikan white | 26736 | Area of parahippocampal (left hemisphere) | No | No |
| **192** | Freesurfer desikan white | 26737 | Area of paracentral (left hemisphere) | No | No |
| **192** | Freesurfer desikan white | 26738 | Area of parsopercularis (left hemisphere) | No | No |
| **192** | Freesurfer desikan white | 26739 | Area of parsorbitalis (left hemisphere) | No | No |
| **192** | Freesurfer desikan white | 26740 | Area of parstriangularis (left hemisphere) | No | No |
| **192** | Freesurfer desikan white | 26741 | Area of pericalcarine (left hemisphere) | No | No |
| **192** | Freesurfer desikan white | 26742 | Area of postcentral (left hemisphere) | No | No |
| **192** | Freesurfer desikan white | 26743 | Area of posteriorcingulate (left hemisphere) | No | No |
| **192** | Freesurfer desikan white | 26744 | Area of precentral (left hemisphere) | No | No |
| **192** | Freesurfer desikan white | 26745 | Area of precuneus (left hemisphere) | No | No |
| **192** | Freesurfer desikan white | 26746 | Area of rostralanteriorcingulate (left hemisphere) | No | No |
| **192** | Freesurfer desikan white | 26747 | Area of rostralmiddlefrontal (left hemisphere) | No | No |
| **192** | Freesurfer desikan white | 26748 | Area of superiorfrontal (left hemisphere) | No | No |
| **192** | Freesurfer desikan white | 26749 | Area of superiorparietal (left hemisphere) | No | No |
| **192** | Freesurfer desikan white | 26750 | Area of superiortemporal (left hemisphere) | No | No |
| **192** | Freesurfer desikan white | 26751 | Area of supramarginal (left hemisphere) | No | No |
| **192** | Freesurfer desikan white | 26752 | Area of frontalpole (left hemisphere) | No | No |
| **192** | Freesurfer desikan white | 26753 | Area of transversetemporal (left hemisphere) | No | No |
| **192** | Freesurfer desikan white | 26754 | Area of insula (left hemisphere) | No | No |
| **192** | Freesurfer desikan white | 26755 | Mean thickness of GlobalMeanMean thickness (left hemisphere) | No | No |
| **192** | Freesurfer desikan white | 26756 | Mean thickness of bankssts (left hemisphere) | No | No |
| **192** | Freesurfer desikan white | 26757 | Mean thickness of caudalanteriorcingulate (left hemisphere) | No | No |
| **192** | Freesurfer desikan white | 26758 | Mean thickness of caudalmiddlefrontal (left hemisphere) | No | No |
| **192** | Freesurfer desikan white | 26759 | Mean thickness of cuneus (left hemisphere) | No | No |
| **192** | Freesurfer desikan white | 26760 | Mean thickness of entorhinal (left hemisphere) | No | No |
| **192** | Freesurfer desikan white | 26761 | Mean thickness of fusiform (left hemisphere) | No | No |
| **192** | Freesurfer desikan white | 26762 | Mean thickness of inferiorparietal (left hemisphere) | No | No |
| **192** | Freesurfer desikan white | 26763 | Mean thickness of inferiortemporal (left hemisphere) | No | No |
| **192** | Freesurfer desikan white | 26764 | Mean thickness of isthmuscingulate (left hemisphere) | No | No |
| **192** | Freesurfer desikan white | 26765 | Mean thickness of lateraloccipital (left hemisphere) | No | No |
| **192** | Freesurfer desikan white | 26766 | Mean thickness of lateralorbitofrontal (left hemisphere) | No | No |
| **192** | Freesurfer desikan white | 26767 | Mean thickness of lingual (left hemisphere) | No | No |
| **192** | Freesurfer desikan white | 26768 | Mean thickness of medialorbitofrontal (left hemisphere) | No | No |
| **192** | Freesurfer desikan white | 26769 | Mean thickness of middletemporal (left hemisphere) | No | No |
| **192** | Freesurfer desikan white | 26770 | Mean thickness of parahippocampal (left hemisphere) | No | No |
| **192** | Freesurfer desikan white | 26771 | Mean thickness of paracentral (left hemisphere) | No | No |
| **192** | Freesurfer desikan white | 26772 | Mean thickness of parsopercularis (left hemisphere) | No | No |
| **192** | Freesurfer desikan white | 26773 | Mean thickness of parsorbitalis (left hemisphere) | No | No |
| **192** | Freesurfer desikan white | 26774 | Mean thickness of parstriangularis (left hemisphere) | No | No |
| **192** | Freesurfer desikan white | 26775 | Mean thickness of pericalcarine (left hemisphere) | No | No |
| **192** | Freesurfer desikan white | 26776 | Mean thickness of postcentral (left hemisphere) | No | No |
| **192** | Freesurfer desikan white | 26777 | Mean thickness of posteriorcingulate (left hemisphere) | No | No |
| **192** | Freesurfer desikan white | 26778 | Mean thickness of precentral (left hemisphere) | No | No |
| **192** | Freesurfer desikan white | 26779 | Mean thickness of precuneus (left hemisphere) | No | No |
| **192** | Freesurfer desikan white | 26780 | Mean thickness of rostralanteriorcingulate (left hemisphere) | No | No |
| **192** | Freesurfer desikan white | 26781 | Mean thickness of rostralmiddlefrontal (left hemisphere) | No | No |
| **192** | Freesurfer desikan white | 26782 | Mean thickness of superiorfrontal (left hemisphere) | No | No |
| **192** | Freesurfer desikan white | 26783 | Mean thickness of superiorparietal (left hemisphere) | No | No |
| **192** | Freesurfer desikan white | 26784 | Mean thickness of superiortemporal (left hemisphere) | No | No |
| **192** | Freesurfer desikan white | 26785 | Mean thickness of supramarginal (left hemisphere) | No | No |
| **192** | Freesurfer desikan white | 26786 | Mean thickness of frontalpole (left hemisphere) | No | No |
| **192** | Freesurfer desikan white | 26787 | Mean thickness of transversetemporal (left hemisphere) | No | No |
| **192** | Freesurfer desikan white | 26788 | Mean thickness of insula (left hemisphere) | No | No |
| **192** | Freesurfer desikan white | 26789 | Volume of bankssts (left hemisphere) | No | No |
| **192** | Freesurfer desikan white | 26790 | Volume of caudalanteriorcingulate (left hemisphere) | No | No |
| **192** | Freesurfer desikan white | 26791 | Volume of caudalmiddlefrontal (left hemisphere) | No | No |
| **192** | Freesurfer desikan white | 26792 | Volume of cuneus (left hemisphere) | No | No |
| **192** | Freesurfer desikan white | 26793 | Volume of entorhinal (left hemisphere) | No | No |
| **192** | Freesurfer desikan white | 26794 | Volume of fusiform (left hemisphere) | No | No |
| **192** | Freesurfer desikan white | 26795 | Volume of inferiorparietal (left hemisphere) | No | No |
| **192** | Freesurfer desikan white | 26796 | Volume of inferiortemporal (left hemisphere) | No | No |
| **192** | Freesurfer desikan white | 26797 | Volume of isthmuscingulate (left hemisphere) | No | No |
| **192** | Freesurfer desikan white | 26798 | Volume of lateraloccipital (left hemisphere) | No | No |
| **192** | Freesurfer desikan white | 26799 | Volume of lateralorbitofrontal (left hemisphere) | No | No |
| **192** | Freesurfer desikan white | 26800 | Volume of lingual (left hemisphere) | No | No |
| **192** | Freesurfer desikan white | 26801 | Volume of medialorbitofrontal (left hemisphere) | No | No |
| **192** | Freesurfer desikan white | 26802 | Volume of middletemporal (left hemisphere) | No | No |
| **192** | Freesurfer desikan white | 26803 | Volume of parahippocampal (left hemisphere) | No | No |
| **192** | Freesurfer desikan white | 26804 | Volume of paracentral (left hemisphere) | No | No |
| **192** | Freesurfer desikan white | 26805 | Volume of parsopercularis (left hemisphere) | No | No |
| **192** | Freesurfer desikan white | 26806 | Volume of parsorbitalis (left hemisphere) | No | No |
| **192** | Freesurfer desikan white | 26807 | Volume of parstriangularis (left hemisphere) | No | No |
| **192** | Freesurfer desikan white | 26808 | Volume of pericalcarine (left hemisphere) | No | No |
| **192** | Freesurfer desikan white | 26809 | Volume of postcentral (left hemisphere) | No | No |
| **192** | Freesurfer desikan white | 26810 | Volume of posteriorcingulate (left hemisphere) | No | No |
| **192** | Freesurfer desikan white | 26811 | Volume of precentral (left hemisphere) | No | No |
| **192** | Freesurfer desikan white | 26812 | Volume of precuneus (left hemisphere) | No | No |
| **192** | Freesurfer desikan white | 26813 | Volume of rostralanteriorcingulate (left hemisphere) | No | No |
| **192** | Freesurfer desikan white | 26814 | Volume of rostralmiddlefrontal (left hemisphere) | No | No |
| **192** | Freesurfer desikan white | 26815 | Volume of superiorfrontal (left hemisphere) | No | No |
| **192** | Freesurfer desikan white | 26816 | Volume of superiorparietal (left hemisphere) | No | No |
| **192** | Freesurfer desikan white | 26817 | Volume of superiortemporal (left hemisphere) | No | No |
| **192** | Freesurfer desikan white | 26818 | Volume of supramarginal (left hemisphere) | No | No |
| **192** | Freesurfer desikan white | 26819 | Volume of frontalpole (left hemisphere) | No | No |
| **192** | Freesurfer desikan white | 26820 | Volume of transversetemporal (left hemisphere) | No | No |
| **192** | Freesurfer desikan white | 26821 | Volume of insula (left hemisphere) | No | No |
| **192** | Freesurfer desikan white | 26822 | Area of TotalSurface (right hemisphere) | No | No |
| **192** | Freesurfer desikan white | 26823 | Area of bankssts (right hemisphere) | No | No |
| **192** | Freesurfer desikan white | 26824 | Area of caudalanteriorcingulate (right hemisphere) | No | No |
| **192** | Freesurfer desikan white | 26825 | Area of caudalmiddlefrontal (right hemisphere) | No | No |
| **192** | Freesurfer desikan white | 26826 | Area of cuneus (right hemisphere) | No | No |
| **192** | Freesurfer desikan white | 26827 | Area of entorhinal (right hemisphere) | No | No |
| **192** | Freesurfer desikan white | 26828 | Area of fusiform (right hemisphere) | No | No |
| **192** | Freesurfer desikan white | 26829 | Area of inferiorparietal (right hemisphere) | No | No |
| **192** | Freesurfer desikan white | 26830 | Area of inferiortemporal (right hemisphere) | No | No |
| **192** | Freesurfer desikan white | 26831 | Area of isthmuscingulate (right hemisphere) | No | No |
| **192** | Freesurfer desikan white | 26832 | Area of lateraloccipital (right hemisphere) | No | No |
| **192** | Freesurfer desikan white | 26833 | Area of lateralorbitofrontal (right hemisphere) | No | No |
| **192** | Freesurfer desikan white | 26834 | Area of lingual (right hemisphere) | No | No |
| **192** | Freesurfer desikan white | 26835 | Area of medialorbitofrontal (right hemisphere) | No | No |
| **192** | Freesurfer desikan white | 26836 | Area of middletemporal (right hemisphere) | No | No |
| **192** | Freesurfer desikan white | 26837 | Area of parahippocampal (right hemisphere) | No | No |
| **192** | Freesurfer desikan white | 26838 | Area of paracentral (right hemisphere) | No | No |
| **192** | Freesurfer desikan white | 26839 | Area of parsopercularis (right hemisphere) | No | No |
| **192** | Freesurfer desikan white | 26840 | Area of parsorbitalis (right hemisphere) | No | No |
| **192** | Freesurfer desikan white | 26841 | Area of parstriangularis (right hemisphere) | No | No |
| **192** | Freesurfer desikan white | 26842 | Area of pericalcarine (right hemisphere) | No | No |
| **192** | Freesurfer desikan white | 26843 | Area of postcentral (right hemisphere) | No | No |
| **192** | Freesurfer desikan white | 26844 | Area of posteriorcingulate (right hemisphere) | No | No |
| **192** | Freesurfer desikan white | 26845 | Area of precentral (right hemisphere) | No | No |
| **192** | Freesurfer desikan white | 26846 | Area of precuneus (right hemisphere) | No | No |
| **192** | Freesurfer desikan white | 26847 | Area of rostralanteriorcingulate (right hemisphere) | No | No |
| **192** | Freesurfer desikan white | 26848 | Area of rostralmiddlefrontal (right hemisphere) | No | No |
| **192** | Freesurfer desikan white | 26849 | Area of superiorfrontal (right hemisphere) | No | No |
| **192** | Freesurfer desikan white | 26850 | Area of superiorparietal (right hemisphere) | No | No |
| **192** | Freesurfer desikan white | 26851 | Area of superiortemporal (right hemisphere) | No | No |
| **192** | Freesurfer desikan white | 26852 | Area of supramarginal (right hemisphere) | No | No |
| **192** | Freesurfer desikan white | 26853 | Area of frontalpole (right hemisphere) | No | No |
| **192** | Freesurfer desikan white | 26854 | Area of transversetemporal (right hemisphere) | No | No |
| **192** | Freesurfer desikan white | 26855 | Area of insula (right hemisphere) | No | No |
| **192** | Freesurfer desikan white | 26856 | Mean thickness of GlobalMeanMean thickness (right hemisphere) | No | No |
| **192** | Freesurfer desikan white | 26857 | Mean thickness of bankssts (right hemisphere) | No | No |
| **192** | Freesurfer desikan white | 26858 | Mean thickness of caudalanteriorcingulate (right hemisphere) | No | No |
| **192** | Freesurfer desikan white | 26859 | Mean thickness of caudalmiddlefrontal (right hemisphere) | No | No |
| **192** | Freesurfer desikan white | 26860 | Mean thickness of cuneus (right hemisphere) | No | No |
| **192** | Freesurfer desikan white | 26861 | Mean thickness of entorhinal (right hemisphere) | No | No |
| **192** | Freesurfer desikan white | 26862 | Mean thickness of fusiform (right hemisphere) | No | No |
| **192** | Freesurfer desikan white | 26863 | Mean thickness of inferiorparietal (right hemisphere) | No | No |
| **192** | Freesurfer desikan white | 26864 | Mean thickness of inferiortemporal (right hemisphere) | No | No |
| **192** | Freesurfer desikan white | 26865 | Mean thickness of isthmuscingulate (right hemisphere) | No | No |
| **192** | Freesurfer desikan white | 26866 | Mean thickness of lateraloccipital (right hemisphere) | No | No |
| **192** | Freesurfer desikan white | 26867 | Mean thickness of lateralorbitofrontal (right hemisphere) | No | No |
| **192** | Freesurfer desikan white | 26868 | Mean thickness of lingual (right hemisphere) | No | No |
| **192** | Freesurfer desikan white | 26869 | Mean thickness of medialorbitofrontal (right hemisphere) | No | No |
| **192** | Freesurfer desikan white | 26870 | Mean thickness of middletemporal (right hemisphere) | No | No |
| **192** | Freesurfer desikan white | 26871 | Mean thickness of parahippocampal (right hemisphere) | No | No |
| **192** | Freesurfer desikan white | 26872 | Mean thickness of paracentral (right hemisphere) | No | No |
| **192** | Freesurfer desikan white | 26873 | Mean thickness of parsopercularis (right hemisphere) | No | No |
| **192** | Freesurfer desikan white | 26874 | Mean thickness of parsorbitalis (right hemisphere) | No | No |
| **192** | Freesurfer desikan white | 26875 | Mean thickness of parstriangularis (right hemisphere) | No | No |
| **192** | Freesurfer desikan white | 26876 | Mean thickness of pericalcarine (right hemisphere) | No | No |
| **192** | Freesurfer desikan white | 26877 | Mean thickness of postcentral (right hemisphere) | No | No |
| **192** | Freesurfer desikan white | 26878 | Mean thickness of posteriorcingulate (right hemisphere) | No | No |
| **192** | Freesurfer desikan white | 26879 | Mean thickness of precentral (right hemisphere) | No | No |
| **192** | Freesurfer desikan white | 26880 | Mean thickness of precuneus (right hemisphere) | No | No |
| **192** | Freesurfer desikan white | 26881 | Mean thickness of rostralanteriorcingulate (right hemisphere) | No | No |
| **192** | Freesurfer desikan white | 26882 | Mean thickness of rostralmiddlefrontal (right hemisphere) | No | No |
| **192** | Freesurfer desikan white | 26883 | Mean thickness of superiorfrontal (right hemisphere) | No | No |
| **192** | Freesurfer desikan white | 26884 | Mean thickness of superiorparietal (right hemisphere) | No | No |
| **192** | Freesurfer desikan white | 26885 | Mean thickness of superiortemporal (right hemisphere) | No | No |
| **192** | Freesurfer desikan white | 26886 | Mean thickness of supramarginal (right hemisphere) | No | No |
| **192** | Freesurfer desikan white | 26887 | Mean thickness of frontalpole (right hemisphere) | No | No |
| **192** | Freesurfer desikan white | 26888 | Mean thickness of transversetemporal (right hemisphere) | No | No |
| **192** | Freesurfer desikan white | 26889 | Mean thickness of insula (right hemisphere) | No | No |
| **192** | Freesurfer desikan white | 26890 | Volume of bankssts (right hemisphere) | No | No |
| **192** | Freesurfer desikan white | 26891 | Volume of caudalanteriorcingulate (right hemisphere) | No | No |
| **192** | Freesurfer desikan white | 26892 | Volume of caudalmiddlefrontal (right hemisphere) | No | No |
| **192** | Freesurfer desikan white | 26893 | Volume of cuneus (right hemisphere) | No | No |
| **192** | Freesurfer desikan white | 26894 | Volume of entorhinal (right hemisphere) | No | No |
| **192** | Freesurfer desikan white | 26895 | Volume of fusiform (right hemisphere) | No | No |
| **192** | Freesurfer desikan white | 26896 | Volume of inferiorparietal (right hemisphere) | No | No |
| **192** | Freesurfer desikan white | 26897 | Volume of inferiortemporal (right hemisphere) | No | No |
| **192** | Freesurfer desikan white | 26898 | Volume of isthmuscingulate (right hemisphere) | No | No |
| **192** | Freesurfer desikan white | 26899 | Volume of lateraloccipital (right hemisphere) | No | No |
| **192** | Freesurfer desikan white | 26900 | Volume of lateralorbitofrontal (right hemisphere) | No | No |
| **192** | Freesurfer desikan white | 26901 | Volume of lingual (right hemisphere) | No | No |
| **192** | Freesurfer desikan white | 26902 | Volume of medialorbitofrontal (right hemisphere) | No | No |
| **192** | Freesurfer desikan white | 26903 | Volume of middletemporal (right hemisphere) | No | No |
| **192** | Freesurfer desikan white | 26904 | Volume of parahippocampal (right hemisphere) | No | No |
| **192** | Freesurfer desikan white | 26905 | Volume of paracentral (right hemisphere) | No | No |
| **192** | Freesurfer desikan white | 26906 | Volume of parsopercularis (right hemisphere) | No | No |
| **192** | Freesurfer desikan white | 26907 | Volume of parsorbitalis (right hemisphere) | No | No |
| **192** | Freesurfer desikan white | 26908 | Volume of parstriangularis (right hemisphere) | No | No |
| **192** | Freesurfer desikan white | 26909 | Volume of pericalcarine (right hemisphere) | No | No |
| **192** | Freesurfer desikan white | 26910 | Volume of postcentral (right hemisphere) | No | No |
| **192** | Freesurfer desikan white | 26911 | Volume of posteriorcingulate (right hemisphere) | No | No |
| **192** | Freesurfer desikan white | 26912 | Volume of precentral (right hemisphere) | No | No |
| **192** | Freesurfer desikan white | 26913 | Volume of precuneus (right hemisphere) | No | No |
| **192** | Freesurfer desikan white | 26914 | Volume of rostralanteriorcingulate (right hemisphere) | No | No |
| **192** | Freesurfer desikan white | 26915 | Volume of rostralmiddlefrontal (right hemisphere) | No | No |
| **192** | Freesurfer desikan white | 26916 | Volume of superiorfrontal (right hemisphere) | No | No |
| **192** | Freesurfer desikan white | 26917 | Volume of superiorparietal (right hemisphere) | No | No |
| **192** | Freesurfer desikan white | 26918 | Volume of superiortemporal (right hemisphere) | No | No |
| **192** | Freesurfer desikan white | 26919 | Volume of supramarginal (right hemisphere) | No | No |
| **192** | Freesurfer desikan white | 26920 | Volume of frontalpole (right hemisphere) | No | No |
| **192** | Freesurfer desikan white | 26921 | Volume of transversetemporal (right hemisphere) | No | No |
| **192** | Freesurfer desikan white | 26922 | Volume of insula (right hemisphere) | No | No |
| **193** | Freesurfer desikan pial | 26923 | Area of TotalSurface (left hemisphere) | No | No |
| **193** | Freesurfer desikan pial | 26924 | Area of bankssts (left hemisphere) | No | No |
| **193** | Freesurfer desikan pial | 26925 | Area of caudalanteriorcingulate (left hemisphere) | No | No |
| **193** | Freesurfer desikan pial | 26926 | Area of caudalmiddlefrontal (left hemisphere) | No | No |
| **193** | Freesurfer desikan pial | 26927 | Area of cuneus (left hemisphere) | No | No |
| **193** | Freesurfer desikan pial | 26928 | Area of entorhinal (left hemisphere) | No | No |
| **193** | Freesurfer desikan pial | 26929 | Area of fusiform (left hemisphere) | No | No |
| **193** | Freesurfer desikan pial | 26930 | Area of inferiorparietal (left hemisphere) | No | No |
| **193** | Freesurfer desikan pial | 26931 | Area of inferiortemporal (left hemisphere) | No | No |
| **193** | Freesurfer desikan pial | 26932 | Area of isthmuscingulate (left hemisphere) | No | No |
| **193** | Freesurfer desikan pial | 26933 | Area of lateraloccipital (left hemisphere) | No | No |
| **193** | Freesurfer desikan pial | 26934 | Area of lateralorbitofrontal (left hemisphere) | No | No |
| **193** | Freesurfer desikan pial | 26935 | Area of lingual (left hemisphere) | No | No |
| **193** | Freesurfer desikan pial | 26936 | Area of medialorbitofrontal (left hemisphere) | No | No |
| **193** | Freesurfer desikan pial | 26937 | Area of middletemporal (left hemisphere) | No | No |
| **193** | Freesurfer desikan pial | 26938 | Area of parahippocampal (left hemisphere) | No | No |
| **193** | Freesurfer desikan pial | 26939 | Area of paracentral (left hemisphere) | No | No |
| **193** | Freesurfer desikan pial | 26940 | Area of parsopercularis (left hemisphere) | No | No |
| **193** | Freesurfer desikan pial | 26941 | Area of parsorbitalis (left hemisphere) | No | No |
| **193** | Freesurfer desikan pial | 26942 | Area of parstriangularis (left hemisphere) | No | No |
| **193** | Freesurfer desikan pial | 26943 | Area of pericalcarine (left hemisphere) | No | No |
| **193** | Freesurfer desikan pial | 26944 | Area of postcentral (left hemisphere) | No | No |
| **193** | Freesurfer desikan pial | 26945 | Area of posteriorcingulate (left hemisphere) | No | No |
| **193** | Freesurfer desikan pial | 26946 | Area of precentral (left hemisphere) | No | No |
| **193** | Freesurfer desikan pial | 26947 | Area of precuneus (left hemisphere) | No | No |
| **193** | Freesurfer desikan pial | 26948 | Area of rostralanteriorcingulate (left hemisphere) | No | No |
| **193** | Freesurfer desikan pial | 26949 | Area of rostralmiddlefrontal (left hemisphere) | No | No |
| **193** | Freesurfer desikan pial | 26950 | Area of superiorfrontal (left hemisphere) | No | No |
| **193** | Freesurfer desikan pial | 26951 | Area of superiorparietal (left hemisphere) | No | No |
| **193** | Freesurfer desikan pial | 26952 | Area of superiortemporal (left hemisphere) | No | No |
| **193** | Freesurfer desikan pial | 26953 | Area of supramarginal (left hemisphere) | No | No |
| **193** | Freesurfer desikan pial | 26954 | Area of frontalpole (left hemisphere) | No | No |
| **193** | Freesurfer desikan pial | 26955 | Area of transversetemporal (left hemisphere) | No | No |
| **193** | Freesurfer desikan pial | 26956 | Area of TotalSurface (right hemisphere) | No | No |
| **193** | Freesurfer desikan pial | 26957 | Area of bankssts (right hemisphere) | No | No |
| **193** | Freesurfer desikan pial | 26958 | Area of caudalanteriorcingulate (right hemisphere) | No | No |
| **193** | Freesurfer desikan pial | 26959 | Area of caudalmiddlefrontal (right hemisphere) | No | No |
| **193** | Freesurfer desikan pial | 26960 | Area of cuneus (right hemisphere) | No | No |
| **193** | Freesurfer desikan pial | 26961 | Area of entorhinal (right hemisphere) | No | No |
| **193** | Freesurfer desikan pial | 26962 | Area of fusiform (right hemisphere) | No | No |
| **193** | Freesurfer desikan pial | 26963 | Area of inferiorparietal (right hemisphere) | No | No |
| **193** | Freesurfer desikan pial | 26964 | Area of inferiortemporal (right hemisphere) | No | No |
| **193** | Freesurfer desikan pial | 26965 | Area of isthmuscingulate (right hemisphere) | No | No |
| **193** | Freesurfer desikan pial | 26966 | Area of lateraloccipital (right hemisphere) | No | No |
| **193** | Freesurfer desikan pial | 26967 | Area of lateralorbitofrontal (right hemisphere) | No | No |
| **193** | Freesurfer desikan pial | 26968 | Area of lingual (right hemisphere) | No | No |
| **193** | Freesurfer desikan pial | 26969 | Area of medialorbitofrontal (right hemisphere) | No | No |
| **193** | Freesurfer desikan pial | 26970 | Area of middletemporal (right hemisphere) | No | No |
| **193** | Freesurfer desikan pial | 26971 | Area of parahippocampal (right hemisphere) | No | No |
| **193** | Freesurfer desikan pial | 26972 | Area of paracentral (right hemisphere) | No | No |
| **193** | Freesurfer desikan pial | 26973 | Area of parsopercularis (right hemisphere) | No | No |
| **193** | Freesurfer desikan pial | 26974 | Area of parsorbitalis (right hemisphere) | No | No |
| **193** | Freesurfer desikan pial | 26975 | Area of parstriangularis (right hemisphere) | No | No |
| **193** | Freesurfer desikan pial | 26976 | Area of pericalcarine (right hemisphere) | No | No |
| **193** | Freesurfer desikan pial | 26977 | Area of postcentral (right hemisphere) | No | No |
| **193** | Freesurfer desikan pial | 26978 | Area of posteriorcingulate (right hemisphere) | No | No |
| **193** | Freesurfer desikan pial | 26979 | Area of precentral (right hemisphere) | No | No |
| **193** | Freesurfer desikan pial | 26980 | Area of precuneus (right hemisphere) | No | No |
| **193** | Freesurfer desikan pial | 26981 | Area of rostralanteriorcingulate (right hemisphere) | No | No |
| **193** | Freesurfer desikan pial | 26982 | Area of rostralmiddlefrontal (right hemisphere) | No | No |
| **193** | Freesurfer desikan pial | 26983 | Area of superiorfrontal (right hemisphere) | No | No |
| **193** | Freesurfer desikan pial | 26984 | Area of superiorparietal (right hemisphere) | No | No |
| **193** | Freesurfer desikan pial | 26985 | Area of superiortemporal (right hemisphere) | No | No |
| **193** | Freesurfer desikan pial | 26986 | Area of supramarginal (right hemisphere) | No | No |
| **193** | Freesurfer desikan pial | 26987 | Area of frontalpole (right hemisphere) | No | No |
| **193** | Freesurfer desikan pial | 26988 | Area of transversetemporal (right hemisphere) | No | No |
| **194** | Freesurfer desikan gw | 26989 | Grey-white contrast in unknown (left hemisphere) | No | No |
| **194** | Freesurfer desikan gw | 26990 | Grey-white contrast in bankssts (left hemisphere) | No | No |
| **194** | Freesurfer desikan gw | 26991 | Grey-white contrast in caudalanteriorcingulate (left hemisphere) | No | No |
| **194** | Freesurfer desikan gw | 26992 | Grey-white contrast in caudalmiddlefrontal (left hemisphere) | No | No |
| **194** | Freesurfer desikan gw | 26993 | Grey-white contrast in cuneus (left hemisphere) | No | No |
| **194** | Freesurfer desikan gw | 26994 | Grey-white contrast in entorhinal (left hemisphere) | No | No |
| **194** | Freesurfer desikan gw | 26995 | Grey-white contrast in fusiform (left hemisphere) | No | No |
| **194** | Freesurfer desikan gw | 26996 | Grey-white contrast in inferiorparietal (left hemisphere) | No | No |
| **194** | Freesurfer desikan gw | 26997 | Grey-white contrast in inferiortemporal (left hemisphere) | No | No |
| **194** | Freesurfer desikan gw | 26998 | Grey-white contrast in isthmuscingulate (left hemisphere) | No | No |
| **194** | Freesurfer desikan gw | 26999 | Grey-white contrast in lateraloccipital (left hemisphere) | No | No |
| **194** | Freesurfer desikan gw | 27000 | Grey-white contrast in lateralorbitofrontal (left hemisphere) | No | No |
| **194** | Freesurfer desikan gw | 27001 | Grey-white contrast in lingual (left hemisphere) | No | No |
| **194** | Freesurfer desikan gw | 27002 | Grey-white contrast in medialorbitofrontal (left hemisphere) | No | No |
| **194** | Freesurfer desikan gw | 27003 | Grey-white contrast in middletemporal (left hemisphere) | No | No |
| **194** | Freesurfer desikan gw | 27004 | Grey-white contrast in parahippocampal (left hemisphere) | No | No |
| **194** | Freesurfer desikan gw | 27005 | Grey-white contrast in paracentral (left hemisphere) | No | No |
| **194** | Freesurfer desikan gw | 27006 | Grey-white contrast in parsopercularis (left hemisphere) | No | No |
| **194** | Freesurfer desikan gw | 27007 | Grey-white contrast in parsorbitalis (left hemisphere) | No | No |
| **194** | Freesurfer desikan gw | 27008 | Grey-white contrast in parstriangularis (left hemisphere) | No | No |
| **194** | Freesurfer desikan gw | 27009 | Grey-white contrast in pericalcarine (left hemisphere) | No | No |
| **194** | Freesurfer desikan gw | 27010 | Grey-white contrast in postcentral (left hemisphere) | No | No |
| **194** | Freesurfer desikan gw | 27011 | Grey-white contrast in posteriorcingulate (left hemisphere) | No | No |
| **194** | Freesurfer desikan gw | 27012 | Grey-white contrast in precentral (left hemisphere) | No | No |
| **194** | Freesurfer desikan gw | 27013 | Grey-white contrast in precuneus (left hemisphere) | No | No |
| **194** | Freesurfer desikan gw | 27014 | Grey-white contrast in rostralanteriorcingulate (left hemisphere) | No | No |
| **194** | Freesurfer desikan gw | 27015 | Grey-white contrast in rostralmiddlefrontal (left hemisphere) | No | No |
| **194** | Freesurfer desikan gw | 27016 | Grey-white contrast in superiorfrontal (left hemisphere) | No | No |
| **194** | Freesurfer desikan gw | 27017 | Grey-white contrast in superiorparietal (left hemisphere) | No | No |
| **194** | Freesurfer desikan gw | 27018 | Grey-white contrast in superiortemporal (left hemisphere) | No | No |
| **194** | Freesurfer desikan gw | 27019 | Grey-white contrast in supramarginal (left hemisphere) | No | No |
| **194** | Freesurfer desikan gw | 27020 | Grey-white contrast in frontalpole (left hemisphere) | No | No |
| **194** | Freesurfer desikan gw | 27021 | Grey-white contrast in temporalpole (left hemisphere) | No | No |
| **194** | Freesurfer desikan gw | 27022 | Grey-white contrast in transversetemporal (left hemisphere) | No | No |
| **194** | Freesurfer desikan gw | 27023 | Grey-white contrast in insula (left hemisphere) | No | No |
| **194** | Freesurfer desikan gw | 27024 | Grey-white contrast in unknown (right hemisphere) | No | No |
| **194** | Freesurfer desikan gw | 27025 | Grey-white contrast in bankssts (right hemisphere) | No | No |
| **194** | Freesurfer desikan gw | 27026 | Grey-white contrast in caudalanteriorcingulate (right hemisphere) | No | No |
| **194** | Freesurfer desikan gw | 27027 | Grey-white contrast in caudalmiddlefrontal (right hemisphere) | No | No |
| **194** | Freesurfer desikan gw | 27028 | Grey-white contrast in cuneus (right hemisphere) | No | No |
| **194** | Freesurfer desikan gw | 27029 | Grey-white contrast in entorhinal (right hemisphere) | No | No |
| **194** | Freesurfer desikan gw | 27030 | Grey-white contrast in fusiform (right hemisphere) | No | No |
| **194** | Freesurfer desikan gw | 27031 | Grey-white contrast in inferiorparietal (right hemisphere) | No | No |
| **194** | Freesurfer desikan gw | 27032 | Grey-white contrast in inferiortemporal (right hemisphere) | No | No |
| **194** | Freesurfer desikan gw | 27033 | Grey-white contrast in isthmuscingulate (right hemisphere) | No | No |
| **194** | Freesurfer desikan gw | 27034 | Grey-white contrast in lateraloccipital (right hemisphere) | No | No |
| **194** | Freesurfer desikan gw | 27035 | Grey-white contrast in lateralorbitofrontal (right hemisphere) | No | No |
| **194** | Freesurfer desikan gw | 27036 | Grey-white contrast in lingual (right hemisphere) | No | No |
| **194** | Freesurfer desikan gw | 27037 | Grey-white contrast in medialorbitofrontal (right hemisphere) | No | No |
| **194** | Freesurfer desikan gw | 27038 | Grey-white contrast in middletemporal (right hemisphere) | No | No |
| **194** | Freesurfer desikan gw | 27039 | Grey-white contrast in parahippocampal (right hemisphere) | No | No |
| **194** | Freesurfer desikan gw | 27040 | Grey-white contrast in paracentral (right hemisphere) | No | No |
| **194** | Freesurfer desikan gw | 27041 | Grey-white contrast in parsopercularis (right hemisphere) | No | No |
| **194** | Freesurfer desikan gw | 27042 | Grey-white contrast in parsorbitalis (right hemisphere) | No | No |
| **194** | Freesurfer desikan gw | 27043 | Grey-white contrast in parstriangularis (right hemisphere) | No | No |
| **194** | Freesurfer desikan gw | 27044 | Grey-white contrast in pericalcarine (right hemisphere) | No | No |
| **194** | Freesurfer desikan gw | 27045 | Grey-white contrast in postcentral (right hemisphere) | No | No |
| **194** | Freesurfer desikan gw | 27046 | Grey-white contrast in posteriorcingulate (right hemisphere) | No | No |
| **194** | Freesurfer desikan gw | 27047 | Grey-white contrast in precentral (right hemisphere) | No | No |
| **194** | Freesurfer desikan gw | 27048 | Grey-white contrast in precuneus (right hemisphere) | No | No |
| **194** | Freesurfer desikan gw | 27049 | Grey-white contrast in rostralanteriorcingulate (right hemisphere) | No | No |
| **194** | Freesurfer desikan gw | 27050 | Grey-white contrast in rostralmiddlefrontal (right hemisphere) | No | No |
| **194** | Freesurfer desikan gw | 27051 | Grey-white contrast in superiorfrontal (right hemisphere) | No | No |
| **194** | Freesurfer desikan gw | 27052 | Grey-white contrast in superiorparietal (right hemisphere) | No | No |
| **194** | Freesurfer desikan gw | 27053 | Grey-white contrast in superiortemporal (right hemisphere) | No | No |
| **194** | Freesurfer desikan gw | 27054 | Grey-white contrast in supramarginal (right hemisphere) | No | No |
| **194** | Freesurfer desikan gw | 27055 | Grey-white contrast in frontalpole (right hemisphere) | No | No |
| **194** | Freesurfer desikan gw | 27056 | Grey-white contrast in temporalpole (right hemisphere) | No | No |
| **194** | Freesurfer desikan gw | 27057 | Grey-white contrast in transversetemporal (right hemisphere) | No | No |
| **194** | Freesurfer desikan gw | 27058 | Grey-white contrast in insula (right hemisphere) | No | No |
| **195** | Freesurfer BA exvivo | 27059 | Area of BA1 (left hemisphere) | No | No |
| **195** | Freesurfer BA exvivo | 27060 | Area of BA2 (left hemisphere) | No | No |
| **195** | Freesurfer BA exvivo | 27061 | Area of BA3a (left hemisphere) | No | No |
| **195** | Freesurfer BA exvivo | 27062 | Area of BA3b (left hemisphere) | No | No |
| **195** | Freesurfer BA exvivo | 27063 | Area of BA4a (left hemisphere) | No | No |
| **195** | Freesurfer BA exvivo | 27064 | Area of BA4p (left hemisphere) | No | No |
| **195** | Freesurfer BA exvivo | 27065 | Area of BA6 (left hemisphere) | No | No |
| **195** | Freesurfer BA exvivo | 27066 | Area of BA44 (left hemisphere) | No | No |
| **195** | Freesurfer BA exvivo | 27067 | Area of BA45 (left hemisphere) | No | No |
| **195** | Freesurfer BA exvivo | 27068 | Area of V1 (left hemisphere) | No | No |
| **195** | Freesurfer BA exvivo | 27069 | Area of V2 (left hemisphere) | No | No |
| **195** | Freesurfer BA exvivo | 27070 | Area of MT (left hemisphere) | No | No |
| **195** | Freesurfer BA exvivo | 27071 | Area of perirhinal (left hemisphere) | No | No |
| **195** | Freesurfer BA exvivo | 27072 | Area of entorhinal (left hemisphere) | No | No |
| **195** | Freesurfer BA exvivo | 27073 | Mean thickness of BA1 (left hemisphere) | No | No |
| **195** | Freesurfer BA exvivo | 27074 | Mean thickness of BA2 (left hemisphere) | No | No |
| **195** | Freesurfer BA exvivo | 27075 | Mean thickness of BA3a (left hemisphere) | No | No |
| **195** | Freesurfer BA exvivo | 27076 | Mean thickness of BA3b (left hemisphere) | No | No |
| **195** | Freesurfer BA exvivo | 27077 | Mean thickness of BA4a (left hemisphere) | No | No |
| **195** | Freesurfer BA exvivo | 27078 | Mean thickness of BA4p (left hemisphere) | No | No |
| **195** | Freesurfer BA exvivo | 27079 | Mean thickness of BA6 (left hemisphere) | No | No |
| **195** | Freesurfer BA exvivo | 27080 | Mean thickness of BA44 (left hemisphere) | No | No |
| **195** | Freesurfer BA exvivo | 27081 | Mean thickness of BA45 (left hemisphere) | No | No |
| **195** | Freesurfer BA exvivo | 27082 | Mean thickness of V1 (left hemisphere) | No | No |
| **195** | Freesurfer BA exvivo | 27083 | Mean thickness of V2 (left hemisphere) | No | No |
| **195** | Freesurfer BA exvivo | 27084 | Mean thickness of MT (left hemisphere) | No | No |
| **195** | Freesurfer BA exvivo | 27085 | Mean thickness of perirhinal (left hemisphere) | No | No |
| **195** | Freesurfer BA exvivo | 27086 | Mean thickness of entorhinal (left hemisphere) | No | No |
| **195** | Freesurfer BA exvivo | 27087 | Volume of BA1 (left hemisphere) | No | No |
| **195** | Freesurfer BA exvivo | 27088 | Volume of BA2 (left hemisphere) | No | No |
| **195** | Freesurfer BA exvivo | 27089 | Volume of BA3a (left hemisphere) | No | No |
| **195** | Freesurfer BA exvivo | 27090 | Volume of BA3b (left hemisphere) | No | No |
| **195** | Freesurfer BA exvivo | 27091 | Volume of BA4a (left hemisphere) | No | No |
| **195** | Freesurfer BA exvivo | 27092 | Volume of BA4p (left hemisphere) | No | No |
| **195** | Freesurfer BA exvivo | 27093 | Volume of BA6 (left hemisphere) | No | No |
| **195** | Freesurfer BA exvivo | 27094 | Volume of BA44 (left hemisphere) | No | No |
| **195** | Freesurfer BA exvivo | 27095 | Volume of BA45 (left hemisphere) | No | No |
| **195** | Freesurfer BA exvivo | 27096 | Volume of V1 (left hemisphere) | No | No |
| **195** | Freesurfer BA exvivo | 27097 | Volume of V2 (left hemisphere) | No | No |
| **195** | Freesurfer BA exvivo | 27098 | Volume of MT (left hemisphere) | No | No |
| **195** | Freesurfer BA exvivo | 27099 | Volume of perirhinal (left hemisphere) | No | No |
| **195** | Freesurfer BA exvivo | 27100 | Volume of entorhinal (left hemisphere) | No | No |
| **195** | Freesurfer BA exvivo | 27101 | Area of BA1 (right hemisphere) | No | No |
| **195** | Freesurfer BA exvivo | 27102 | Area of BA2 (right hemisphere) | No | No |
| **195** | Freesurfer BA exvivo | 27103 | Area of BA3a (right hemisphere) | No | No |
| **195** | Freesurfer BA exvivo | 27104 | Area of BA3b (right hemisphere) | No | No |
| **195** | Freesurfer BA exvivo | 27105 | Area of BA4a (right hemisphere) | No | No |
| **195** | Freesurfer BA exvivo | 27106 | Area of BA4p (right hemisphere) | No | No |
| **195** | Freesurfer BA exvivo | 27107 | Area of BA6 (right hemisphere) | No | No |
| **195** | Freesurfer BA exvivo | 27108 | Area of BA44 (right hemisphere) | No | No |
| **195** | Freesurfer BA exvivo | 27109 | Area of BA45 (right hemisphere) | No | No |
| **195** | Freesurfer BA exvivo | 27110 | Area of V1 (right hemisphere) | No | No |
| **195** | Freesurfer BA exvivo | 27111 | Area of V2 (right hemisphere) | No | No |
| **195** | Freesurfer BA exvivo | 27112 | Area of MT (right hemisphere) | No | No |
| **195** | Freesurfer BA exvivo | 27113 | Area of perirhinal (right hemisphere) | No | No |
| **195** | Freesurfer BA exvivo | 27114 | Area of entorhinal (right hemisphere) | No | No |
| **195** | Freesurfer BA exvivo | 27115 | Mean thickness of BA1 (right hemisphere) | No | No |
| **195** | Freesurfer BA exvivo | 27116 | Mean thickness of BA2 (right hemisphere) | No | No |
| **195** | Freesurfer BA exvivo | 27117 | Mean thickness of BA3a (right hemisphere) | No | No |
| **195** | Freesurfer BA exvivo | 27118 | Mean thickness of BA3b (right hemisphere) | No | No |
| **195** | Freesurfer BA exvivo | 27119 | Mean thickness of BA4a (right hemisphere) | No | No |
| **195** | Freesurfer BA exvivo | 27120 | Mean thickness of BA4p (right hemisphere) | No | No |
| **195** | Freesurfer BA exvivo | 27121 | Mean thickness of BA6 (right hemisphere) | No | No |
| **195** | Freesurfer BA exvivo | 27122 | Mean thickness of BA44 (right hemisphere) | No | No |
| **195** | Freesurfer BA exvivo | 27123 | Mean thickness of BA45 (right hemisphere) | No | No |
| **195** | Freesurfer BA exvivo | 27124 | Mean thickness of V1 (right hemisphere) | No | No |
| **195** | Freesurfer BA exvivo | 27125 | Mean thickness of V2 (right hemisphere) | No | No |
| **195** | Freesurfer BA exvivo | 27126 | Mean thickness of MT (right hemisphere) | No | No |
| **195** | Freesurfer BA exvivo | 27127 | Mean thickness of perirhinal (right hemisphere) | No | No |
| **195** | Freesurfer BA exvivo | 27128 | Mean thickness of entorhinal (right hemisphere) | No | No |
| **195** | Freesurfer BA exvivo | 27129 | Volume of BA1 (right hemisphere) | No | No |
| **195** | Freesurfer BA exvivo | 27130 | Volume of BA2 (right hemisphere) | No | No |
| **195** | Freesurfer BA exvivo | 27131 | Volume of BA3a (right hemisphere) | No | No |
| **195** | Freesurfer BA exvivo | 27132 | Volume of BA3b (right hemisphere) | No | No |
| **195** | Freesurfer BA exvivo | 27133 | Volume of BA4a (right hemisphere) | No | No |
| **195** | Freesurfer BA exvivo | 27134 | Volume of BA4p (right hemisphere) | No | No |
| **195** | Freesurfer BA exvivo | 27135 | Volume of BA6 (right hemisphere) | No | No |
| **195** | Freesurfer BA exvivo | 27136 | Volume of BA44 (right hemisphere) | No | No |
| **195** | Freesurfer BA exvivo | 27137 | Volume of BA45 (right hemisphere) | No | No |
| **195** | Freesurfer BA exvivo | 27138 | Volume of V1 (right hemisphere) | No | No |
| **195** | Freesurfer BA exvivo | 27139 | Volume of V2 (right hemisphere) | No | No |
| **195** | Freesurfer BA exvivo | 27140 | Volume of MT (right hemisphere) | No | No |
| **195** | Freesurfer BA exvivo | 27141 | Volume of perirhinal (right hemisphere) | No | No |
| **195** | Freesurfer BA exvivo | 27142 | Volume of entorhinal (right hemisphere) | No | No |
| **196** | Freesurfer DKT | 27143 | Area of caudalanteriorcingulate (left hemisphere) | No | No |
| **196** | Freesurfer DKT | 27144 | Area of caudalmiddlefrontal (left hemisphere) | No | No |
| **196** | Freesurfer DKT | 27145 | Area of cuneus (left hemisphere) | No | No |
| **196** | Freesurfer DKT | 27146 | Area of entorhinal (left hemisphere) | No | No |
| **196** | Freesurfer DKT | 27147 | Area of fusiform (left hemisphere) | No | No |
| **196** | Freesurfer DKT | 27148 | Area of inferiorparietal (left hemisphere) | No | No |
| **196** | Freesurfer DKT | 27149 | Area of inferiortemporal (left hemisphere) | No | No |
| **196** | Freesurfer DKT | 27150 | Area of isthmuscingulate (left hemisphere) | No | No |
| **196** | Freesurfer DKT | 27151 | Area of lateraloccipital (left hemisphere) | No | No |
| **196** | Freesurfer DKT | 27152 | Area of lateralorbitofrontal (left hemisphere) | No | No |
| **196** | Freesurfer DKT | 27153 | Area of lingual (left hemisphere) | No | No |
| **196** | Freesurfer DKT | 27154 | Area of medialorbitofrontal (left hemisphere) | No | No |
| **196** | Freesurfer DKT | 27155 | Area of middletemporal (left hemisphere) | No | No |
| **196** | Freesurfer DKT | 27156 | Area of parahippocampal (left hemisphere) | No | No |
| **196** | Freesurfer DKT | 27157 | Area of paracentral (left hemisphere) | No | No |
| **196** | Freesurfer DKT | 27158 | Area of parsopercularis (left hemisphere) | No | No |
| **196** | Freesurfer DKT | 27159 | Area of parsorbitalis (left hemisphere) | No | No |
| **196** | Freesurfer DKT | 27160 | Area of parstriangularis (left hemisphere) | No | No |
| **196** | Freesurfer DKT | 27161 | Area of pericalcarine (left hemisphere) | No | No |
| **196** | Freesurfer DKT | 27162 | Area of postcentral (left hemisphere) | No | No |
| **196** | Freesurfer DKT | 27163 | Area of posteriorcingulate (left hemisphere) | No | No |
| **196** | Freesurfer DKT | 27164 | Area of precentral (left hemisphere) | No | No |
| **196** | Freesurfer DKT | 27165 | Area of precuneus (left hemisphere) | No | No |
| **196** | Freesurfer DKT | 27166 | Area of rostralanteriorcingulate (left hemisphere) | No | No |
| **196** | Freesurfer DKT | 27167 | Area of rostralmiddlefrontal (left hemisphere) | No | No |
| **196** | Freesurfer DKT | 27168 | Area of superiorfrontal (left hemisphere) | No | No |
| **196** | Freesurfer DKT | 27169 | Area of superiorparietal (left hemisphere) | No | No |
| **196** | Freesurfer DKT | 27170 | Area of superiortemporal (left hemisphere) | No | No |
| **196** | Freesurfer DKT | 27171 | Area of supramarginal (left hemisphere) | No | No |
| **196** | Freesurfer DKT | 27172 | Area of transversetemporal (left hemisphere) | No | No |
| **196** | Freesurfer DKT | 27173 | Area of insula (left hemisphere) | No | No |
| **196** | Freesurfer DKT | 27174 | Mean thickness of caudalanteriorcingulate (left hemisphere) | No | No |
| **196** | Freesurfer DKT | 27175 | Mean thickness of caudalmiddlefrontal (left hemisphere) | No | No |
| **196** | Freesurfer DKT | 27176 | Mean thickness of cuneus (left hemisphere) | No | No |
| **196** | Freesurfer DKT | 27177 | Mean thickness of entorhinal (left hemisphere) | No | No |
| **196** | Freesurfer DKT | 27178 | Mean thickness of fusiform (left hemisphere) | No | No |
| **196** | Freesurfer DKT | 27179 | Mean thickness of inferiorparietal (left hemisphere) | No | No |
| **196** | Freesurfer DKT | 27180 | Mean thickness of inferiortemporal (left hemisphere) | No | No |
| **196** | Freesurfer DKT | 27181 | Mean thickness of isthmuscingulate (left hemisphere) | No | No |
| **196** | Freesurfer DKT | 27182 | Mean thickness of lateraloccipital (left hemisphere) | No | No |
| **196** | Freesurfer DKT | 27183 | Mean thickness of lateralorbitofrontal (left hemisphere) | No | No |
| **196** | Freesurfer DKT | 27184 | Mean thickness of lingual (left hemisphere) | No | No |
| **196** | Freesurfer DKT | 27185 | Mean thickness of medialorbitofrontal (left hemisphere) | No | No |
| **196** | Freesurfer DKT | 27186 | Mean thickness of middletemporal (left hemisphere) | No | No |
| **196** | Freesurfer DKT | 27187 | Mean thickness of parahippocampal (left hemisphere) | No | No |
| **196** | Freesurfer DKT | 27188 | Mean thickness of paracentral (left hemisphere) | No | No |
| **196** | Freesurfer DKT | 27189 | Mean thickness of parsopercularis (left hemisphere) | No | No |
| **196** | Freesurfer DKT | 27190 | Mean thickness of parsorbitalis (left hemisphere) | No | No |
| **196** | Freesurfer DKT | 27191 | Mean thickness of parstriangularis (left hemisphere) | No | No |
| **196** | Freesurfer DKT | 27192 | Mean thickness of pericalcarine (left hemisphere) | No | No |
| **196** | Freesurfer DKT | 27193 | Mean thickness of postcentral (left hemisphere) | No | No |
| **196** | Freesurfer DKT | 27194 | Mean thickness of posteriorcingulate (left hemisphere) | No | No |
| **196** | Freesurfer DKT | 27195 | Mean thickness of precentral (left hemisphere) | No | No |
| **196** | Freesurfer DKT | 27196 | Mean thickness of precuneus (left hemisphere) | No | No |
| **196** | Freesurfer DKT | 27197 | Mean thickness of rostralanteriorcingulate (left hemisphere) | No | No |
| **196** | Freesurfer DKT | 27198 | Mean thickness of rostralmiddlefrontal (left hemisphere) | No | No |
| **196** | Freesurfer DKT | 27199 | Mean thickness of superiorfrontal (left hemisphere) | No | No |
| **196** | Freesurfer DKT | 27200 | Mean thickness of superiorparietal (left hemisphere) | No | No |
| **196** | Freesurfer DKT | 27201 | Mean thickness of superiortemporal (left hemisphere) | No | No |
| **196** | Freesurfer DKT | 27202 | Mean thickness of supramarginal (left hemisphere) | No | No |
| **196** | Freesurfer DKT | 27203 | Mean thickness of transversetemporal (left hemisphere) | No | No |
| **196** | Freesurfer DKT | 27204 | Mean thickness of insula (left hemisphere) | No | No |
| **196** | Freesurfer DKT | 27205 | Volume of caudalanteriorcingulate (left hemisphere) | No | No |
| **196** | Freesurfer DKT | 27206 | Volume of caudalmiddlefrontal (left hemisphere) | No | No |
| **196** | Freesurfer DKT | 27207 | Volume of cuneus (left hemisphere) | No | No |
| **196** | Freesurfer DKT | 27208 | Volume of entorhinal (left hemisphere) | No | No |
| **196** | Freesurfer DKT | 27209 | Volume of fusiform (left hemisphere) | No | No |
| **196** | Freesurfer DKT | 27210 | Volume of inferiorparietal (left hemisphere) | No | No |
| **196** | Freesurfer DKT | 27211 | Volume of inferiortemporal (left hemisphere) | No | No |
| **196** | Freesurfer DKT | 27212 | Volume of isthmuscingulate (left hemisphere) | No | No |
| **196** | Freesurfer DKT | 27213 | Volume of lateraloccipital (left hemisphere) | No | No |
| **196** | Freesurfer DKT | 27214 | Volume of lateralorbitofrontal (left hemisphere) | No | No |
| **196** | Freesurfer DKT | 27215 | Volume of lingual (left hemisphere) | No | No |
| **196** | Freesurfer DKT | 27216 | Volume of medialorbitofrontal (left hemisphere) | No | No |
| **196** | Freesurfer DKT | 27217 | Volume of middletemporal (left hemisphere) | No | No |
| **196** | Freesurfer DKT | 27218 | Volume of parahippocampal (left hemisphere) | No | No |
| **196** | Freesurfer DKT | 27219 | Volume of paracentral (left hemisphere) | No | No |
| **196** | Freesurfer DKT | 27220 | Volume of parsopercularis (left hemisphere) | No | No |
| **196** | Freesurfer DKT | 27221 | Volume of parsorbitalis (left hemisphere) | No | No |
| **196** | Freesurfer DKT | 27222 | Volume of parstriangularis (left hemisphere) | No | No |
| **196** | Freesurfer DKT | 27223 | Volume of pericalcarine (left hemisphere) | No | No |
| **196** | Freesurfer DKT | 27224 | Volume of postcentral (left hemisphere) | No | No |
| **196** | Freesurfer DKT | 27225 | Volume of posteriorcingulate (left hemisphere) | No | No |
| **196** | Freesurfer DKT | 27226 | Volume of precentral (left hemisphere) | No | No |
| **196** | Freesurfer DKT | 27227 | Volume of precuneus (left hemisphere) | No | No |
| **196** | Freesurfer DKT | 27228 | Volume of rostralanteriorcingulate (left hemisphere) | No | No |
| **196** | Freesurfer DKT | 27229 | Volume of rostralmiddlefrontal (left hemisphere) | No | No |
| **196** | Freesurfer DKT | 27230 | Volume of superiorfrontal (left hemisphere) | No | No |
| **196** | Freesurfer DKT | 27231 | Volume of superiorparietal (left hemisphere) | No | No |
| **196** | Freesurfer DKT | 27232 | Volume of superiortemporal (left hemisphere) | No | No |
| **196** | Freesurfer DKT | 27233 | Volume of supramarginal (left hemisphere) | No | No |
| **196** | Freesurfer DKT | 27234 | Volume of transversetemporal (left hemisphere) | No | No |
| **196** | Freesurfer DKT | 27235 | Volume of insula (left hemisphere) | No | No |
| **196** | Freesurfer DKT | 27236 | Area of caudalanteriorcingulate (right hemisphere) | No | No |
| **196** | Freesurfer DKT | 27237 | Area of caudalmiddlefrontal (right hemisphere) | No | No |
| **196** | Freesurfer DKT | 27238 | Area of cuneus (right hemisphere) | No | No |
| **196** | Freesurfer DKT | 27239 | Area of entorhinal (right hemisphere) | No | No |
| **196** | Freesurfer DKT | 27240 | Area of fusiform (right hemisphere) | No | No |
| **196** | Freesurfer DKT | 27241 | Area of inferiorparietal (right hemisphere) | No | No |
| **196** | Freesurfer DKT | 27242 | Area of inferiortemporal (right hemisphere) | No | No |
| **196** | Freesurfer DKT | 27243 | Area of isthmuscingulate (right hemisphere) | No | No |
| **196** | Freesurfer DKT | 27244 | Area of lateraloccipital (right hemisphere) | No | No |
| **196** | Freesurfer DKT | 27245 | Area of lateralorbitofrontal (right hemisphere) | No | No |
| **196** | Freesurfer DKT | 27246 | Area of lingual (right hemisphere) | No | No |
| **196** | Freesurfer DKT | 27247 | Area of medialorbitofrontal (right hemisphere) | No | No |
| **196** | Freesurfer DKT | 27248 | Area of middletemporal (right hemisphere) | No | No |
| **196** | Freesurfer DKT | 27249 | Area of parahippocampal (right hemisphere) | No | No |
| **196** | Freesurfer DKT | 27250 | Area of paracentral (right hemisphere) | No | No |
| **196** | Freesurfer DKT | 27251 | Area of parsopercularis (right hemisphere) | No | No |
| **196** | Freesurfer DKT | 27252 | Area of parsorbitalis (right hemisphere) | No | No |
| **196** | Freesurfer DKT | 27253 | Area of parstriangularis (right hemisphere) | No | No |
| **196** | Freesurfer DKT | 27254 | Area of pericalcarine (right hemisphere) | No | No |
| **196** | Freesurfer DKT | 27255 | Area of postcentral (right hemisphere) | No | No |
| **196** | Freesurfer DKT | 27256 | Area of posteriorcingulate (right hemisphere) | No | No |
| **196** | Freesurfer DKT | 27257 | Area of precentral (right hemisphere) | No | No |
| **196** | Freesurfer DKT | 27258 | Area of precuneus (right hemisphere) | No | No |
| **196** | Freesurfer DKT | 27259 | Area of rostralanteriorcingulate (right hemisphere) | No | No |
| **196** | Freesurfer DKT | 27260 | Area of rostralmiddlefrontal (right hemisphere) | No | No |
| **196** | Freesurfer DKT | 27261 | Area of superiorfrontal (right hemisphere) | No | No |
| **196** | Freesurfer DKT | 27262 | Area of superiorparietal (right hemisphere) | No | No |
| **196** | Freesurfer DKT | 27263 | Area of superiortemporal (right hemisphere) | No | No |
| **196** | Freesurfer DKT | 27264 | Area of supramarginal (right hemisphere) | No | No |
| **196** | Freesurfer DKT | 27265 | Area of transversetemporal (right hemisphere) | No | No |
| **196** | Freesurfer DKT | 27266 | Area of insula (right hemisphere) | No | No |
| **196** | Freesurfer DKT | 27267 | Mean thickness of caudalanteriorcingulate (right hemisphere) | No | No |
| **196** | Freesurfer DKT | 27268 | Mean thickness of caudalmiddlefrontal (right hemisphere) | No | No |
| **196** | Freesurfer DKT | 27269 | Mean thickness of cuneus (right hemisphere) | No | No |
| **196** | Freesurfer DKT | 27270 | Mean thickness of entorhinal (right hemisphere) | No | No |
| **196** | Freesurfer DKT | 27271 | Mean thickness of fusiform (right hemisphere) | No | No |
| **196** | Freesurfer DKT | 27272 | Mean thickness of inferiorparietal (right hemisphere) | No | No |
| **196** | Freesurfer DKT | 27273 | Mean thickness of inferiortemporal (right hemisphere) | No | No |
| **196** | Freesurfer DKT | 27274 | Mean thickness of isthmuscingulate (right hemisphere) | No | No |
| **196** | Freesurfer DKT | 27275 | Mean thickness of lateraloccipital (right hemisphere) | No | No |
| **196** | Freesurfer DKT | 27276 | Mean thickness of lateralorbitofrontal (right hemisphere) | No | No |
[truncated: 475,144 more chars]
