# Supplementary figures and images for: Transforming and evaluating the UK Biobank to the OMOP Common Data Model for COVID-19 research and beyond
Source: J Am Med Inform Assoc. 2022 Oct 13;30(1):103–11. doi: 10.1093/jamia/ocac203 (PMC9619789; doi:10.1093/jamia/ocac203)

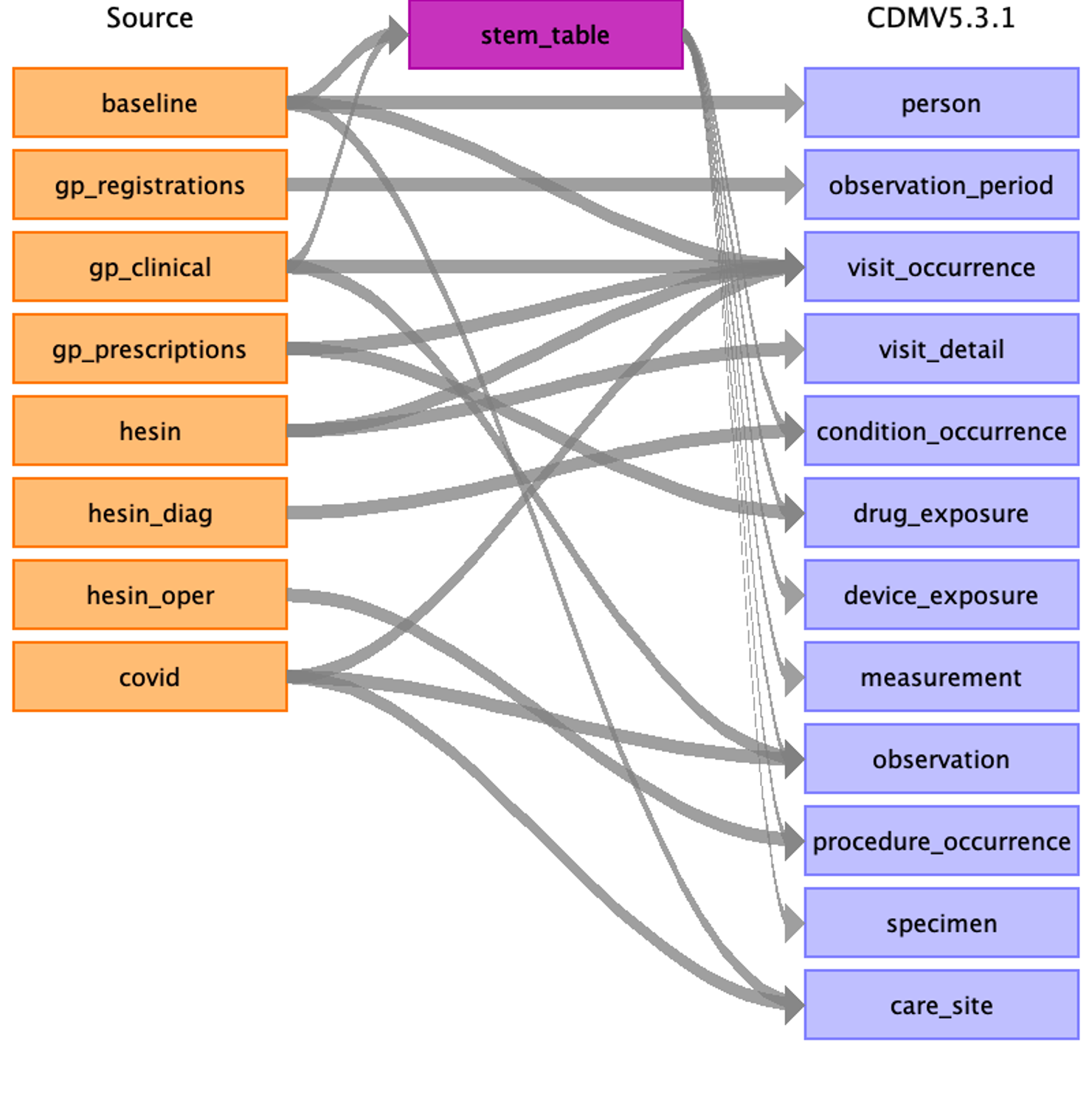

Supplement: ocac203_Supplementary_Data [file ocac203_supplementary_data.zip › ocac203_Supplementary_Data/Supplementary_Figure_1.png]

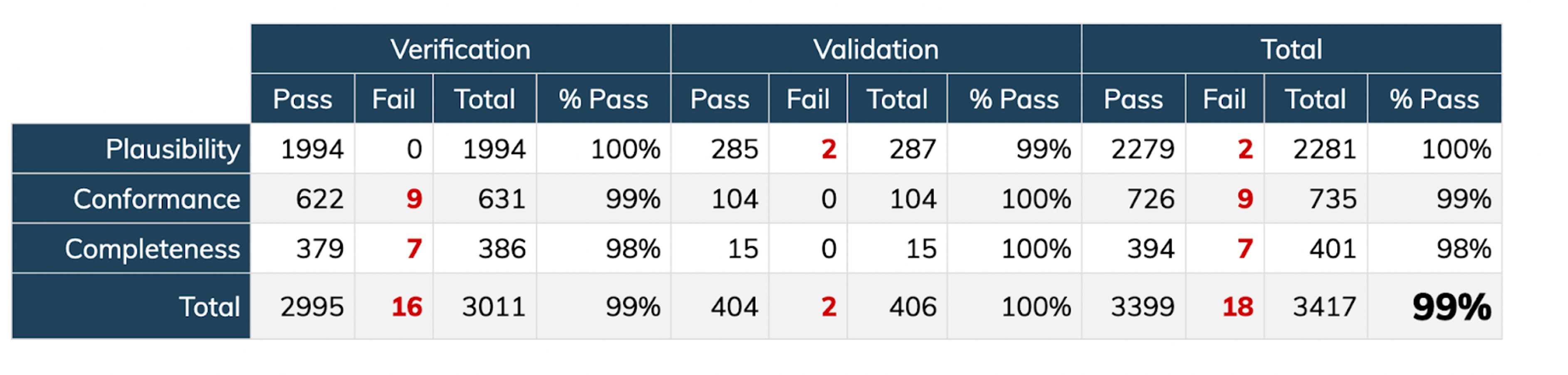

Supplement: ocac203_Supplementary_Data [file ocac203_supplementary_data.zip › ocac203_Supplementary_Data/Supplementary_Figure_2.png]

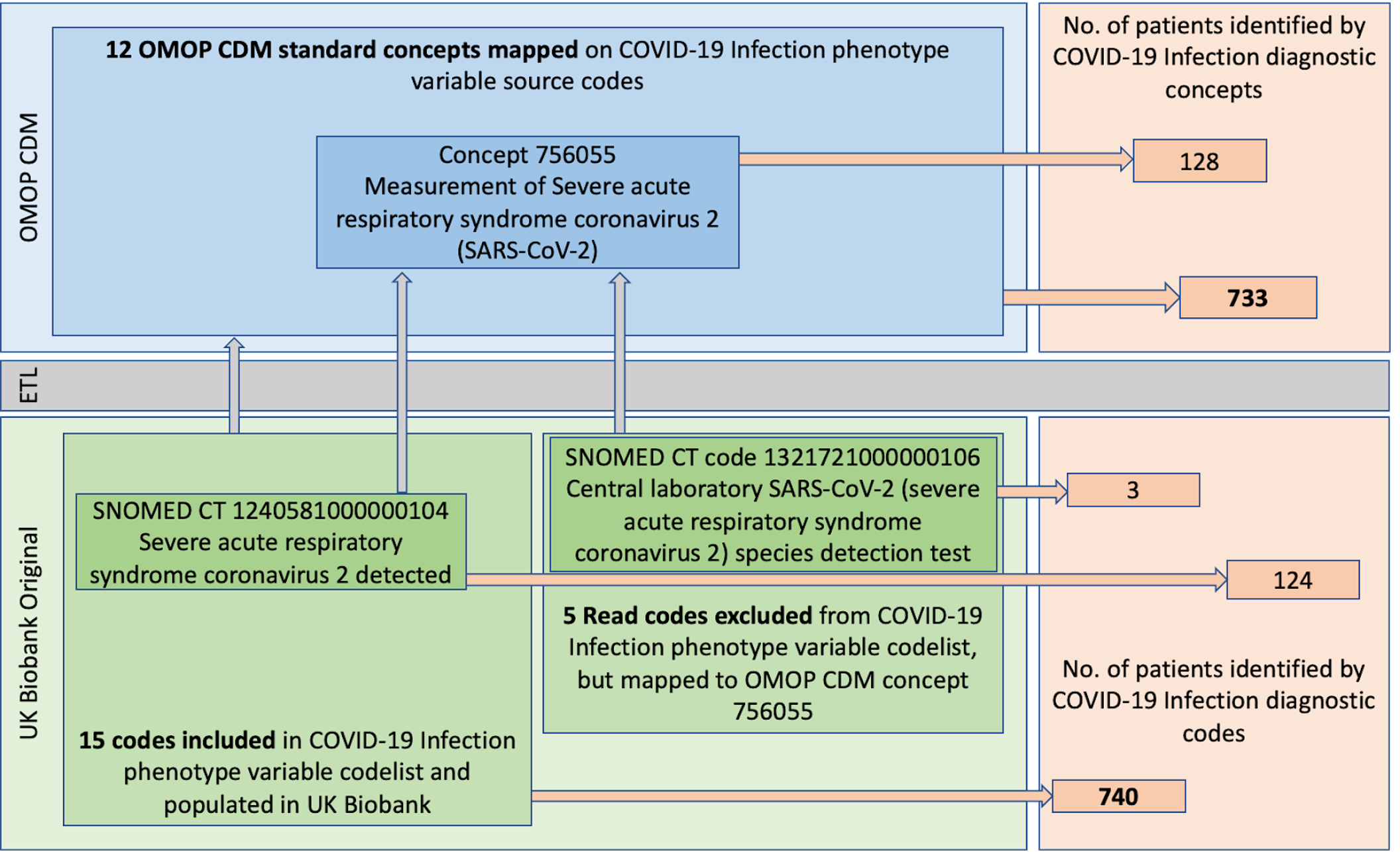

Supplement: ocac203_Supplementary_Data [file ocac203_supplementary_data.zip › ocac203_Supplementary_Data/Supplementary_Figure_3.png]
